# Synthesis of enones, pyrazolines and pyrrolines with *gem*-difluoroalkyl side chains

**DOI:** 10.3762/bjoc.9.230

**Published:** 2013-09-26

**Authors:** Assaad Nasr El Dine, Ali Khalaf, Danielle Grée, Olivier Tasseau, Fares Fares, Nada Jaber, Philippe Lesot, Ali Hachem, René Grée

**Affiliations:** 1 Université de Rennes 1, Institut des Sciences Chimiques de Rennes, CNRS UMR 6226, Avenue du Général Leclerc, 35042 Rennes Cedex, France; 2 Laboratory for Medicinal Chemistry and Natural Products, Lebanese University, Faculty of Sciences (1) and PRASE-EDST, Hadath, Beyrouth, Lebanon; 3 RMN en Milieu Orienté, ICMMO, CNRS UMR 8182, Université de Paris Sud, 91405 Orsay cedex, France

**Keywords:** ^19^F/^1^H HOESY, *gem*-difluoroalkyl derivatives, organo-fluorine compounds, palladium catalysis, pyrazolines, pyrrolines

## Abstract

Starting from easily accessible *gem*-difluoropropargylic derivatives, a DBU-mediated isomerisation affords enones in fair yields with a *gem*-difluoroalkyl chain. These derivatives were used to prepare pyrazolines and pyrrolines with the desired *gem*-difluoroalkyl side chain by cyclocondensations in good yields and with excellent stereoselectivity. A one-pot process was also successfully developed for these sequential reactions. By carrying out various types of Pd-catalyzed coupling reactions for compounds with a *p*-bromophenyl substituent a route to focused chemical libraries was demonstrated.

## Introduction

**Scheme 1:**
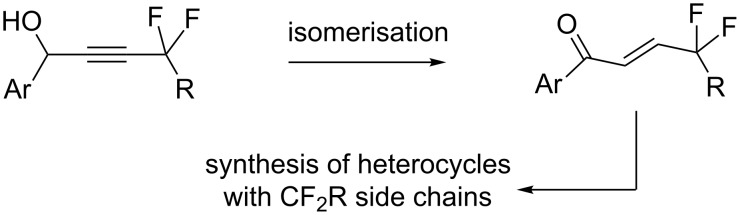
Strategy towards the target molecules.

A widely used strategy in bioorganic, medicinal chemistry and in chemical biology is the selected introduction of fluorine in organic molecules since it strongly modifies their properties [1–9]. On the other hand, heterocyclic molecules – in particular, the so-called privileged scaffolds – are introduced very classically in the core of pharmaceutical products [10–11]. Therefore, it appears to be of much interest to design novel methodologies for the preparation of new fluorinated heterocyclic molecules. We developed a programme to investigate the preparation and uses of new propargylic fluorides [12–14], which have been employed in the synthesis of fluorinated analogues of lipids [15–16] and in carbocyclic systems [17–18]. They have also been used for the preparation of several 5 and 6-membered heterocycles [19–22]. The goal of the present work is to demonstrate that selected propargylic derivatives [23–25] can be employed for the preparation of enones with a *gem*-difluoroalkyl chain by using an isomerisation process (Scheme 1).

These intermediates can be employed for the preparation of representative 5-membered heterocyclic systems with CF_2_R side chains by using cyclocondensation reactions. Furthermore, selected molecules in these series were functionalized by using appropriate palladium-catalyzed coupling reactions en route to chemical libraries.

## Results and Discussion

The first example of a base-mediated isomerisation process for an alkyne activated by an ester group was reported by Nineham and Raphael in 1949 [26]. Later, extension to other electrophilic alkynes was demonstrated by Sonye and Koide [27]. Recently, it has been established by Yamazaki’s group that propargylic alcohols bearing a CF_3_ group on the triple bond could be isomerised to the corresponding enones. In that case, Et_3_N proved to be sufficient as a catalyst to perform this transformation [28].

**Scheme 2:**
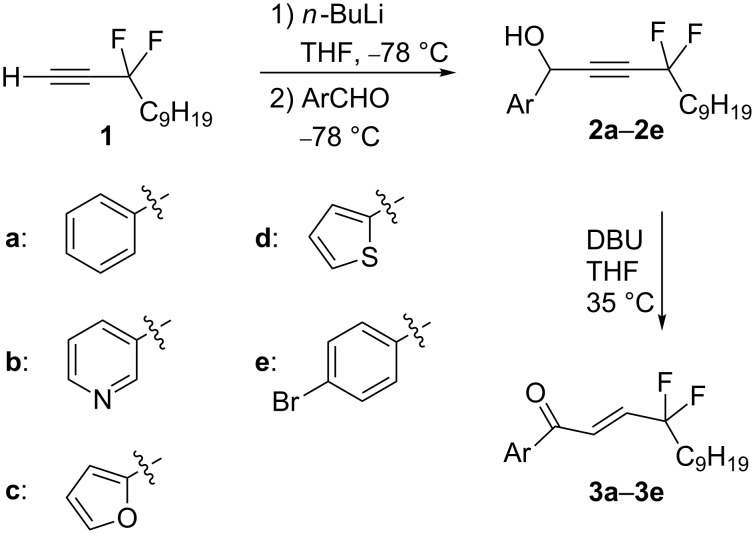
Synthesis of enones with a *gem*-difluoroalkyl side chain.

The required starting propargylic alcohols were obtained by a reaction of the lithium salt of easily available *gem*-difluoro propargylic derivative **1** [18] with aromatic aldehydes, affording compounds **2a**–**2e** in 71–82% yields (Scheme 2 and Table 1). With these *gem*-difluoro intermediates, Et_3_N was not an efficient catalyst since only a low conversion was observed and the reaction was not clean. On the contrary, the DBU-mediated isomerisation was successful, affording the desired enones **3a**–**3e** in 60–63% yields. The selectivity was excellent since in all cases the *E*-isomer was obtained almost exclusively (>98%). Similar reactions were carried out with propargylic derivatives bearing alkyl groups instead of the (Ar) aromatic or heteroaromatic group, but these reactions were not successful. Therefore, this reaction appears limited to derivatives with aryl or heteroaryl substituents as in the case of the CF_3_-substituted propargylic derivatives [28].

**Table 1:** Synthesis of enones **3a**–**3e**.

Entry	Ar	Step 1 Yield (%)	Step 2 Yield (%)

1	Ph	**2a** (82)	**3a** (62)
2	C_5_H_4_N	**2b** (78)	**3b** (62)
3	C_4_H_3_O	**2c** (79)	**3c** (63)
4	C_4_H_3_S	**2d** (71)	**3d** (60)
5	*p*-PhBr	**2e** (81)	**3e** (61)

**Scheme 3:**
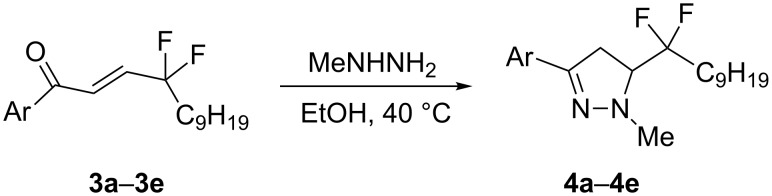
Synthesis of pyrazolines with a *gem*-difluoro side chain.

Next, we turned towards the preparation of heterocyclic structures from enones **3**. Pyrazolines are well-recognized heterocyclic cores for pharmacologically active molecules [29]. Therefore, they were selected as first examples of 5-membered heterocyclic targets with the fluorinated side chain. Reaction of **3a**–**3e** with methylhydrazine gave the desired pyrazolines **4a**–**4e** in 79–86% yields (Scheme 3).

**Scheme 4:**
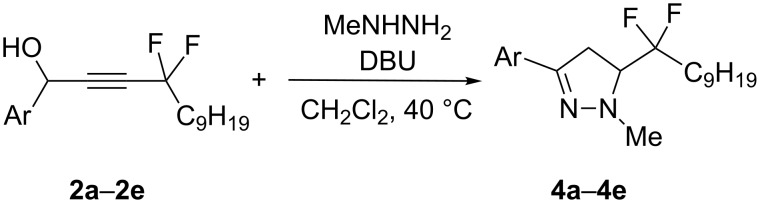
One-pot synthesis of pyrazolines with a *gem*-difluoro side chain.

It appeared that the reaction conditions for the synthesis of these pyrazolines were compatible with the first isomerisation step, therefore the possibility of a "one-pot" reaction was considered. Indeed, by heating a mixture of propargylic alcohols **2a**–**2e** with DBU (1,8-diazabicycloundec-7-ene) in the presence of methylhydrazine (Scheme 4) the pyrazolines **4a**–**4e** were obtained after 3–7 h in excellent yields (82–92%, Table 2). This very short and efficient synthesis of pyrazolines **4** can be related to another excellent one-pot reaction with 3-components where the first step is a Pd-catalyzed coupling–isomerisation process followed by a cyclocondensation [30].

**Table 2:** Synthesis of pyrazolines **4a**–**4e**.

Entry	Ar	Yield (%) (**3** to **4**)	Yield (%) (**1** to **4**)

1	Ph	**4a** (85)	**4a** (86)
2	C_5_H_4_N	**4b** (79)	**4b** (82)
3	C_4_H_3_O	**4c** (81)	**4c** (84)
4	C_4_H_3_S	**4d** (82)	**4d** (83)
5	*p*-PhBr	**4e** (86)	**4e** (92)

**Scheme 5:**
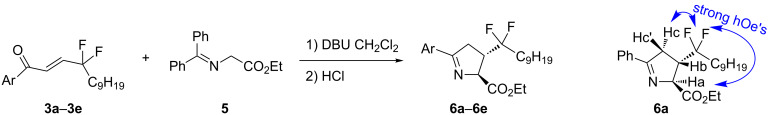
Synthesis of pyrrolines with a *gem*-difluoro alkyl side chain.

Pyrroline is another noteworthy example of a heterocyclic scaffold useful in bioorganic and medicinal chemistry [31–32]. It is also well-recognized for agrochemicals, especially in combination with CF_3_ substituents. Recently, the group of Shibata has developed an elegant organocatalyzed asymmetric approach to such pyrrolines [33]. Moreover, an efficient synthesis of β-trifluoromethylated Δ^1^-pyrrolines has been reported [34]. Therefore, we selected pyrrolines with CF_2_R side chains as a second example of 5-membered heterocyclic targets. Condensation of the anion of glycine ester diphenylimine **5** with the enones **3a**–**3e** afforded the desired pyrrolines **6a**–**6e** (Scheme 4 and Table 3). In all cases good yields were obtained, and a complete selectivity for the *trans*-isomer was observed as established by NMR analysis of the crude reaction mixtures. This is different from the results obtained by starting from enones with CF_2_CF_2_X side chains, where *trans*/*cis* mixtures were reported [34]. The ^3^*J*_HH_ (6.3–6.5 Hz, *trans*) of our pyrrolines were very close to those of similar molecules bearing CF_2_–CF_3_ chains (6.4–6.6 Hz), while for latter derivatives the *cis* coupling constants were larger (≥ 8.3 Hz) [34]. This was confirmed in the case of pyrroline **6a** by performing additional ^19^F/^1^H hOe 2D experiments which revealed strong correlations between the fluorine atoms of the CF_2_ group and the cyclic protons H_c_ and H_a_ (see Scheme 5 and 2D spectrum in the Supporting Information File 1 for details).

**Table 3:** Synthesis of pyrrolines **6a**–**6e**.

Entry	Ar	Yield (%) (**3** to **6**)	Yield (%) (**1** to **6**)

1	Ph	**6a** (74)	**6a** (73)
2	C_5_H_4_N	**6b** (73)	**6b** (76)
3	C_4_H_3_O	**6c** (75)	**6c** (73)
4	C_4_H_3_S	**6d** (74)	**6d** (71)
5	*p*-PhBr	**6e** (78)	**6e** (75)

**Scheme 6:**
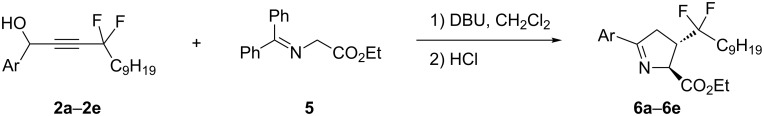
One-pot synthesis of pyrrolines with a *gem*-difluoro side chain.

The reaction conditions were compatible with both steps and an example of a one-pot reaction was performed starting from **2a**–**2e**. The desired pyrrolines **6a–6e** were obtained in excellent yields (71–76%, Scheme 6 and Table 3).

Another important issue was the possibility of using these molecules as scaffolds for the preparation of focused chemical libraries. In order to explore this possibility we developed representative examples of Pd-catalyzed reactions starting from *p*-bromo derivatives **4e** and **6e**.

**Scheme 7:**
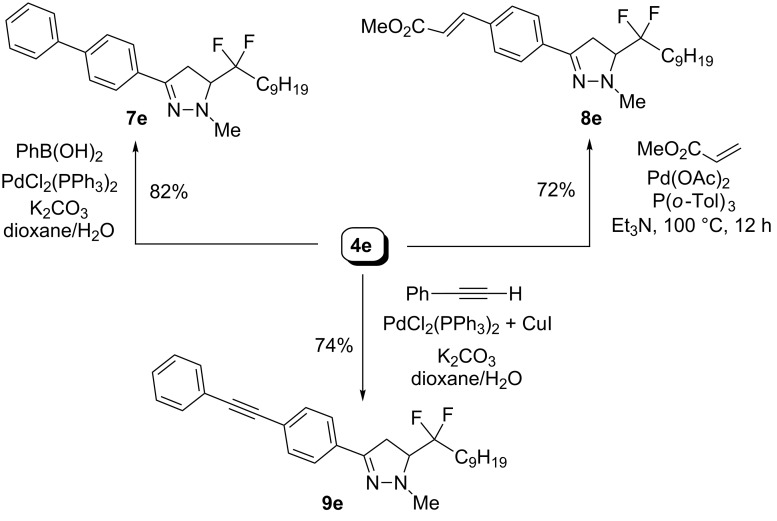
Pd-catalyzed coupling reactions towards chemical libraries of pyrazolines with a *gem*-difluoro side chain.

**Scheme 8:**
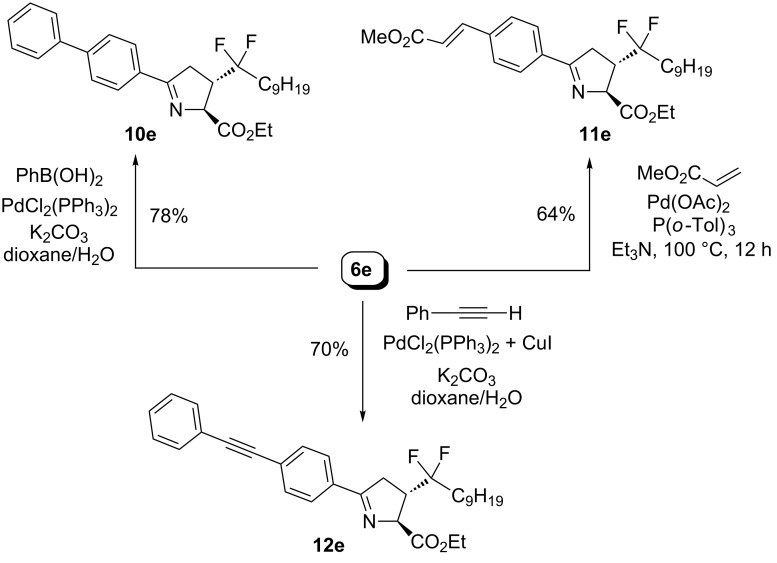
Pd-catalyzed coupling reactions towards chemical libraries of pyrrolines with a *gem-*difluoro side chain.

The results are given in Scheme 7 for pyrazoline **4e**. Suzuki–Miyaura coupling [35] gave biphenyl derivative **7e** in 82% yield, while the Heck [36] and Sonogashira [37] reactions afforded also the desired targets **8e** and **9e** in 72% and 77% yield respectively. Similar results were obtained in Pd-mediated reactions starting from pyrroline **6e**, as indicated in Scheme 8. The desired molecules **10e**–**12e** were obtained in good yields.

## Conclusion

In summary, we developed an efficient access to enones with *gem*-difluoroalkyl side chains through a base-mediated isomerisation of fluorinated propargylic alcohols. Although this method is, to date, limited to compounds with aryl or heteroaryl substituents, corresponding enones appear as versatile intermediates for the preparation of heterocyclic derivatives. This has been established through the synthesis of pyrazolines and pyrrolines with *gem*-difluoroalkyl side chains. With appropriate substituents, derivatives of this type can be used for the preparation of chemical libraries.

## Supporting Information

File 1 Experimental details, NMR analysis and characterization data of new compounds.
