# Supplementary material for: Synthesis of enones, pyrazolines and pyrrolines with gem-difluoroalkyl side chains
Source: Beilstein J Org Chem. 2013 Sep 26;9:1943–8. doi: 10.3762/bjoc.9.230 (PMC3817575; doi:10.3762/bjoc.9.230)
Supplement: File 1 — Experimental details, NMR analysis and characterization data of new compounds. [file Beilstein_J_Org_Chem-09-1943-s001.pdf]

Supporting Information

for

Synthesis of enones, pyrazolines and pyrrolines with *gem*-difluoroalkyl side chains

Assaad Nasr El Dine[1,2], Ali Khalaf[1,2], Danielle Grée[1], Olivier Tasseau[1],

Fares Fares[2], Nada Jaber[2], Philippe Lesot[3], Ali Hachem*[2] and René Grée*[1]

Address: [1]Université de Rennes 1, Institut des Sciences Chimiques de Rennes, CNRS UMR 6226, Avenue du Général Leclerc, 35042 Rennes Cedex, France, [2]Laboratory for Medicinal Chemistry and Natural Products, Lebanese University, Faculty of Sciences (1) and PRASE-EDST, Hadath, Beyrouth, Lebanon and [3]RMN en Milieu Orienté, ICMMO, CNRS UMR 8182, Université de Paris Sud, 91405 Orsay cedex, France

Email: René Grée* - rene.gree@univ-rennes1.fr; Ali Hachem* - ahachem@ul.edu.lb

*Corresponding author

Experimental details, NMR analysis and characterization data of new compounds

General Methods S4
1. General procedure for the synthesis of difluoropropargylic alcohols (**2**) S4
1.1. Synthesis of 4,4-difluoro-1-phenyl-tridec-2-yn-1-ol ((±)-**2a**) S5
1.2. Synthesis of 4,4-difluoro-1-(pyridine-3-yl)-tridec-2-yn-1-ol ((±)-**2b**) S5
1.3. Synthesis of 4,4-difluoro-1-(furane-2-yl)-tridec-2-yn-1-ol ((±)-**2c**) S5
1.4. Synthesis of 4,4-difluoro-1-(thiophene-2-yl)-tridec-2-yn-1-ol ((±)-**2d**) S6
1.5. Synthesis of 1-(4-bromophenyl)-4,4-difluoro-tridec-2-yn-1-ol ((±)-**2e**) S6
2. General procedure for the synthesis of *gem*-difluoroenones (**3**) S7
2.1. Synthesis of 4,4-difluoro-1-phenyl-tridec-2-en-1-one (**3a**) S7
2.2. Synthesis of 4,4-difluoro-1-(pyridine-3-yl)-tridec-1-en-one (**3b**) S7
2.3. Synthesis of 4,4-difluoro-1-(furane-2-yl)-tridec-2-en-1-one (**3c**) S8
2.4. Synthesis of 4,4-difluoro-1-(thiophene-2-yl)-tridec-2-en-1-one (**3d**) S8
2.5. Synthesis of 1-(4-bromophenyl)-4,4-difluoro-tridec-2-en-1-one (**3e**) S9
3. General procedure for the synthesis of pyrazolines (**4**) S9
3.1. Synthesis of 5-(1,1-difluorodecyl)-1-methyl-3-phenyl-4,5-dihydro-1*H*-pyrazole ((±)-**4a**) S9
3.2. Synthesis 3-[5-(1,1-difluorodecyl)-1-methyl-4,5-dihydro-1*H*-pyrazole-3-yl]pyridine ((±)-**4b**) S10
3.3. Synthesis of 5-(1,1-difluorodecyl)-3-furan-2-yl-1-methyl-4,5-dihydro-1*H*-pyrazole ((±)-**4c**) S10
3.4. Synthesis of 5-(1,1-difluorodecyl)-1-methyl-3-(thiophen-2-yl)-4,5-dihydro-1*H*-pyrazole ((±)-**4d**) S11
3.5. Synthesis of 3-(4-bromophenyl)-5-(1,1-difluorodecyl)-1-methyl-4,5-dihydro-1*H*-pyrazole ((±)-**4e**) S12
4. General procedure for the synthesis of pyrrolines (**6**) S13
4.1. Synthesis of 3-(1,1-difluorodecyl)-5-phenyl-3,4-dihydro-2*H*-pyrrole-2-carboxylic acid ethyl ester ((±)-**6a**) S13
4.2. Synthesis of 3-(1,1-Difluorodecyl)-5-(pyridin-3-yl)-3,4-dihydro-2*H*-pyrrole-2-carboxylic acid ethyl ester ((±)-**6b**) S13
4.3. Synthesis of 3-(1,1-difluorodecyl)-5-(furan-2-yl)-3,4-dihydro-2*H*-pyrrole-2-carboxylic acid ethyl ester ((±)-**6c**) S14
4.4. Synthesis of 3-(1,1-difluorodecyl)-5-(thiophen-2-yl)-3,4-dihydro-2*H*-pyrrole-2-carboxylic acid ethyl ester ((±)-**6d**) S15
4.5. Synthesis of 5-(4-bromophenyl)-3-(1,1-difluorodecyl)-3,4-dihydro-2*H*-pyrrole-2-carboxylic acid ethyl ester ((±)-**6e**) S15

5. General procedure for the Suzuki–Miyaura reaction S16
5.1. Synthesis of 3-biphenyl-4-yl-5(1,1-difluorodecyl)-1-methyl-4,5-dihydro-1*H*-pyrazole ((±)-**7e**) S16
5.2. Synthesis of 5-biphenyl-4-yl-3-(1,1-difluorodecyl)-3,4-dihydro-2*H*-pyrrole-2-carboxylic acid ethyl ester ((±)-**10e**) S16
6. General procedure for the Heck reaction S17
6.1. Synthesis of 3-{4-[5-(1,1-difluorodecyl)-1-methyl-4,5-dihydro-1*H*-pyrazol-3-yl]-phenyl}-acrylic acid methyl ester ((±)-**8e**) S17
6.2. Synthesis of 3-(1,1-difluorodecyl)-5-[4-(2-methoxycarbonyl-vinyl)-phenyl]-3,4-dihydro-2*H*-pyrrole-2-carboxylic acid ethyl ester ((±)-**11e**) S18
7. General procedure for the Sonogashira reaction S18
7.1. Synthesis of 5-(1,1-difluorodecyl)-1-methyl-3-(4-phenylethynyl-phenyl)-4,5-dihydro-1*H*-pyrazole ((±)-**9e**) S18
7.2. Synthesis of 3-(1,1-difluorodecyl)-5-(4-phenylethynyl-phenyl)-3,4-dihydro-2*H*-pyrrole-2-carboxylic acid ethyl ester ((±)-**12e**) S19
8. Analysis of $^{19}F/^{1}H$ HOESY 2D-NMR spectrum of (±)-**6a** S20
9. Library of ^{1}H, ^{13}C-{^{1}H}, ^{19}F-{^{1}H} and ^{19}F 1D-NMR spectra S21

*Note: all compounds **2** and **4–12** are prepared in racemic form only.*

General Methods

All reagents were obtained commercially and used without further purification. All reactions were carried out under a nitrogen atmosphere and dry conditions. The solvents used were freshly distilled under anhydrous conditions, unless otherwise specified. The reaction mixtures were magnetically stirred with Teflon stirring bars, and the temperatures were measured externally. Reactions that required anhydrous conditions were carried out by using oven dried (120 °C, 24 h) glassware. Yields refer to chromatographically and spectroscopically (^{1}H, ^{13}C, and ^{19}F NMR) homogeneous materials. Reactions were monitored by ^{19}F NMR and by thin-layer chromatography (TLC), carried out on 0.25 mm Merck silica gel plates (60 F254) with detection by UV light or staining with *p*-anisaldehyde. The eluents used were mixtures of pentane (Pent) and ether (Et_2O). Acros silica gel (60, particle size 0.040–0.063 mm) was used for column chromatography. Nuclear magnetic resonance (NMR) spectra have been recorded with Bruker Avance 500, 400 and 300 spectrometers. ^{1}H NMR spectra: δ (H) are given in ppm relative to tetramethylsilane (TMS), using [δ ($CHCl_3$) = 7.26 ppm] as internal reference. ^{13}C NMR spectra: δ (C) are given in ppm relative to TMS, using [δ ($CDCl_3$) = 77.0 ppm] as internal reference. ^{19}F NMR spectra: δ (F) are given in ppm relative to $CFCl_3$ = 0.0 ppm as external reference. Multiplicities were designated as singlet (s), doublet (d), triplet (t), quadruplet (q), quintuplet (qt), multiplet (m) or br (broad). Mass spectral analyses have been performed at the Centre Régional de Mesures Physiques de l'Ouest (CRMPO) in Rennes (France).

1. General procedure for the synthesis of difluoropropargylic alcohols (2)

To a solution of propargylic *gem*-difluoro intermediate **1** (1 equiv) in anhydrous THF (2 ml per mmol) cooled at −78 °C, was added, dropwise under nitrogen, a solution of *n*-BuLi in hexanes (1.2 equiv). The mixture was stirred for 1 h at $T \leq -40$ °C. Then the aldehyde (1.2 equiv) in anhydrous THF (1 ml per mmol) was added at −78 °C and the resulting solution was allowed to warm to room temperature over 2 h. The mixture was treated with saturated ammonium chloride solution and extracted with ether (3 x 10 ml). The combined organic phases were washed with water, dried over $MgSO_4$ and concentrated in vacuo. The crude product is purified by chromatography on silica gel, using a mixture of ether/pentane as eluent.

1.1. Synthesis of 4,4-difluoro-1-phenyl-tridec-2-yn-1-ol ((±)-2a)

The reaction was performed with **1** (990 mg, 4.90 mmol) and benzaldehyde (0.6 ml, 9.8 mmol), according to the general procedure. After purification by chromatography on silica gel, propargylic alcohol **2a** was obtained as a yellow oil (1.30 g, 82% yield). $\mathbf{R}_f$ = 0.38 (Et_2O/pentane 1/9). ^1H NMR ($CDCl_3$, 300 MHz), δ ppm: 7.54-7.41 (m, 2H); 7.38-7.27 (m, 3H); 5.47 (t, 1H, $^5J_{HF}$ = 3.9 Hz); 2.19 (bs, 1H); 2.13-1.97 (m, 2H); 1.60-1.52 (m, 2H); 1.30-1.27 (m, 12H); 0.89 (t, 3H, J = 6.7 Hz). ^{13}C NMR ($CDCl_3$, 75 MHz), δ ppm: 139.0 (t, $^5J_{CF}$ = 1.4 Hz); 128.9 (2C); 126.6 (2C); 114.9 (t, $^1J_{CF}$ = 232.8 Hz); 86.3 (t, $^3J_{CF}$ = 6.7 Hz); 79.7 (t, $^2J_{CF}$ = 41.2 Hz); 64.3 (t, $^4J_{CF}$ = 1.9 Hz); 39.5 (t, $^2J_{CF}$ = 25.7 Hz); 31.9; 29.4; 29.32; 29.26; 28.9; 22.7 (t, $^3J_{CF}$ = 3.5 Hz); 22.7; 14.1. ^{19}F NMR ($CDCl_3$, 282 MHz), δ ppm: -83.10 (td, J_{FH} = 15.0 Hz, 3.9 Hz). HRMS (ESI) calcd for $C_{19}H_{26}F_2O^{79}BrNa$: $[M+Na]^+$: *m/z* 331.19518. Found*: m/z* 331.1953 (1ppm).

1.2. Synthesis of 4,4-difluoro-1-(pyridine-3-yl)-tridec-2-yn-1-ol ((±)-2b)

The reaction was performed with **1** (400 mg, 1.29 mmol) and 3-pyridinecarboxaldehyde (0.22 ml, 2.38 mmol), according to the general procedure. After purification by chromatography on silica gel, propargylic alcohol **2b** was obtained as a yellow oil (472 mg, 78% yield). $\mathbf{R}_f$ = 0.29 (Et_2O/pentane 7/3). ^1H NMR ($CDCl_3$, 300 MHz), δ ppm: 8.66 (s, 1H); 8.54 (d, 1H, J = 4.4 Hz); 7.92 (d, 1H, J = 7.4 Hz); 7.38 (dd, 1H, J = 7.4 Hz, J = 4.4 Hz) ; 5.62 (t, 1H, $^5J_{HF}$ = 3.8 Hz); 3.80 (bs, 1H); 2.13-1.92 (m, 2H); 1.61-1.43 (m, 2H); 1.16-1.45 (m, 12H); 0.88 (t, H_1, J = 6.6 Hz). ^{13}C NMR ($CDCl_3$, 75 MHz), δ ppm: 148.6; 147.3; 136.3; 135.2; 123.9; 114.8 (t, $^1J_{CF}$ = 232.9 Hz); 86.1 (t, $^3J_{CF}$ = 6.8 Hz); 79.6 (t, $^2J_{CF}$ = 41.2 Hz); 61.3; 39.1 (t, $^2J_{CF}$ = 25.7 Hz); 31.8; 29.4; 29.3; 29.2; 28.9; 22.7 (t, $^3J_{CF}$ = 3.4 Hz); 22.7; 14.1. ^{19}F NMR ($CDCl_3$, 282 MHz), δ ppm: -83.23 (td, J_{FH} = 15.3 Hz, 3.8 Hz). HRMS (ESI) calcd for $C_{22}H_{32}NOF_2{}^{79}BrNa$: $[M+Na]^+$: *m/z* 332.18019. Found*: m/z* 332.1812 (3 ppm).

1.3. Synthesis of 4,4-difluoro-1-(furane-2-yl)-tridec-2-yn-1-ol ((±)-2c)

The reaction was performed with **1** (400 mg, 1.29 mmol) and furane-2-carbaldehyde (0.2 ml, 2.38 mmol), according to the general procedure. After purification by chromatography on silica gel, propargylic alcohol **2c** was obtained as a yellow oil (463 mg, 79% yield). $\mathbf{R}_f$ = 0.46 (Et_2O/pentane 2/8). ^1H NMR ($CDCl_3$, 300 MHz), δ ppm: 7.44 (dd, 1H, J = 1.8 Hz, J = 0.8

Hz); 6.47 (dt, 1H, J = 3.3 Hz, J = 0.7 Hz); 6.38 (dd, 1H, J = 3.3 Hz, J = 1.8 Hz); 5.54 (t, 1H, $^5J_{HF}$ = 3.8 Hz); 2.47 (bs, 1H); 2.19-1.98 (m, 2H); 1.61-1.42 (m, 2H); 1.15-1.34 (m, 12H); 0.88 (t, H_1, J = 6.3 Hz). ^{13}C NMR ($CDCl_3$, 75 MHz), δ ppm: 151.4 (t, $^5J_{CF}$ = 2.0 Hz); 143.4; 114.8 (t, $^1J_{CF}$ = 233.1 Hz); 110.6; 108.4; 83.9 (t, $^3J_{CF}$ = 6.7 Hz); 78.8 (t, $^2J_{CF}$ = 41.4 Hz); 57.8 (t, $^4J_{CF}$ = 1.8 Hz); 39.1 (t, $^2J_{CF}$ = 25.6 Hz); 31.8; 29.4; 29.30; 29.2; 28.9; 22.7 (t, $^3J_{CF}$ = 3.5 Hz); 22.7; 14.1. ^{19}F NMR ($CDCl_3$, 282 MHz), δ ppm: -83.51 (td, J_{FH} = 15.2 Hz, 3.8 Hz). HRMS (ESI) calcd for $C_{17}H_{24}O_2F_2{}^{79}BrNa$: [M + Na]$^+$: *m/z* 321.16421. Found*: m/z* 321.1641 (0 ppm).

1.4. Synthesis of 4,4-difluoro-1-(thiophene-2-yl)-tridec-2-yn-1-ol ((±)-2d)

The reaction was performed with **1** (877 mg, 4.34 mmol) and thiophene-2-carbaldehyde (0.47 ml, 5.21 mmol), according to the general procedure. After purification by chromatography on silica gel, propargylic alcohol **2d** was obtained as a yellow oil (966 mg, 71% yield). $\mathbf{R_f}$ = 0.43 (Et_2O/pentane 2/8). ^{1}H NMR ($CDCl_3$, 300 MHz), δ ppm: 7.24 (dd, 1H, J = 5.1 Hz, J = 1.2 Hz); 7.18 (dt, 1H, J = 3.4 Hz, 0.9 Hz); 6.38 (dd, 1H, J = 5.1 Hz, J = 3.4 Hz); 5.62 (bs, 1H); 2.72 (bs, 1H); 2.18-1.84 (m, 2H); 1.61-1.42 (m, 2H); 1.37-1.17 (m, 12H); 0.83 (t, H_1, J = 6.7 Hz). ^{13}C NMR ($CDCl_3$, 75 MHz), δ ppm: 142.7 (t, $^5J_{CF}$ = 1.7 Hz); 126.9; 126.6; 126.1; 114.9 (t, $^1J_{CF}$ = 233.2 Hz); 85.6 (t, $^3J_{CF}$ = 6.7 Hz); 79.1 (t, $^2J_{CF}$ = 41.4 Hz); 59.9 (t, $^4J_{CF}$ = 1.8 Hz); 39.1 (t, $^2J_{CF}$ = 25.6 Hz); 31.9; 29.4; 29.33; 29.27; 29.0; 22.7 (t, $^3J_{CF}$ = 3.8 Hz); 22.7; 14.1. ^{19}F NMR ($CDCl_3$, 282 MHz), δ ppm: -83.45 (td, J_{FH} = 15.0 Hz, 3.8 Hz). HRMS (ESI) calcd for $C_{17}H_{24}OF_2S^{79}BrNa$: [M +Na]$^+$: m/z 377.14136. Found: m/z 337.1416 (1 ppm).

1.5. Synthesis of 1-(4-bromophenyl)-4,4-difluoro-tridec-2-yn-1-ol ((±)-2e)

The reaction was performed with **1** (400 mg, 1.98 mmol) and 4-bromobenzaldehyde (0.44 mg, 2.376 mmol), according to the general procedure. After purification by chromatography on silica gel, propargylic alcohol **2e** was obtained as a yellow oil (614 mg, 81% yield). $\mathbf{R_f}$ = 0.34 (Et_2O/pentane 1/9). ^{1}H NMR ($CDCl_3$, 300 MHz), δ ppm: 7.41-7.32 (m, 2H); 7.25-7.16 (m, 2H); 5.28 (t, 1H, $^5J_{HF}$ = 3.7 Hz); 2.46 (bs, 1H); 2.02-1.88 (m, 2H); 1.50-1.35 (m, 2H); 1.29-1.11 (m, 12H); 0.80 (t, H_1, J = 6.4 Hz). ^{13}C NMR ($CDCl_3$, 75 MHz), δ ppm: 138.0; 131.9; 128.3; 122.9; 114.8 (t, $^1J_{CF}$ = 233.0 Hz); 85.8 (t, $^3J_{CF}$ = 6.7 Hz); 79.9 (t, $^2J_{CF}$ = 41.3 Hz); 63.4 (t, $^4J_{CF}$ = 1.8 Hz); 39.1 (t, $^2J_{CF}$ = 25.7 Hz); 32.0; 29.43; 29.35; 29.30; 28.9; 22.7 (t, $^3J_{CF}$ = 3.5 Hz); 22.7; 14.1. ^{19}F NMR ($CDCl_3$, 282 MHz), δ ppm: -83.25 (td, J_{FH} = 15.1 Hz, 3.7

Hz). HRMS (ESI) calcd for $C_{19}H_{25}OF_2Br^{79}BrNa$: $[M +Na]^+$: *m/z* 409.09545. Found*: m/z* 409.0951 (2 ppm).

2. General procedure for the synthesis of *gem*-difluoroenones (3)

The difluoropropargylic alcohol **2** (1 equiv) was dissolved in THF (2 ml per mmol), then DBU (1.5 equiv) was added, and stirred at 35 °C for the appropriate time (monitored by ^{19}F NMR). After completion of the reaction, the mixture was neutralized with a saturated solution of NH_4Cl. After extraction with diethyl ether, the organic phases were washed with water, dried ($MgSO_4$) and concentrated in vacuo. The crude product was purified by chromatography on silica gel, using ether/pentane as eluent.

2.1. Synthesis of 4,4-difluoro-1-phenyl-tridec-2-en-1-one (3a)

The reaction was performed with **2a** (920 mg, 2.99 mmol) according to the general procedure. After 6 h, ^{19}F NMR showed 100% conversion, and there were two enones, *cis* and *trans* (1/100). After purification by flash chromatography on silica gel, the enone **3a** was isolated as a yellow oil (570 mg, 62%). $\mathbf{R}_f$ = 0.30 (Et_2O/pentane 2/98). ^1H NMR ($CDCl_3$, 400 MHz), δ ppm: 8.02-7.94 (m, 2H); 7.66-7.57 (m, 1H); 7.55-7.46 (m, 2H); 7.31 (dt, 1H, J = 15.6 Hz, $^4J_{HF}$ = 2.3 Hz); 6.85 (dt, 1H, J = 15.6 Hz, $^3J_{HF}$ = 11.6 Hz); 2.08-1.91 (m, 2H); 1.51-1.43 (m, 2H); 1.41-1.17 (m, 12H); 0.88 (t, 3H, J = 6.7 Hz). ^{13}C NMR ($CDCl_3$, 100 MHz), δ ppm: 189.3; 138.9 (t, $^2J_{CF}$ = 27.3 Hz); 133.6; 136.9; 128.8 (2C); 128.7 (2C); 127.40 (t, $^3J_{CF}$ = 7.5 Hz); 121.3 (t, $^1J_{CF}$ = 239.9 Hz); 37.3 (t, $^2J_{CF}$ = 25.7 Hz); 31.8; 29.4; 29.3; 29.2 (2C); 22.7; 22.2 (t, $^3J_{CF}$ = 4.1 Hz); 14.1. ^{19}F NMR ($CDCl_3$, 282 MHz), δ ppm: -98.36 (tdd, J_{FH} = 16.0 Hz, 11.6 Hz, 2.3Hz). HRMS (ESI) calcd for $C_{19}H_{26}F_2O^{79}BrNa$: $[M +Na]^+$: *m/z* 331.19518. Found*: m/z* 331.1952 (0ppm).

2.2. Synthesis of 4,4-difluoro-1-(pyridine-3-yl)-tridec-1-en-one (3b)

The reaction was performed with **2b** (386 mg, 1.25 mmol) according to the general procedure. After 30 min, ^{19}F NMR showed 100% conversion, and that a single product is formed. After purification by flash chromatography on silica gel, the enone **3b** was isolated as a sticky syrup (225 mg, 62%). $\mathbf{R}_f$ = 0.26 (Et_2O/pentane 3/7). ^1H NMR ($CDCl_3$, 300 MHz), δ ppm: 9.16 (dd, 1H, J = 2.1 Hz, J = 0.5 Hz); 8.80 (dd, 1H, J = 4.8 Hz, J = 1.6 Hz); 8.23 (ddd,

1H, J = 7.9 Hz, J = 2.1 Hz, J = 1.6 Hz); 7.44 (ddd, 1H, J = 7.9 Hz, J = 4.8 Hz, J = 0.5 Hz); 7.26 (dt, 1H, J = 15.5 Hz, $^4J_{HF}$ = 2.3 Hz); 6.88 (dt, 1H, J = 15.5 Hz, $^3J_{HF}$ = 11.9 Hz); 2.06-1.90 (m, 2H); 1.51-1.40 (m, 2H); 1.39-1.12 (m, 12H); 0.85 (t, 3H, J = 6.7 Hz). ^{13}C NMR ($CDCl_3$, 75 MHz), δ ppm: 188.0; 153.8; 149.9; 140.0 (t, $^2J_{CF}$ = 27.5 Hz); 135.9; 132.1; 126.6 (t, $^3J_{CF}$ = 7.5 Hz); 123.7; 121.0 (t, $^1J_{CF}$ = 239.9 Hz); 37.1 (t, $^2J_{CF}$ = 25.6 Hz); 31.8; 29.3; 29.2; 29.1 (2C); 22.6; 22.1 (t, $^3J_{CF}$ = 4.1 Hz); 14.0. ^{19}F NMR ($CDCl_3$, 282 MHz), δ ppm: -98.63 (td, J_{FH} = 15.4 Hz, 11.9 Hz). HRMS (ESI) calcd for $C_{18}H_{25}NOF_2{}^{79}BrNa$: [M +Na]$^+$: *m/z* 332.18019 Found*: m/z* 332.1814 (4 ppm).

2.3. Synthesis of 4,4-difluoro-1-(furane-2-yl)-tridec-2-en-1-one (3c)

The reaction was performed with **2c** (386 mg, 1.25 mmol) according to the general procedure. After 40 min, ^{19}F NMR showed 100% conversion, and there were two enones, *cis* and *trans* (1/100). After purification by flash chromatography on silica gel, the enone **3c** was isolated as yellow oil (138 mg, 63%). $\mathbf{R}_f$ = 0.31 (Et_2O/pentane 5/95). ^{1}H NMR ($CDCl_3$, 300 MHz), δ ppm: 7.67 (dd, 1H, J = 1.7 Hz, J = 0.7 Hz); 7.33 (dd, 1H, J = 3.7 Hz, J = 0.7 Hz); 7.18 (dt, 1H, J = 15.6 Hz, $^4J_{HF}$ = 2.2 Hz); 6.92 (dt, 1H, J = 15.6 Hz, $^3J_{HF}$ = 11.5 Hz); 6.59 (dd, 1H, J = 3.6 Hz, J = 1.7 Hz); 2.08-1.85 (m, 2H); 1.55-1.39 (m, 2H); 1.35-1.14 (m, 12H); 0.85 (t, 3H, J = 6.7 Hz). ^{13}C NMR ($CDCl_3$, 75 MHz), δ ppm: 176.7; 152.9; 147.4; 138.4 (t, $^2J_{CF}$ = 27.5 Hz); 126.7 (t, $^3J_{CF}$ = 7.6 Hz); 121.2 (t, $^1J_{CF}$ = 239.7 Hz); 119.0; 112.8; 37.24 (t, $^2J_{CF}$ = 25.7 Hz); 31.82; 29.37; 29.3; 29.2 (2C); 22.6; 22.1 (t, C_8, $^3J_{CF}$ = 4.1 Hz); 14.1. ^{19}F NMR ($CDCl_3$, 282 MHz), δ ppm: -98.48 (tdd, J_{FH} = 16.1 Hz, 11.5 Hz, 2.1 Hz). HRMS (ESI) calcd for $C_{17}H_{24}O_2F_2{}^{79}BrNa$: [M +Na]$^+$: *m/z* 321.16421. Found*: m/z* 321.1640 (1 ppm).

2.4. Synthesis of 4,4-difluoro-1-(thiophene-2-yl)-tridec-2-en-1-one (3d)

The reaction was performed with **2d** (730 mg, 2.32 mmol) according to the general procedure. After 1 h, ^{19}F NMR showed 100% conversion, and there were two enones, *cis* and *trans* (1/100). After purification by flash chromatography on silica gel, the enone **3c** was isolated as a yellow oil (437 mg, 60%). $\mathbf{R}_f$ = 0.24 (Et_2O/pentane 2/98). ^{1}H NMR ($CDCl_3$, 300 MHz), δ ppm: 7.83 (dd, 1H, J = 3.9 Hz, J = 1.1 Hz); 7.73 (dd, 1H, J = 5.0 Hz, J = 1.1 Hz); 7.19 (dt, 1H, J = 15.4 Hz, $^4J_{HF}$ = 2.3 Hz); 7.18 (dd, 1H, J = 5.0 Hz, J = 3.9 Hz); 6.89 (dt, 1H, J = 15.4 Hz, $^3J_{HF}$ = 11.6 Hz); 2.09-1.90 (m, 2H); 1.52-1.43 (m, 2H); 1.38-1.18 (m, 12H); 0.87

(t, 3H, J = 6.2 Hz). ^{13}C NMR ($CDCl_3$, 75 MHz), δ ppm: 181.0; 144.4; 138.4 (t, $^{2}J_{CF}$ = 27.2 Hz); 135.2; 133.0; 128.5; 127.1 (t, $^{3}J_{CF}$ = 7.5 Hz); 121.2 (t, $^{1}J_{CF}$ = 239.7 Hz); 37.3 (t, $^{2}J_{CF}$ = 25.7 Hz); 31.8; 29.4; 29.3; 29.2 (2C); 22.7; 22.1 (t, $^{3}J_{CF}$ = 4.1 Hz); 14.1. ^{19}F NMR ($CDCl_3$, 282 MHz), δ ppm: -98.45 (tdd, J_{FH} = 16.1 Hz, 11.6 Hz, 2.3 Hz). HRMS (ESI) calcd for $C_{17}H_{24}OF_2S^{79}BrNa$: [M +Na]$^+$: *m/z* 377.14136. Found*: m/z* 337.1418 (1 ppm).

2.5. Synthesis of 1-(4-bromophenyl)-4,4-difluoro-tridec-2-en-1-one (3e)

The reaction was performed with **2e** (332 mg, 1.07 mmol) according to the general procedure. After 1 h, ^{19}F NMR showed 100% conversion, and there were two enones, *cis* and *trans* (1/100). After purification by flash chromatography on silica gel, the enone **3c** was isolated as a sticky syrup (203 mg, 61%). $\mathbf{R}_f$ = 0.31 (pentane). ^{1}H NMR ($CDCl_3$, 300 MHz), δ ppm: 7.86-7.62 (m, 4H); 7.26 (dt, 1H , J = 15.5 Hz, $^{4}J_{HF}$ = 2.3 Hz); 6.86 (dt, 1H, J = 15.5 Hz, $^{3}J_{HF}$ = 11.5 Hz); 2.07-1.89 (m, 2H); 1.53-1,41 (m, 2H, H_8); 1.40-1.2 (m, 12H); 0.87 (t, 3H, J = 6.7 Hz). ^{13}C NMR ($CDCl_3$, 75 MHz), δ ppm: 188.2; 139.5 (t, $^{2}J_{CF}$ = 27.3 Hz); 135.60; 132.2 (2C); 130.2 (2C); 128.9; 126.8 (t, $^{3}J_{CF}$ = 7.5 Hz); 121.1(t, $^{1}J_{CF}$ = 239.8 Hz); 37.3 (t, $^{2}J_{CF}$ = 25.7 Hz); 31.9; 29.4; 29.3; 29.2 (2C); 22.7; 22.2 (t, $^{3}J_{CF}$ = 4.1 Hz); 14.1. ^{19}F NMR ($CDCl_3$, 282 MHz), δ ppm: -98.47 (td, J_{FH} = 16.1 Hz, 11.5 Hz, 2.3 Hz). HRMS (ESI) calcd for $C_{19}H_{25}OF_2Br^{79}BrNa$: [M +Na]$^+$: *m/z* 409.09545. Found*: m/z* 409.0952 (1 ppm).

3. General procedure for the synthesis of pyrazolines (4)

The enone **3** (1 equiv) was dissolved in ethanol (2 ml per 1 mmol), then methylhydrazine (4 equiv) was added. The reaction mixture was stirred at 40 °C for the appropriate time (monitored by ^{19}F NMR). After evaporation of ethanol, the crude product was crystallized and the pyrazoline **4** was purified by chromatography on silica gel, using ether/pentane as eluent.

3.1. Synthesis of 5-(1,1-difluorodecyl)-1-methyl-3-phenyl-4,5-dihydro-1H-pyrazole ((±)-4a)

The reaction was performed with **3a** (100 mg, 0.33 mmol) according to the general procedure. After 11.5 h, ^{19}F NMR monitoring showed the absence of starting material. After purification by flash chromatography on silica gel, the pyrazoline **4a** was isolated as a sticky syrup (90 mg, 85%). $\mathbf{R}_f$ = 0.47 ((Et_2O/pentane 1/9). ^{1}H NMR ($CDCl_3$, 400 MHz), δ ppm: 7.72-7.62 (m, 2H); 7.34-7.33 (m, 3H); 3.54 (dddd, 1H, $^{3}J_{HF}$ = 11.7 Hz, J = 11.6 Hz, J = 11.6

Hz, $^3J_{HF}$ = 7.4 Hz); 3.36 (dd, 1H, J = 16.8 Hz, J = 11.6 Hz); 3.07 (dd, 1H, J = 16.8 Hz, J = 11.6 Hz); 2.99 (s, 3H); 2.05-1.82 (m, 2H); 1.62-1.54 (m, 2H); 1.38-1.22 (m, 12H); 0.89 (t, 3H, J = 6.7 Hz). ^{13}C NMR ($CDCl_3$, 100 MHz); δ ppm: 149.3; 132.1; 128.9; 128.8 (2C); 127.1 (2C); 124.7 (t, $^1J_{CF}$ = 251.9 Hz); 71.19 (dd, $^2J_{CF}$ = 27.5 Hz, $^2J_{CF}$ = 26.6 Hz); 43.9; 35.8 (dd, $^3J_{CF}$ = 5.3 Hz, $^3J_{CF}$ = 2.0 Hz); 33.0 (t, $^2J_{CF}$ = 24.2 Hz); 31.9; 29.43; 29.40; 29.3; 22.7; 22.2 (t, $^3J_{CF}$ = 4.2 Hz); 14.1. ^{19}F NMR ($CDCl_3$, 282 MHz), δ ppm: -102.11(dddd, J_{FF} = 250.6Hz, J_{FH} = 20.8 Hz, 15.6 Hz, 11.7 Hz); -104.33 (dddd, J_{FF} = 250.6 Hz, J_{FH} = 22.1 Hz, 16.5 Hz, 7.4 Hz). HRMS (ESI) calcd for $C_{20}H_{30}F_2N_2{}^{79}BrNa$: $[M+Na]^+$: *m*/z 359.22747. Found*: m/z* 359.2274 (0 ppm).

3.2. Synthesis 3-[5-(1,1-difluorodecyl)-1-methyl-4,5-dihydro-1*H*-pyrazole-3-yl]-pyridine ((±)-4b)

The reaction was performed with **3b** (100 mg, 0.29 mmol) according to the general procedure. After 2.5 h, ^{19}F NMR monitoring showed the absence of starting material. After purification by flash chromatography on silica gel, the pyrazoline **4b** was isolated as yellow crystals (88 mg, 79%). Mp: 51-53 °C. $\mathbf{R}_f$ = 0.44 (Et_2O/pentane 5/5). ^{1}H NMR ($CDCl_3$, 300 MHz), δ ppm: 8.72 (s, 1H); 8.50 (d, 1H, J = 3.8 Hz); 7.93 (dt, 1H, J = 8.0 Hz, J = 1.6 Hz); 7.23 (dd, 1H, J = 8.0 Hz, J = 3.8 Hz); 3.51 (dddd, 1H, J = 11.9 Hz, J = 11.7 Hz, $^3J_{HF}$ = 11.7 Hz, $^3J_{HF}$ = 6.6 Hz); 3.26 (dd, 1H, J = 16.9 Hz, J = 11.7 Hz); 2.94 (dd, 1H, J = 16.9 Hz, J = 11.9 Hz); 2.93 (s, 3H); 2.03-1.66 (m, 2H); 1.56-1.41 (m, 2H); 1.34-1.09 (m, 12H); 0.80 (t, 3H, J = 6.7 Hz). ^{13}C NMR ($CDCl_3$, 75 MHz), δ ppm: 149.6; 147.1; 146.1; 132.8; 128.2; 124.36 (t, C_{10}, $^1J_{CF}$ = 242.8 Hz); 123.4; 71.1 (dd, $^2J_{CF}$ = 27.9 Hz, $^2J_{CF}$ = 27.2 Hz); 43.67 (t, J = 1.5 Hz); 35.2 (dd, $^3J_{CF}$ = 5.1 Hz, $^3J_{CF}$ = 2.6 Hz); 32.90 (t, $^2J_{CF}$ = 24.3 Hz); 31.2; 29.39 (2C); 29.36 ; 29.2; 22.6; 21.1 (t, $^3J_{CF}$ = 4.0 Hz) ; 14.1. ^{19}F NMR ($CDCl_3$, 282 MHz), δ ppm: -102.55 (ddt, J_{FF} = 250.7 Hz, J_{FH} = 26.5 Hz, 11.7 Hz); -104.54 (dddd, J_{FF} = 250.7 Hz, J_{FH} = 26.3 Hz, 10.4 Hz, 6.6 Hz). HRMS (ESI) calcd for $C_{19}H_{29}F_2N_3{}^{79}BrNa$: $[M+H]^+$: *m/z* 338.24078. Found*: m/z* 338.2408 (0 ppm).

3.3. Synthesis of 5-(1,1-difluorodecyl)-3-furan-2-yl-1-methyl-4,5-dihydro-1*H*-pyrazole ((±)-4c)

The reaction was performed with **3c** (58 mg, 0.17 mmol) according to the general procedure. After 2 h 35 min, ^{19}F NMR monitoring showed the absence of starting material. After purification by flash chromatography on silica gel, the pyrazoline **4c** was isolated as white

crystals (54 mg, 81%). Mp: 68-70 °C. **R**$_f$ = 0.31 (Et_2O/pentane 1/9). ^{1}H NMR ($CDCl_3$, 300 MHz), δ ppm: 7.44 (dd, 1H, , *J* = 1.8 Hz, 0.7 Hz); 6.52 (dd, 1H, *J* = 3.4 Hz, *J* = 0.7 Hz); 6.43 (dd, 1H, *J* = 3.4 Hz, *J* = 1.8 Hz); 3.50 (dddd, 1H, *J* = 11.9 Hz, $^3J_{HF}$ = 11,9 Hz, *J* = 11.8 Hz , $^3J_{HF}$ = 6.2 Hz); 3.24 (dd, 1H, *J* = 16.9 Hz, *J* = 11.9 Hz); 3.00 (dd, 1H, *J* = 16.9 Hz, *J* = 12.1 Hz); 2.87 (s, 3H); 2.02-1.77 (m, 2H); 1.61-1.49 (m, 2H); 1.39-1.19 (m, 12H); 0.85 (t, 3H, *J* = 6.7 Hz). ^{13}C NMR ($CDCl_3$, 125 MHz), δ ppm: 147.5; 143.5; 141.3; 124.5 (t, $^1J_{CF}$ = 242.8 Hz); 111.5; 109.8; 123.4; 71.9 (dd, $^2J_{CF}$ = 28.5 Hz, $^2J_{CF}$ = 27.5 Hz); 43.9; 35.7 (dd, $^3J_{CF}$ = 5.4 Hz, $^3J_{CF}$ = 2.1 Hz); 32.9 (t, $^2J_{CF}$ = 24.3 Hz); 31.9; 29.43 (2C); 29.39; 29.2; 22.7; 21.1 (t, $^3J_{CF}$ = 4.0 Hz); 14.1. ^{19}F NMR ($CDCl_3$, 282 MHz), δ ppm: -102.05 (ddt, J_{FF} = 251.6 Hz, J_{FH} = 27.3 Hz, 11.9 Hz); -104.17 (dddd, J_{FF} = 251.6 Hz, J_{FH} = 25.8 Hz, 11.6 Hz, 6.2 Hz). HRMS (ESI) calcd for $C_{18}H_{28}F_2N_2O^{79}BrNa$: [M +Na]$^+$: *m/z* 349.20674. Found*: m/z* 349.2067 (0 ppm).

3.4. Synthesis of 5-(1,1-difluorodecyl)-1-methyl-3-(thiophen-2-yl)-4,5-dihydro-1*H*-pyrazole ((±)-4d)

The reaction was performed with **3d** (155 mg, 0.45 mmol) according to the general procedure. After 4 h 35 min, ^{19}F NMR monitoring showed the absence of starting material. After purification by flash chromatography on silica gel, the pyrazoline **4d** was isolated as white crystals (137 mg, 82%). Mp: 67-69 °C. **R**$_f$ = 0.29 (Et_2O/pentane 5/95). ^{1}H NMR ($CDCl_3$, 300 MHz), δ ppm: 7.28 (dd, 1H, *J* = 5.0 Hz, 1.2 Hz); 7.06 (dd, 1H, *J* = 3.6 Hz, *J* = 1.2 Hz); 7.00 (dd, 1H, *J* = 5.0 Hz, *J* = 3.6 Hz); 3.52 (dddd, 1H, *J* = 11.9 Hz, *J* = 11.5 Hz, $^3J_{HF}$ = 11.5 Hz, $^3J_{HF}$ = 6.7 Hz); 3.31 (dd, 1H, *J* = 16.7 Hz, *J* = 11.5 Hz); 3.05 (dd, 1H, *J* = 16.7 Hz, *J* = 11.9 Hz); 2.89 (s, 3H); 2.09-1.76 (m, 2H); 1.65-1.49 (m, 2H); 1.43-1.23 (m, 12H); 0.88 (t, 3H, *J* = 6.7 Hz). ^{13}C NMR ($CDCl_3$, 75 MHz), δ ppm: 145.1; 135.8; 127.2; 126.8; 126.3; 124.5 (t, $^1J_{CF}$ = 242.6 Hz); 71.6 (dd, $^2J_{CF}$ = 28.5 Hz, $^2J_{CF}$ = 27.6 Hz); 43.8 (t, ^3J = 1.4 Hz); 36.5 (dd, $^3J_{CF}$ = 5.4 Hz, $^3J_{CF}$ = 2.4 Hz); 32.8 (t, $^2J_{CF}$ = 24.2 Hz); 31.2; 29.37 (2C); 29.34; 29.2; 22.6; 21.0 (t, $^3J_{CF}$ = 4.0 Hz); 14.0. ^{19}F NMR ($CDCl_3$, 282 MHz), δ ppm: -101.92 (ddt, J_{FF} = 250.9 Hz, J_{FH} = 27.9 Hz, 11.5 Hz); -104.21 (dddd, J_{FF} = 250.9 Hz, J_{FH} = 27.5 Hz, 11.2 Hz, 6.7 Hz). HRMS (ESI) calcd for $C_{18}H_{28}F_2N_2S^{79}BrNa$: [M +Na]$^+$: *m/z* 365.18390. Found*: m/z* 365.1849 (3 ppm).

3.5. Synthesis of 3-(4-bromophenyl)-5-(1,1-difluorodecyl)-1-methyl-4,5-dihydro-1*H*-pyrazole ((±)-4e)

The reaction was performed with **3e** (75 mg, 0.18 mmol) according to the general procedure. After 2.5 h, ^{19}F NMR monitoring showed the absence of starting material. After purification by flash chromatography on silica gel, the pyrazoline **4e** was isolated as a sticky syrup (74 mg, 86%). $\mathbf{R}_f$ = 0.43 (Et_2O/pentane 5/95). ^{1}H NMR ($CDCl_3$, 300 MHz), δ ppm: 7.47 (s, 4H); 3.55 (dddd, 1H, J = 12.0 Hz, J = 11.8 Hz, $^3J_{HF}$ = 11.8 Hz, $^3J_{HF}$ = 6.6 Hz); 3.29 (dd, 1H, J = 16.9 Hz, J = 11.8 Hz); 3.00 (dd, 1H, J = 16.9 Hz, J = 12.0 Hz); 2.99 (s, 3H,) ; 2.08-1.75 (m, 2H); 1.64-1.49 (m, 2H); 1.43-1.18 (m, 12H); 0.88 (t, 3H, J = 6.7 Hz). ^{13}C NMR ($CDCl_3$, 75 MHz), δ ppm: 148.0; 131.7 (2C); 131.1; 127.7 (2C); 124.5 (t, $^1J_{CF}$ = 242.6 Hz); 123.0; 71.3 (dd, $^2J_{CF}$ = 28.5 Hz, $^2J_{CF}$ = 27.5 Hz); 43.8 (t, 1.4 Hz); 35.6 (dd, $^3J_{CF}$ = 5.1 Hz, $^3J_{CF}$ = 2.5 Hz); 33.1 (t, $^2J_{CF}$ = 24.3 Hz); 31.9; 29.5 (2C); 29.4; 29.3; 22.7; 21.1 (t, $^3J_{CF}$ = 3.9 Hz); 14.1. ^{19}F NMR ($CDCl_3$, 282 MHz), δ ppm: -102.23 (ddt, J_{FF} = 250.8 Hz, J_{FH} = 26.9 Hz, 11.8 Hz); -104.38 (dddd, J_{FF} = 250.8 Hz, J_{FH} = 27.3 Hz, 11.5 Hz, 6.6 Hz). HRMS (ESI) calcd for $C_{20}H_{29}F_2N_2Br^{79}BrNa$: $[M+Na]^+$: m/z 437.13799. Found: m/z 437.1382 (0 ppm).

4. General procedure for the synthesis of pyrrolines (6)

To a solution of *gem*-difluoroenone **3** (1 equiv) and Schiff base **5** (1 equiv) in CH_2Cl_2 (1.5 ml per mmol) cooled to 5 °C was added DBU (0.5 equiv). After stirring at 25 °C for the appropriate time (monitored by ^{19}F NMR), HCl (12 N, 1.5 equiv) was added and the mixture was stirred at 25 °C for the time *t* (monitored by ^{19}F NMR). Then water was added and the mixture was extracted with CH_2Cl_2. The organic layers were combined, dried over $MgSO_4$, filtered and concentrated in vacuo. The crude residue was then purified by flash chromatography over silica gel.

4.1. Synthesis of 3-(1,1-difluorodecyl)-5-phenyl-3,4-dihydro-2*H*-pyrrole-2-carboxylic acid ethyl ester ((±)-6a)

The reaction was performed from **3a** (400 mg, 1.29 mmol) according to the general procedure. After purification by chromatography on silica gel, pyrroline **6a** was obtained as beige crystals (378 mg, 74% yield). Mp: 66-68 °C. $\mathbf{R}_f$ = 0.31 (Et_2O/pentane 2/8). ^{1}H NMR (C_6D_6, 300 MHz), δ ppm: 7.85-7.75 (dd, 2H, *J* = 7.7 Hz, *J* = 1.4 Hz); 7.11-7.03 (m, 3H); 5.23 (dt, 1H, *J* = 6.5 Hz, $^4J_{HF}$ = 1.7 Hz); 4.41-3.94 (m, 2H); 3.51-3.29 (m, 1H); 2.92 (ddd, 1H, *J* = 17.4 Hz, *J* = 7.2 Hz, $^4J_{HF}$ = 1.7 Hz); 2.82 (ddd, 1H, *J* = 17.4 Hz, *J* = 10.0 Hz, $^4J_{HF}$ = 2.3 Hz); 1.73-1.62 (m, 2H); 1.56-1.43 (m, 2H); 1.33-1.12 (m, 12H); 0.96 (t, 3H, *J* = 7.1 Hz), 0.91 (t, 3H, *J* = 6.6 Hz). ^{13}C NMR ($CDCl_3$, 75 MHz), δ ppm: 174.1; 171.8; 133.2; 131.2; 128.4 (2C); 128.0 (2C); 124,4 (t, $^1J_{CF}$ = 243,1 Hz); 75.5 (dd, $^3J_{CF}$ = 4.0 Hz, $^3J_{CF}$ = 2.7 Hz); 61.6; 46.1 (t, $^2J_{CF}$ = 25.1 Hz); 36.7 (dd, $^3J_{CF}$ = 5.0 Hz, $^3J_{CF}$ = 2.7 Hz); 35.3 (t, $^2J_{CF}$ = 25.1 Hz); 31.8; 29.4; 29.32; 29.27; 29.2; 22.6; 21.8 (t, $^3J_{CF}$ = 4.3 Hz); 14.11; 14.05. ^{19}F NMR (C_6D_6, 282 MHz), δ ppm: -103.05 (dq, 1F, J_{FF} = 244.1 Hz, J_{FH} = 16.5 Hz); -104,8 (dq, 1F, J_{FF} = 244.1 Hz, J_{FH} = 16.2 Hz). HRMS (ESI) calcd for $C_{23}H_{33}F_2NO_2{}^{79}BrNa$: $[M+Na]^+$: *m/z* 416.23771. Found*: m/z* 416.2370 (2 ppm).

4.2. Synthesis of 3-(1,1-Difluorodecyl)-5-(pyridin-3-yl)-3,4-dihydro-2*H*-pyrrole-2-carboxylic acid ethyl ester ((±)-6b)

The reaction was performed from **3b** (205 mg, 0.66 mmol) according to the general procedure. After purification by chromatography on silica gel, pyrroline **6b** was obtained as

white crystals (191 mg, 73% yield). Mp: 43-45 °C. **R**$_f$ = 0.32 (Et_2O/pentane 7/3). ^{1}H NMR (C_6D_6, 300 MHz), δ ppm: 8.91 (d, 1H, *J* = 1.2 Hz); 8.46 (dd, 1H, *J* = 4.6 Hz, *J* = 1.2 Hz); 7.94 (dt, 1H, *J* = 8,0 Hz, *J* = 1.8 Hz); 6.61 (ddd, 1H, *J* = 8,0 Hz, *J* = 4.6 Hz, *J* = 0.6 Hz); 5.19 (dt, 1H, *J* = 6.5 Hz, $^4J_{HF}$ = 1.7 Hz); 4.10-3.94 (m, 2H); 3.45-3.21 (m, 1H); 2.74 (ddd, 1H, *J* = 17.5 Hz, *J* = 7.2 Hz, $^4J_{HF}$ = 1.7 Hz); 2.63 (ddd, 1H , *J* = 17.5 Hz, *J* = 10.0 Hz, $^4J_{HF}$ = 2.4 Hz); 1.75-1.59 (m, 2H); 1.54-1.45 (m, 2H); 1.28-1.13 (m, 12H); 0.96 (t, 3H, *J* = 7.1 Hz), 0.92 (t, 3H, *J* = 7.0 Hz). ^{13}C NMR ($CDCl_3$, 75 MHz), δ ppm: 171.9; 171.5; 152.0; 149.3; 135.1; 129.0; 127.5; 124,3 (t, $^1J_{CF}$ = 243,3 Hz); 123.5; 75.6 (dd, $^3J_{CF}$ = 4.2 Hz, $^3J_{CF}$ = 2.8 Hz); 61.8; 46.1 (t, $^2J_{CF}$ = 25.3 Hz); 36.6 (dd, $^3J_{CF}$ = 5.2 Hz, $^3J_{CF}$ = 2.7 Hz); 35.7 (t, $^2J_{CF}$ = 24.8 Hz); 31.8; 29.4; 29.34; 29.30; 29.2; 22.6; 21.8 (t, $^3J_{CF}$ = 3.9 Hz); 14.1; 14.0. ^{19}F NMR ($CDCl_3$, 282 MHz), δ ppm: -103.55 (dq, 1F, J_{FF} = 244.4 Hz, J_{FH} = 17.8 Hz); -105,86 (dq, 1F, J_{FF} = 244.5 Hz, J_{FH} = 15.5 Hz). HRMS (ESI) calcd for $C_{22}H_{32}F_2N_2O_2{}^{79}BrNa$: $[M +Na]^+$: *m/z* 417.23295. Found*: m/z* 417.2328 (0 ppm).

4.3. Synthesis of 3-(1,1-difluorodecyl)-5-(furan-2-yl)-3,4-dihydro-2*H*-pyrrole-2-carboxylic acid ethyl ester ((±)-6c)

The reaction was performed from **3c** (236 mg, 0.79 mmol) according to the general procedure. After purification by chromatography on silica gel, pyrroline **6c** was obtained as beige crystals (227 mg, 75% yield). Mp: 64-66 °C. **R**$_f$ = 0.28 (Et_2O/pentane 3/7). ^{1}H NMR (C_6D_6, 300 MHz), δ ppm: 6.96 (d, 1H, *J* = 1.5 Hz); 6.74 (d, 1H, *J* = 3.5 Hz); 5.94 (dd, 1H, *J* = 3.5 Hz, *J* = 1.5 Hz); 5.21 (dt, 1H, *J* = 6.3 Hz, $^4J_{HF}$ = 1.9 Hz); 4.07-3.91 (m, 2H); 3.43-3.21 (m, 1H); 2.95 (ddd, 1H, *J* = 17.7 Hz, *J* = 7.4 Hz, $^4J_{HF}$ = 1.8 Hz); 2.86 (ddd, 1H , *J* = 17.7 Hz, *J* = 9.9 Hz, $^4J_{HF}$ = 2.1 Hz); 1.74-1.56 (m, 2H); 1.52-1.38 (m, 2H); 1.34-1.08 (m, 12H); 0.94 (t, 3H, *J* = 7.2 Hz), 0.91 (t, 3H, *J* = 6.9 Hz). ^{13}C NMR (C_6D_6, 75 MHz), δ ppm: 171.7; 164.4; 150.0; 144.5; 124,8 (t, $^1J_{CF}$ = 243,0 Hz); 113.3; 111.8; 76.3 (dd, $^3J_{CF}$ = 3.7 Hz, $^3J_{CF}$ = 2.9 Hz); 61.3; 46.2 (t, $^2J_{CF}$ = 25.3 Hz); 36.9 (dd, $^3J_{CF}$ = 4.9 Hz, $^3J_{CF}$ = 3.0 Hz); 35.9 (t, $^2J_{CF}$ = 25.1 Hz); 32.1; 29.7; 29.6; 29.5; 29.4; 22.9; 22.1 (t, $^3J_{CF}$ = 4.2 Hz); 14.2; 13.9. ^{19}F NMR (C_6D_6, 282 MHz), δ ppm: -103.43 (dq, 1F, J_{FF} = 244.3 Hz, J_{FH} = 16.4 Hz); -104,57 (dq, 1F, J_{FF} = 244.3 Hz, J_{FH} = 15.9 Hz). HRMS (ESI) calcd for $C_{21}H_{31}F_2NO_3{}^{79}BrNa$: $[M +Na]^+$: *m/z* 406.21697. Found*: m/z* 406.2168 (0 ppm).

4.4. Synthesis of 3-(1,1-difluorodecyl)-5-(thiophen-2-yl)-3,4-dihydro-2*H*-pyrrole-2-carboxylic acid ethyl ester ((±)-6d)

The reaction was performed from **3d** (250 mg, 0.79 mmol) according to the general procedure. After purification by chromatography on silica gel, pyrroline **6d** was obtained as white crystals (235 mg, 74% yield). Mp: 67-69 °C. $\mathbf{R}_f$ = 0.24 (Et_2O/pentane 2/8). ^{1}H NMR (C_6D_6, 400 MHz), δ ppm: 6.87 (dd, 1H, J = 3.6 Hz, J = 0.8 Hz); 6.81 (dd, 1H, J = 4.9 Hz, J = 0.8 Hz); 6.59 (dd, 1H, J = 4.9 Hz, J = 3.6 Hz); 5.14 (dt, 1H, J = 6.4 Hz, $^4J_{HF}$ = 1.4 Hz); 4.08-3.92 (m, 2H); 3.49-3.29 (m, 1H); 2.91 (ddd, 1H, J = 17.2 Hz, J = 7.1 Hz, $^4J_{HF}$ = 1.5 Hz); 2.80 (ddd, 1H , J = 17.2 Hz, J = 10.1 Hz, $^4J_{HF}$ = 2.2 Hz); 1.73-1.61 (m, 2H); 1.55-1.42 (m, 2H); 1.32-1.09 (m, 12H); 0.93 (t, 3H, J = 7.2 Hz), 0.88 (t, 3H, J = 6.8 Hz). ^{13}C NMR (C_6D_6, 75 MHz), δ ppm: 171.8; 168.2; 150.0; 138.7; 130.4; 130.3; 127.3; 124.9 (t, $^1J_{CF}$ = 243,0 Hz); 76.0 (dd, $^3J_{CF}$ = 4.2 Hz, $^3J_{CF}$ = 1.2 Hz); 61.5; 47.0 (t, $^2J_{CF}$ = 25.3 Hz); 37.4 (dd, $^3J_{CF}$ = 4.9 Hz, $^3J_{CF}$ = 1.9 Hz); 36.0 (t, $^2J_{CF}$ = 25.1 Hz); 32.2; 29.8; 29.71; 29.66; 29.59; 23.1; 22.2 (t, $^3J_{CF}$ = 4.2 Hz); 14.3; 14.0. ^{19}F NMR (C_6D_6, 282 MHz), δ ppm: -102.73 (dq, 1F, J_{FF} = 244.3 Hz, J_{FH} = 16.8 Hz); -104,8 (dq, 1F, J_{FF} = 244.3 Hz, J_{FH} = 16.1 Hz). HRMS (ESI) calcd for $C_{21}H_{31}F_2NO_2S^{79}BrNa$: $[M +Na]^+$: m/z 422.19413. Found: m/z 422.1927 (3 ppm).

4.5. Synthesis of 5-(4-bromophenyl)-3-(1,1-difluorodecyl)-3,4-dihydro-2*H*-pyrrole-2-carboxylic acid ethyl ester ((±)-6e)

The reaction was performed from **3e** (380 mg, 0.98 mmol) according to the general procedure. After purification by chromatography on silica gel, pyrroline **6e** was obtained as white crystals (363 mg, 78% yield). Mp: 45-47 °C. $\mathbf{R}_f$ = 0.29 (Et_2O/pentane 1/9). ^{1}H NMR (C_6D_6, 400 MHz), δ ppm: 7.40 (d, 1H, J = 8.6 Hz, J = 1.9 Hz); 7.17 (dd, 1H, J = 8.6 Hz, J = 1.6 Hz); 5.16 (dt, 1H, J = 6.5 Hz, $^4J_{HF}$ = 1.7 Hz); 4.09-3.95 (m, 2H); 3.48-3.21 (m, 1H); 2.78 (ddd, 1H, J = 17.4 Hz, J = 7.3 Hz, $^4J_{HF}$ = 1.5 Hz); 2.65 (ddd, 1H , J = 17.4 Hz, J = 10.1 Hz, $^4J_{HF}$ = 2.3 Hz); 1.76-1.64 (m, 2H); 1.57-1.45 (m, 2H); 1.34-1.14 (m, 12H); 0.91 (t, 3H, J = 7.1 Hz), 0.86 (t, 3H, J = 6.5 Hz). ^{13}C NMR ($CDCl_3$, 75 MHz), δ ppm: 173.2; 171.7; 132.1; 131.8 (2C); 129.6 (2C); 125.9; 124.4 (t, $^1J_{CF}$ = 243.2 Hz); 75.6 (dd, $^3J_{CF}$ = 4.1 Hz, $^3J_{CF}$ = 2.6 Hz); 61.8; 46.2 (t, $^2J_{CF}$ = 25.2 Hz); 36.7 (dd, $^3J_{CF}$ = 5.1 Hz, $^3J_{CF}$ = 2.6 Hz); 35.7 (t, $^2J_{CF}$ = 24.9 Hz); 31.9; 29.4; 29.39; 29.34; 29.28; 22.7; 21.9 (t, $^3J_{CF}$ = 4.1 Hz); 14.2; 14.1. ^{19}F NMR ($CDCl_3$, 282 MHz), δ ppm: -103.48 (dq, 1F, J_{FF} = 244.5 Hz , J_{FH} = 13.6 Hz); -105.73 (dq, 1F, J_{FF} = 244.5 Hz, J_{FH} = 15.6 Hz). HRMS (ESI) calcd for $C_{23}H_{32}F_2NO_2Br^{79}BrNa$: $[M +Na]^+$: m/z 494.14822. Found: *m/z* 494.1483 (0 ppm).

5. General procedure for the Suzuki–Miyaura reaction

A solution of brominated derivates **4e** or **6e** (1 equiv), phenylboronic acid (2 equiv), palladium dichlorobistriphenylphosphine (5 mol %) and potassium carbonate (2 equiv) in a 5/1 mixture of dioxane and water (1 ml per 0.1 mmol) was stirred at 90 °C for 24 h. After addition of $MgSO_4$ and filtration, the residues were washed with ether and the solution concentated in vacuo. The crude residue was then purified by flash chromatography on silica gel.

5.1. Synthesis of 3-biphenyl-4-yl-5(1,1-difluorodecyl)-1-methyl-4,5-dihydro-1*H*-pyrazole ((±)-7e)

The reaction was performed from **4e** (78 mg, 0.19 mmol) according to the general procedure. After purification by chromatography on silica gel, the product **7e** was isolated as a sticky syrup (64 mg, 82% yield). $\mathbf{R}_f$ = 0.31 (Et_2O/pentane 5/95). ^{1}H NMR ($CDCl_3$, 300 MHz), δ ppm: 7.72-7.67(m, 2H); 7.64-7.57(m, 4H); 7.49-7.34 (m, 3H); 3.54 (dddd, 1H, J = 12.0 Hz, J = 11.7 Hz, $^3J_{HF}$ = 11.7 Hz, $^3J_{HF}$ = 6.6 Hz); 3.29 (dd, 1H, J = 16.9 Hz, J = 11.7 Hz); 3.02 (dd, 1H, J = 16.9 Hz, J = 12.0 Hz); 3.00 (s, 3H,) ; 2.09-1.74 (m, 2H); 1.63-1.51 (m, 2H); 1.43-1.18 (m, 12H); 0.87 (t, 3H, J = 6.7 Hz). ^{13}C NMR ($CDCl_3$, 75 MHz), δ ppm: 149.3; 141.7; 140.3; 130.8; 128.82 (2C); 127.6; 127.2 (2C); 127.0 (2C); 126.4 (2C); 124.5 (t, $^1J_{CF}$ = 242.8 Hz); 71.1 (dd, $^2J_{CF}$ = 28.4 Hz, $^2J_{CF}$ = 27.4 Hz); 44.0; 35.8 (dd, $^3J_{CF}$ = 5.1 Hz, $^3J_{CF}$ = 2.1 Hz); 33.0 (t, $^2J_{CF}$ = 24.4 Hz); 31.8; 29.43 (2C); 29.40; 29.3; 22.7; 21.1 (t, $^3J_{CF}$ = 4.1 Hz); 14.1. ^{19}F NMR ($CDCl_3$, 282 MHz), δ ppm: -102.13 (ddt, J_{FF} = 250.5 Hz, J_{FH} = 26.8 Hz, 11.7 Hz); -104.33 (dddd, J_{FF} = 250.5 Hz, J_{FH} = 27.2 Hz, 11.5 Hz, 6.6 Hz). HRMS (ESI) calcd for $C_{26}H_{34}F_2N_2{}^{79}BrNa$: $[M+Na]^+$: 435.25878. m/z Found: 435.2584 (1 ppm).

5.2. Synthesis of 5-biphenyl-4-yl-3-(1,1-difluorodecyl)-3,4-dihydro-2*H*-pyrrole-2-carboxylic acid ethyl ester (±)-10e

The reaction was performed from **6e** (67 mg, 0.14 mmol) according to the general procedure. After purification by chromatography on silica gel, the product **10e** was isolated as a sticky syrup (52 mg, 78% yield). $\mathbf{R}_f$ = 0.35 (Et_2O/pentane 2/8). ^{1}H NMR (C_6D_6, 300 MHz), δ ppm: 7.88-7.79 (m, 2H); 7.43-7.34 (m, 4H); 7.24-7.16 (m, 3H); 5.28 (dt, 1H, J = 6.4 Hz, $^4J_{HF}$ = 1.5 Hz); 4.06-3.92 (m, 2H); 3.55-3.32 (m, 1H); 3.01 (ddd, 1H, J = 17.4 Hz, J = 7.3 Hz, $^4J_{HF}$ = 1.6

Hz); 2.88 (ddd, 1H , J = 17.4 Hz, J = 10.1 Hz, $^4J_{HF}$ = 2.3 Hz); 1.84-1.63 (m, 2H); 1.61-1.48 (m, 2H); 1.36-1.12 (m, 12H); 0.99 (t, 3H, J = 7.1 Hz), 0.92 (t, 3H, J = 7.0 Hz). ^{13}C NMR (C_6D_6, 75 MHz), δ ppm: 173.3; 172.1; 144.0; 140.7; 132.9; 129.2 (2C); 129.1(2C); 127.3 (2C); 127,2 (2C); 125.1 (t, $^1J_{CF}$ = 242,9 Hz); 76.4 (dd, $^3J_{CF}$ = 3.7 Hz, $^3J_{CF}$ = 2.7 Hz); 61.5; 46.8 (t, $^2J_{CF}$ = 24.8 Hz); 36.9 (dd, ^3J = 4.6 Hz, $^3J_{CF}$ = 2.7 Hz); 35.1 (t, $^2J_{CF}$ = 25.1 Hz); 32.2; 29.84; 29.75; 29.68; 29.65; 23.1; 22.3 (t, $^3J_{CF}$ = 4.3 Hz); 14.3; 14.1. ^{19}F NMR (C_6D_6, 282 MHz), δ ppm: -102.9 (dq, 1F, J_{FF} = 243.8 Hz, J_{FH} = 16.7 Hz); -104.8 (dq, 1F, J_{FF} = 243.8 Hz, J_{FH} = 16.2 Hz). HRMS (ESI) calcd for $C_{29}H_{37}F_2NO_2{}^{79}BrNa$: $[M +Na]^+$: m/z 492.26901. Found: *m/z* 492.2682 (2 ppm).

6. General procedure for the Heck reaction

Et_3N (1 ml per 0.1 mmol) was first degassed by bubbling argon for 10 min. Then were added successively: bromo-compound **4e** or **6e** (1 equiv), palladium acetate (10 mol %), tri-*o*-tolylphosphine (0.2 equiv) and methyl acrylate (3.2 equiv). The reaction mixture was stirred under argon at 100 °C for 12 h. After addition of ether, the reaction mixture was washed with water and the aqueous phase was extracted with ether. The combined organic phases were dried ($MgSO_4$), filtered and concentrated in vacuo. The crude residue was then purified by flash chromatography on silica gel.

6.1. Synthesis of 3-{4-[5-(1,1-difluorodecyl)-1-methyl-4,5-dihydro-1*H*-pyrazol-3-yl]-phenyl}-acrylic acid methyl ester ((±)-8e)

The reaction was performed from **4e** (111 mg, 0.27 mmol) according to the general procedure. After purification by chromatography on silica gel, the product **8e** was isolated as a sticky syrup (81 mg, 72% yield). $\mathbf{R}_f$ = 0.30 (Et_2O/pentane 1/9). ^{1}H NMR ($CDCl_3$, 300 MHz), δ ppm: 7.67 (d, 1H, J = 16.1 Hz); 7.61 (d, 2H, J = 8.4 Hz); 7.50 (d, 2H, J = 8.4 Hz); 6.45 (d, 1H, J = 16.1 Hz); 3.80 (s, 3H); 3.58 (dddd, 1H, J = 12.0 Hz, $^3J_{HF}$ = 11.9 Hz, J = 11.7 Hz, $^3J_{HF}$ = 6.5 Hz); 3.33 (dd, 1H, J = 16.8 Hz, J = 11.7 Hz); 3.02 (dd, 1H, J = 16.8 Hz, J = 12.0 Hz); 3.01 (s, 3H,) ; 2.08-1.76 (m, 2H); 1.63-1.50 (m, 2H); 1.39-1.17 (m, 12H); 0.88 (t, 3H, J = 6.7 Hz). ^{13}C NMR ($CDCl_3$, 75 MHz), δ ppm: 167.3; 148.1; 144.0; 134.6; 133.9; 128.2 (2C); 126.2 (2C); 124.5 (t, $^1J_{CF}$ = 242.6 Hz); 118.0; 71.2 (t, $^2J_{CF}$ = 28.8 Hz); 51.7; 43.7; 35.5 (dd, $^3J_{CF}$ = 5.0 Hz, $^3J_{CF}$ = 2.3 Hz); 33.0 (t, $^2J_{CF}$ = 24.3 Hz); 31.8; 29.41 (2C); 29.37; 29.2; 22.6; 21.1 (t, $^3J_{CF}$ = 4.0 Hz); 14.1. ^{19}F NMR ($CDCl_3$, 282 MHz), δ ppm: -102.22 (ddt, J_{FF} = 250.5 Hz, J_{FH} =

27.0 Hz, 11.9 Hz); -104.34 (dddd, J_{FF} = 250.9 Hz, J_{FH} = 26.7 Hz, 12.2 Hz, 6.5 Hz). HRMS (ESI) calcd for $C_{24}H_{34}F_2N_2O_2Na$: $[M +Na]^+$: m/z 443.24860. Found: m/z 443.2787 (0 ppm).

6.2. Synthesis of 3-(1,1-difluorodecyl)-5-[4-(2-methoxycarbonyl-vinyl)-phenyl]-3,4-dihydro-2*H*-pyrrole-2-carboxylic acid ethyl ester ((±)-11e)

The reaction was performed from **6e** (124 mg, 0.26 mmol) according to the general procedure. After purification by chromatography on silica gel, the product **11e** was isolated as a sticky syrup (80 mg, 64% yield). $\mathbf{R_f}$ = 0.14 (Et_2O/pentane 2/8). ^{1}H NMR (C_6D_6, 300 MHz), δ ppm: 7.70 (d, 1H, J = 16.1 Hz); 7.62 (d, 2H, J = 8.3 Hz); 7.00 (d, 2H, J = 8.3 Hz); 6.37 (d, 1H, J = 16.1 Hz); 5.23 (dt, 1H, J = 6.4 Hz, $^4J_{HF}$ = 1.8 Hz); 4.12-3.94 (m, 2H); 3.48 (s, H); 3.47-3.29 (m, 1H); 2.83 (ddd, 1H, J = 17.4 Hz, J = 7.3 Hz, $^4J_{HF}$ = 1.6 Hz); 2.65 (ddd, 1H , J = 17.4 Hz, J = 10.1 Hz, $^4J_{HF}$ = 2.3 Hz); 1.80-1.62 (m, 2H); 1.60-1.45 (m, 2H); 1.31-1.13 (m, 12H); 0.98 (t, 3H, J = 7.1 Hz), 0.92 (t, 3H, J = 6.5 Hz). ^{13}C NMR (C_6D_6, 75 MHz), δ ppm: 173.0; 171.9; 166.7; 143.8; 137.1; 135.1; 128.8 (2C); 128.2; 124.4 (t, $^1J_{CF}$ = 242.7 Hz); 119.6; 75.4 (dd, $^3J_{CF}$ = 4.2 Hz, $^3J_{CF}$ = 2.7 Hz); 61.6; 51.3; 46.7 (t, $^2J_{CF}$ = 25.4 Hz); 36.8 (dd, $^3J_{CF}$ = 5.0 Hz, $^3J_{CF}$ = 2.7 Hz); 36.1 (t, $^2J_{CF}$ = 25.0 Hz); 32.2; 29.8; 29.73; 29.67; 29.6; 23.1; 22.3 (t, $^3J_{CF}$ = 4.0 Hz); 14.3; 14.1. ^{19}F NMR (C_6D_6, 282 MHz), δ ppm: -102.90 (dq, 1F, J_{FF} = 244.3 Hz, J_{FH} = 16.2 Hz); -105.0 (dq, 1F, J_{FF} = 244.3 Hz, J_{FH} = 16.8 Hz). HRMS (ESI) calcd for $C_{27}H_{37}F_2NO_4Na$: $[M +Na]^+$: m/z 500.25883. Found: *m/z* 500.2590 (0 ppm).

7. General procedure for the Sonogashira reaction

Dioxane (1 ml per 0.1 mmol) was first degassed by bubbling argon. Then were added successively: bromo-compound (**4e** or **6e**) (1 equiv), palladium dichlorobistriphenylphosphine (5 mol %), copper iodide (2.5 mol %), potassium carbonate (2 equiv) and ethynylbenzene (1 equiv). The reaction mixture was stirred under argon at 90 °C for 2 d. After addition of ether the reaction mixture was washed with water, the aqueous phase was extracted with ether. The combined organic phases were dried ($MgSO_4$), filtered and concentrated in vacuo. The crude residue was then purified by flash chromatography on silica gel.

7.1. Synthesis of 5-(1,1-difluorodecyl)-1-methyl-3-(4-phenylethynyl-phenyl)-4,5-dihydro-1*H*-pyrazole ((±)-9e)

The reaction was performed from **4e** (100 mg, 0.26 mmol) according to the general procedure. After purification by chromatography on silica gel, the product **9e** was isolated as

a sticky syrup (78 mg, 74% yield). $\mathbf{R}_f$ = 0.29 (Et_2O/pentane 5/95). ^{1}H NMR ($CDCl_3$, 300 MHz), δ ppm: 7.67-7.53 (m, 6H); 7.42-7.36 (m, 3H); 3.61 (dddd, 1H, J = 12.0 Hz, $^3J_{HF}$ = 11.8 Hz; J = 11.7 Hz, $^3J_{HF}$ = 6.4 Hz); 3.29 (dd, 1H, J = 16.8 Hz, J = 11.7 Hz); 3.00 (dd, 1H, J = 16.8 Hz, J = 12.0 Hz); 3.04 (s, 3H,) ; 2.15-1.81 (m, 2H); 1.63-1.58 (m, 2H); 1.41-1.24 (m, 12H); 0.91 (t, 3H, J = 6.5 Hz). ^{13}C NMR ($CDCl_3$, 75 MHz), δ ppm: 148.4; 131.9; 131.7 (2C); 131.6 (2C); 128.4 (2C); 125,9 (2C); 125.3; 124.6 (t, $^1J_{CF}$ = 242.7 Hz); 123.6; 123.1; 90.9; 89.3; 71.3 (dd, $^2J_{CF}$ = 28.6 Hz, J_{CF} = 27.4 Hz); 43.8; 35.5 (dd, $^3J_{CF}$ = 5.1 Hz, J_{CF} = 2.3 Hz); 33.0 (t, $^2J_{CF}$ = 24.5 Hz); 31.9; 29.43 (2C); 29.40; 29.3; 22.7; 21.1 (t, $^3J_{CF}$ = 4.1 Hz); 14.1. ^{19}F NMR ($CDCl_3$, 282 MHz), δ ppm: -102.23 (ddt, J_{FF} = 250.8Hz, J_{FH} = 26.9 Hz, 11.8 Hz); -104.38 (dddd, J_{FF} = 250.8 Hz, J_{FH} = 27.3 Hz, 11.5 Hz, 6.4 Hz). HRMS (ESI) calcd for $C_{28}H_{34}F_2N_2{}^{79}BrNa$: $[M+Na]^+$: m/z 459.2583. Found: m/z 459.25877 (0 ppm).

7.2. Synthesis of 3-(1,1-difluorodecyl)-5-(4-phenylethynyl-phenyl)-3,4-dihydro-2*H*-pyrrole-2-carboxylic acid ethyl ester ((±)-12e)

The reaction was performed from **6e** (100 mg, 0.21 mmol) according to the general procedure. After purification by chromatography on silica gel, the product **12e** was isolated as a sticky syrup (73 mg, 70% yield). $\mathbf{R}_f$ = 0.21 (Et_2O/pentane 1/9). ^{1}H NMR (C_6D_6, 400 MHz), δ ppm: 7.69-7.61 (m, 2H); 7.55-7.49 (m, 2H); 7.46-7.40 (2H); 7.1-6.8 (3H); 5.22 (dt, 1H, J = 6.5 Hz, $^4J_{HF}$ = 1.6 Hz); 4.13-3.97 (m, 2H); 3.49-3.32 (m, 1H); 2.85 (ddd, 1H, J = 17.4 Hz, J = 7.4 Hz, $^4J_{HF}$ = 1.6 Hz); 2.71 (ddd, 1H , J = 17.4 Hz, J = 10.2 Hz, $^4J_{HF}$ = 2.3Hz); 1.77-1.64 (m, 2H); 1.57-1.47 (m, 2H); 1.29-1.15 (m, 12H); 0.99 (t, 3H, J = 7.1 Hz), 0.92 (t, 3H, J = 6.9 Hz). ^{13}C NMR ($CDCl_3$, 75 MHz), δ ppm: 172.7; 171.6; 133.3; 131.7 (2C); 131.6 (2C); 128.4 (2C); 128.3 (2C); 126.1; 124.7 (t, $^1J_{CF}$ = 242,9 Hz); 92.0; 89.6; 76.1; 61.3; 46.2 (t, $^2J_{CF}$ = 25.1 Hz); 36.5 (dd, ^3J = 5.0 Hz, $^3J_{CF}$ = 2.0 Hz); 35.9 (t, $^2J_{CF}$ = 25.1 Hz); 32.0; 29.6; 29.5; 29.4; 22. 8; 21.0 (t, $^3J_{CF}$ = 3.9 Hz); 14.1; 13.8. ^{19}F NMR (C_6D_6, 282 MHz), δ ppm: -102.91 (dq, 1F, J_{FF} = 243.9 Hz, J_{FH} = 16.5 Hz); -104.75 (dq, 1F, J_{FF} = 243.9 Hz, J_{FH} = 16.1 Hz). HRMS (ESI) calcd for $C_{31}H_{37}F_2NO_2{}^{79}BrNa$: $[M+Na]^+$: m/z 516.26901 . Found: m/z 516.2689 (0 ppm).

8. Analysis of $^{19}F/^{1}H$ HOESY 2D-NMR spectrum of (±)-6a

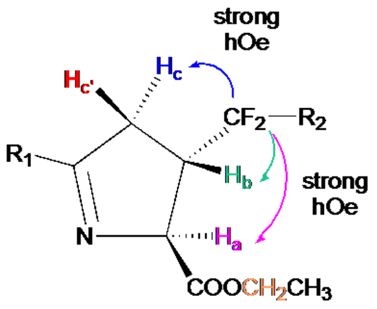

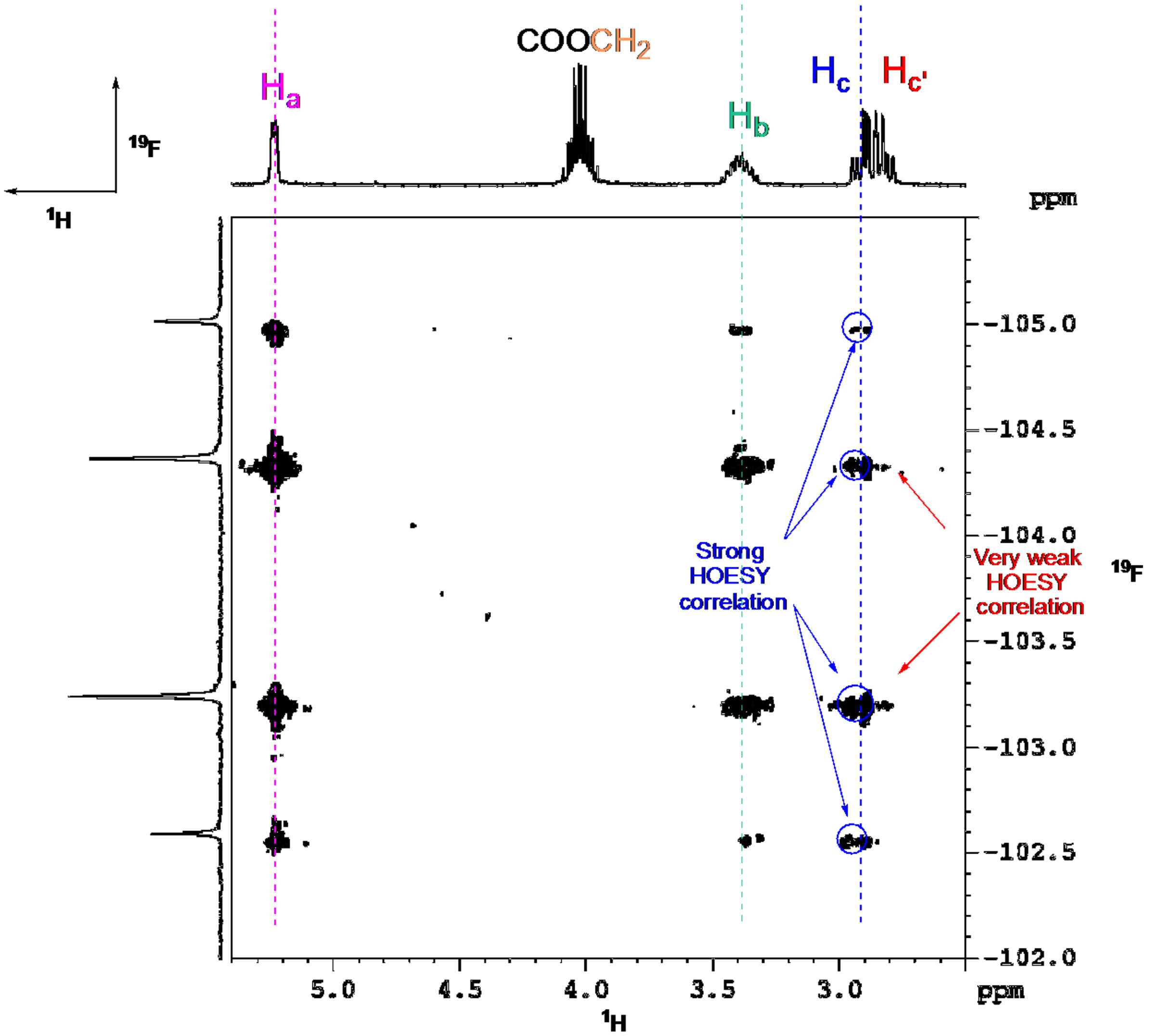

Figure S-1: Pulse gradient $^{19}F/^{1}H$ HOESY 2D-NMR spectrum of **(±)-6a** in C_6D_6 recorded at 300 K and 9.4 T (Bruker avance I NMR spectrometer equipped with a QXO probe, ($\nu(^{19}F)$ = 376,5 MHz/$\nu(^{1}H)$ = 400.1 MHz) and 300 K. The map was recorded using a 2D matrix of 4096(t_1) × 256(t_2) data points, and then zero-filled to 4096(t_1) × 1024(t_2) data points. The mixing time was set up at 800 ms. 16 scans were added per t_1 increment. Exponential filtering (LB = 5 Hz) was applied in both spectral dimensions. The F_1 and F_2 projections displayed correspond to ^{19}F-{^{1}H} and ^{1}H 1D spectra, respectively.

9. Library of ^{1}H, ^{13}C-{^{1}H}, ^{19}F-{^{1}H} and ^{19}F 1D-NMR spectra

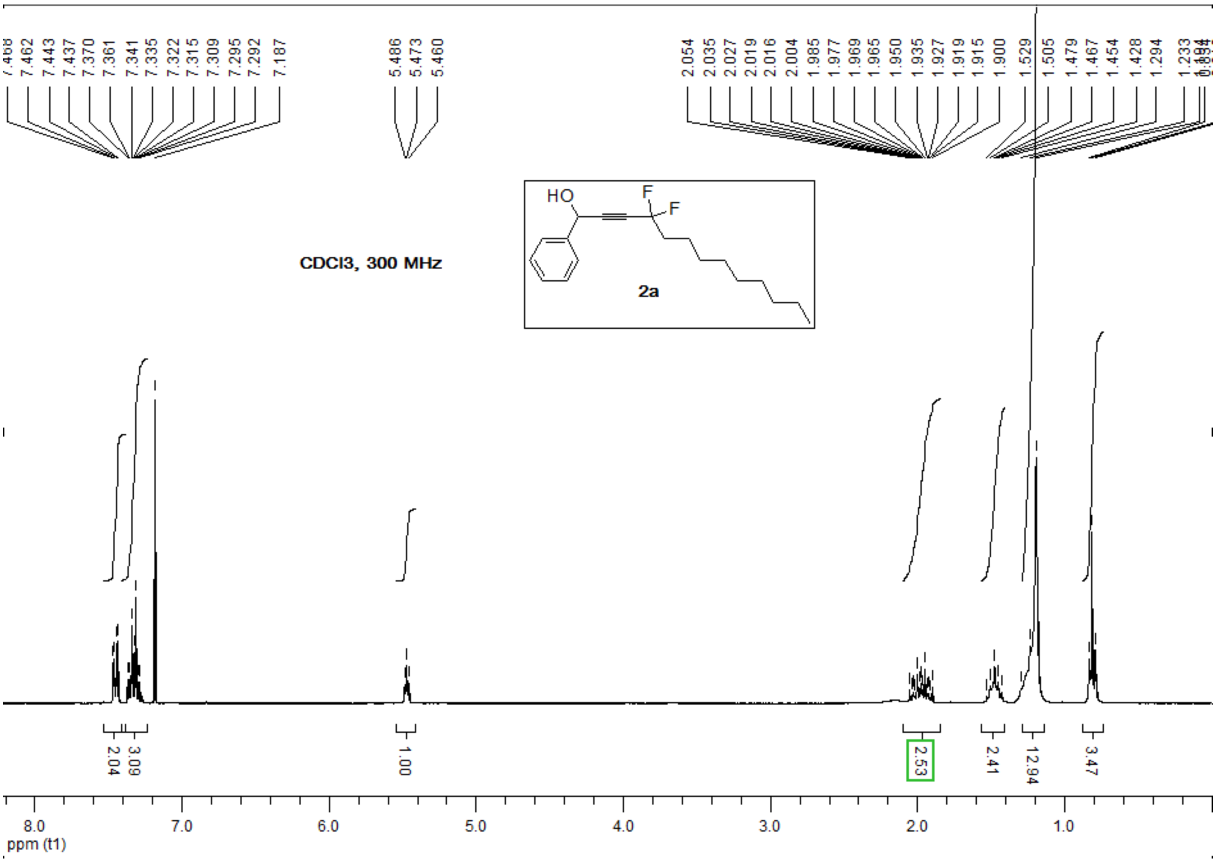

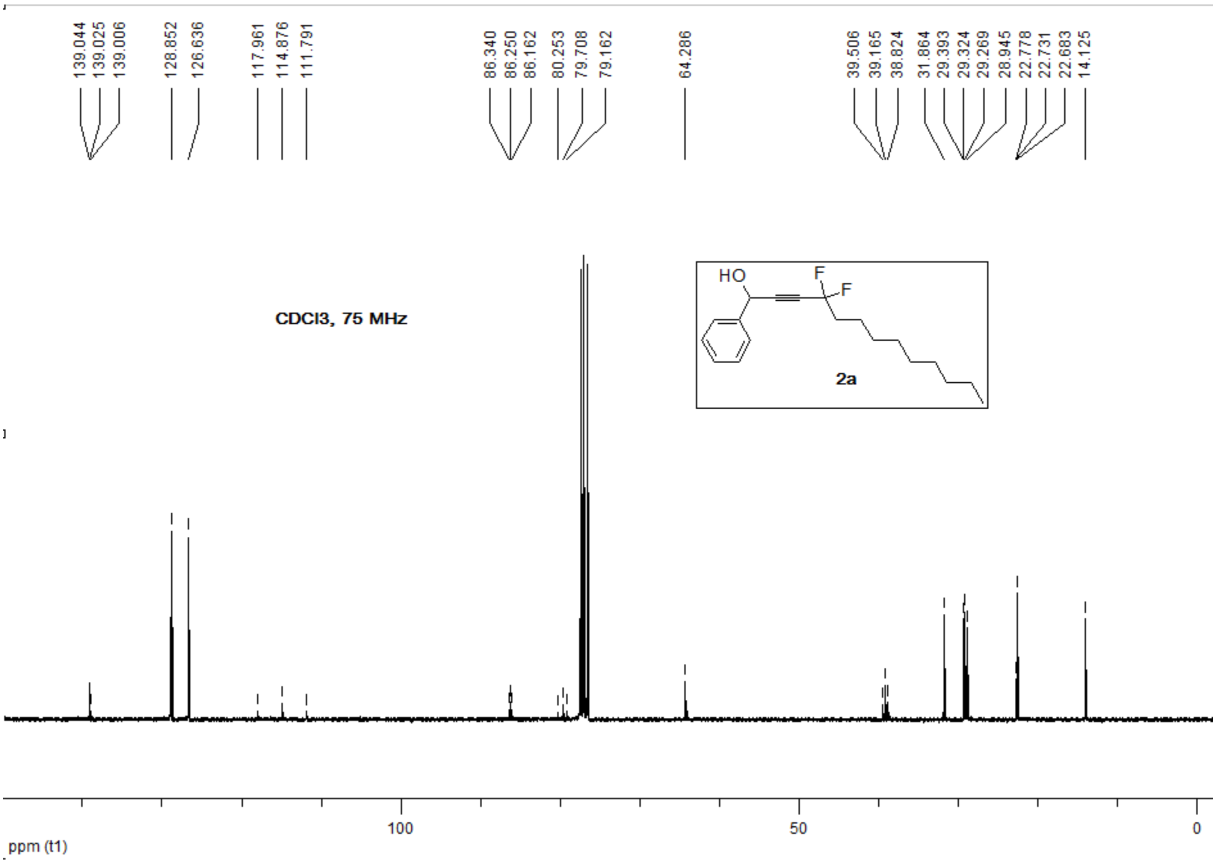

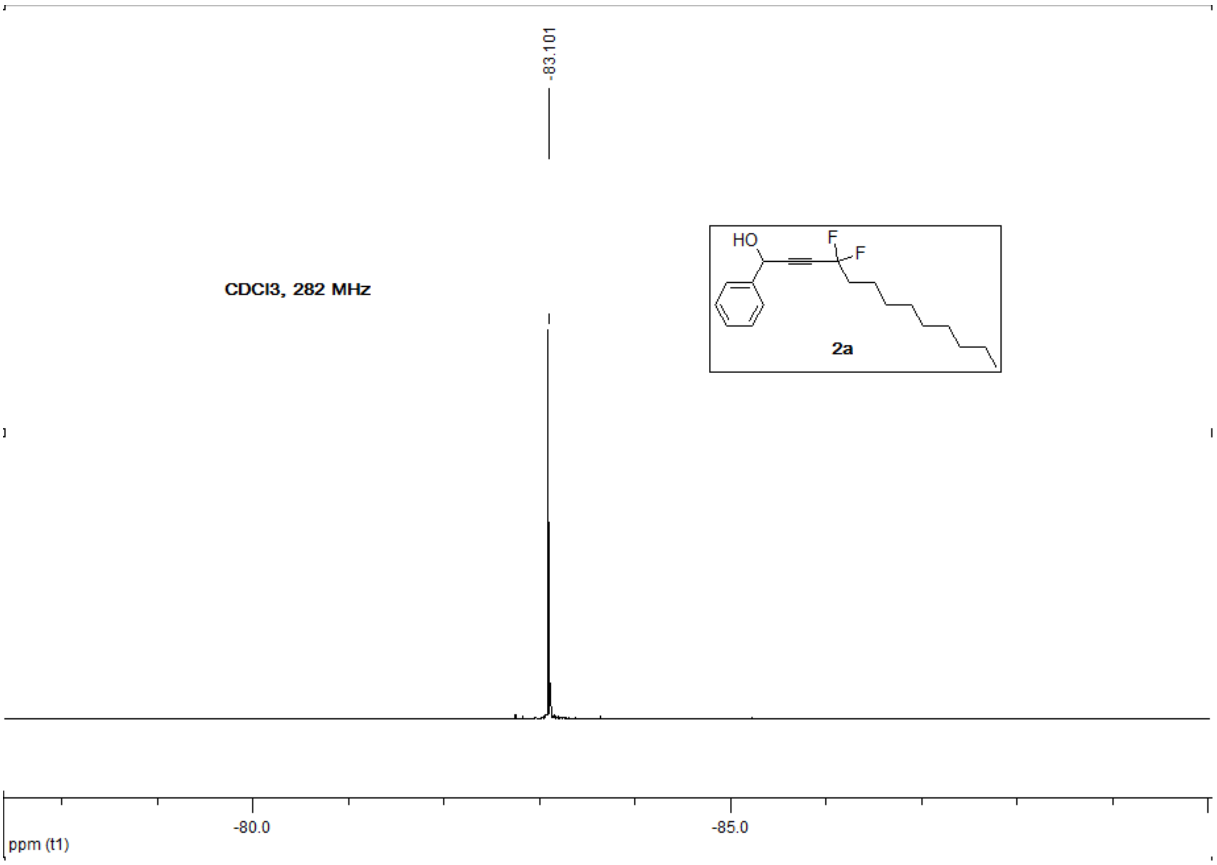
-83.101
CDCl3, 282 MHz
HO
F
F
2a
-80.0
-85.0
ppm (t1)

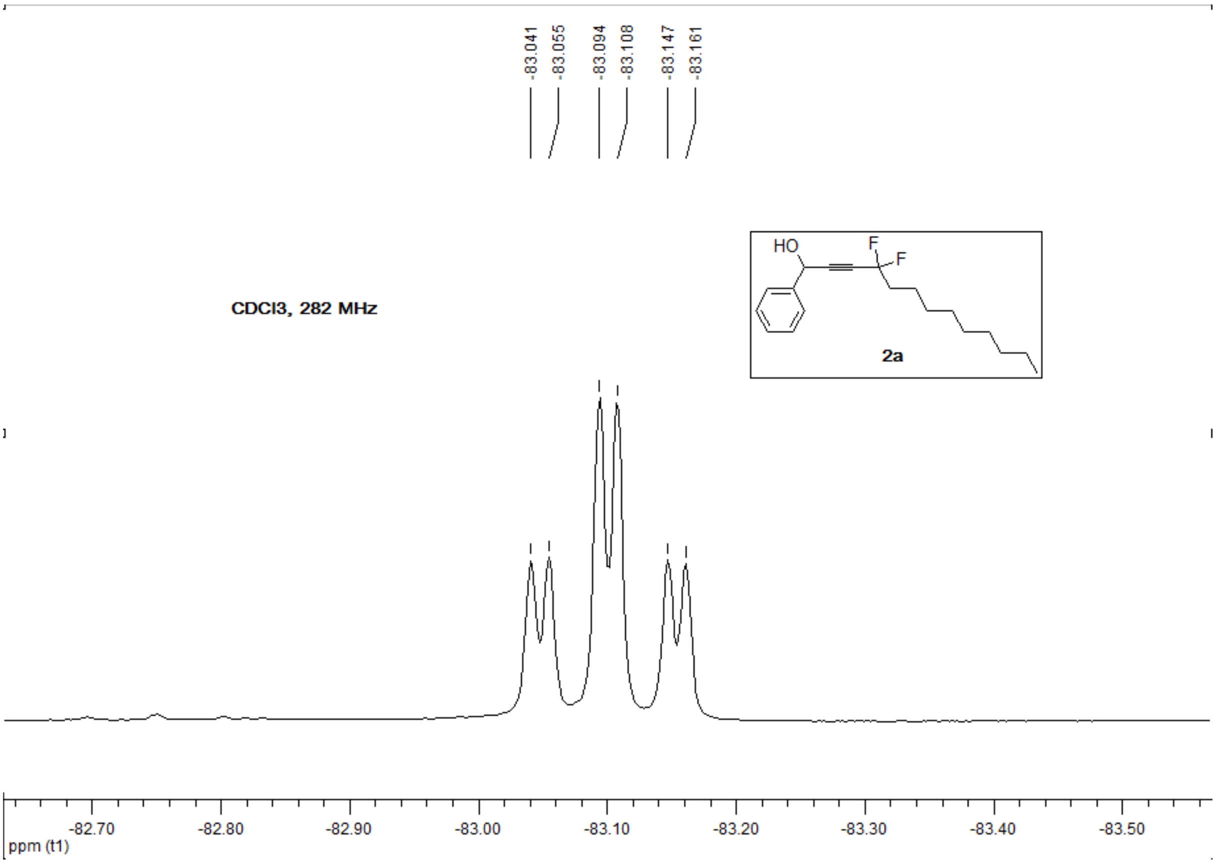
-83.041
-83.055
-83.094
-83.108
-83.147
-83.161
CDCl3, 282 MHz
HO
F
F
2a
-82.70
-82.80
-82.90
-83.00
-83.10
-83.20
-83.30
-83.40
-83.50
ppm (t1)

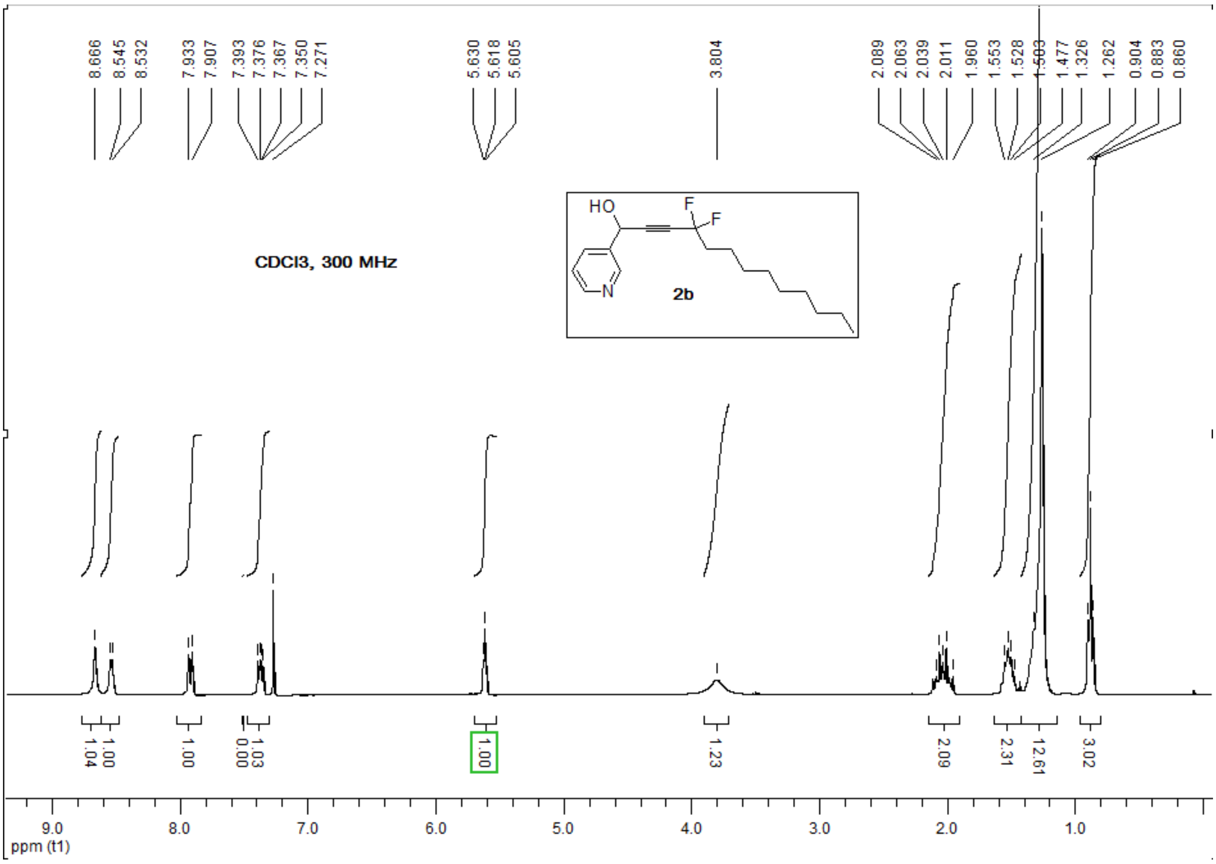
8.666
8.545
8.532
7.933
7.907
7.393
7.376
7.367
7.350
7.271
5.630
5.618
5.605
3.804
2.089
2.063
2.039
2.011
1.960
1.553
1.528
1.503
1.477
1.326
1.262
0.904
0.883
0.860
CDCl3, 300 MHz
HO
F
F
N
2b
1.04
1.00
1.00
0.00
1.03
1.00
1.23
2.09
2.31
12.61
3.02
9.0
8.0
7.0
6.0
5.0
4.0
3.0
2.0
1.0
ppm (t1)

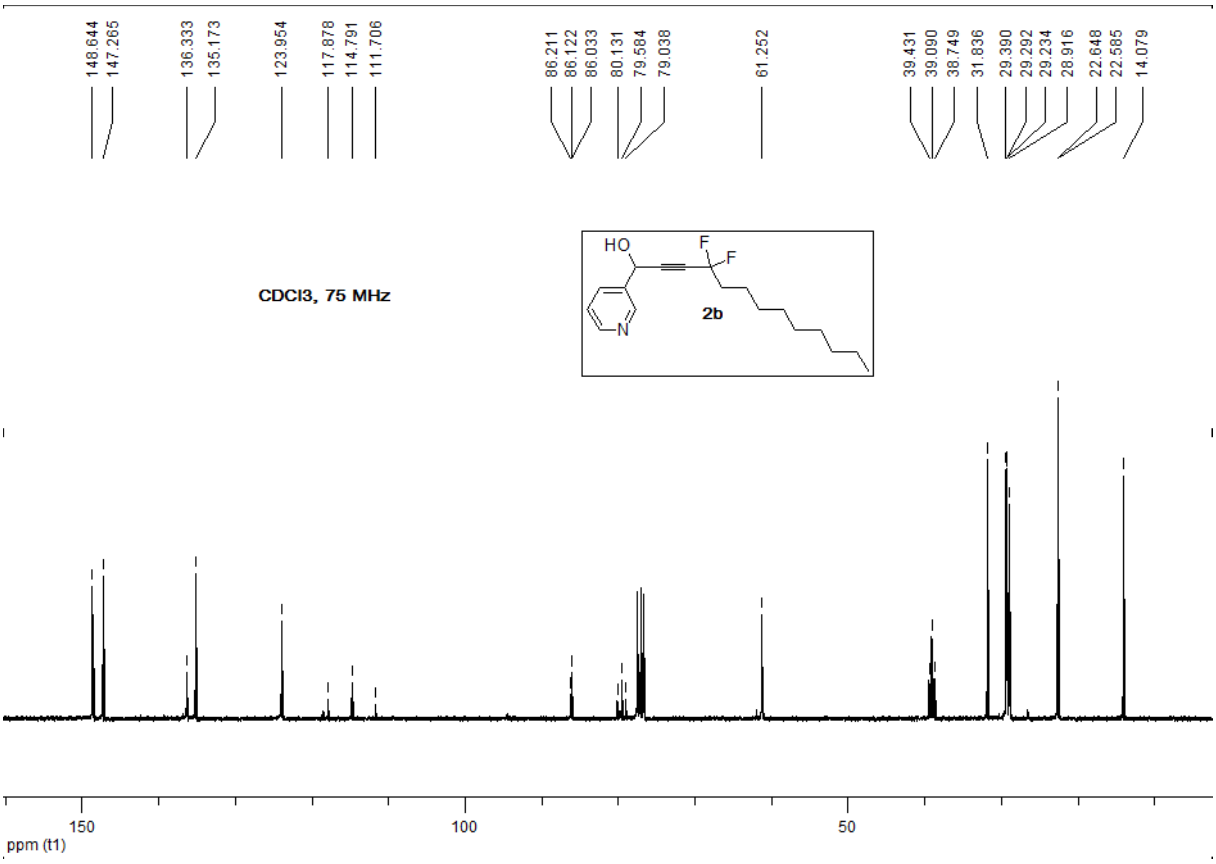
148.644
147.265
136.333
135.173
123.954
117.878
114.791
111.706
86.211
86.122
86.033
80.131
79.584
79.038
61.252
39.431
39.090
38.749
31.836
29.390
29.292
29.234
28.916
22.648
22.585
14.079
CDCl3, 75 MHz
HO
F
F
N
2b
150
100
50
ppm (t1)

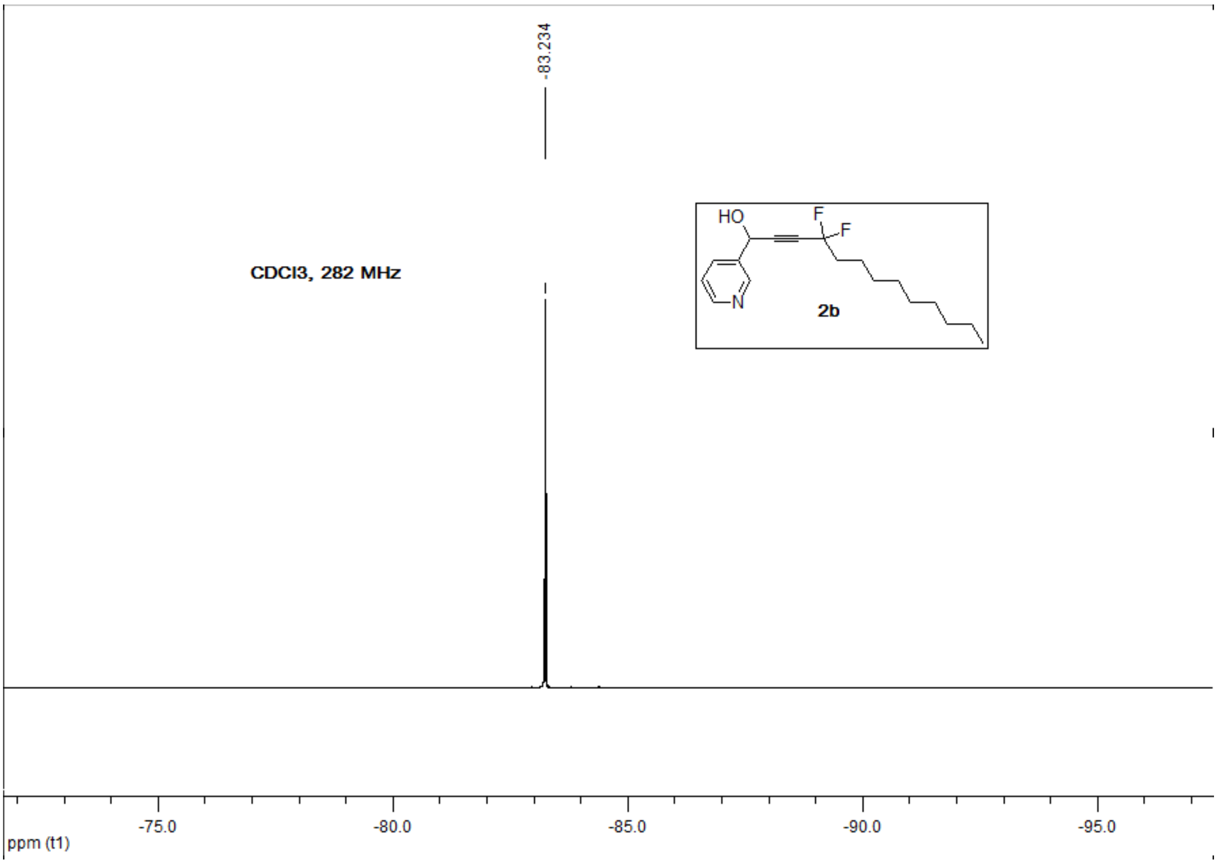
-83.234
CDCl3, 282 MHz
HO
F
F
N
2b
-75.0
-80.0
-85.0
-90.0
-95.0
ppm (t1)

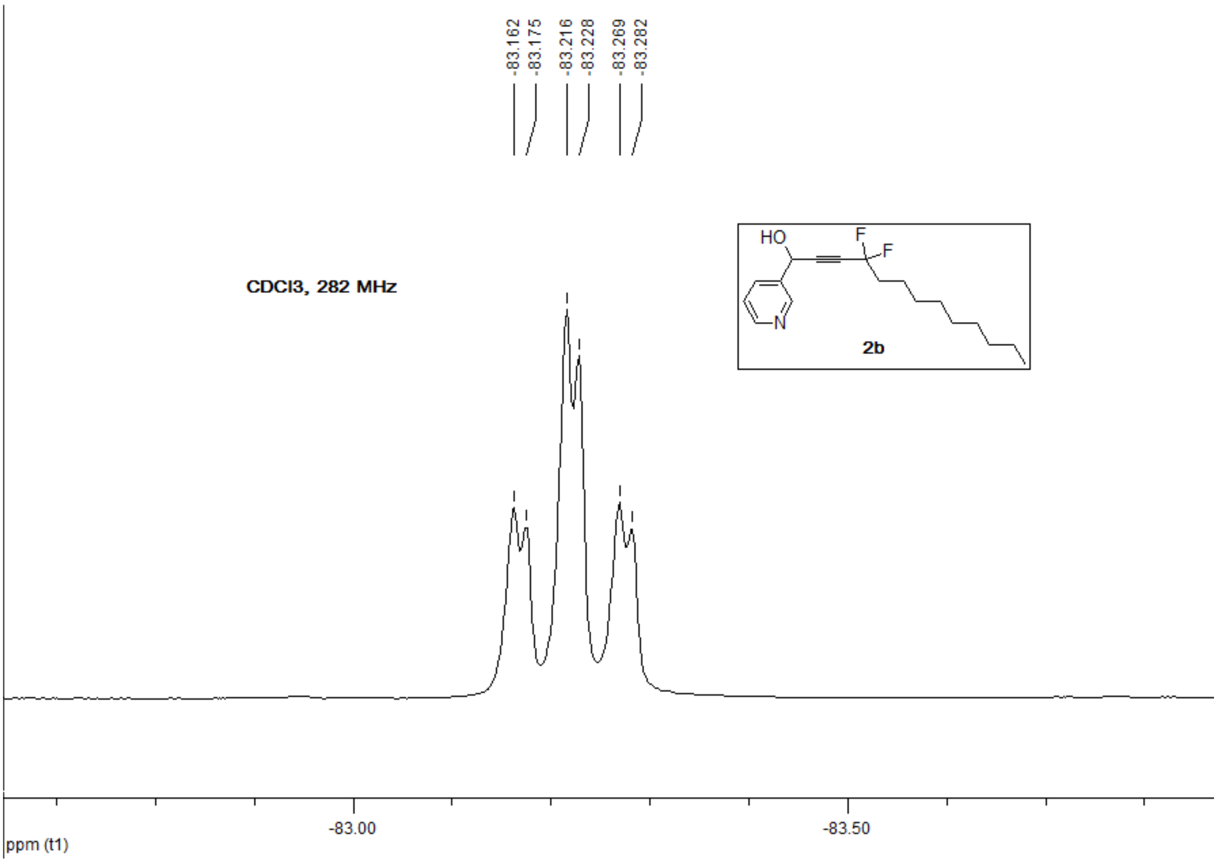
-83.162
-83.175
-83.216
-83.228
-83.269
-83.282
CDCl3, 282 MHz
HO
F
F
N
2b
-83.00
-83.50
ppm (t1)

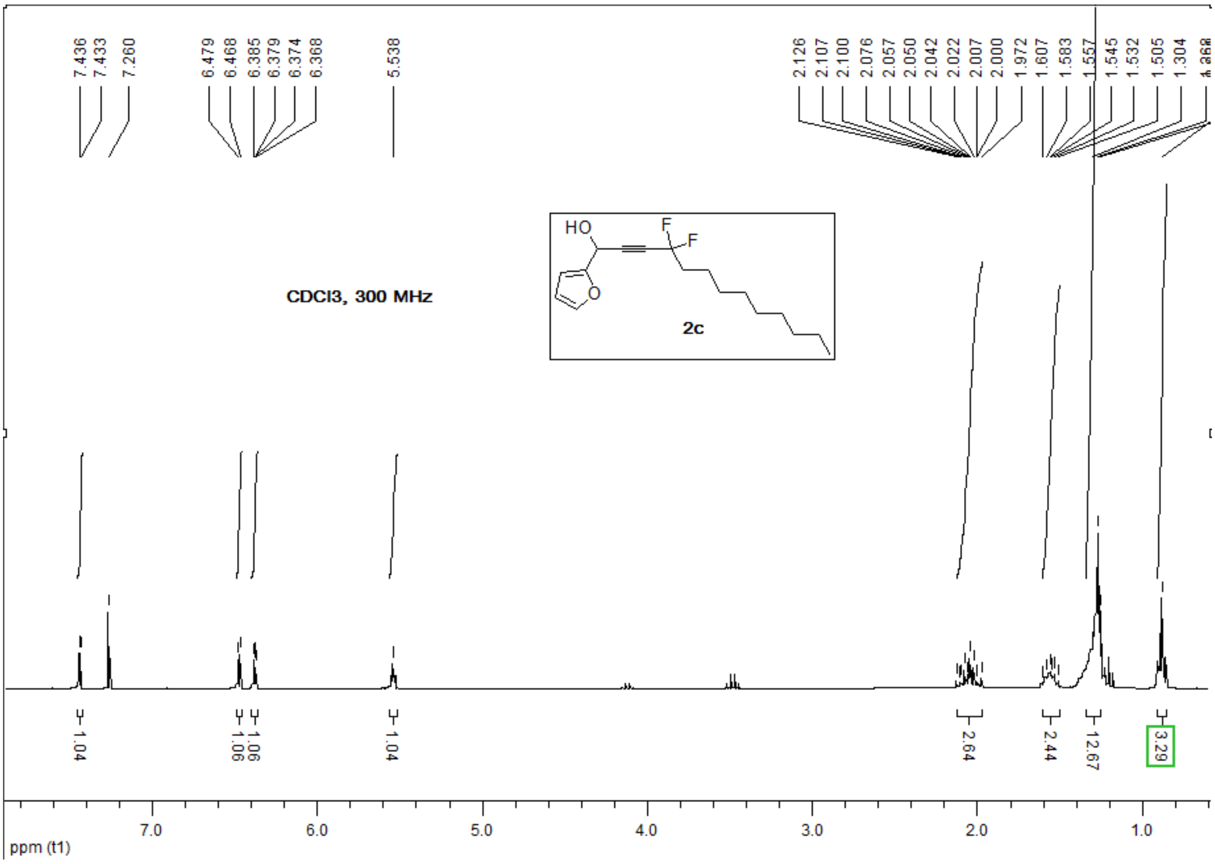
7.436 7.433 7.260 6.479 6.468 6.385 6.379 6.374 6.368 5.538 2.126 2.107 2.100 2.076 2.057 2.050 2.042 2.022 2.007 2.000 1.972 1.607 1.583 1.557 1.545 1.532 1.505 1.304
CDCl3, 300 MHz
HO
F
F
O
2c
1.04
1.06
1.06
1.04
2.64
2.44
12.67
3.29
7.0
6.0
5.0
4.0
3.0
2.0
1.0
ppm (t1)

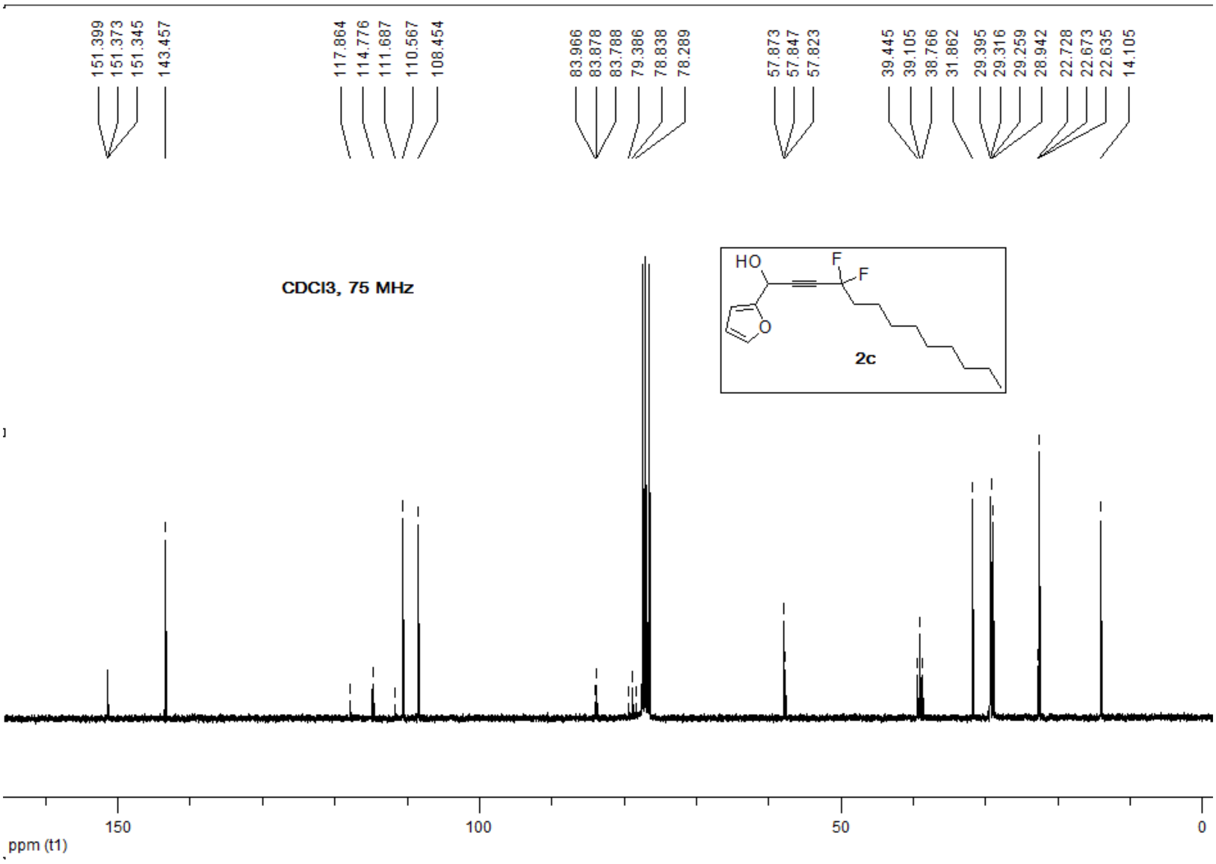
151.399 151.373 151.345 143.457 117.864 114.776 111.687 110.567 108.454 83.966 83.878 83.788 79.386 78.838 78.289 57.873 57.847 57.823 39.445 39.105 38.766 31.862 29.395 29.316 29.259 28.942 22.728 22.673 22.635 14.105
CDCl3, 75 MHz
HO
F
F
O
2c
150
100
50
0
ppm (t1)

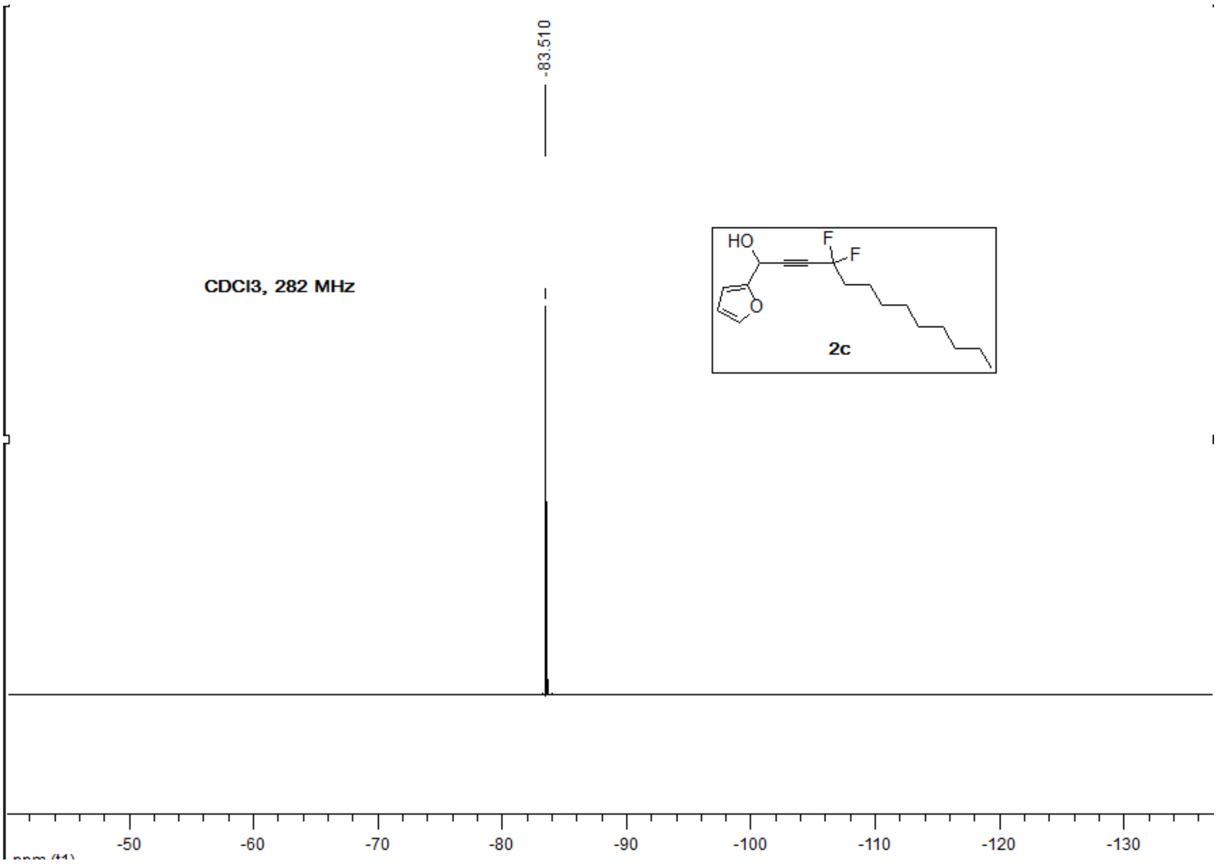
-83.510
CDCl3, 282 MHz
HO
F
F
2c
-50
-60
-70
-80
-90
-100
-110
-120
-130

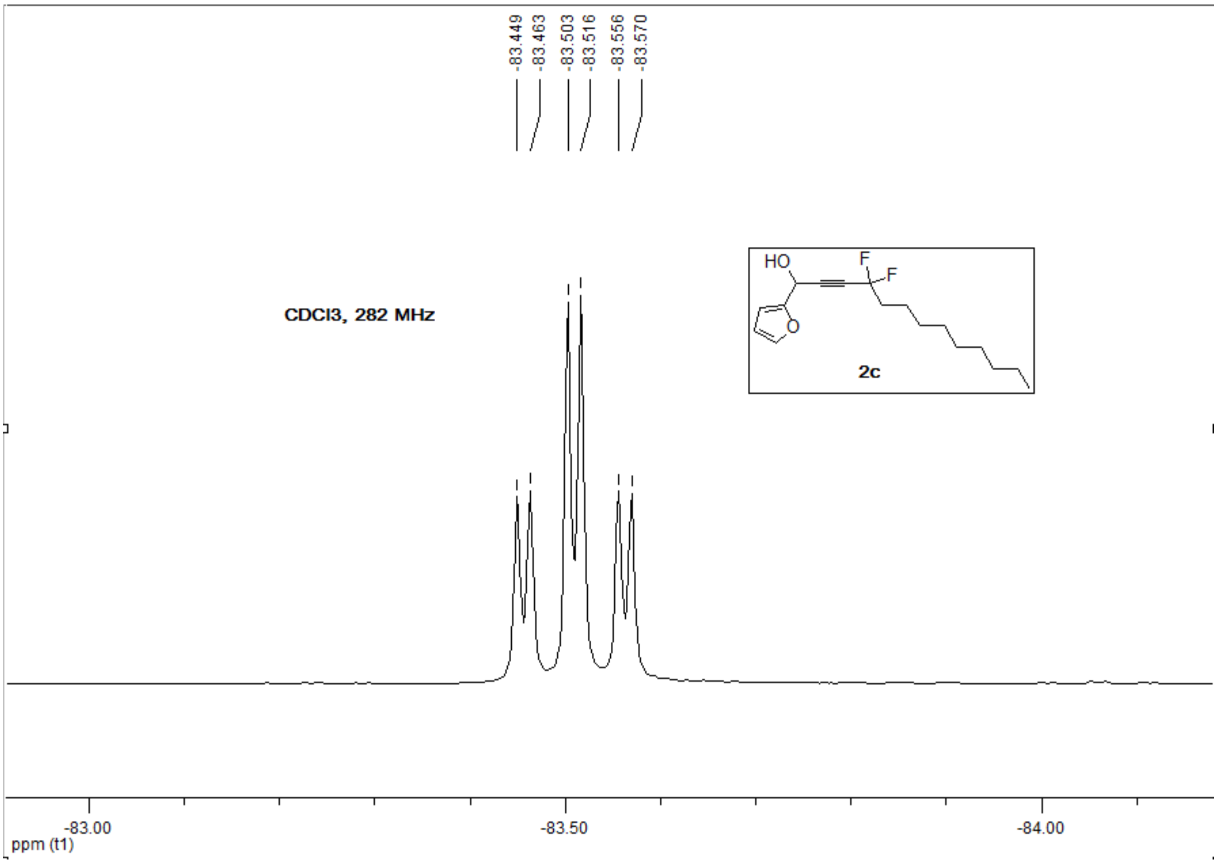
-83.449
-83.463
-83.503
-83.516
-83.556
-83.570
CDCl3, 282 MHz
HO
F
F
2c
-83.00
-83.50
-84.00
ppm (t1)

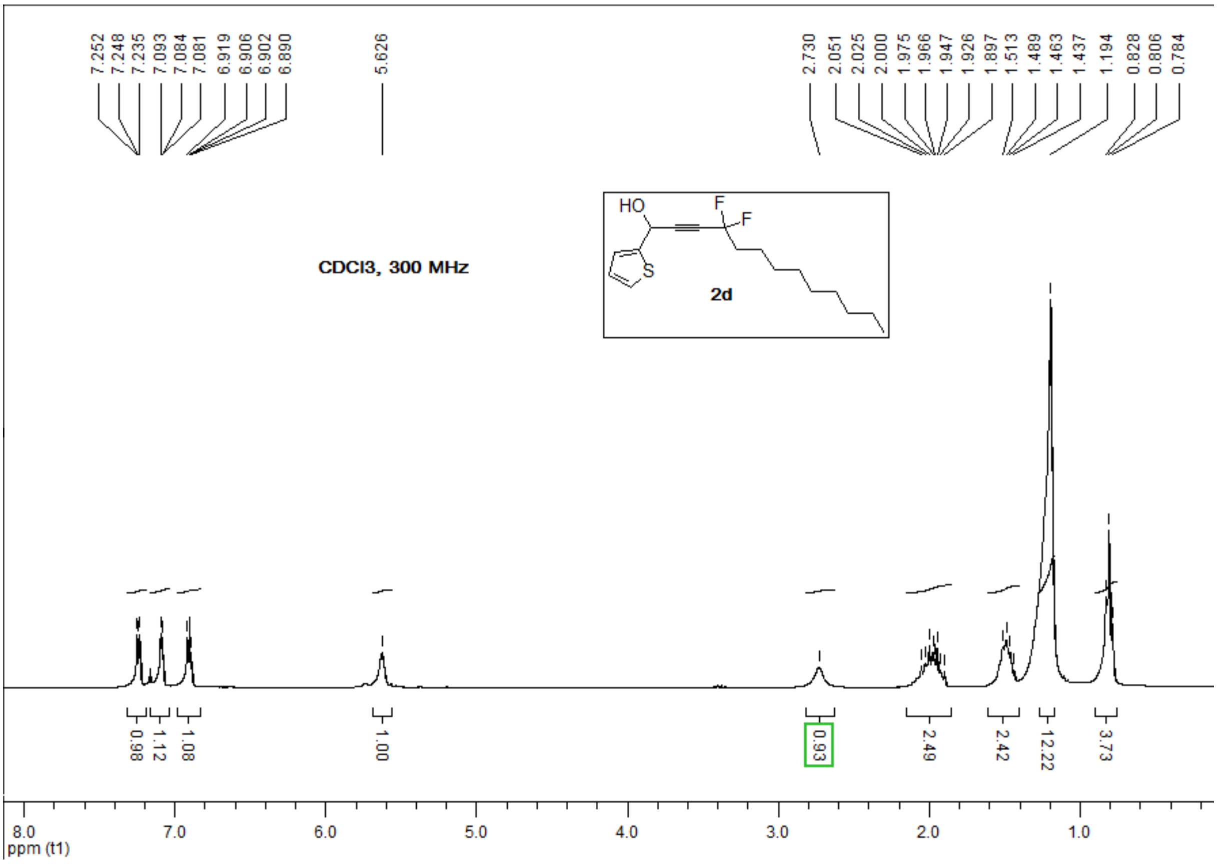
7.252
7.248
7.235
7.093
7.084
7.081
6.919
6.906
6.902
6.890
5.626
2.730
2.051
2.025
2.000
1.975
1.966
1.947
1.926
1.897
1.513
1.489
1.463
1.437
1.194
0.828
0.806
0.784
CDCl3, 300 MHz
HO
F
F
S
2d
0.98
1.12
1.08
1.00
0.93
2.49
2.42
12.22
3.73
8.0
7.0
6.0
5.0
4.0
3.0
2.0
1.0
ppm (t1)

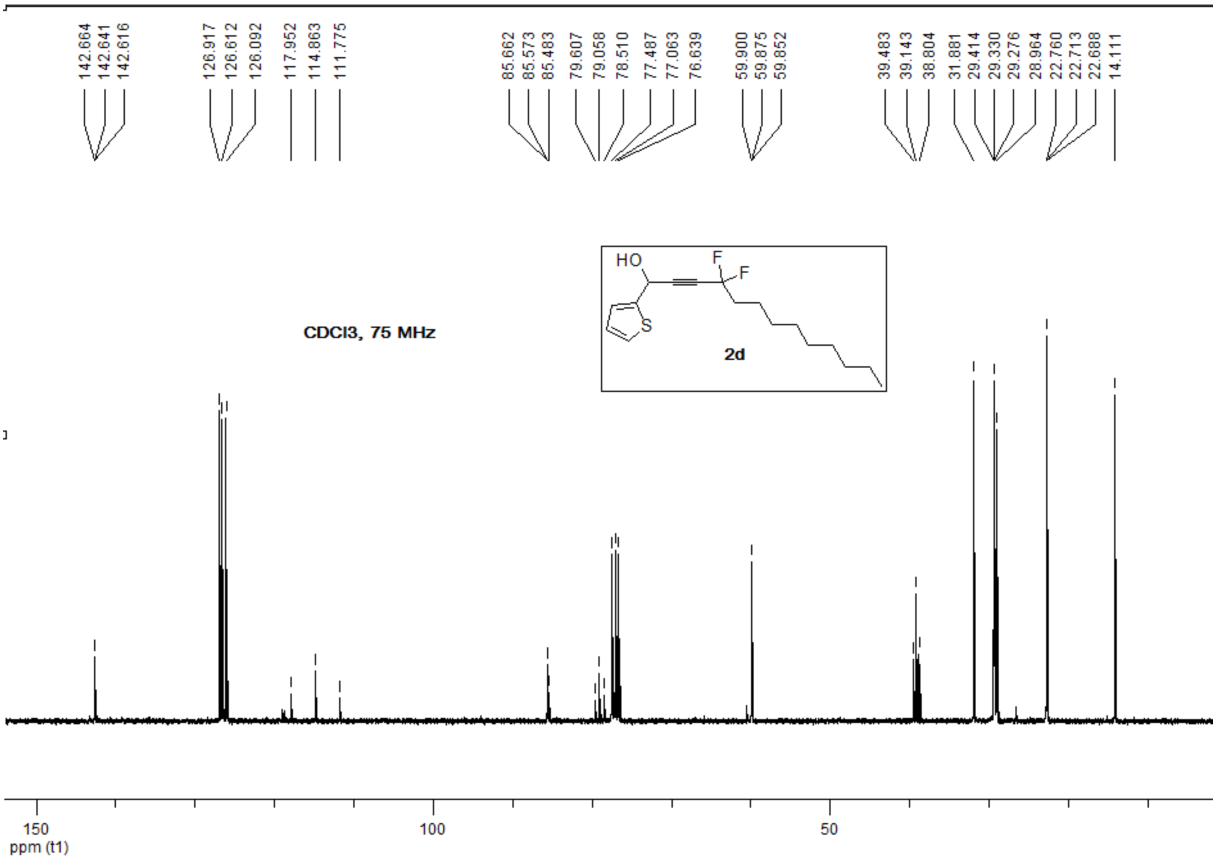
142.664
142.641
142.616
126.917
126.612
126.092
117.952
114.863
111.775
85.662
85.573
85.483
79.607
79.058
78.510
77.487
77.063
76.639
59.900
59.875
59.852
39.483
39.143
38.804
31.881
29.414
29.330
29.276
28.964
22.760
22.713
22.688
14.111
CDCl3, 75 MHz
HO
F
F
S
2d
150
100
50
ppm (t1)

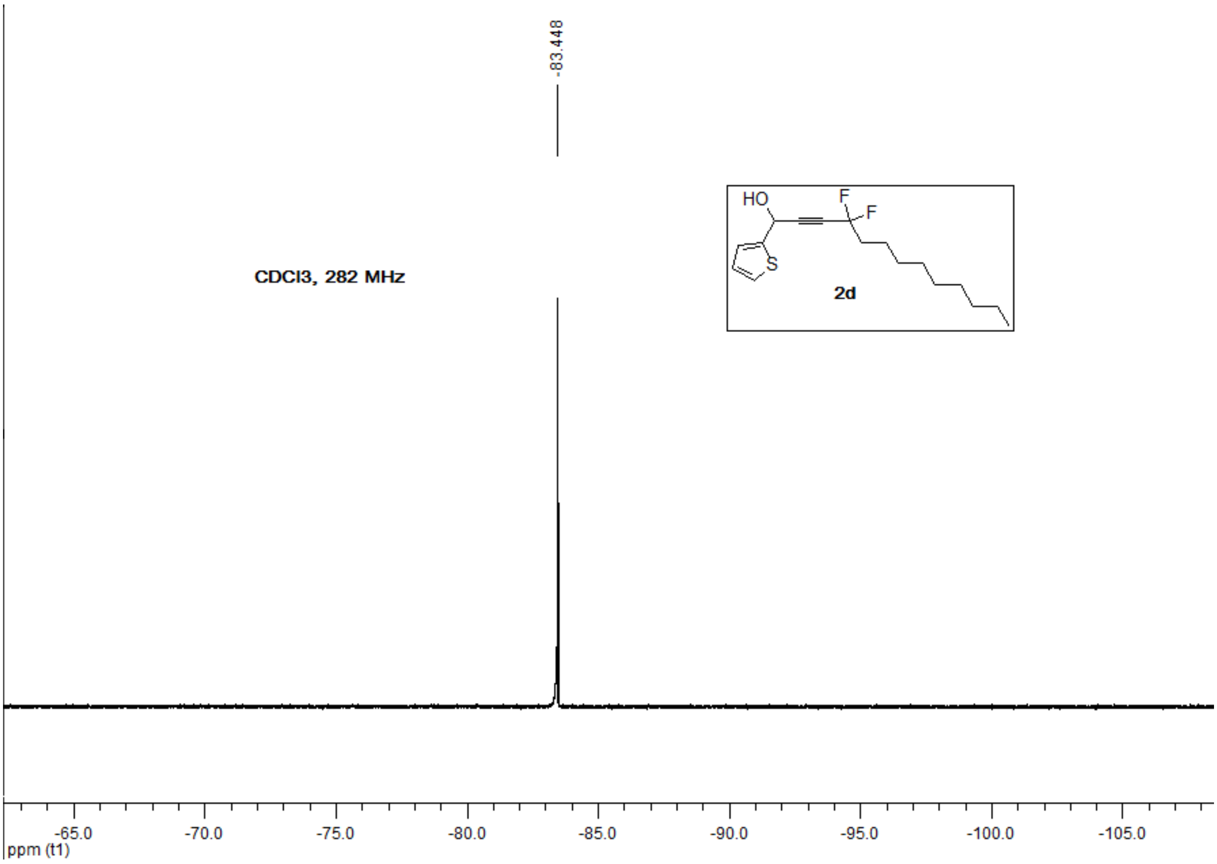
-83.448
CDCl3, 282 MHz
HO
F
F
S
2d
-65.0
-70.0
-75.0
-80.0
-85.0
-90.0
-95.0
-100.0
-105.0
ppm (t1)

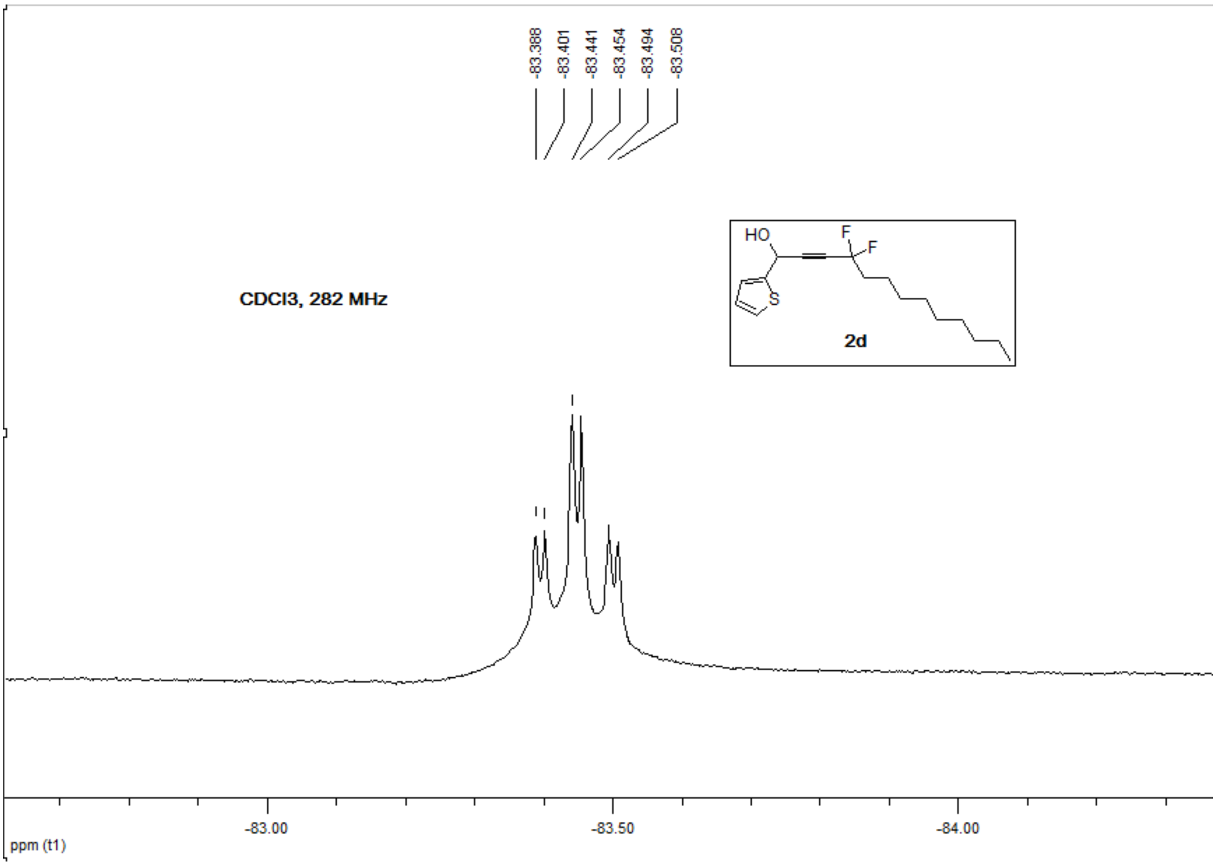
-83.388
-83.401
-83.441
-83.454
-83.494
-83.508
CDCl3, 282 MHz
HO
F
F
S
2d
-83.00
-83.50
-84.00
ppm (t1)

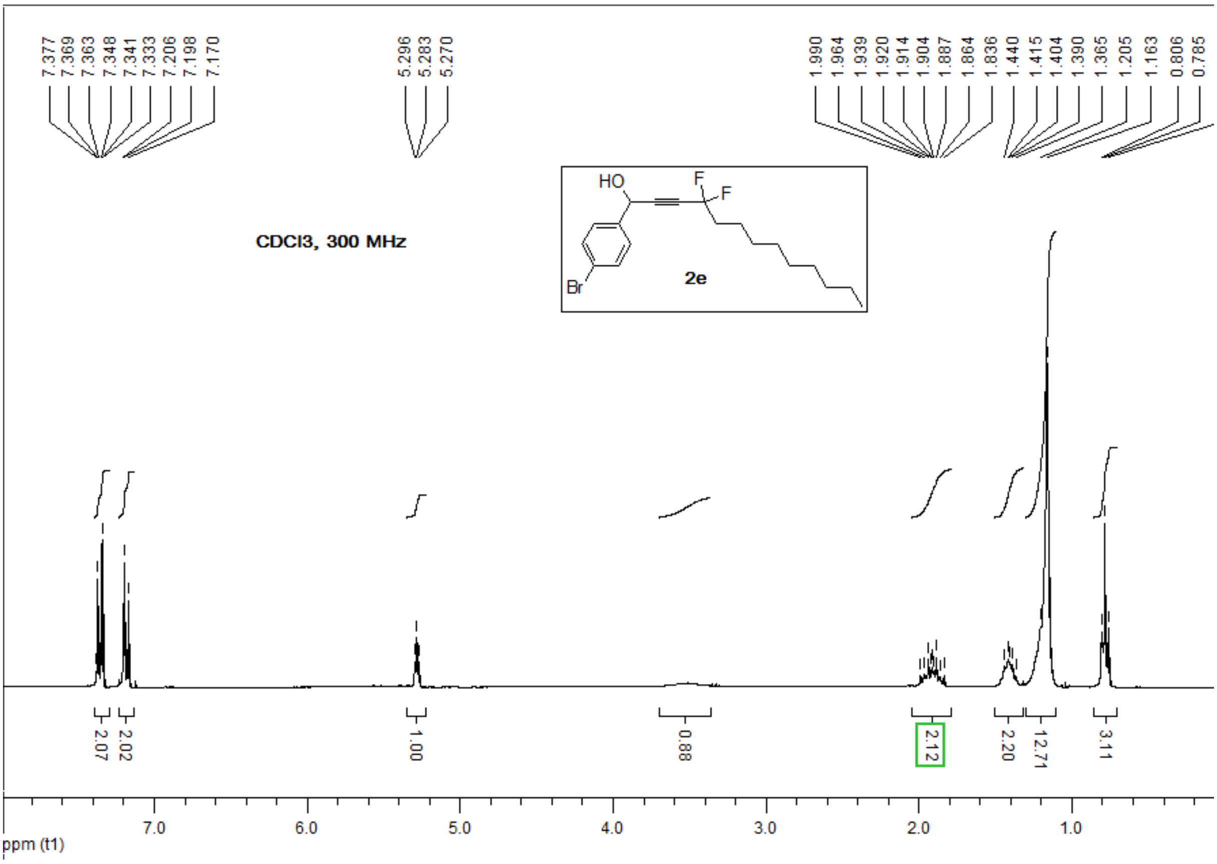

7.377
7.369
7.363
7.348
7.341
7.333
7.206
7.198
7.170
5.296
5.283
5.270
1.990
1.964
1.939
1.920
1.914
1.904
1.887
1.864
1.836
1.440
1.415
1.404
1.390
1.365
1.205
1.163
0.806
0.785
CDCl3, 300 MHz
HO
F
F
Br
2e
2.07
2.02
1.00
0.88
2.12
2.20
12.71
3.11
7.0
6.0
5.0
4.0
3.0
2.0
1.0
ppm (t1)

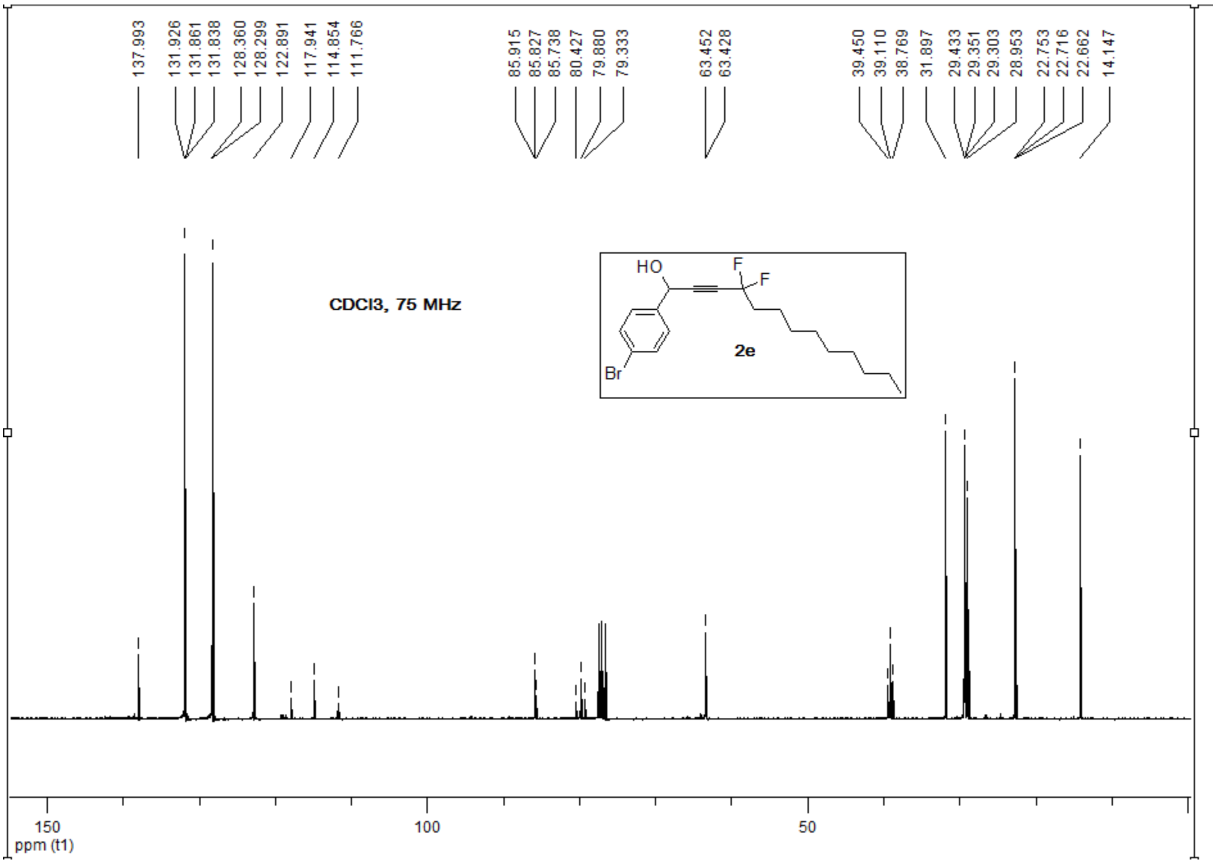

137.993
131.926
131.861
131.838
128.360
128.299
122.891
117.941
114.854
111.766
85.915
85.827
85.738
80.427
79.880
79.333
63.452
63.428
39.450
39.110
38.769
31.897
29.433
29.351
29.303
28.953
22.753
22.716
22.662
14.147
CDCl3, 75 MHz
HO
F
F
Br
2e
150
100
50
ppm (t1)

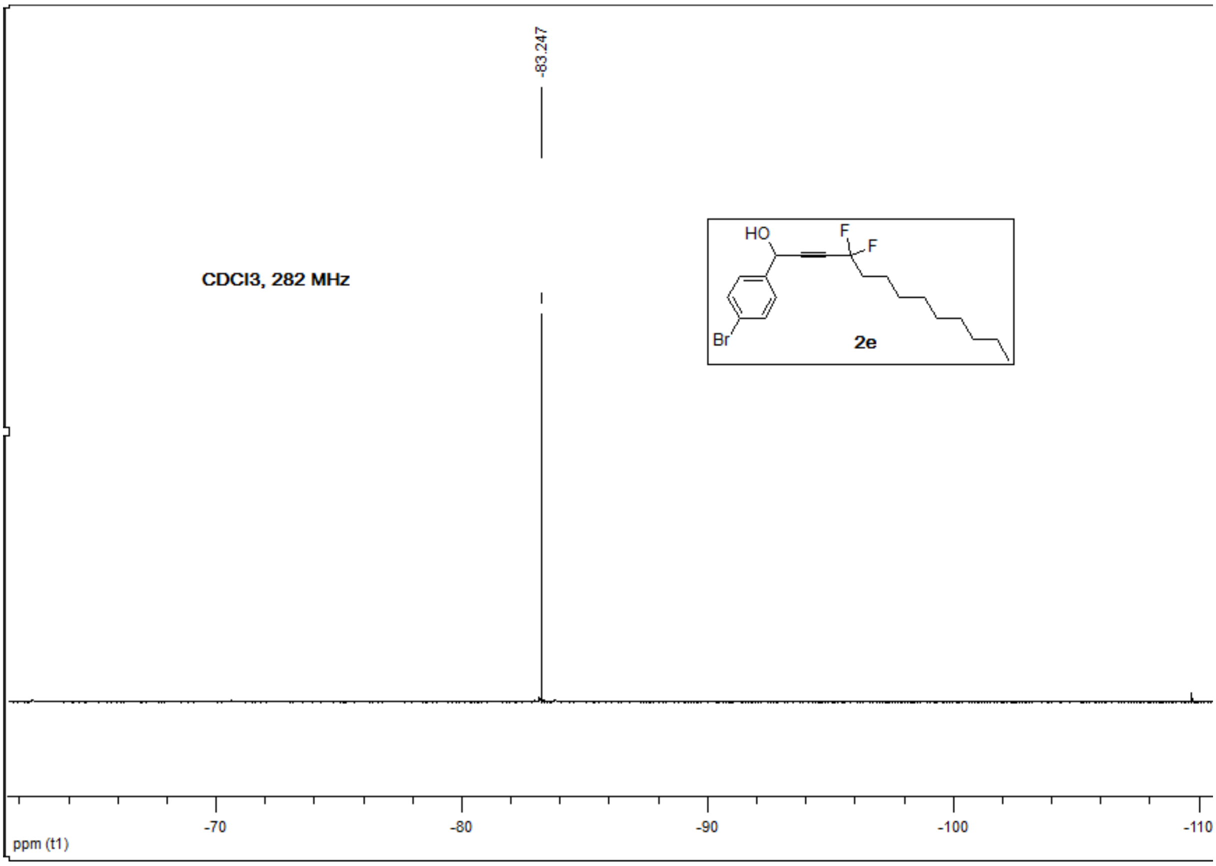
-83.247
CDCl3, 282 MHz
HO
F
F
Br
2e
-70
-80
-90
-100
-110
ppm (t1)

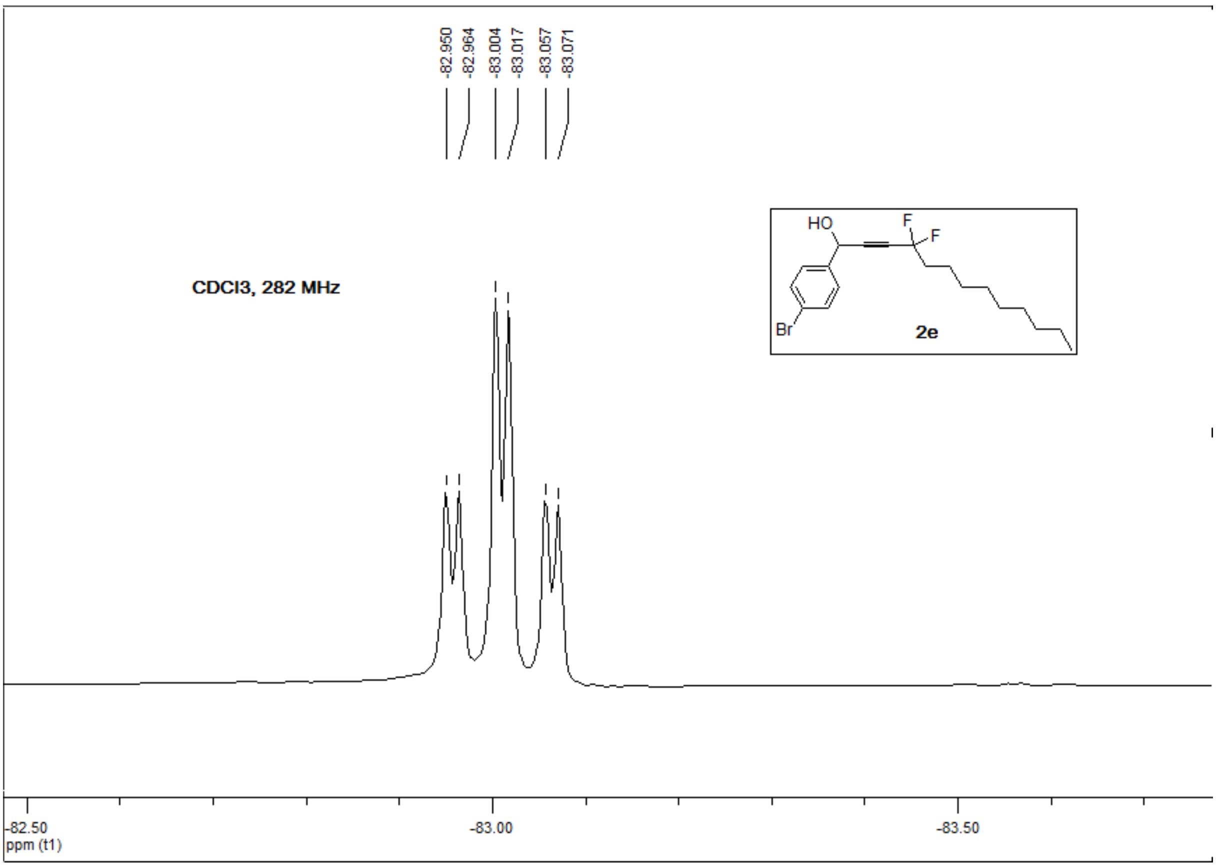
-82.950
-82.964
-83.004
-83.017
-83.057
-83.071
CDCl3, 282 MHz
HO
F
F
Br
2e
-82.50
-83.00
-83.50
ppm (t1)

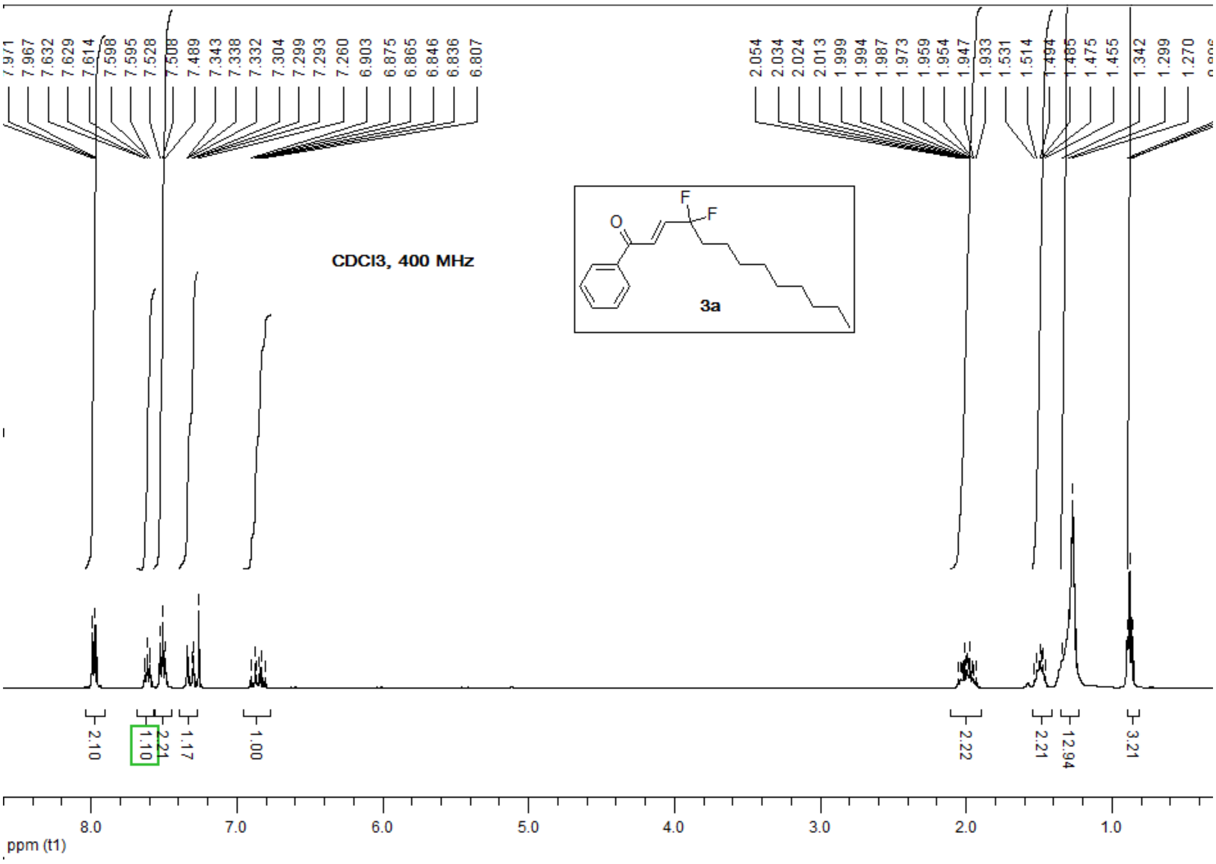
CDCl3, 400 MHz
3a
ppm (t1)

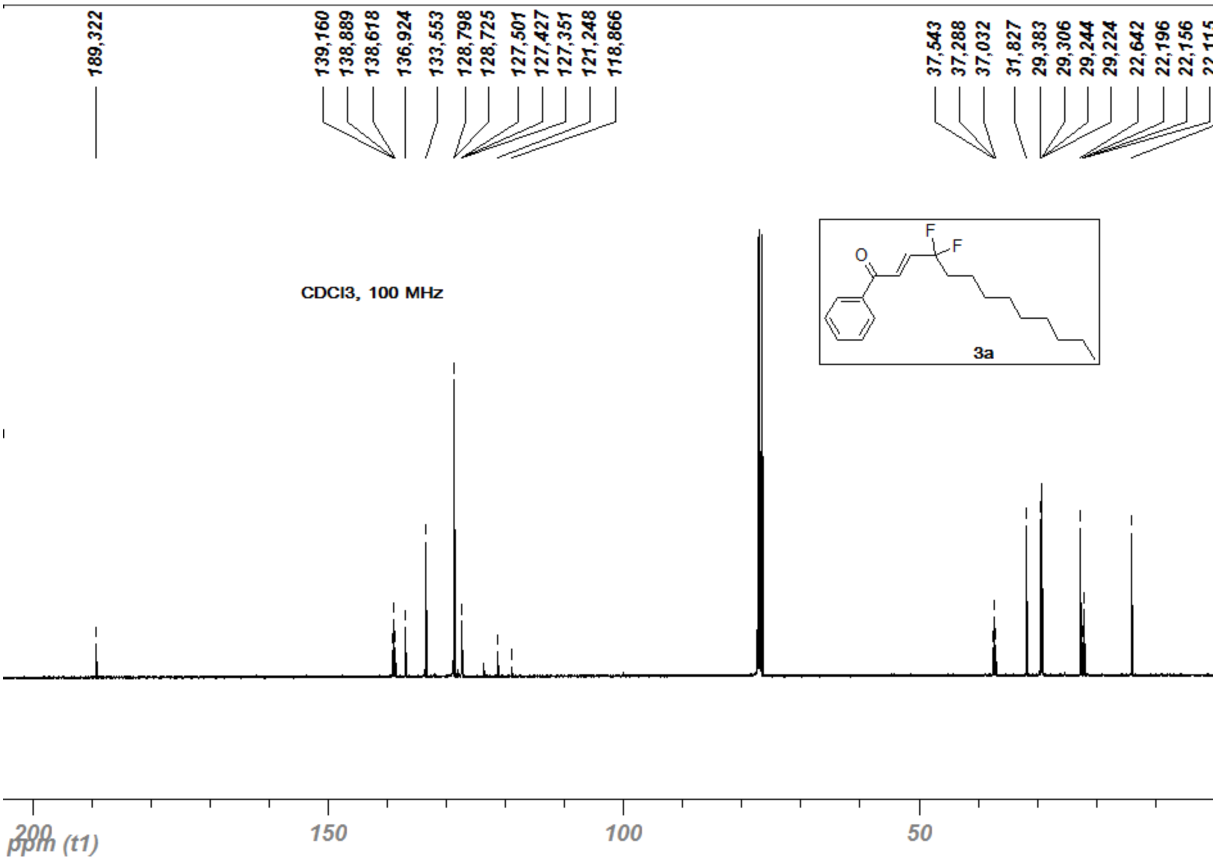
CDCl3, 100 MHz
3a
ppm (t1)

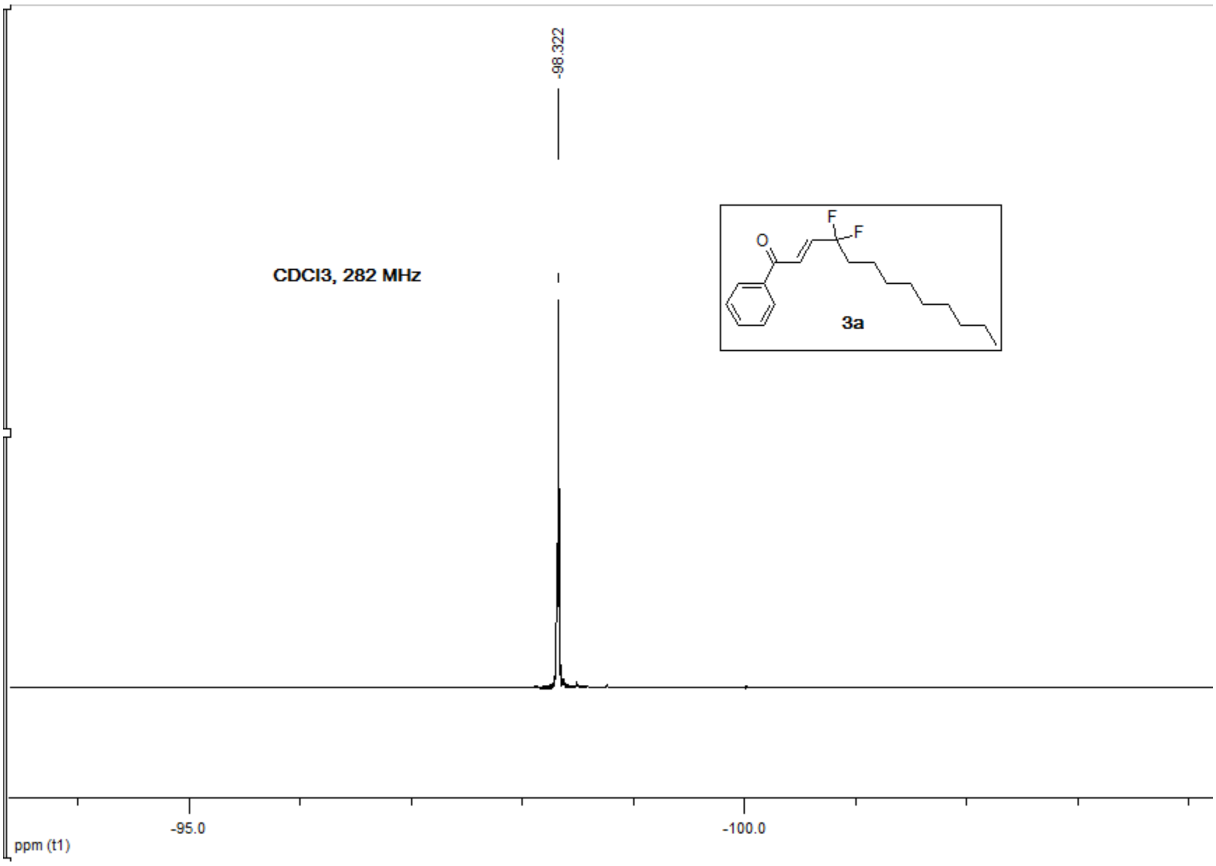
-98.322
CDCl3, 282 MHz
O
F
F
3a
-95.0
-100.0
ppm (t1)

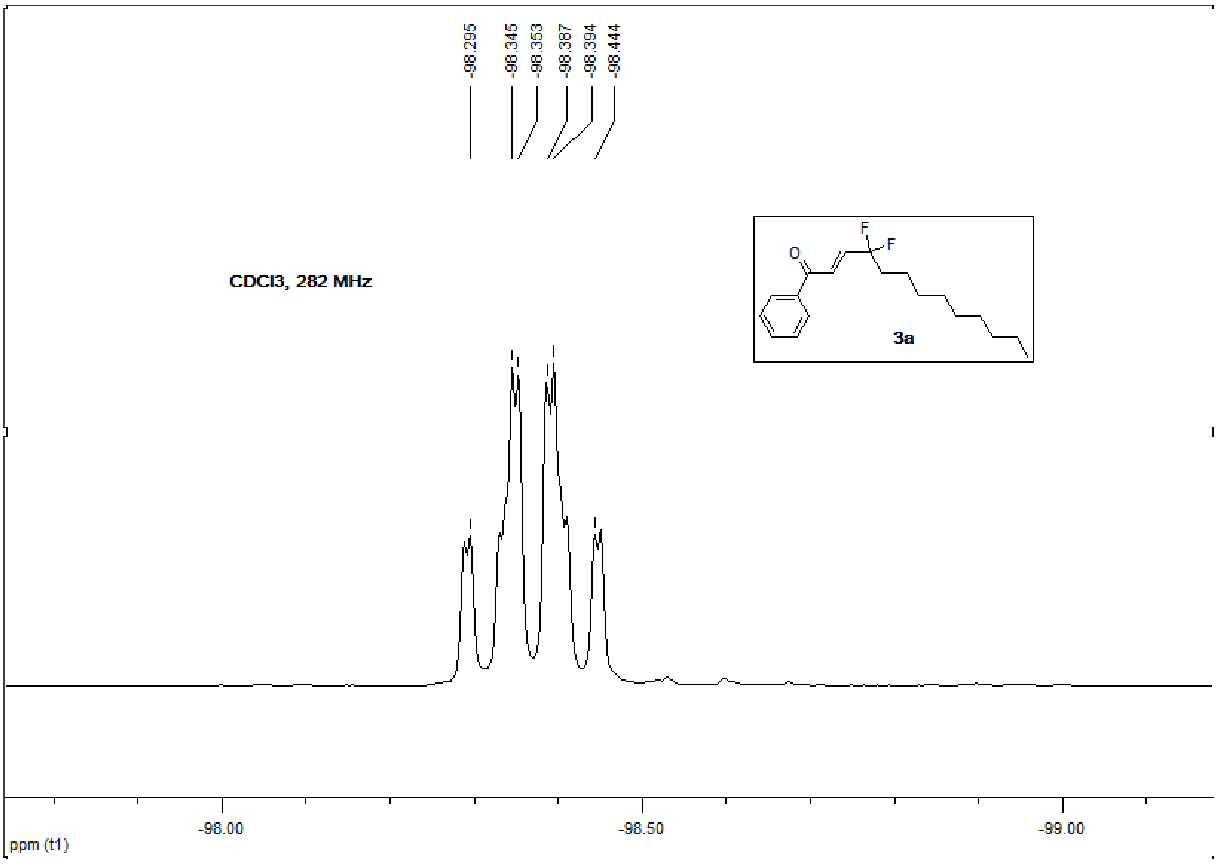
-98.295
-98.345
-98.353
-98.387
-98.394
-98.444
CDCl3, 282 MHz
O
F
F
3a
-98.00
-98.50
-99.00
ppm (t1)

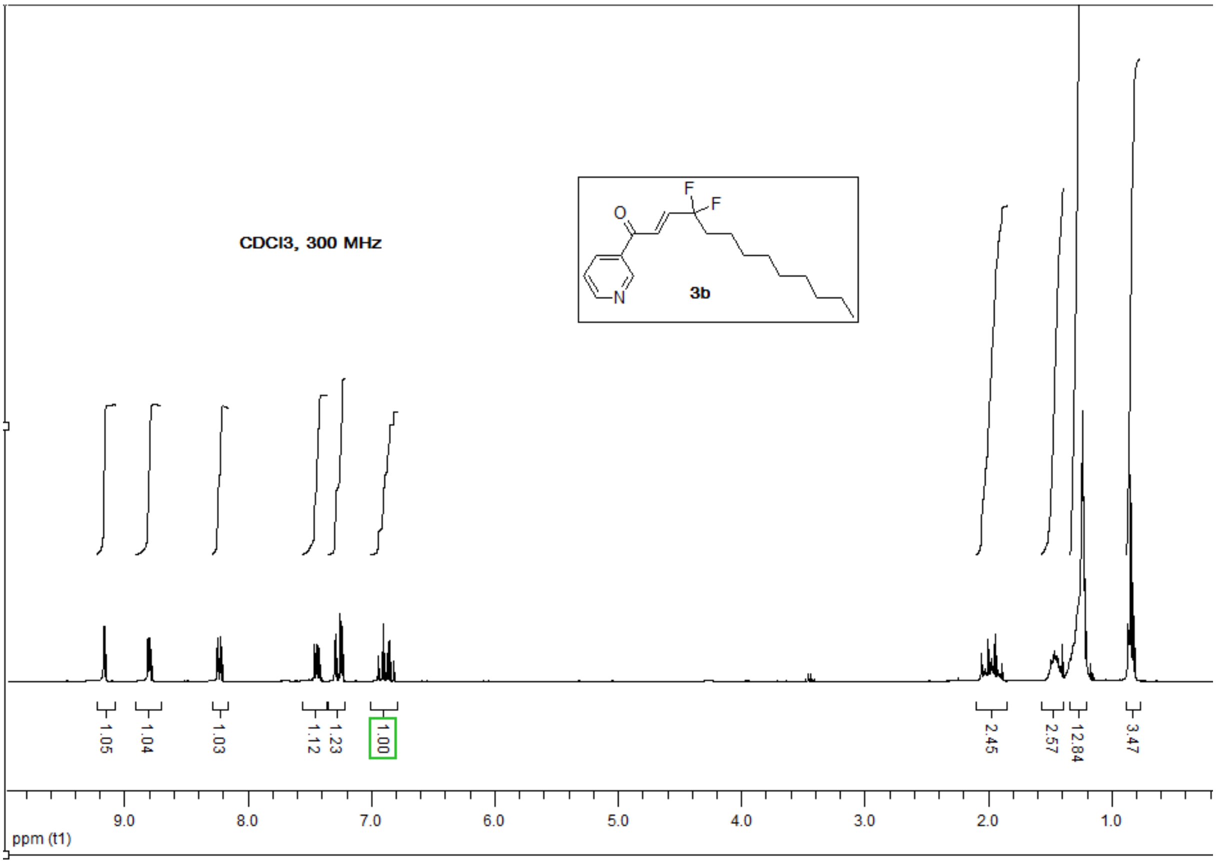

CDCl3, 300 MHz
3b
1.05
1.04
1.03
1.12
1.23
1.00
2.45
2.57
12.84
3.47
ppm (t1)

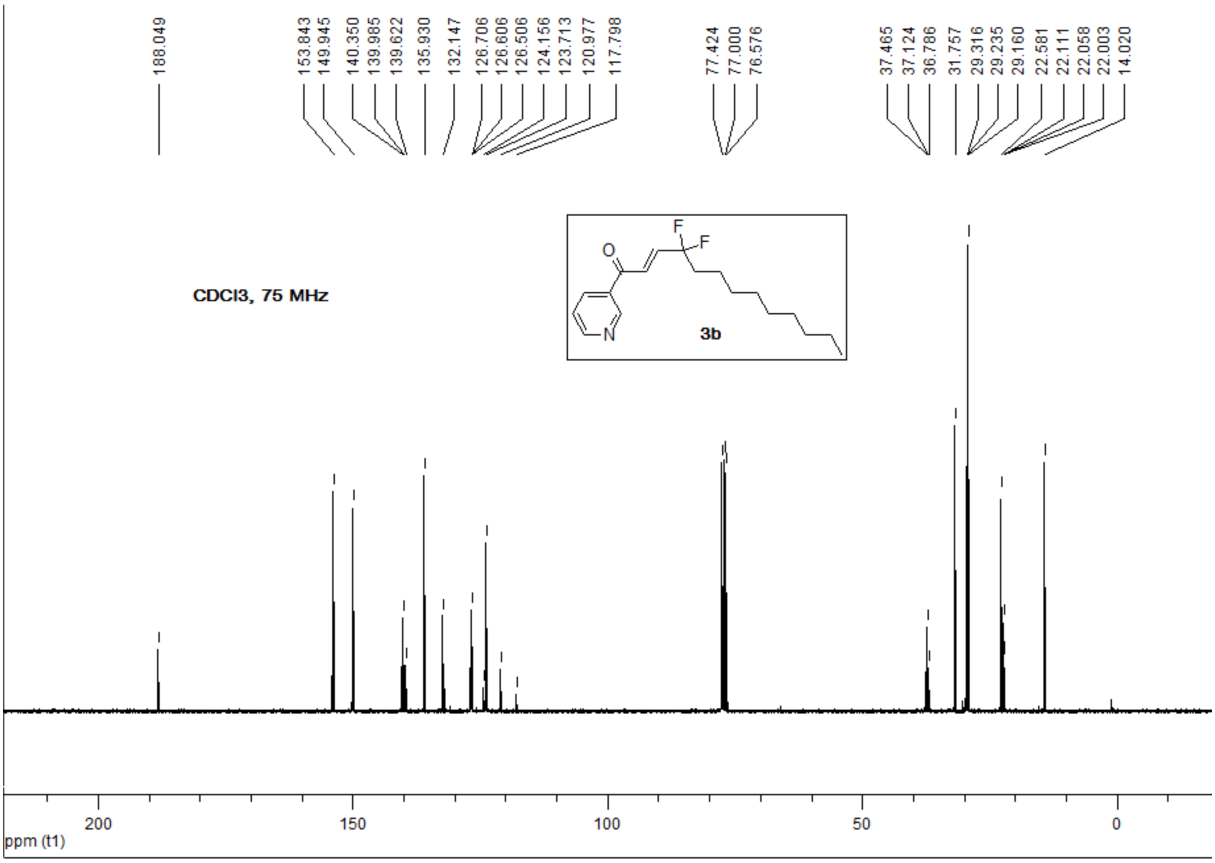

188.049
153.843
149.945
140.350
139.985
139.622
135.930
132.147
126.706
126.606
126.506
124.156
123.713
120.977
117.798
77.424
77.000
76.576
37.465
37.124
36.786
31.757
29.316
29.235
29.160
22.581
22.111
22.058
22.003
14.020
CDCl3, 75 MHz
3b
ppm (t1)

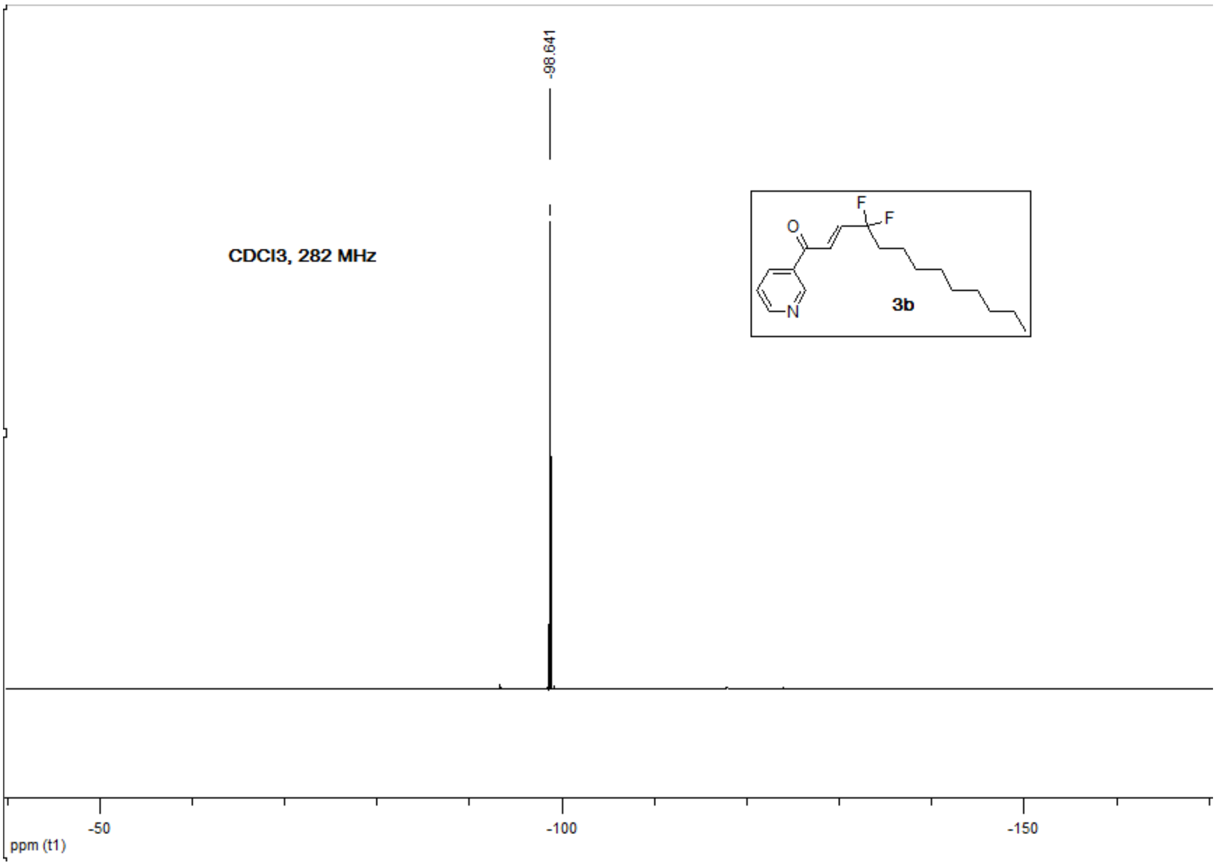
-98.641
CDCl3, 282 MHz
O
F
F
N
3b
-50
-100
-150
ppm (t1)

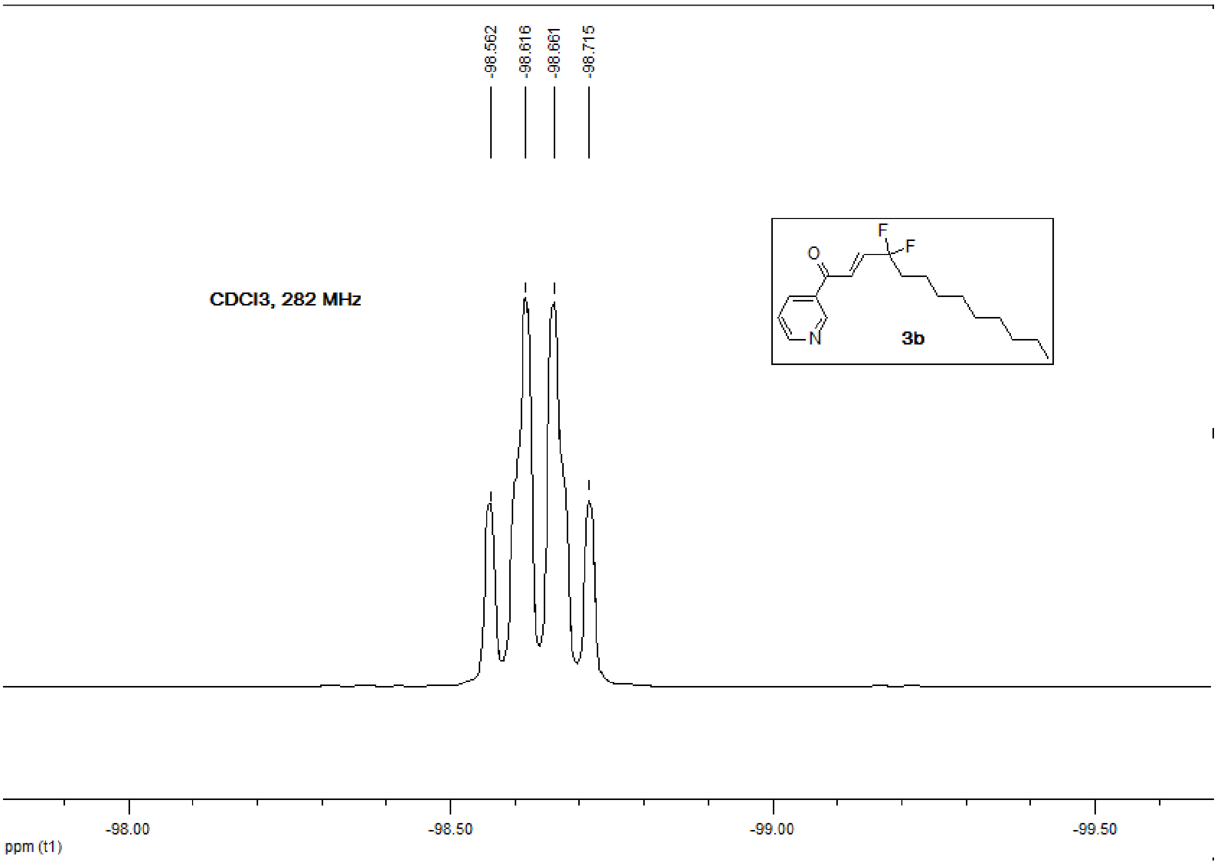
-98.562
-98.616
-98.661
-98.715
CDCl3, 282 MHz
O
F
F
N
3b
-98.00
-98.50
-99.00
-99.50
ppm (t1)

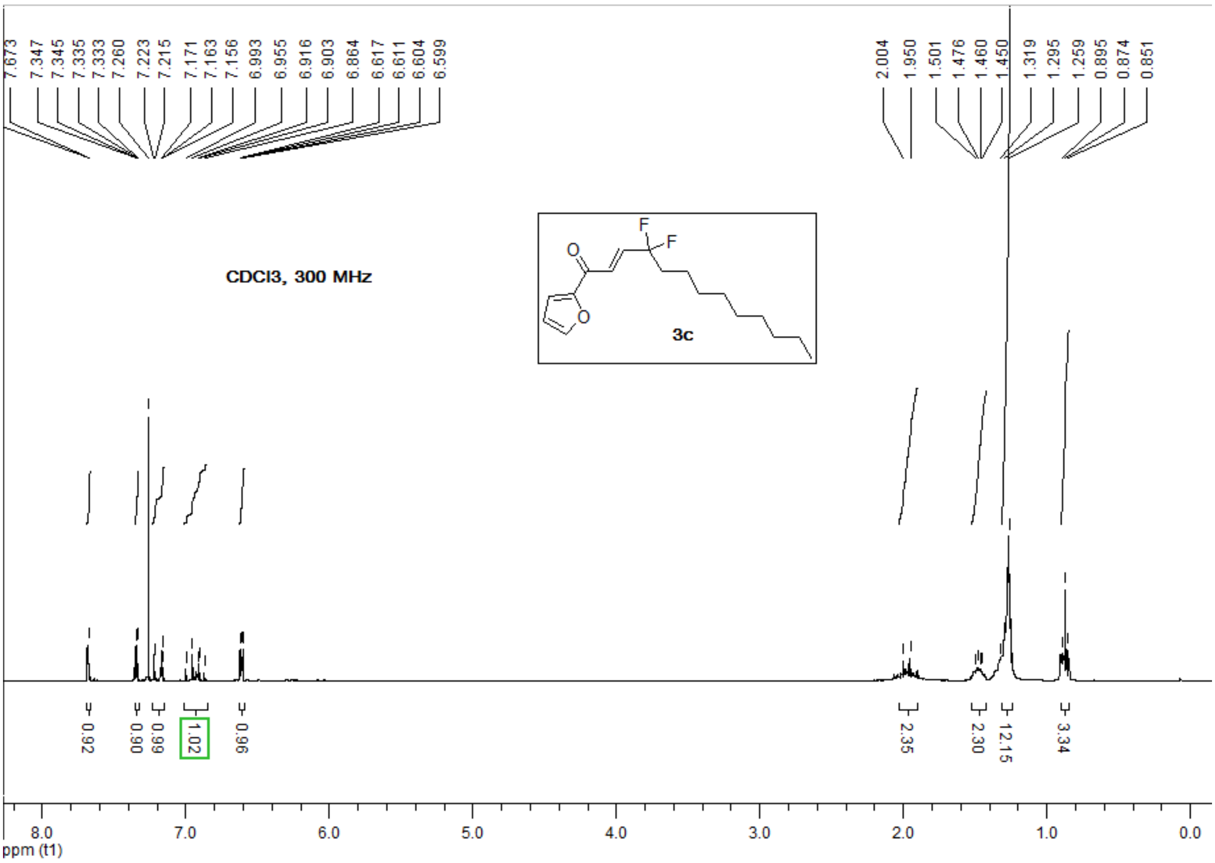
7.673
7.347
7.345
7.335
7.333
7.260
7.223
7.215
7.171
7.163
7.156
6.993
6.955
6.916
6.903
6.864
6.617
6.611
6.604
6.599
2.004
1.950
1.501
1.476
1.460
1.450
1.319
1.295
1.259
0.895
0.874
0.851
CDCl3, 300 MHz
3c
0.92
0.90
0.99
1.02
0.96
2.35
2.30
12.15
3.34
ppm (t1)

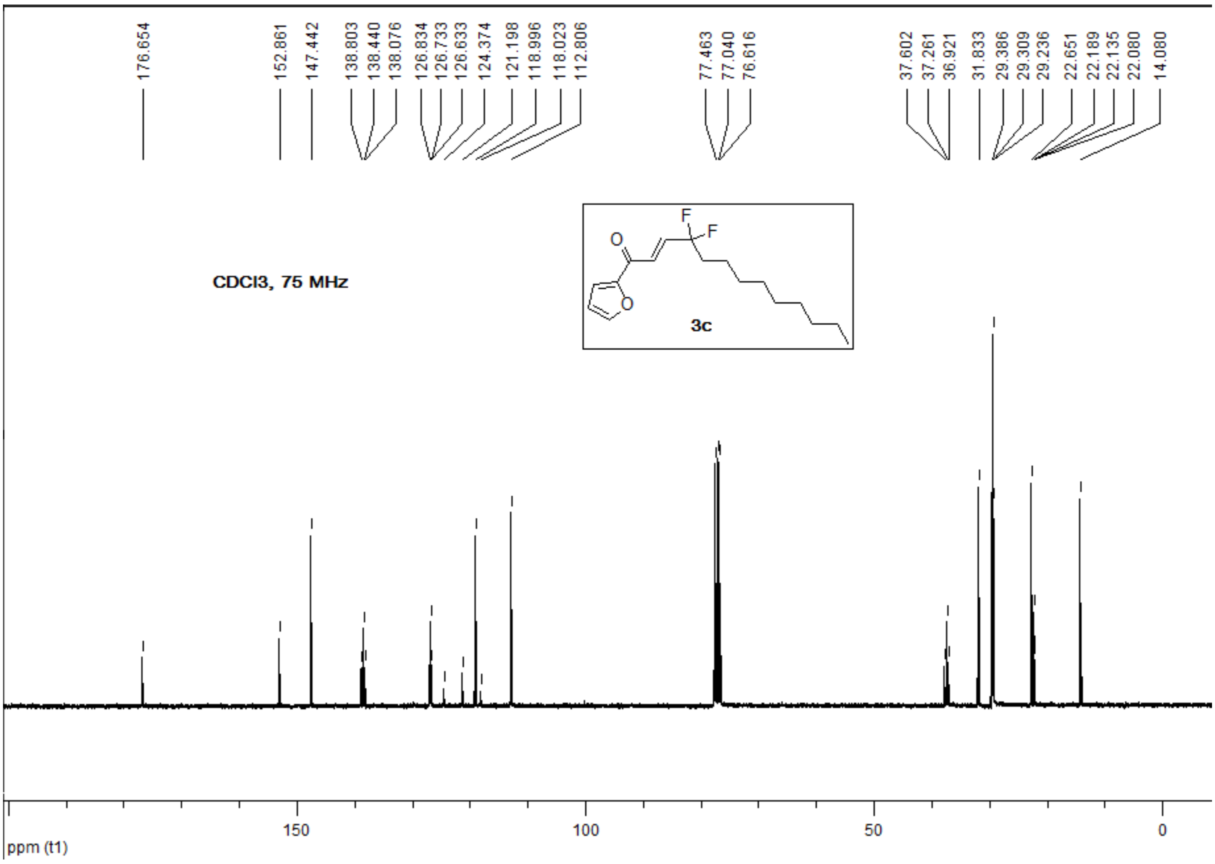
176.654
152.861
147.442
138.803
138.440
138.076
126.834
126.733
126.633
124.374
121.198
118.996
118.023
112.806
77.463
77.040
76.616
37.602
37.261
36.921
31.833
29.386
29.309
29.236
22.651
22.189
22.135
22.080
14.080
CDCl3, 75 MHz
3c
ppm (t1)

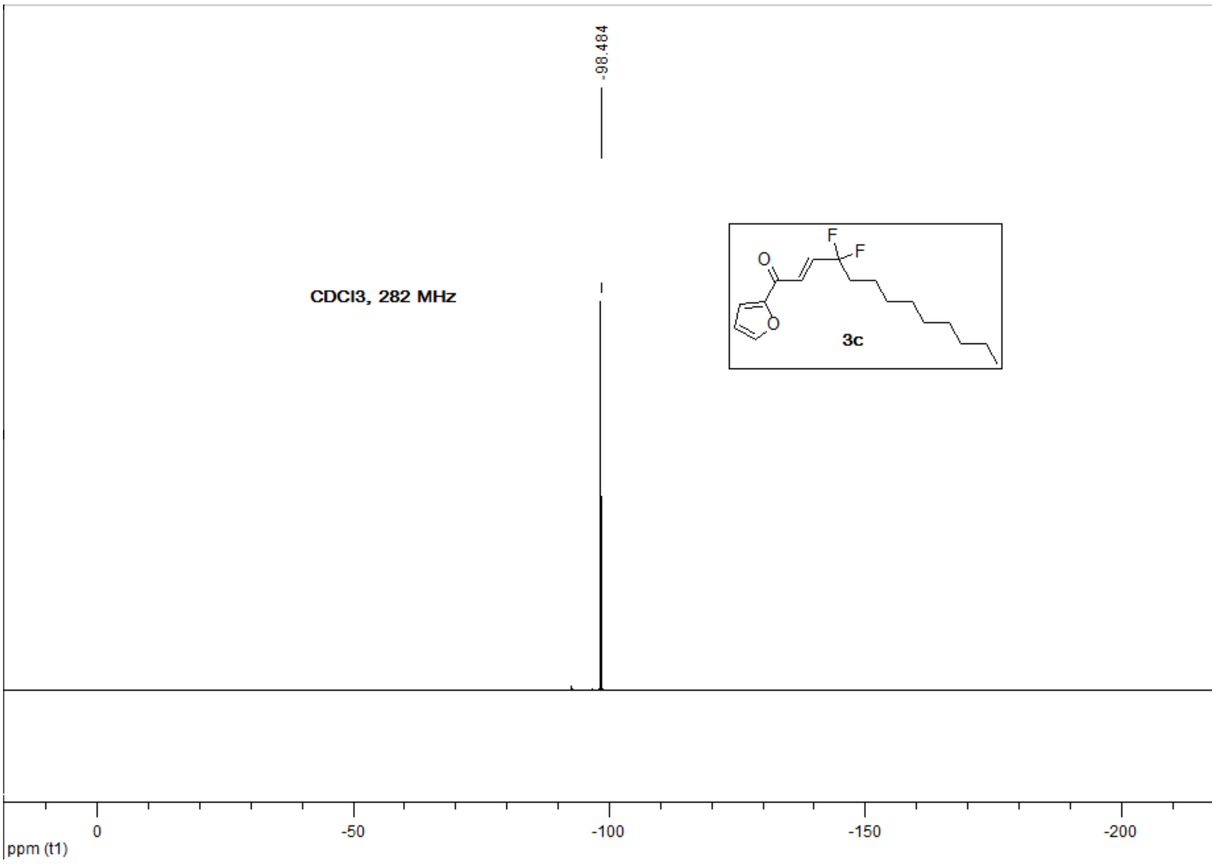
-98.484
CDCl3, 282 MHz
F
F
O
O
3c
0
-50
-100
-150
-200
ppm (t1)

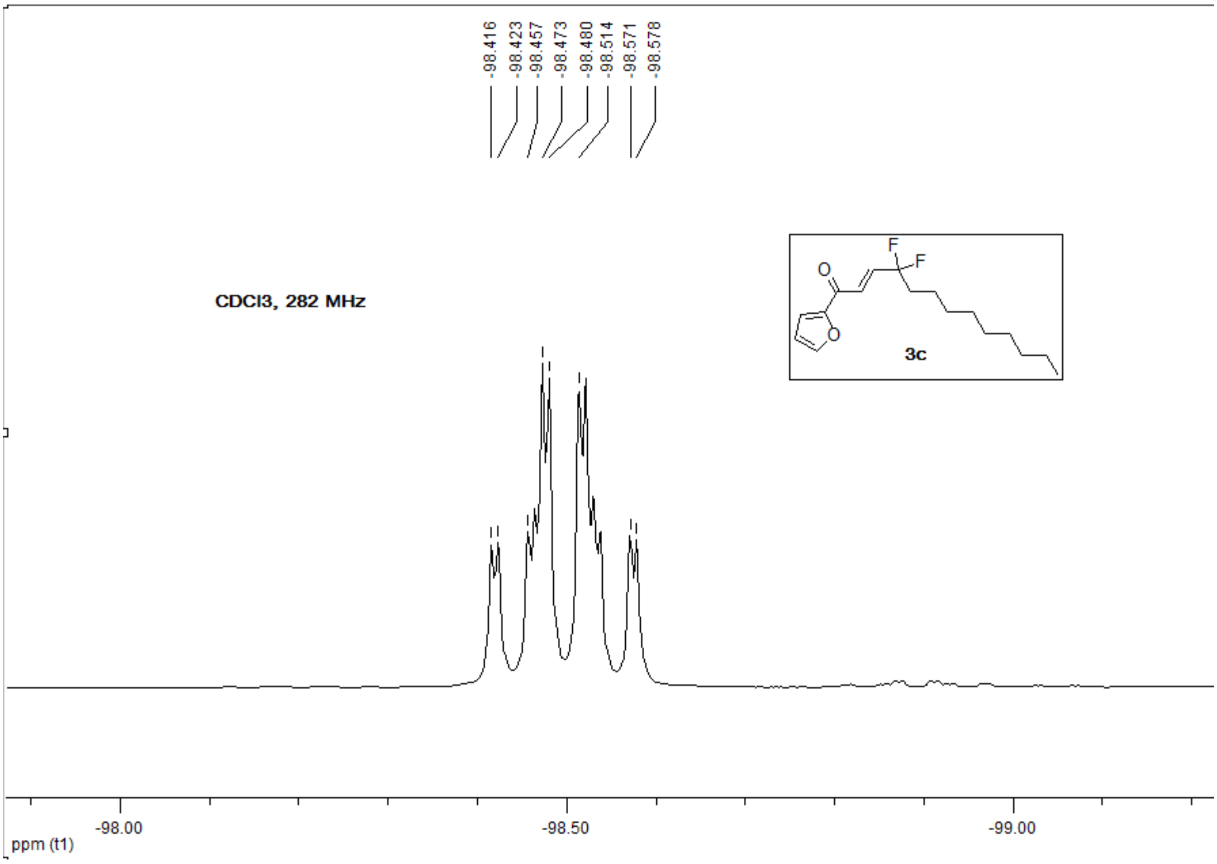
-98.416
-98.423
-98.457
-98.473
-98.480
-98.514
-98.571
-98.578
CDCl3, 282 MHz
F
F
O
O
3c
-98.00
-98.50
-99.00
ppm (t1)

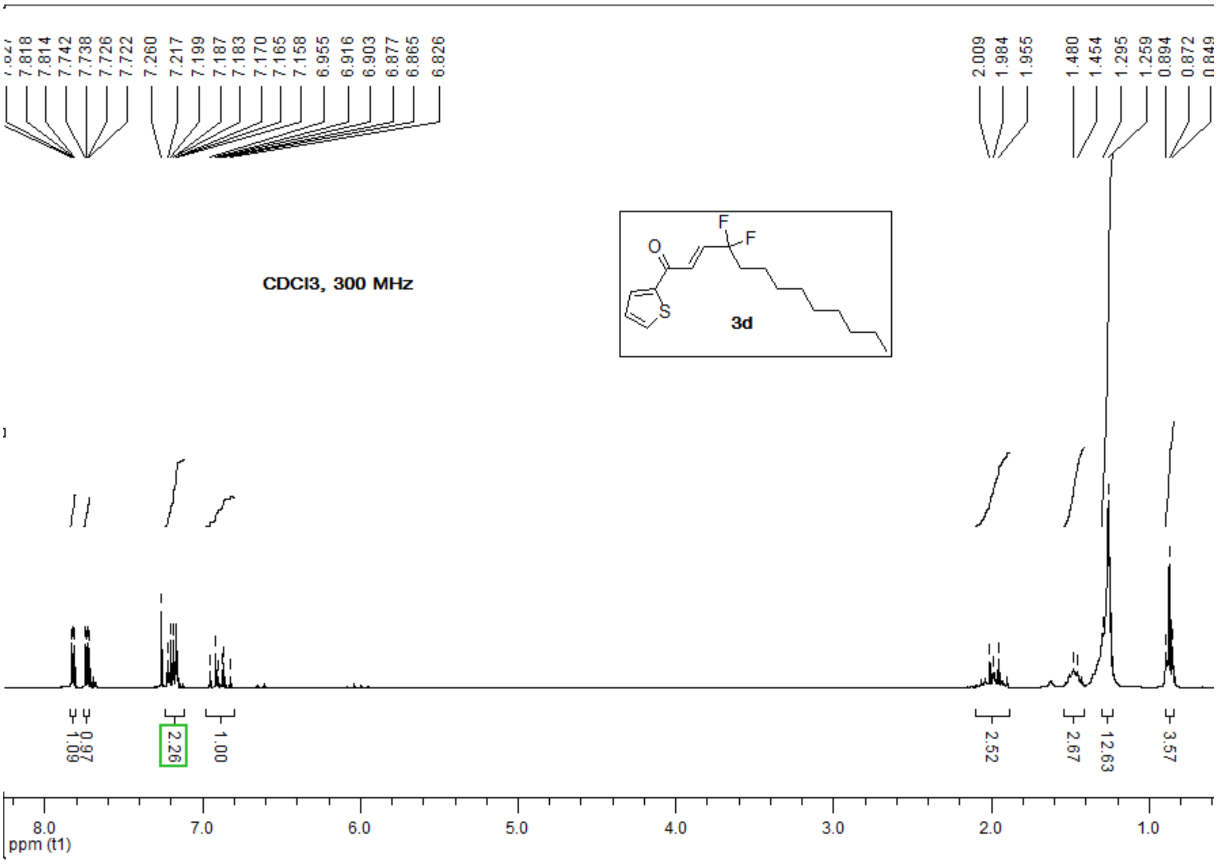
CDCl3, 300 MHz
3d
ppm (t1)
8.0
7.0
6.0
5.0
4.0
3.0
2.0
1.0

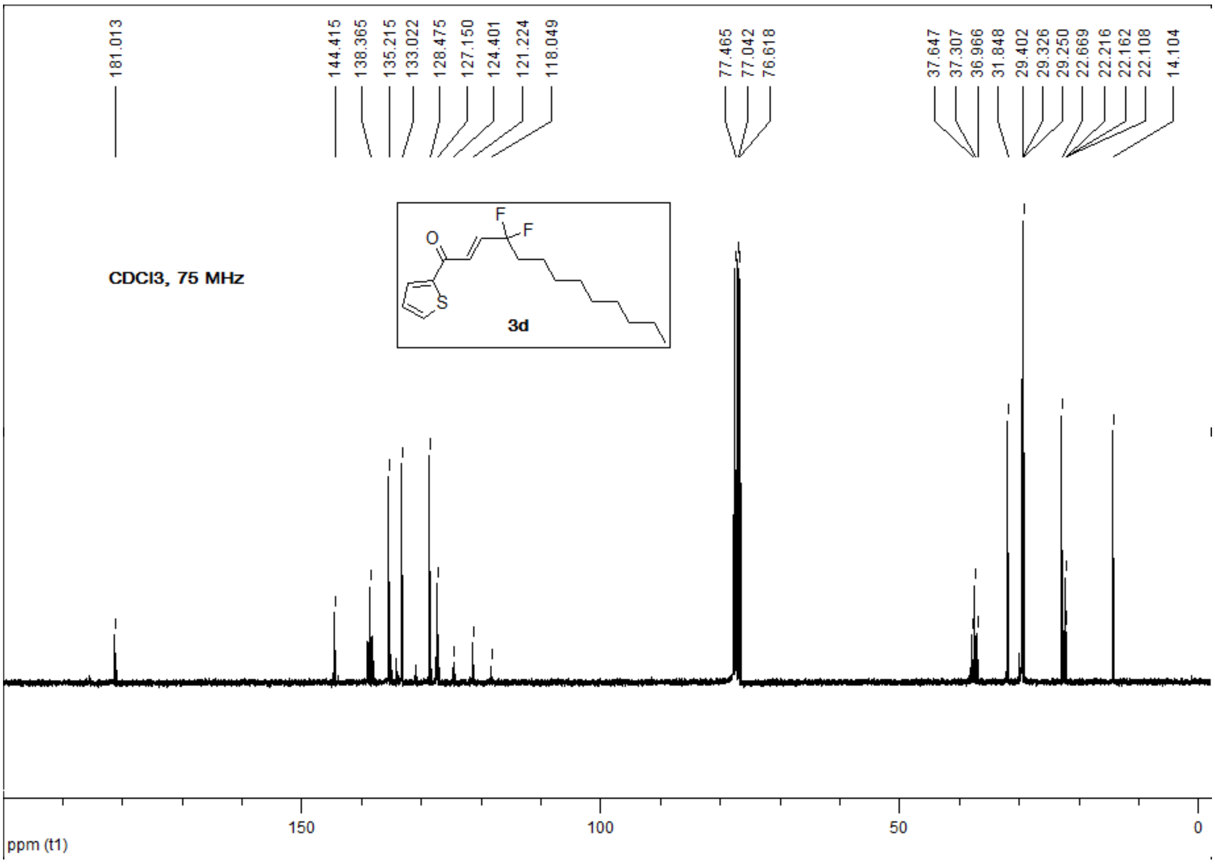
181.013
144.415
138.365
135.215
133.022
128.475
127.150
124.401
121.224
118.049
77.465
77.042
76.618
37.647
37.307
36.966
31.848
29.402
29.326
29.250
22.669
22.216
22.162
22.108
14.104
CDCl3, 75 MHz
3d
ppm (t1)
150
100
50
0

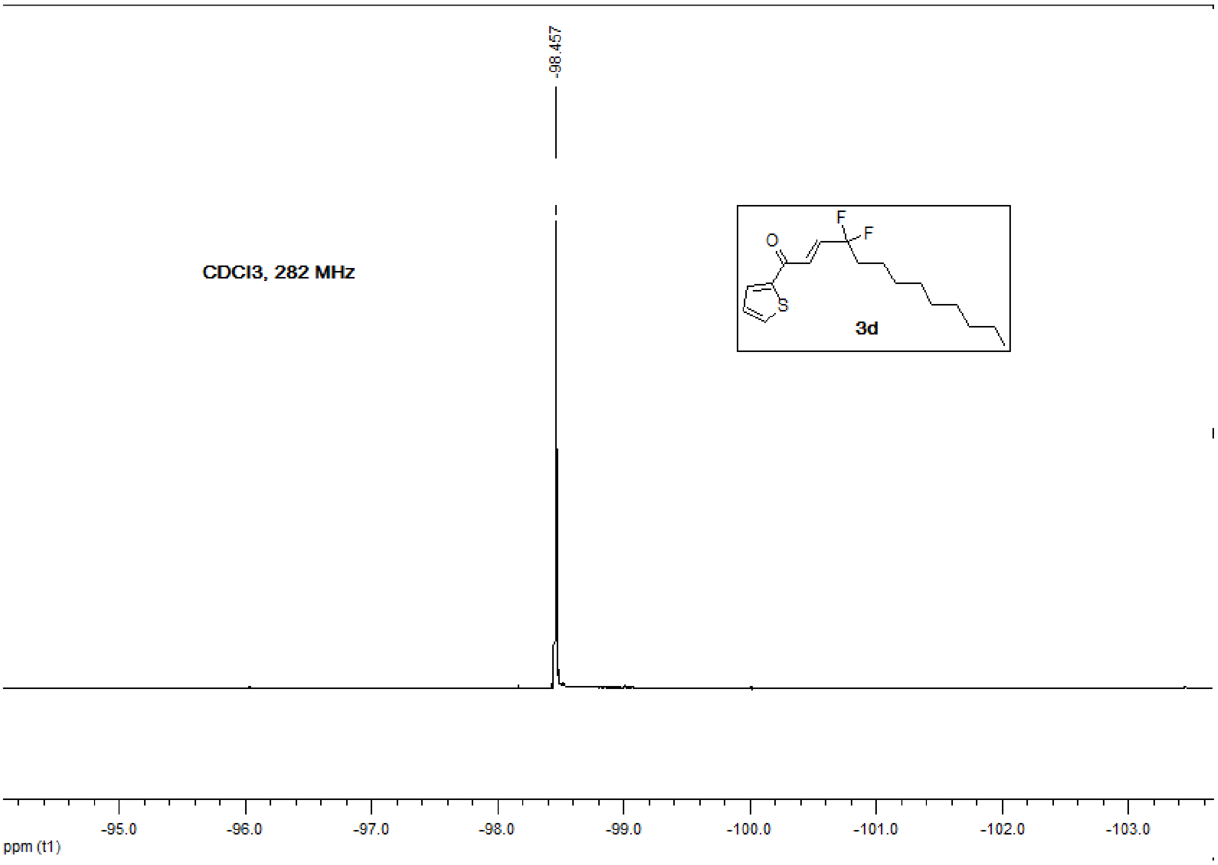
-98.457
CDCl3, 282 MHz
O
F
F
S
3d
-95.0
-96.0
-97.0
-98.0
-99.0
-100.0
-101.0
-102.0
-103.0
ppm (t1)

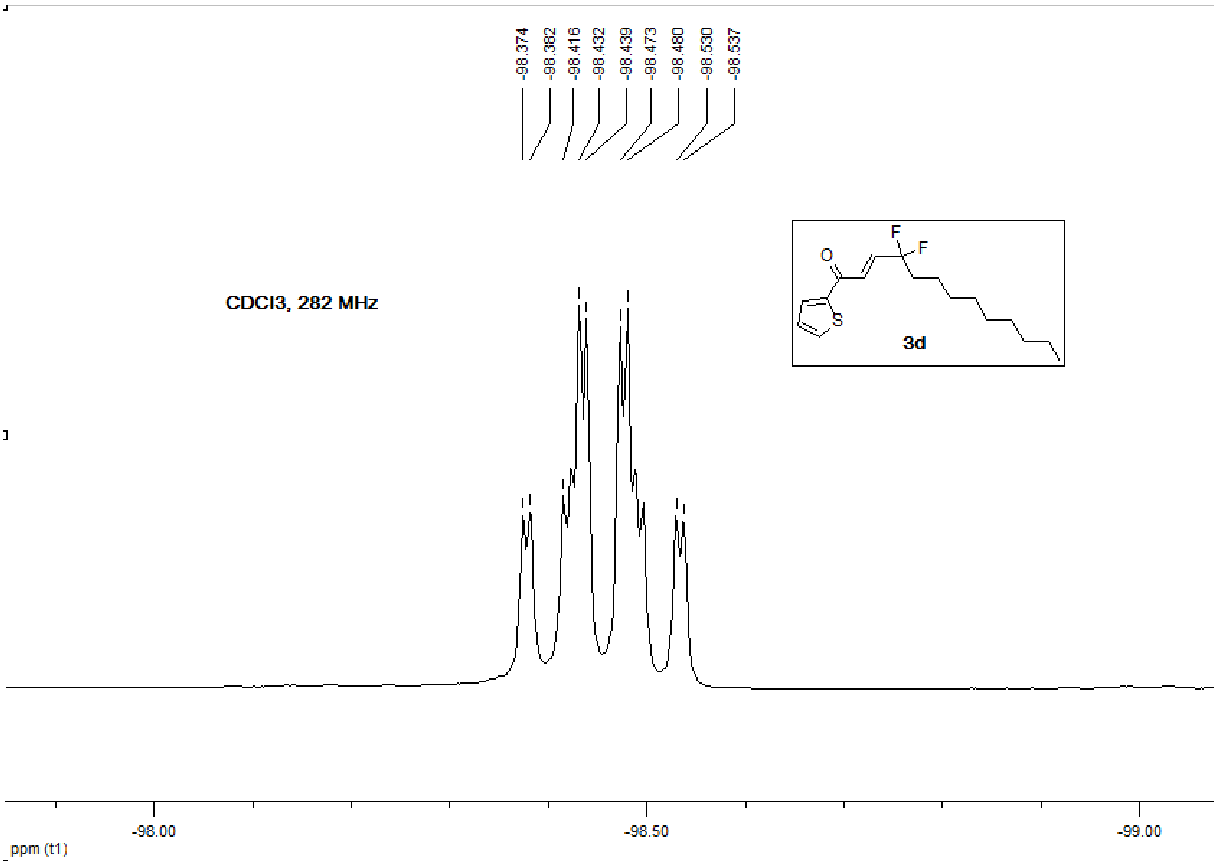
-98.374
-98.382
-98.416
-98.432
-98.439
-98.473
-98.480
-98.530
-98.537
CDCl3, 282 MHz
O
F
F
S
3d
-98.00
-98.50
-99.00
ppm (t1)

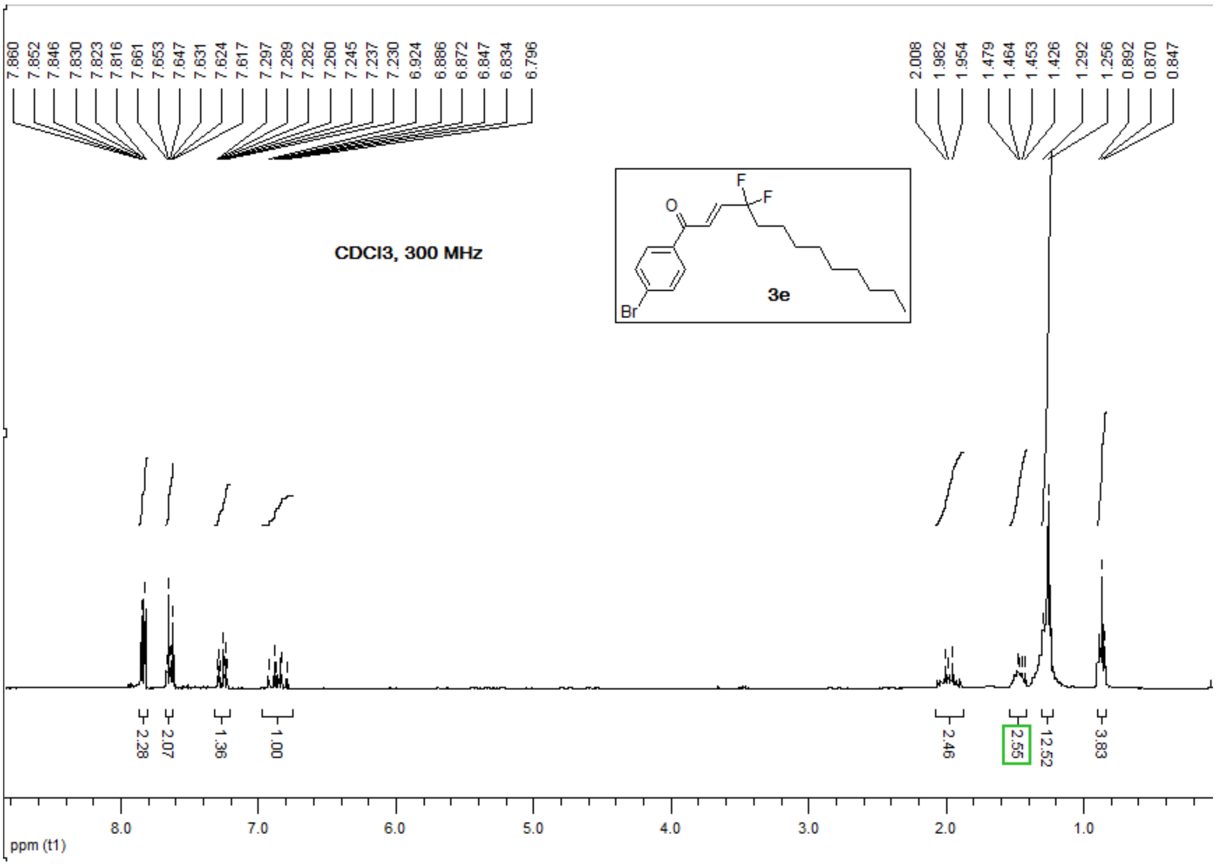
7.860
7.852
7.846
7.830
7.823
7.816
7.661
7.653
7.647
7.631
7.624
7.617
7.297
7.289
7.282
7.260
7.245
7.237
7.230
6.924
6.886
6.872
6.847
6.834
6.796
2.008
1.982
1.954
1.479
1.464
1.453
1.426
1.292
1.256
0.892
0.870
0.847
CDCl3, 300 MHz
O
F
F
Br
3e
2.28
2.07
1.36
1.00
2.46
2.55
12.52
3.83
8.0
7.0
6.0
5.0
4.0
3.0
2.0
1.0
ppm (t1)

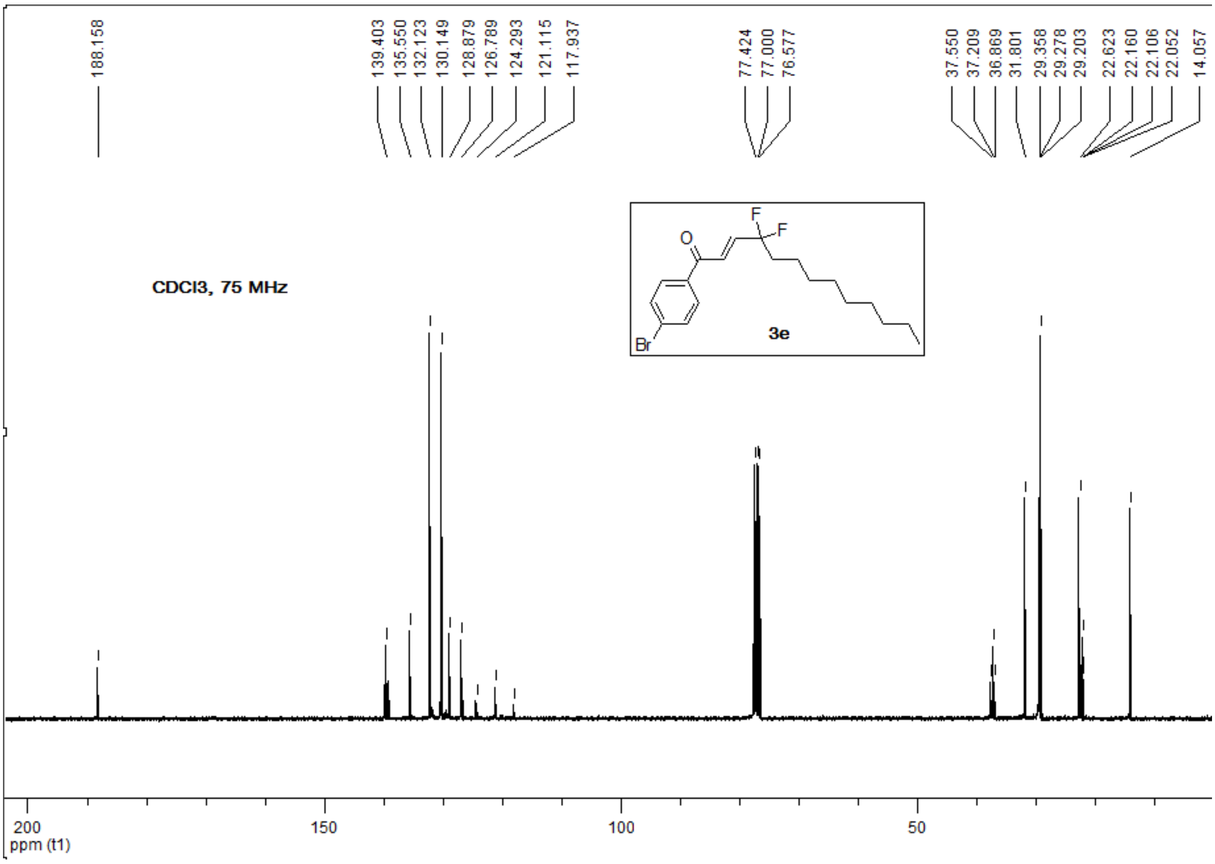
188.158
139.403
135.550
132.123
130.149
128.879
126.789
124.293
121.115
117.937
77.424
77.000
76.577
37.550
37.209
36.869
31.801
29.358
29.278
29.203
22.623
22.160
22.106
22.052
14.057
CDCl3, 75 MHz
O
F
F
Br
3e
200
150
100
50
ppm (t1)

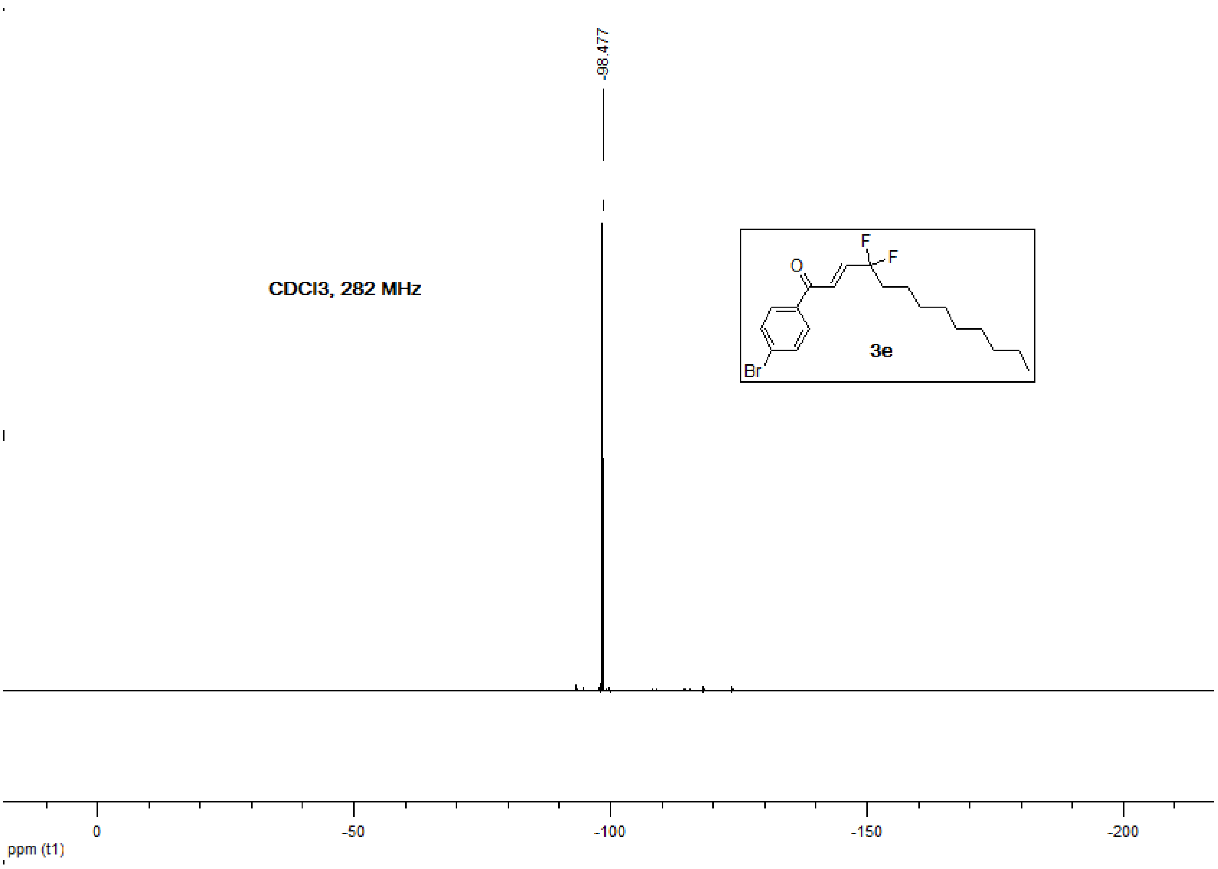

-98.477
CDCl3, 282 MHz
3e
ppm (t1)

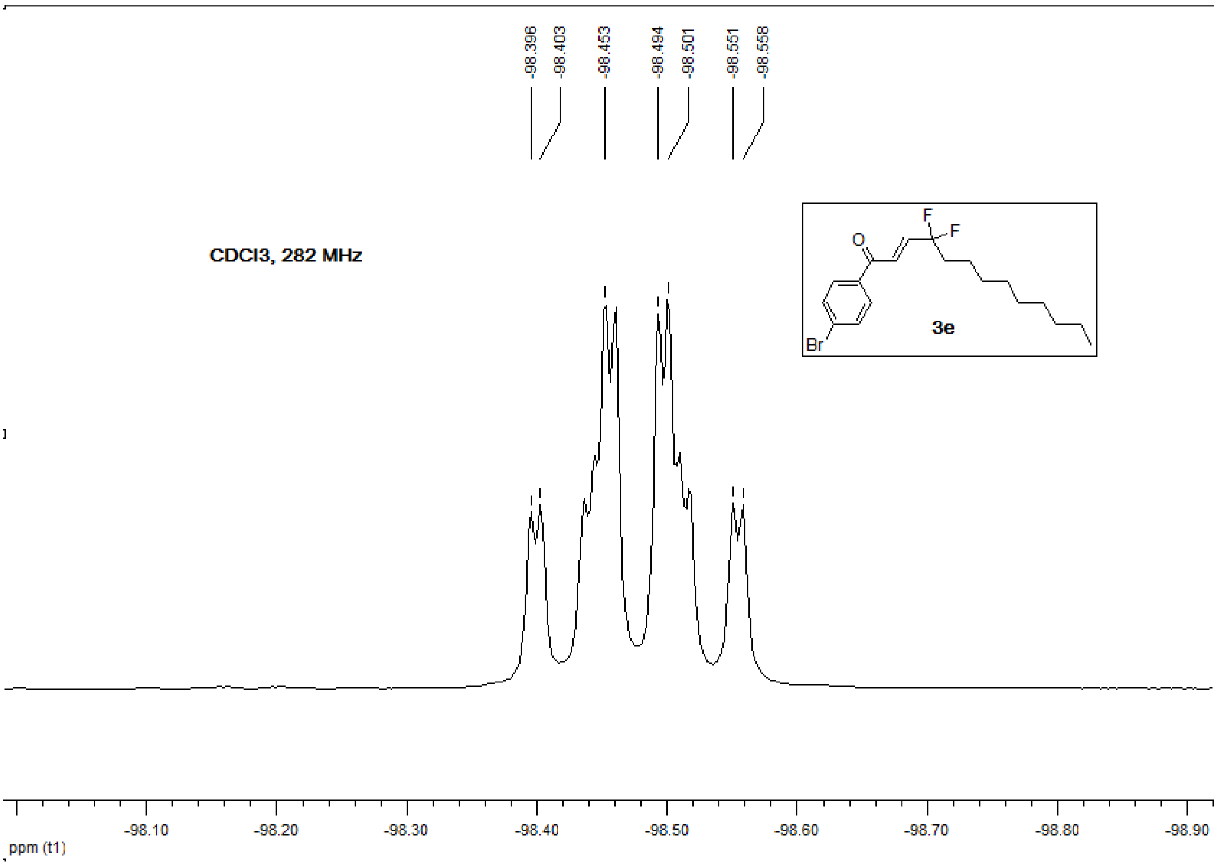

-98.396
-98.403
-98.453
-98.494
-98.501
-98.551
-98.558
CDCl3, 282 MHz
3e
ppm (t1)

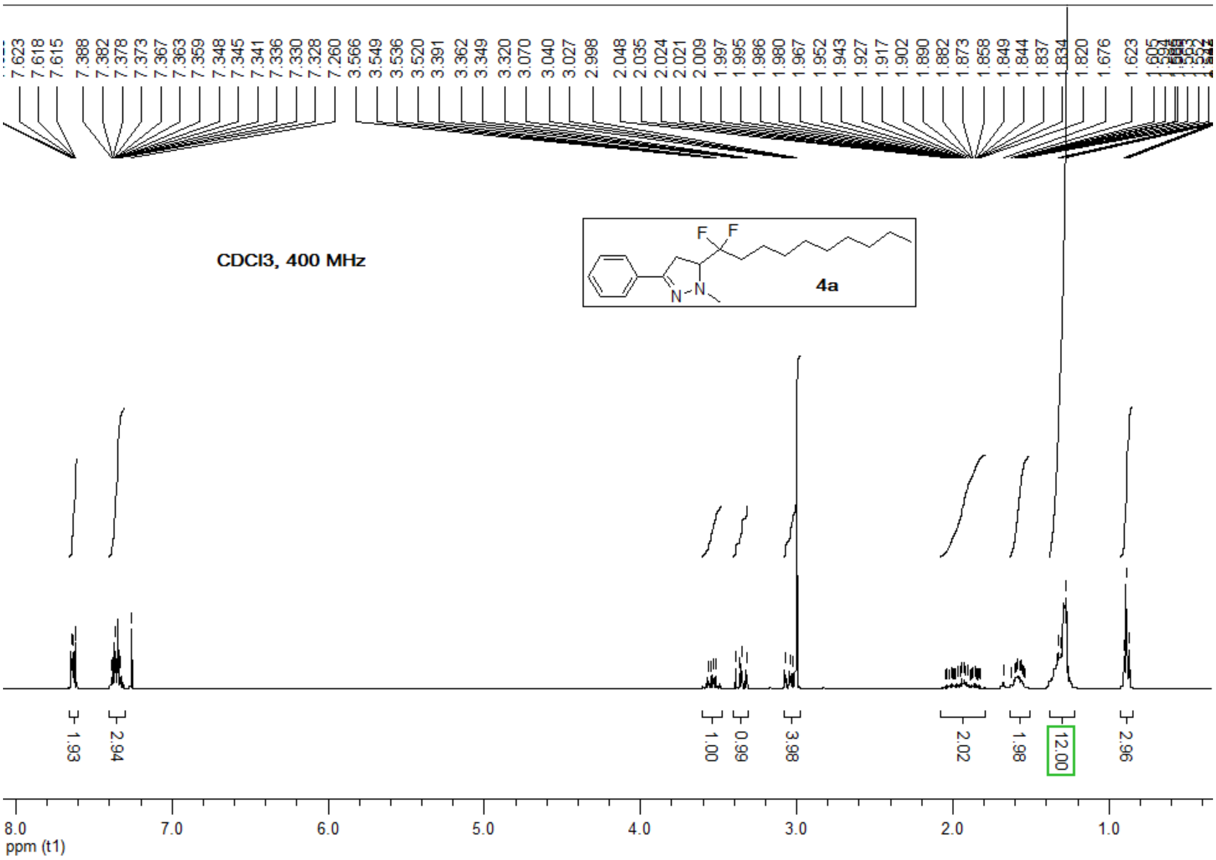
7.623
7.618
7.615
7.388
7.382
7.378
7.373
7.367
7.363
7.359
7.348
7.345
7.341
7.336
7.330
7.328
7.260
3.566
3.549
3.536
3.520
3.391
3.362
3.349
3.320
3.070
3.040
3.027
2.998
2.048
2.035
2.024
2.021
2.009
1.997
1.995
1.986
1.980
1.967
1.952
1.943
1.927
1.917
1.902
1.890
1.882
1.873
1.858
1.849
1.844
1.837
1.834
1.820
1.676
1.623
1.605
CDCl3, 400 MHz
F
F
N
N
4a
1.93
2.94
1.00
0.99
3.98
2.02
1.98
12.00
2.96
8.0
7.0
6.0
5.0
4.0
3.0
2.0
1.0
ppm (t1)

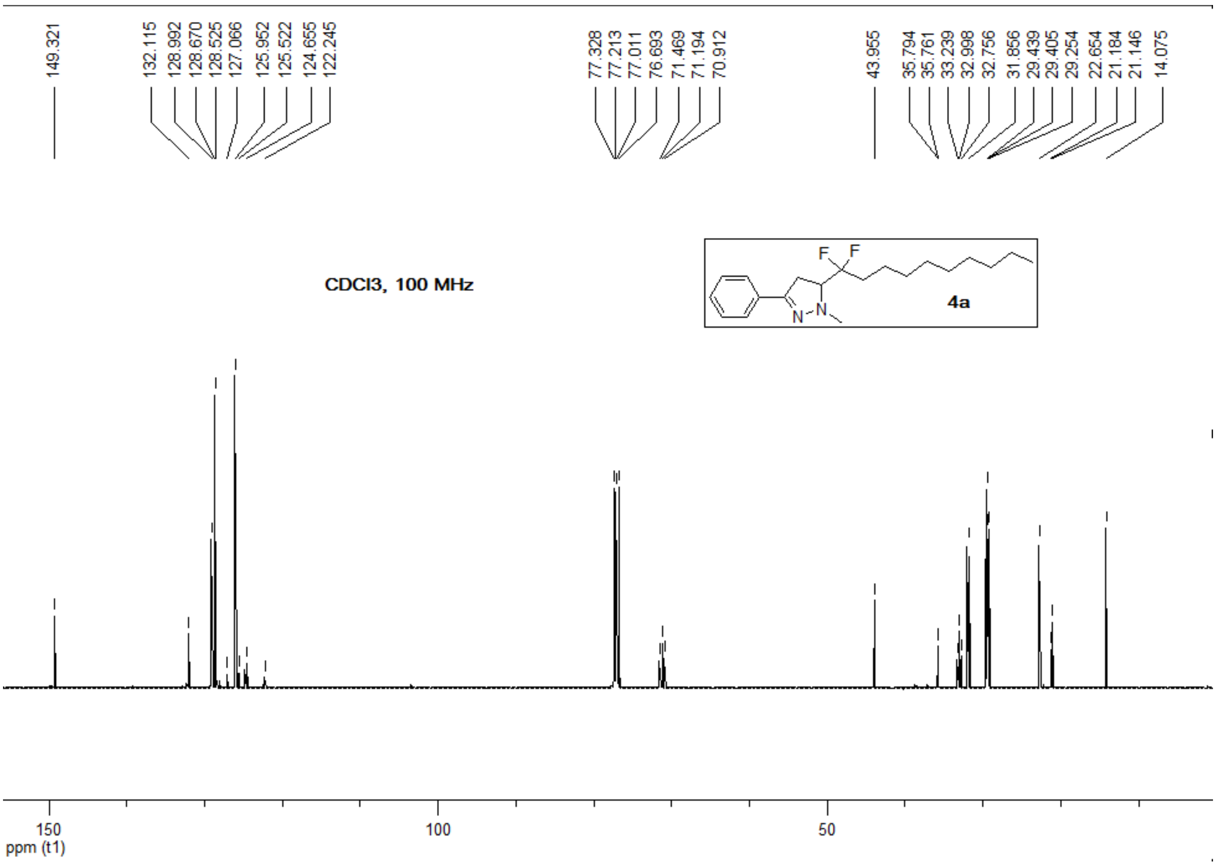
149.321
132.115
128.992
128.670
128.525
127.066
125.952
125.522
124.655
122.245
77.328
77.213
77.011
76.693
71.469
71.194
70.912
43.955
35.794
35.761
33.239
32.998
32.756
31.856
29.439
29.405
29.254
22.654
21.184
21.146
14.075
CDCl3, 100 MHz
F
F
N
N
4a
150
100
50
ppm (t1)

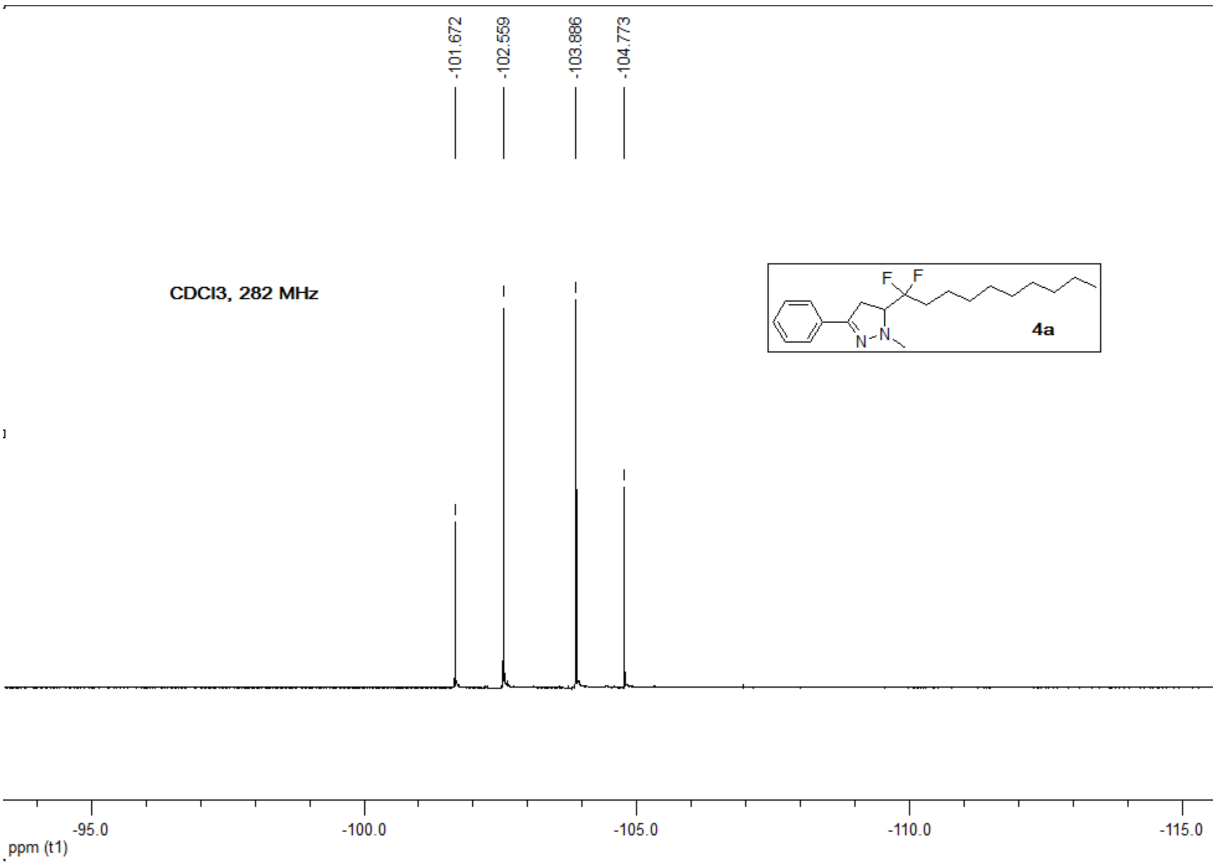
-101.672
-102.559
-103.886
-104.773
CDCl3, 282 MHz
4a
-95.0
-100.0
-105.0
-110.0
-115.0
ppm (t1)

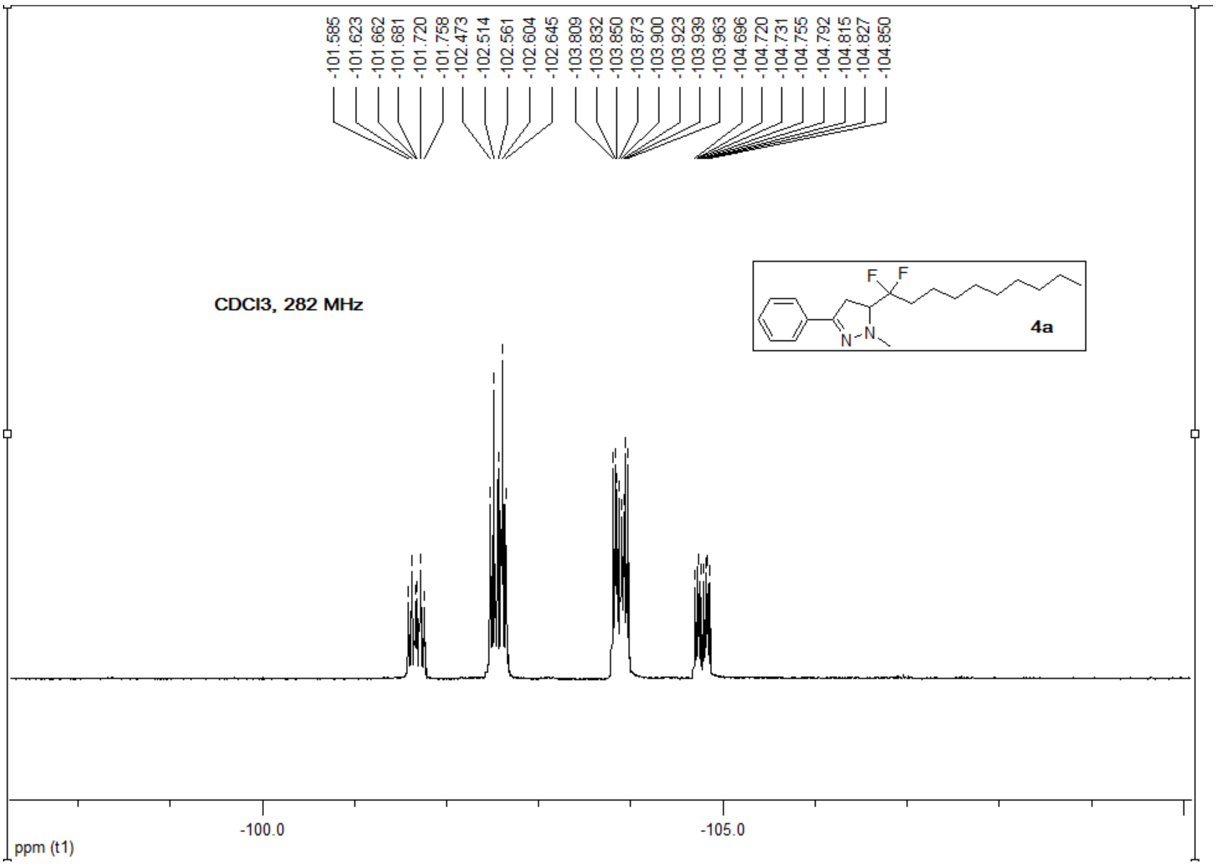
-101.585
-101.623
-101.662
-101.681
-101.720
-101.758
-102.473
-102.514
-102.561
-102.604
-102.645
-103.809
-103.832
-103.850
-103.873
-103.900
-103.923
-103.939
-103.963
-104.696
-104.720
-104.731
-104.755
-104.792
-104.815
-104.827
-104.850
CDCl3, 282 MHz
4a
-100.0
-105.0
ppm (t1)

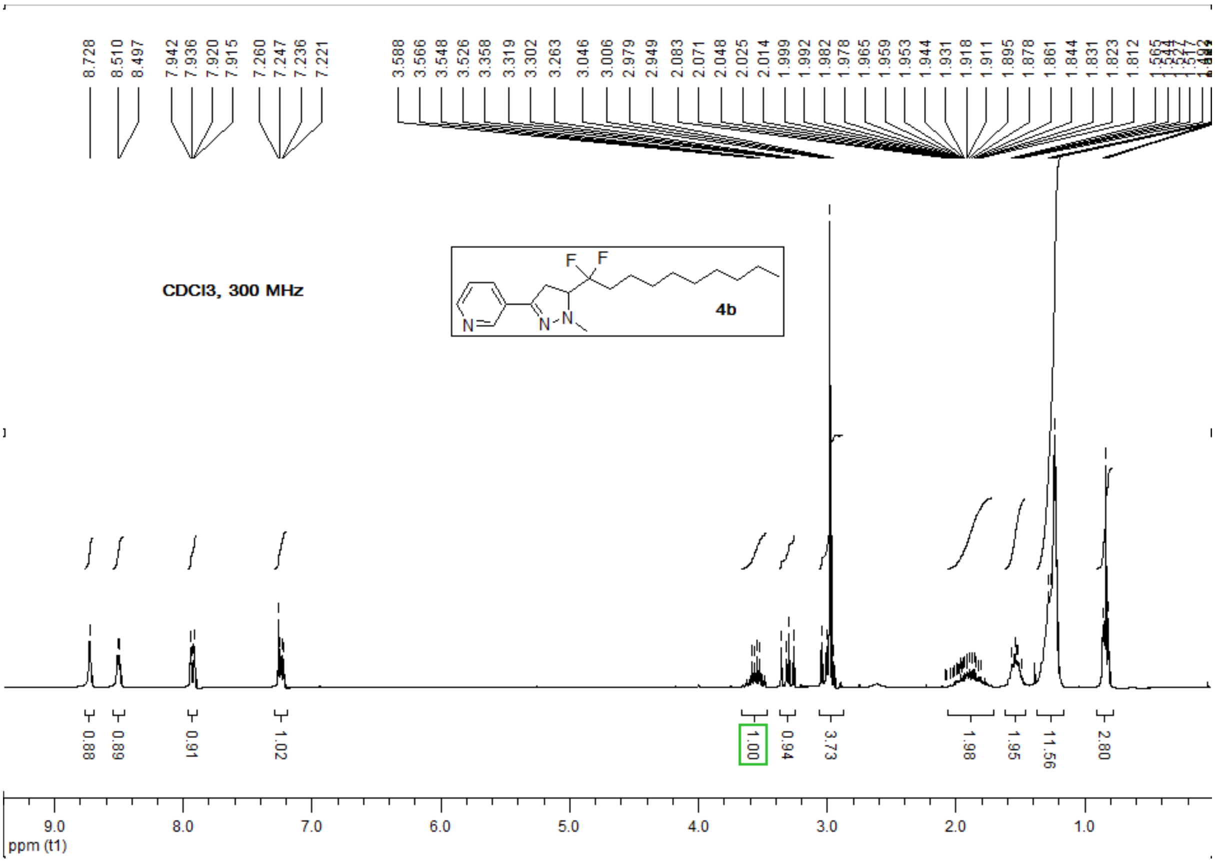
8.728
8.510
8.497
7.942
7.936
7.920
7.915
7.260
7.247
7.236
7.221
3.588
3.566
3.548
3.526
3.358
3.319
3.302
3.263
3.046
3.006
2.979
2.949
2.083
2.071
2.048
2.025
2.014
1.999
1.992
1.982
1.978
1.965
1.959
1.953
1.944
1.931
1.918
1.911
1.895
1.878
1.861
1.844
1.831
1.823
1.812
CDCl3, 300 MHz
4b
0.88
0.89
0.91
1.02
1.00
0.94
3.73
1.98
1.95
11.56
2.80
9.0
8.0
7.0
6.0
5.0
4.0
3.0
2.0
1.0
ppm (t1)

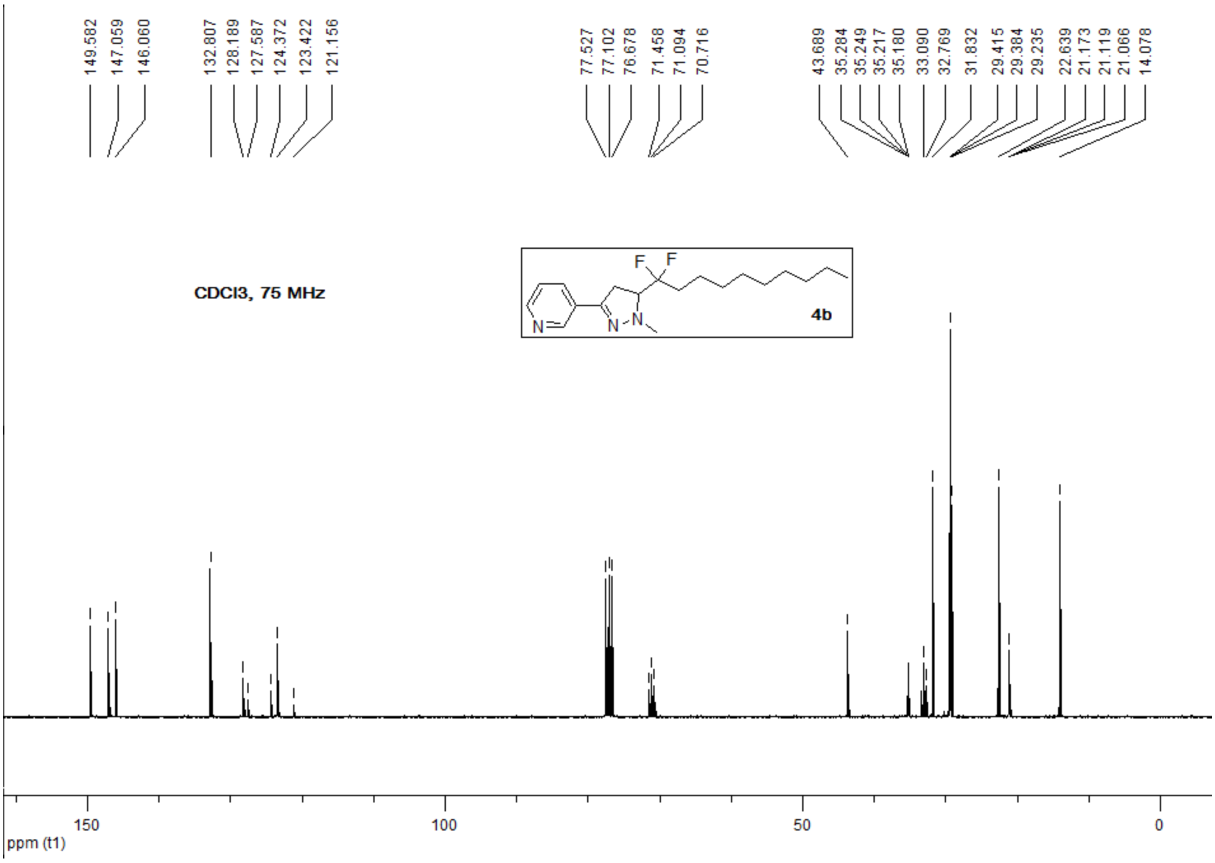
149.582
147.059
146.060
132.807
128.189
127.587
124.372
123.422
121.156
77.527
77.102
76.678
71.458
71.094
70.716
43.689
35.284
35.249
35.217
35.180
33.090
32.769
31.832
29.415
29.384
29.235
22.639
21.173
21.119
21.066
14.078
CDCl3, 75 MHz
4b
150
100
50
0
ppm (t1)

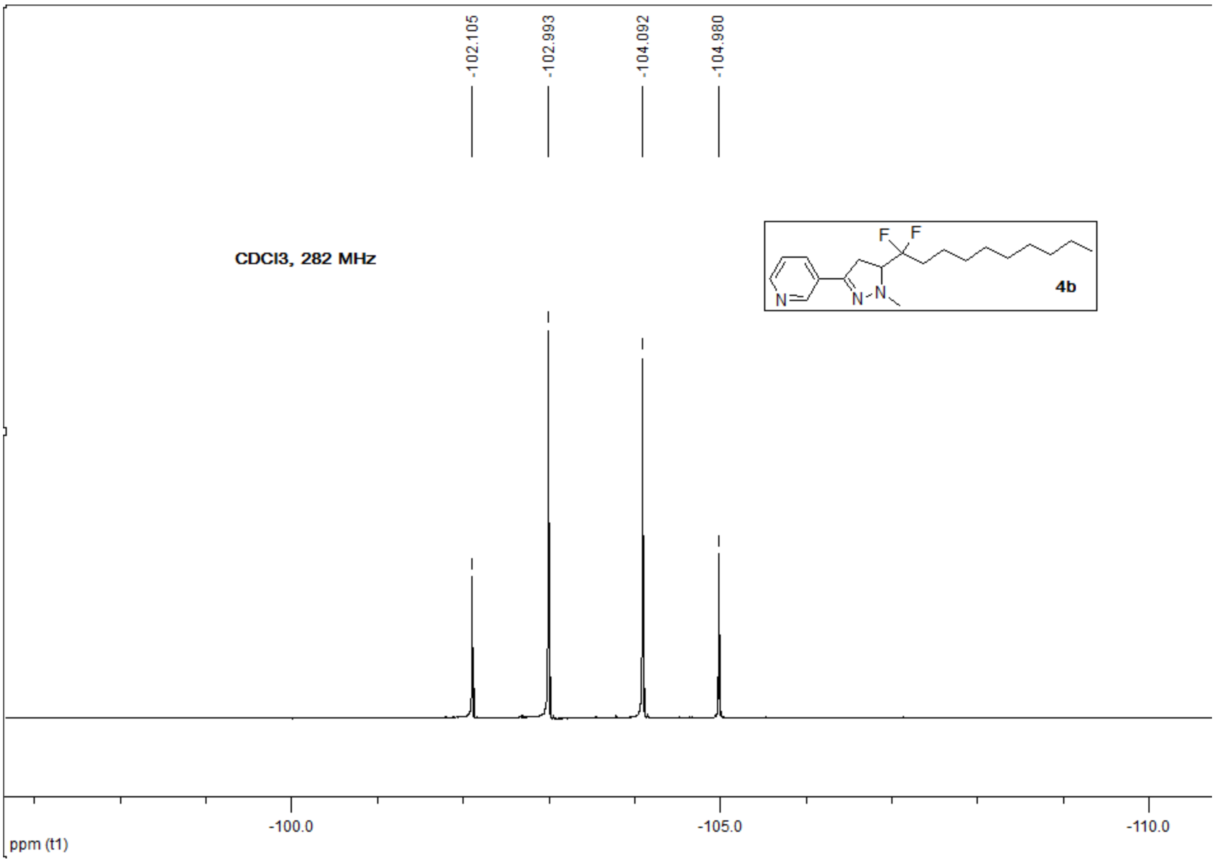

-102.105
-102.993
-104.092
-104.980
CDCl3, 282 MHz
4b
-100.0
-105.0
-110.0
ppm (t1)

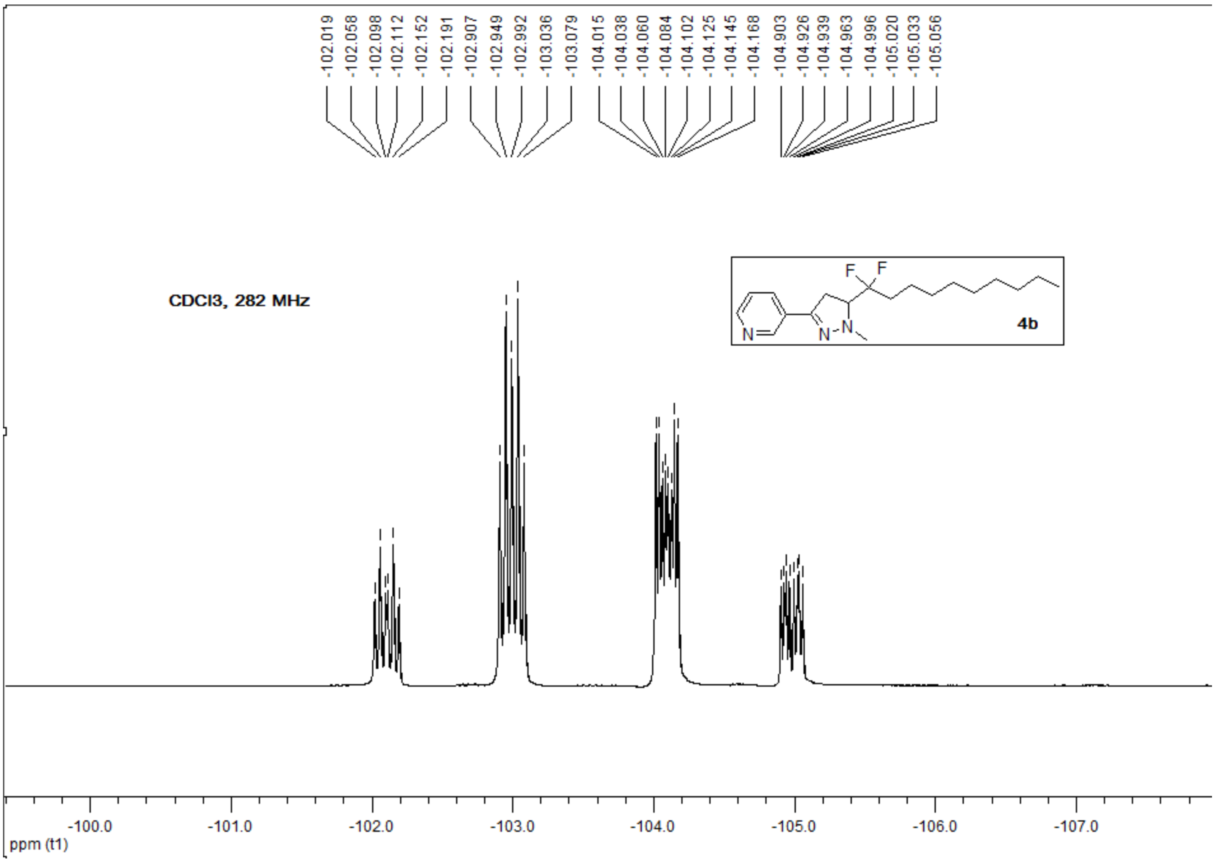

-102.019
-102.058
-102.098
-102.112
-102.152
-102.191
-102.907
-102.949
-102.992
-103.036
-103.079
-104.015
-104.038
-104.060
-104.084
-104.102
-104.125
-104.145
-104.168
-104.903
-104.926
-104.939
-104.963
-104.996
-105.020
-105.033
-105.056
CDCl3, 282 MHz
4b
-100.0
-101.0
-102.0
-103.0
-104.0
-105.0
-106.0
-107.0
ppm (t1)

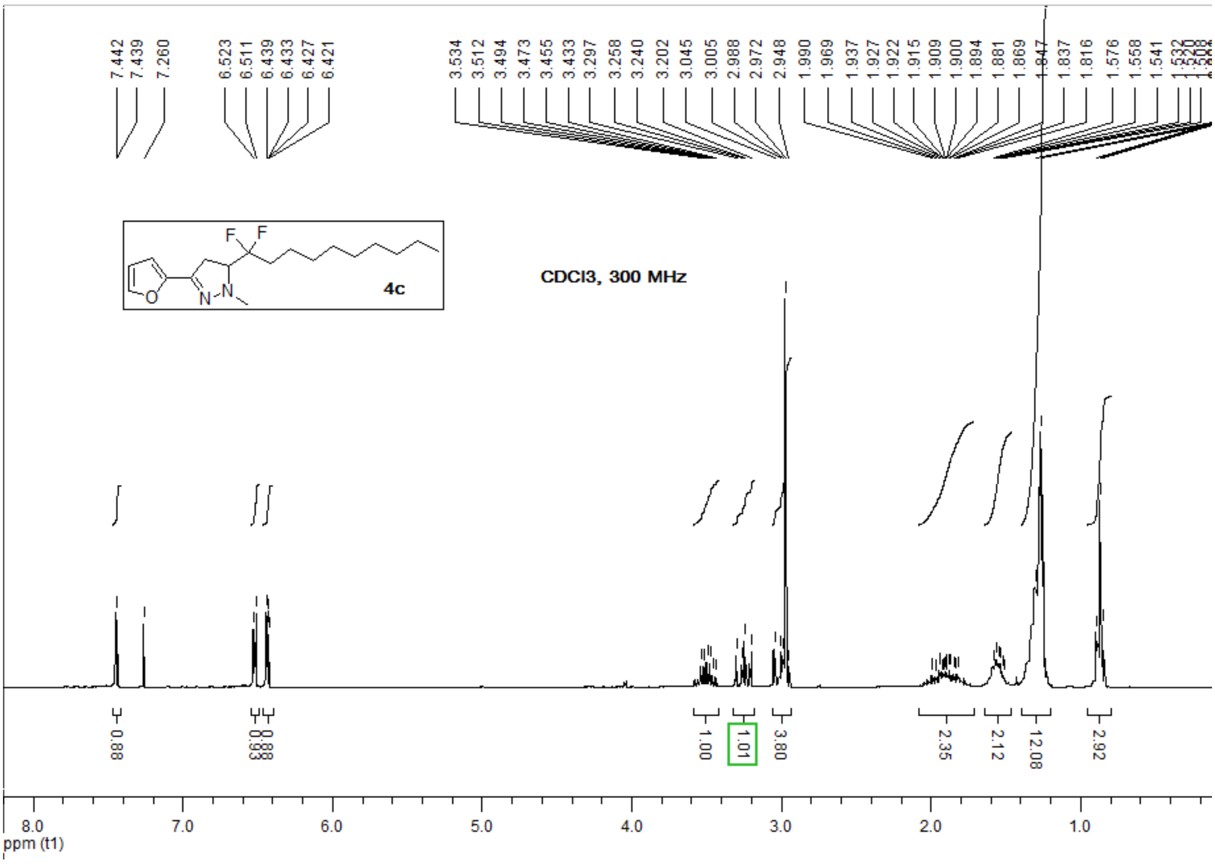

7.442
7.439
7.260
6.523
6.511
6.439
6.433
6.427
6.421
3.534
3.512
3.494
3.473
3.455
3.433
3.297
3.258
3.240
3.202
3.045
3.005
2.988
2.972
2.948
1.990
1.969
1.937
1.927
1.922
1.915
1.909
1.900
1.894
1.881
1.869
1.847
1.837
1.816
1.576
1.558
1.541
1.532
CDCl3, 300 MHz
4c
0.88
0.93
0.88
1.00
1.01
3.80
2.35
2.12
12.08
2.92
8.0
7.0
6.0
5.0
4.0
3.0
2.0
1.0
ppm (t1)

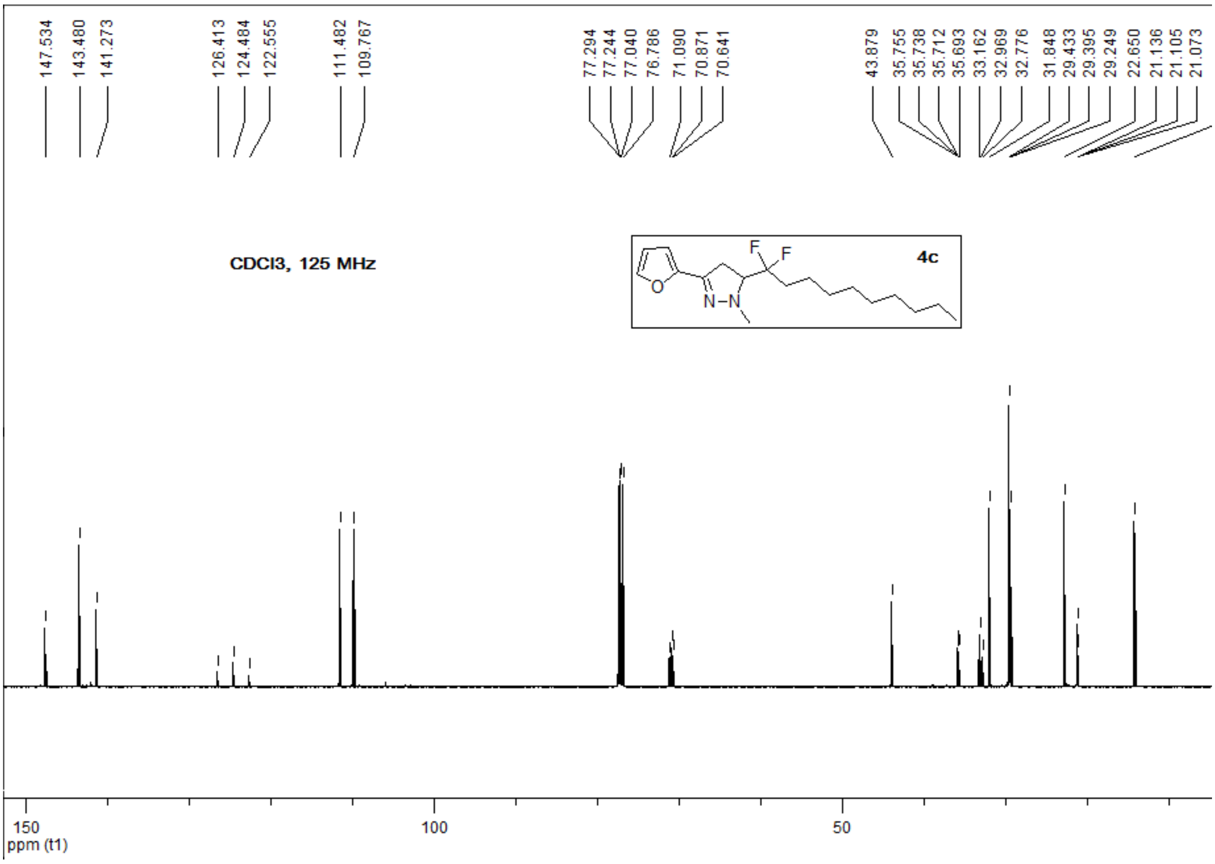

147.534
143.480
141.273
126.413
124.484
122.555
111.482
109.767
77.294
77.244
77.040
76.786
71.090
70.871
70.641
43.879
35.755
35.738
35.712
35.693
33.162
32.969
32.776
31.848
29.433
29.395
29.249
22.650
21.136
21.105
21.073
CDCl3, 125 MHz
4c
150
100
50
ppm (t1)

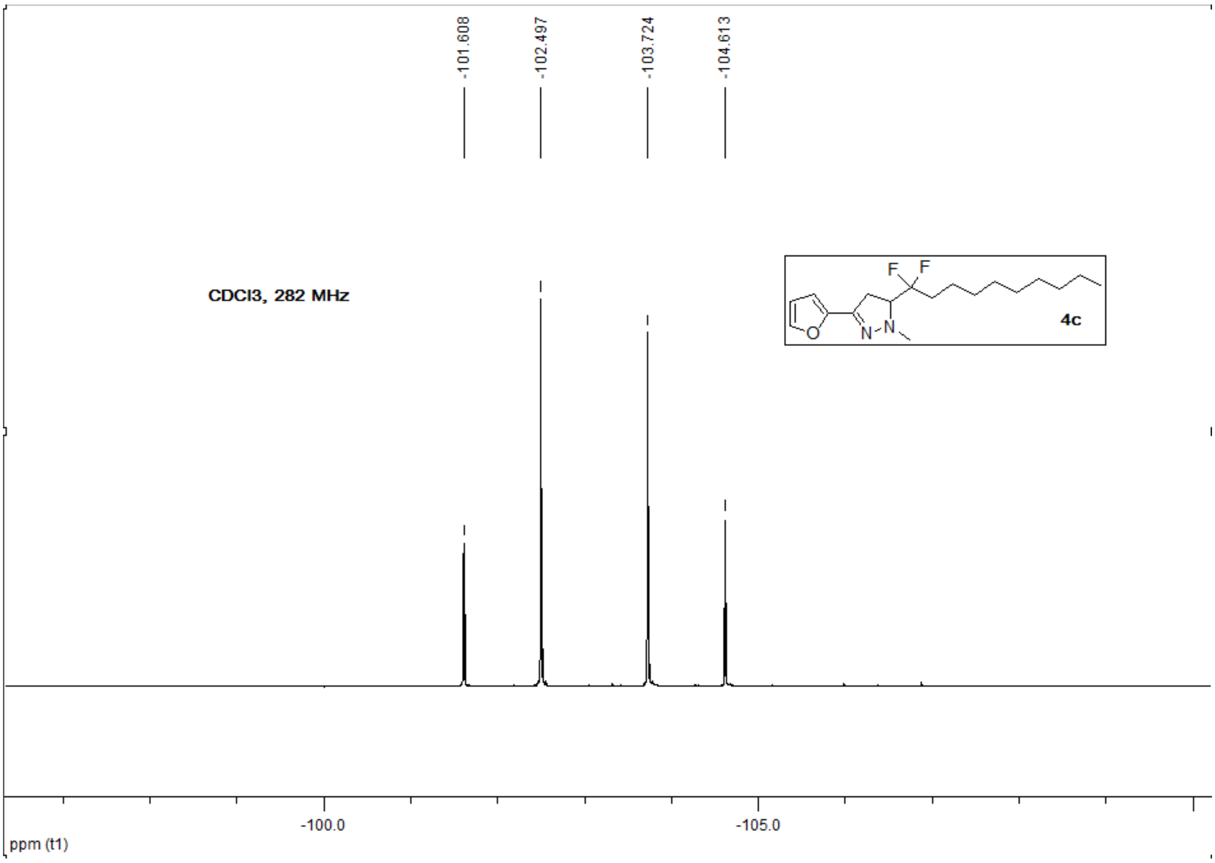
-101.608
-102.497
-103.724
-104.613
CDCl3, 282 MHz
F
F
O
N
N
4c
-100.0
-105.0
ppm (t1)

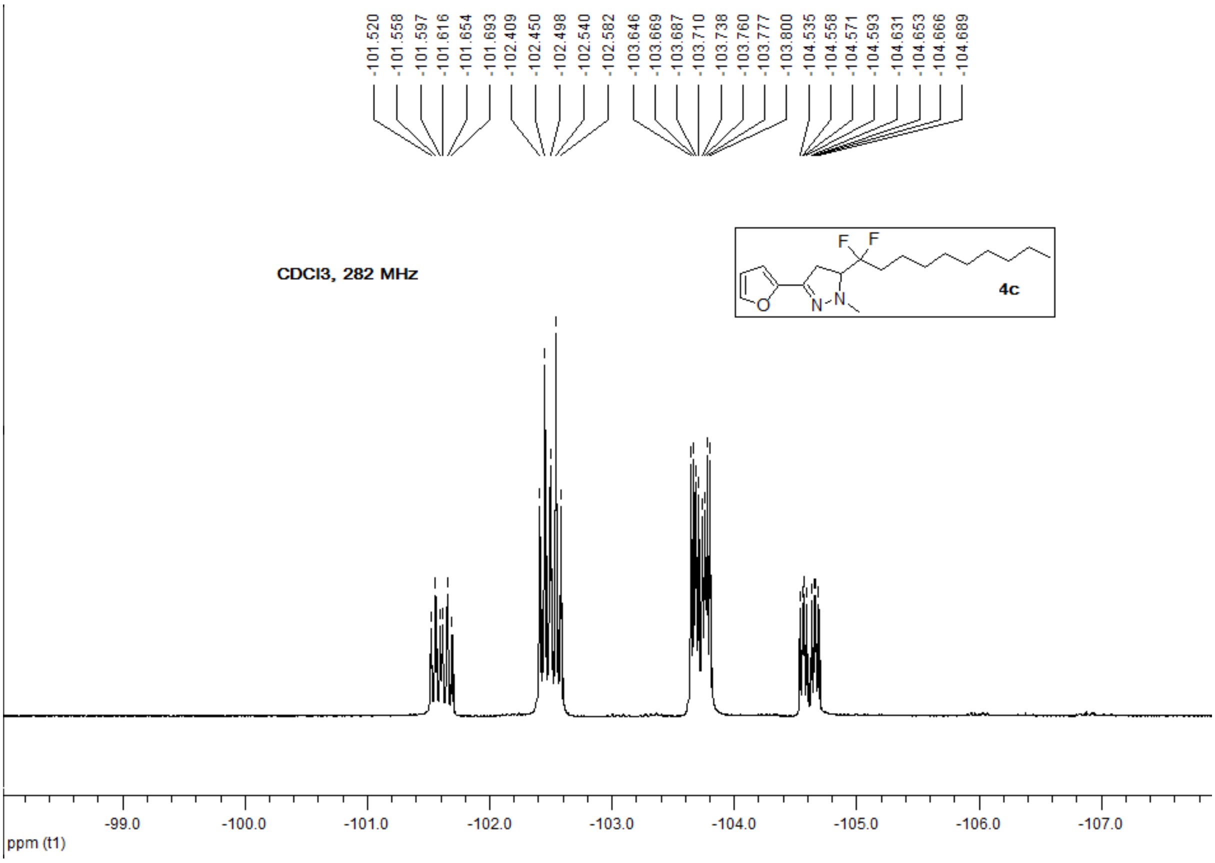
-101.520
-101.558
-101.597
-101.616
-101.654
-101.693
-102.409
-102.450
-102.498
-102.540
-102.582
-103.646
-103.669
-103.687
-103.710
-103.738
-103.760
-103.777
-103.800
-104.535
-104.558
-104.571
-104.593
-104.631
-104.653
-104.666
-104.689
CDCl3, 282 MHz
F
F
O
N
N
4c
-99.0
-100.0
-101.0
-102.0
-103.0
-104.0
-105.0
-106.0
-107.0
ppm (t1)

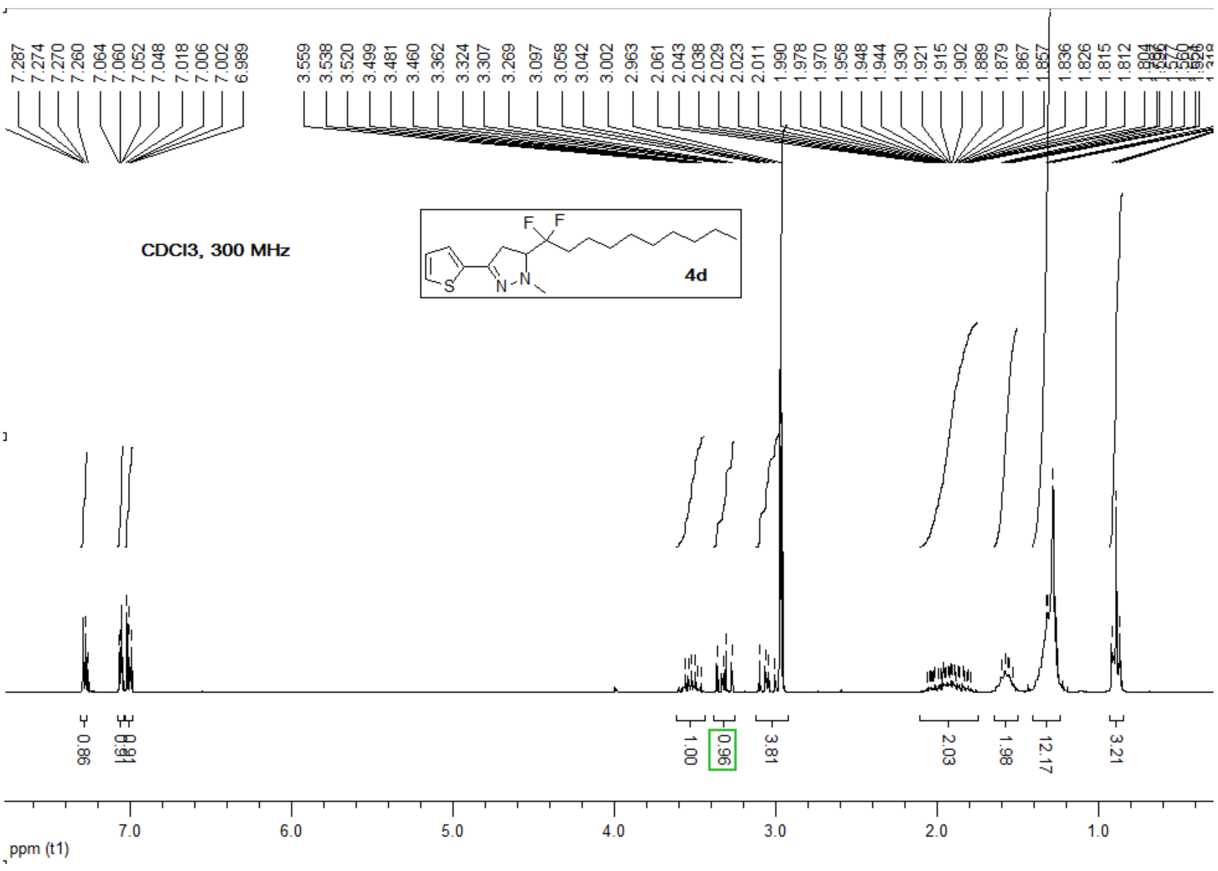
CDCl3, 300 MHz
4d
ppm (t1)

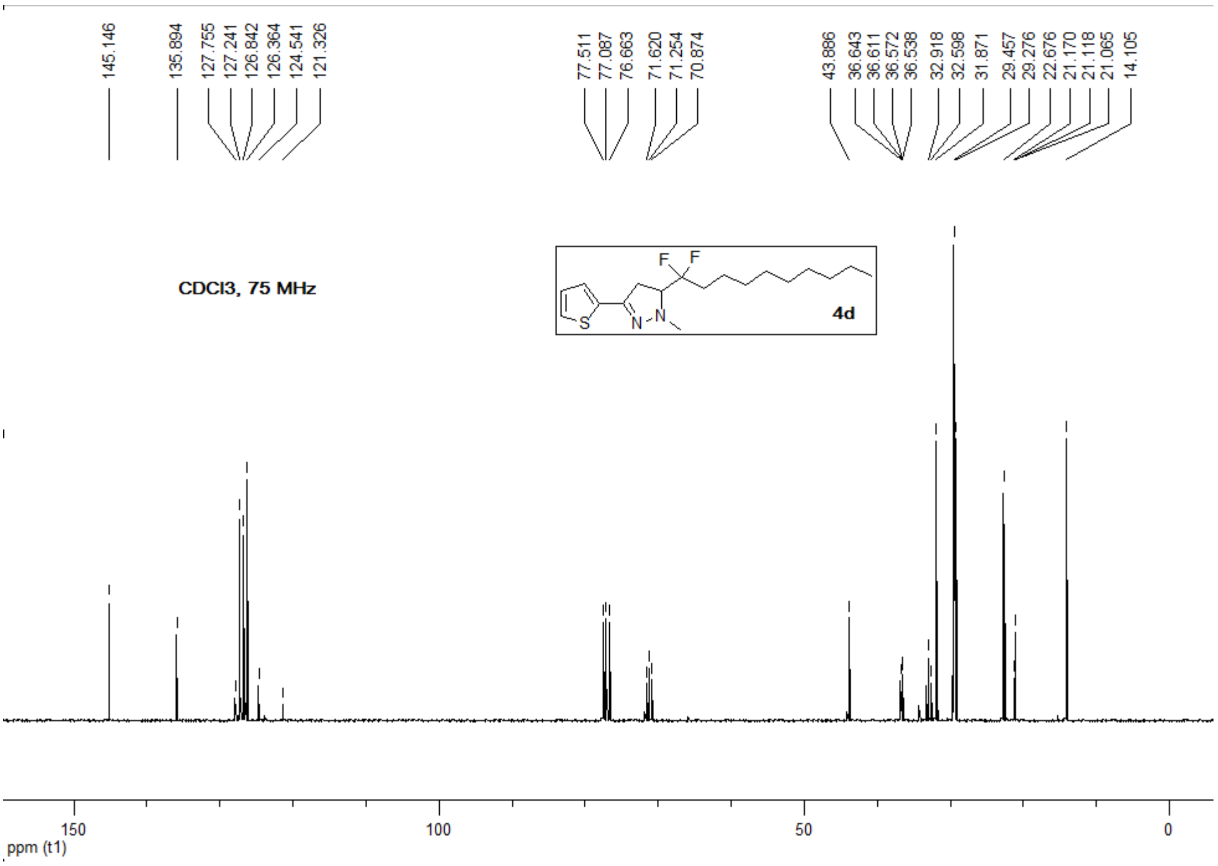
CDCl3, 75 MHz
4d
ppm (t1)

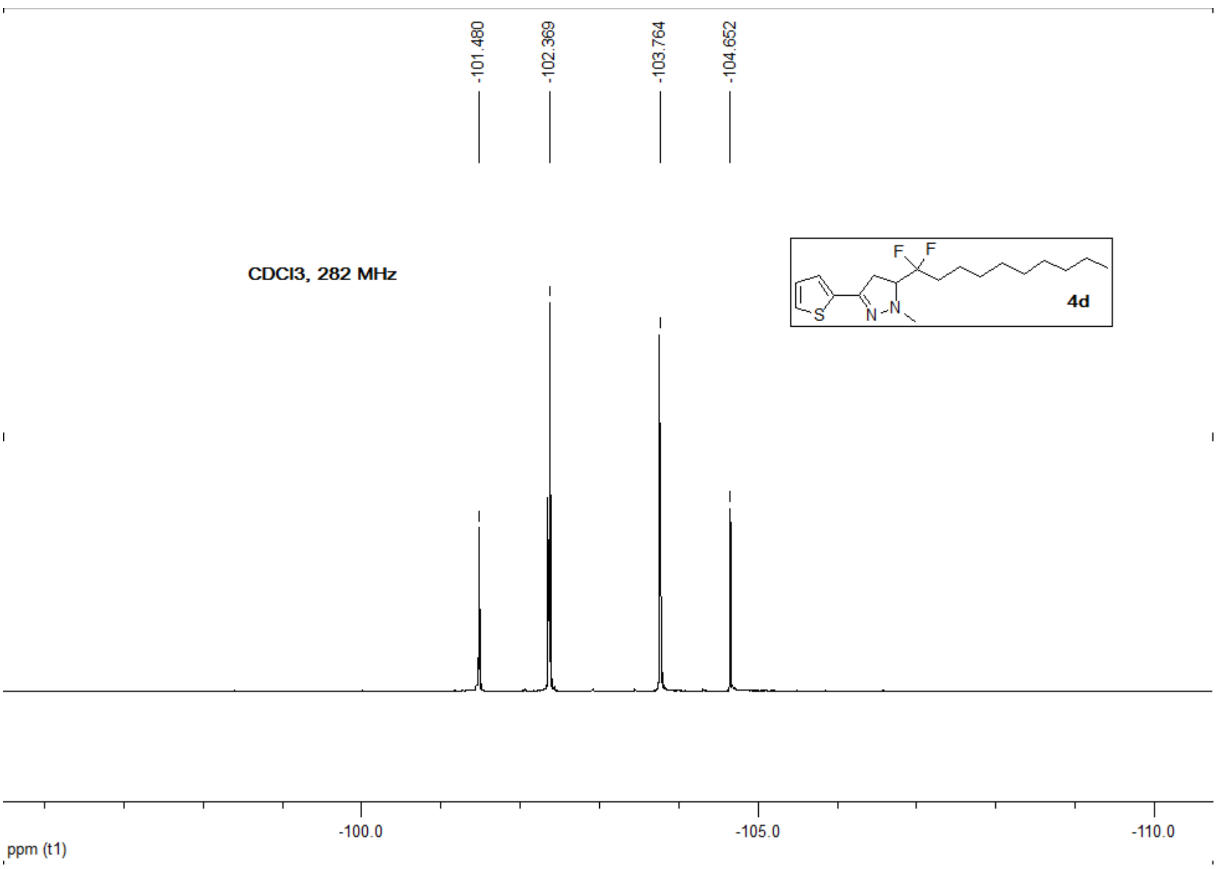
-101.480
-102.369
-103.764
-104.652
CDCl3, 282 MHz
F F
S
N
N
4d
-100.0
-105.0
-110.0
ppm (t1)

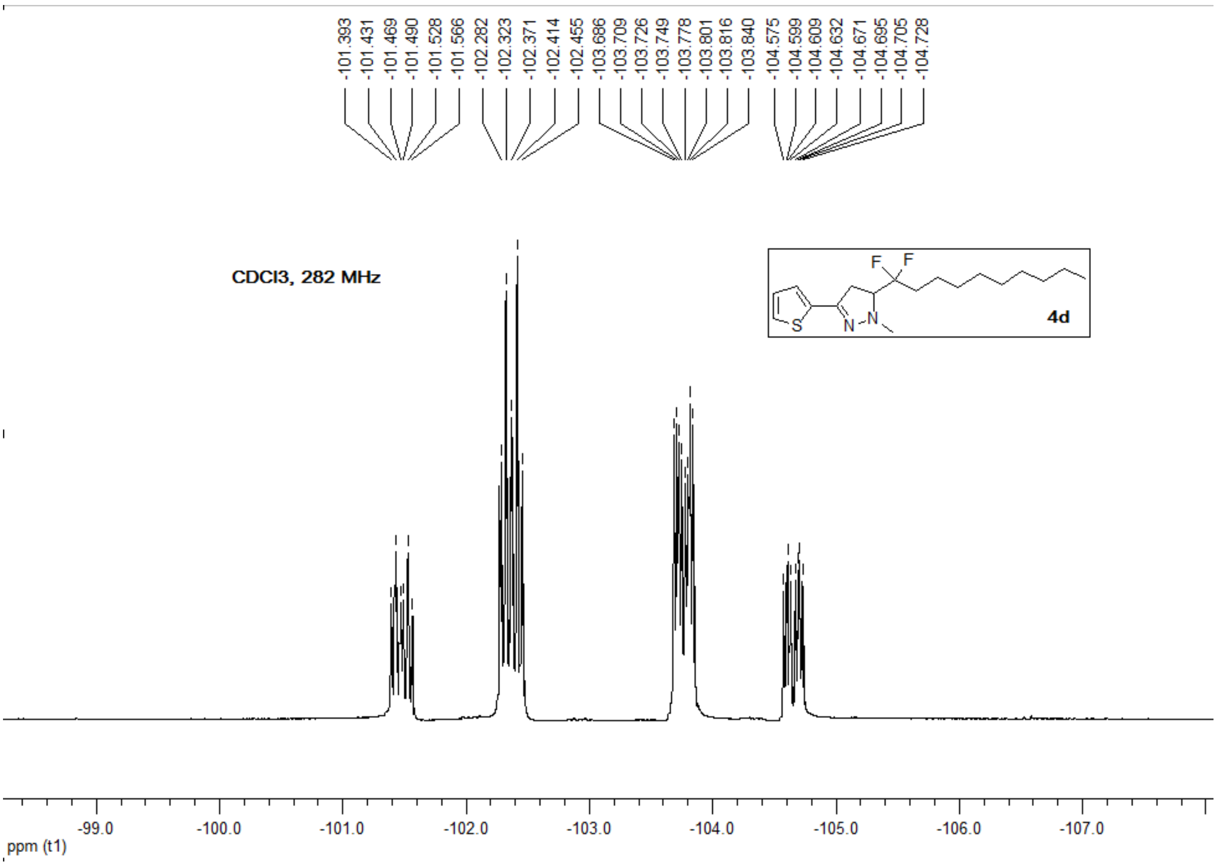
-101.393
-101.431
-101.469
-101.490
-101.528
-101.566
-102.282
-102.323
-102.371
-102.414
-102.455
-103.686
-103.709
-103.726
-103.749
-103.778
-103.801
-103.816
-103.840
-104.575
-104.599
-104.609
-104.632
-104.671
-104.695
-104.705
-104.728
CDCl3, 282 MHz
F F
S
N
N
4d
-99.0
-100.0
-101.0
-102.0
-103.0
-104.0
-105.0
-106.0
-107.0
ppm (t1)

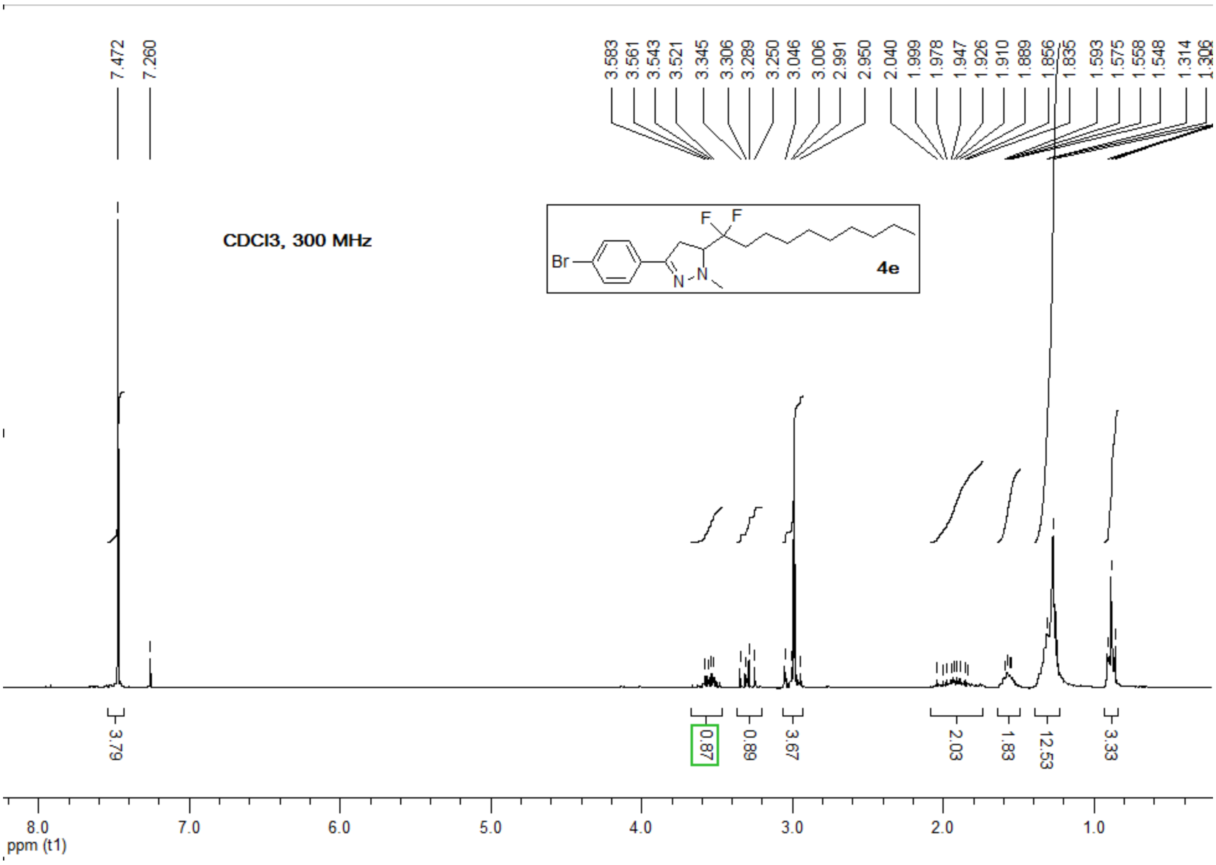

7.472
7.260
3.583 3.561 3.543 3.521 3.345 3.306 3.289 3.250 3.046 3.006 2.991 2.950 2.040 1.999 1.978 1.947 1.926 1.910 1.889 1.856 1.835 1.593 1.575 1.558 1.548 1.314 1.306
CDCl3, 300 MHz
F F
Br
N N
4e
3.79
0.87
0.89
3.67
2.03
1.83
12.53
3.33
8.0 7.0 6.0 5.0 4.0 3.0 2.0 1.0
ppm (t1)

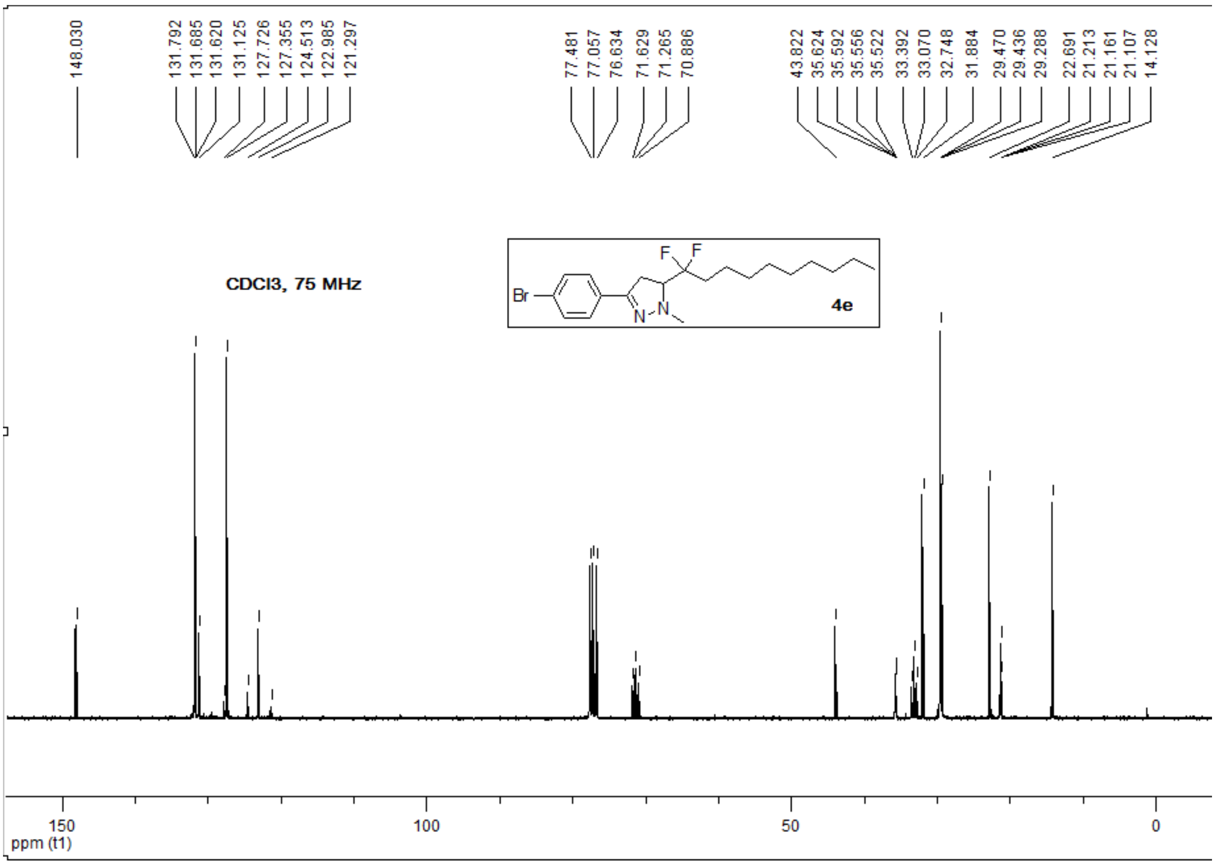

148.030
131.792 131.685 131.620 131.125 127.726 127.355 124.513 122.985 121.297
77.481 77.057 76.634 71.629 71.265 70.886
43.822 35.624 35.592 35.556 35.522 33.392 33.070 32.748 31.884 29.470 29.436 29.288 22.691 21.213 21.161 21.107 14.128
CDCl3, 75 MHz
F F
Br
N N
4e
150 100 50 0
ppm (t1)

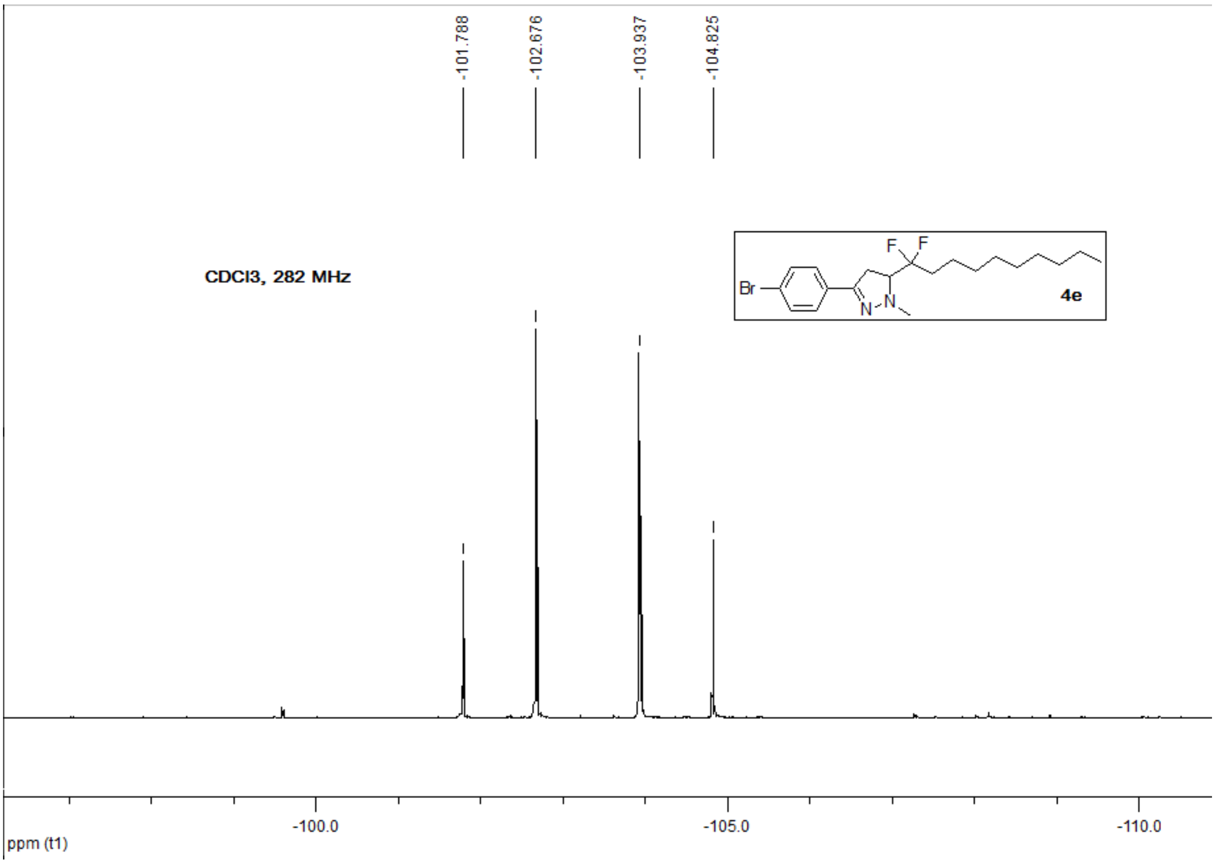
-101.788
-102.676
-103.937
-104.825
CDCl3, 282 MHz
Br
F F
N N
4e
-100.0
-105.0
-110.0
ppm (t1)

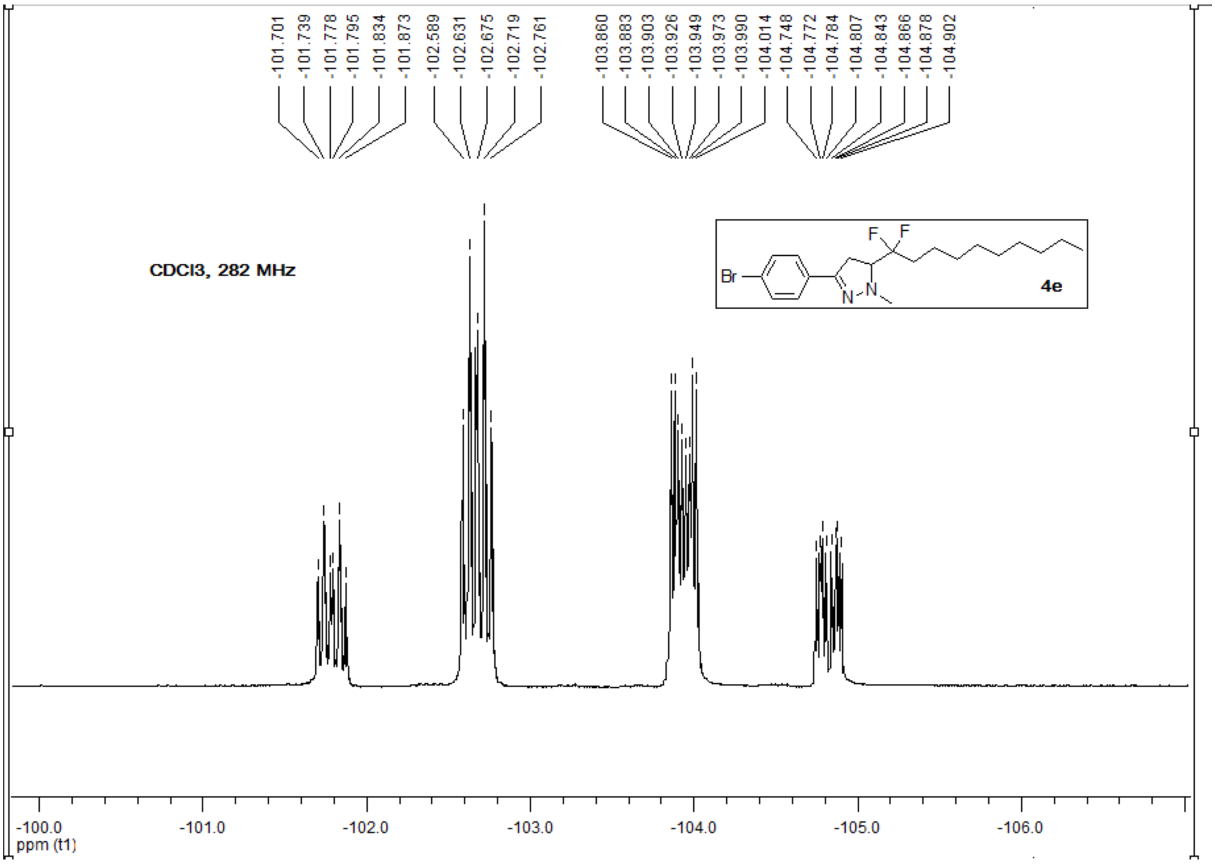
-101.701
-101.739
-101.778
-101.795
-101.834
-101.873
-102.589
-102.631
-102.675
-102.719
-102.761
-103.860
-103.883
-103.903
-103.926
-103.949
-103.973
-103.990
-104.014
-104.748
-104.772
-104.784
-104.807
-104.843
-104.866
-104.878
-104.902
CDCl3, 282 MHz
Br
F F
N N
4e
-100.0
ppm (t1)
-101.0
-102.0
-103.0
-104.0
-105.0
-106.0

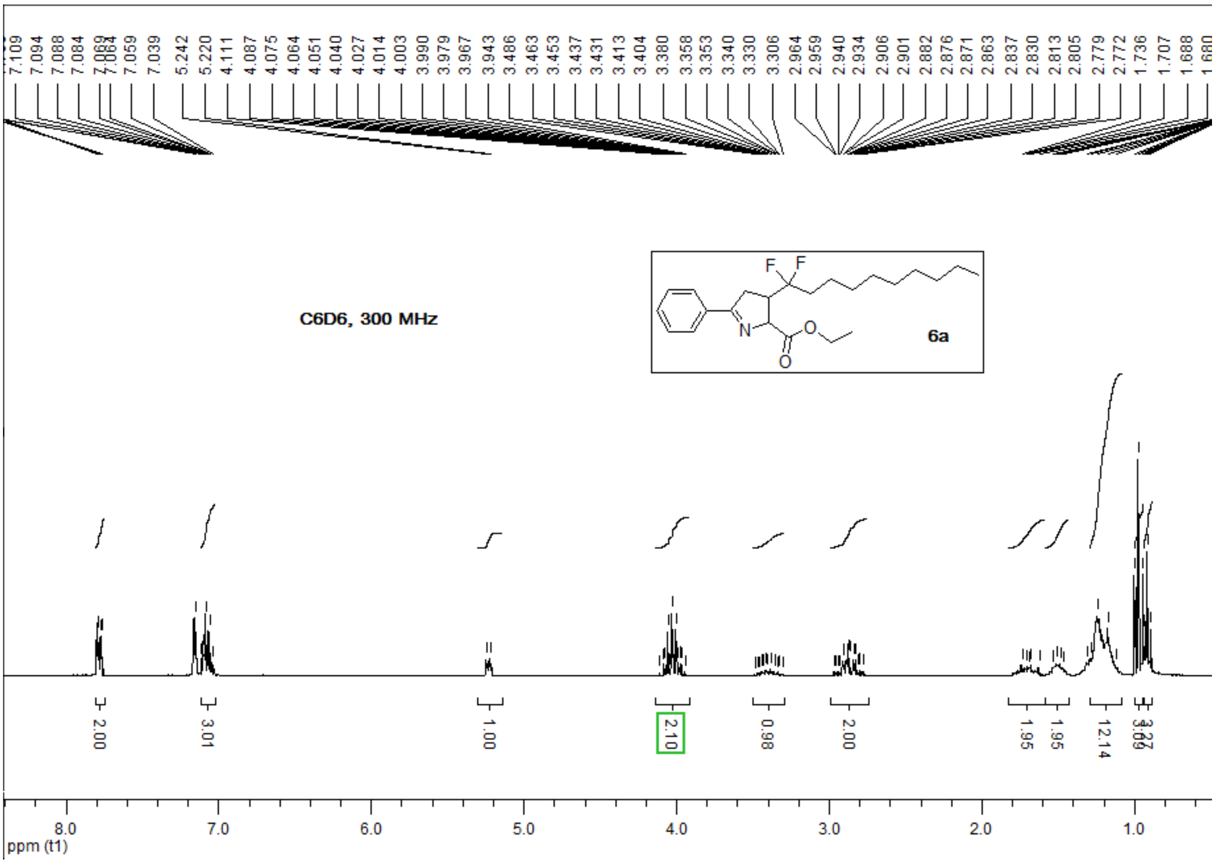
7.109
7.094
7.088
7.084
7.069
7.064
7.059
7.039
5.242
5.220
4.111
4.087
4.075
4.064
4.051
4.040
4.027
4.014
4.003
3.990
3.979
3.967
3.943
3.486
3.463
3.453
3.437
3.431
3.413
3.404
3.380
3.358
3.353
3.340
3.330
3.306
2.964
2.959
2.940
2.934
2.906
2.901
2.882
2.876
2.871
2.863
2.837
2.830
2.813
2.805
2.779
2.772
1.736
1.707
1.688
1.680
C6D6, 300 MHz
F
F
N
O
O
6a
2.00
3.01
1.00
2.10
0.98
2.00
1.95
1.95
12.14
8.0
7.0
6.0
5.0
4.0
3.0
2.0
1.0
ppm (t1)

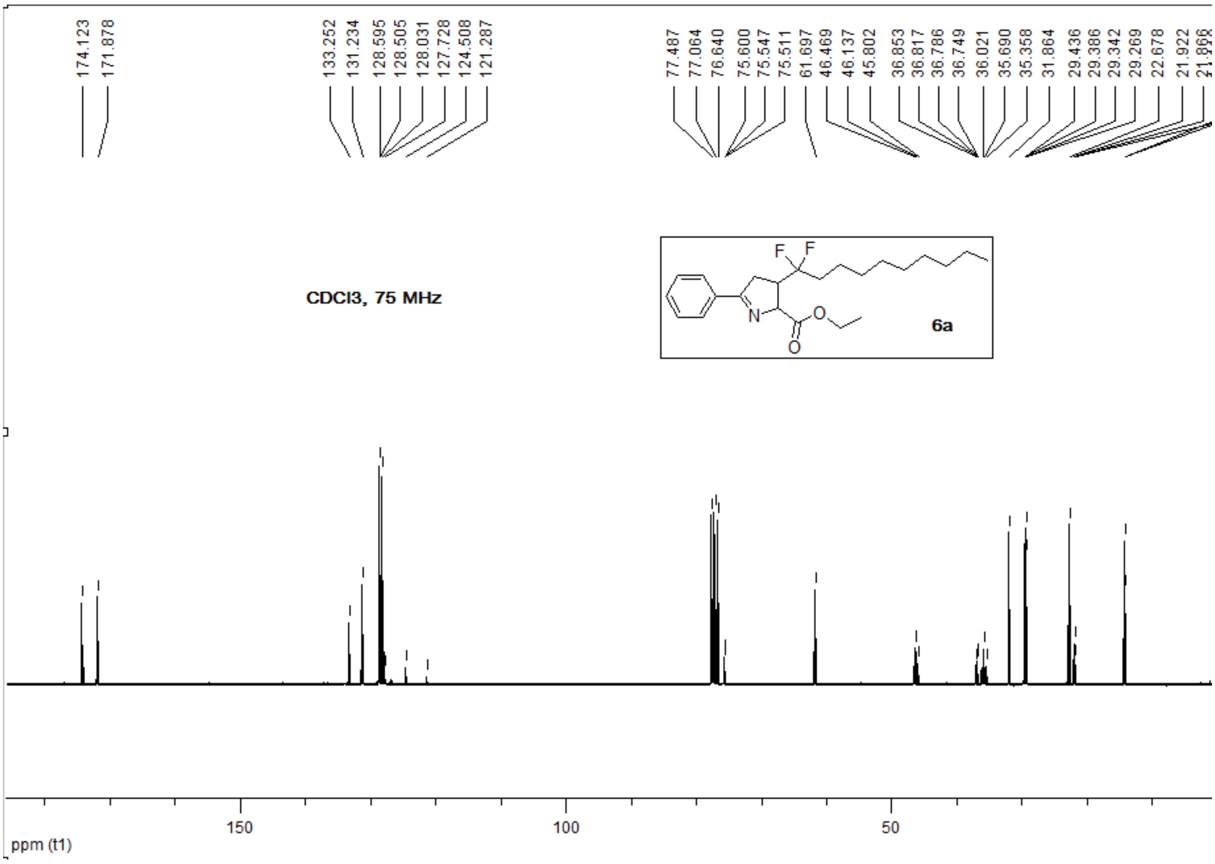
174.123
171.878
133.252
131.234
128.595
128.505
128.031
127.728
124.508
121.287
77.487
77.064
76.640
75.600
75.547
75.511
61.697
46.469
46.137
45.802
36.853
36.817
36.786
36.749
36.021
35.690
35.358
31.864
29.436
29.386
29.342
29.269
22.678
21.922
CDCl3, 75 MHz
F
F
N
O
O
6a
150
100
50
ppm (t1)

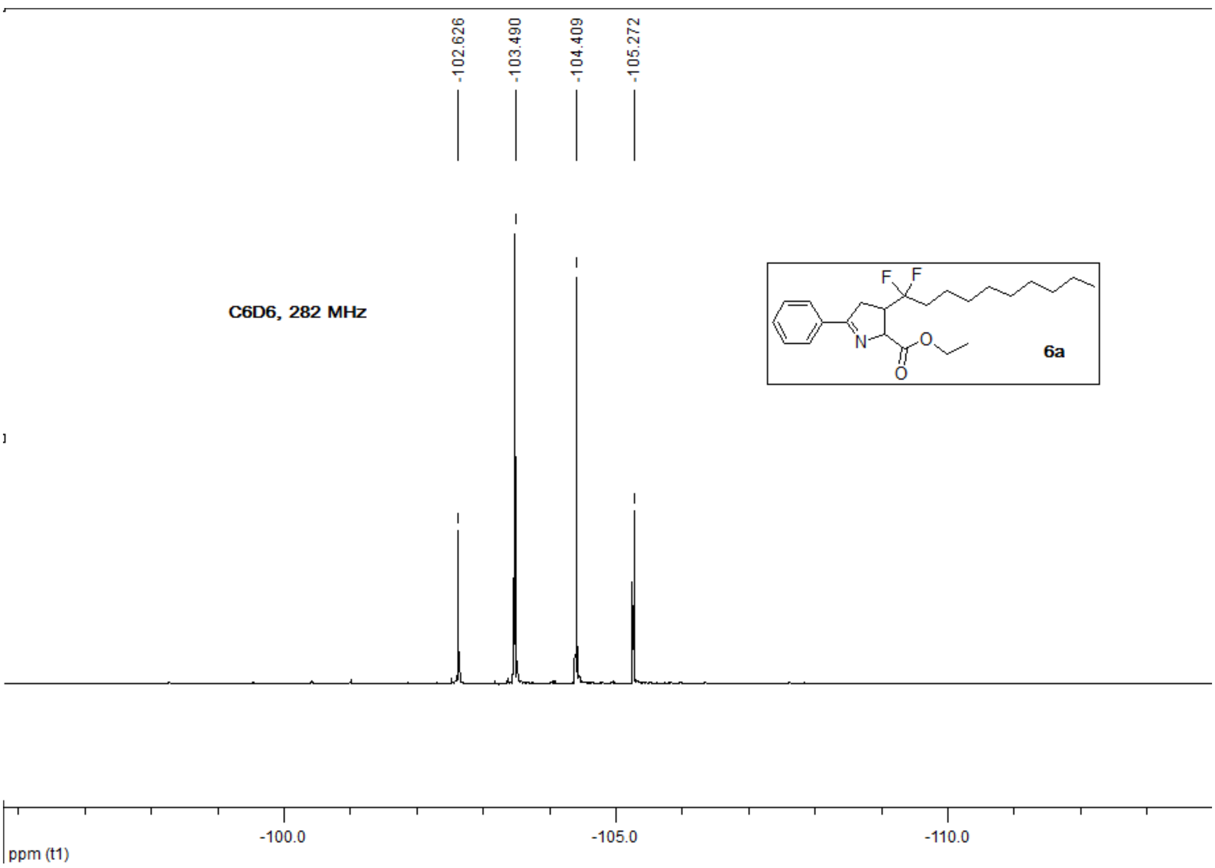
-102.626
-103.490
-104.409
-105.272
C6D6, 282 MHz
F F
N
O
O
6a
-100.0
-105.0
-110.0
ppm (t1)

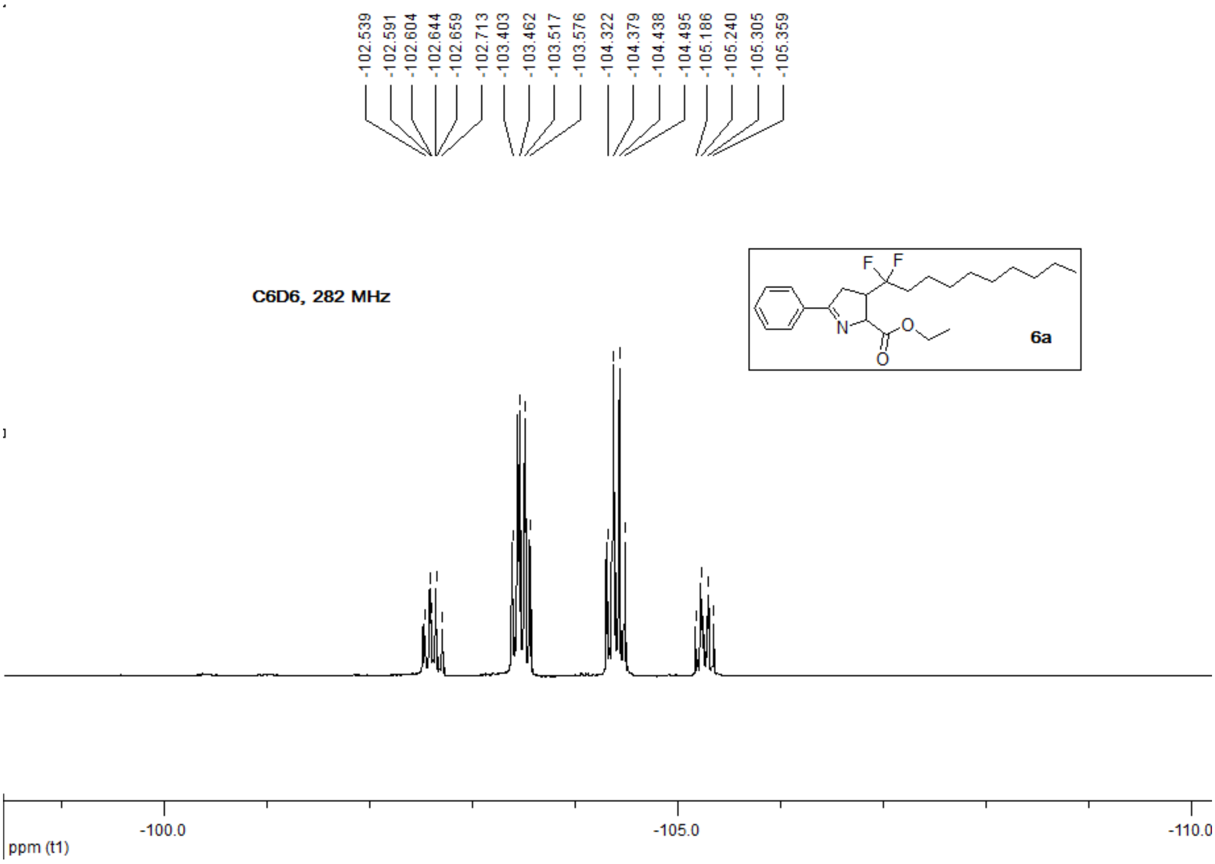
-102.539
-102.591
-102.604
-102.644
-102.659
-102.713
-103.403
-103.462
-103.517
-103.576
-104.322
-104.379
-104.438
-104.495
-105.186
-105.240
-105.305
-105.359
C6D6, 282 MHz
F F
N
O
O
6a
-100.0
-105.0
-110.0
ppm (t1)

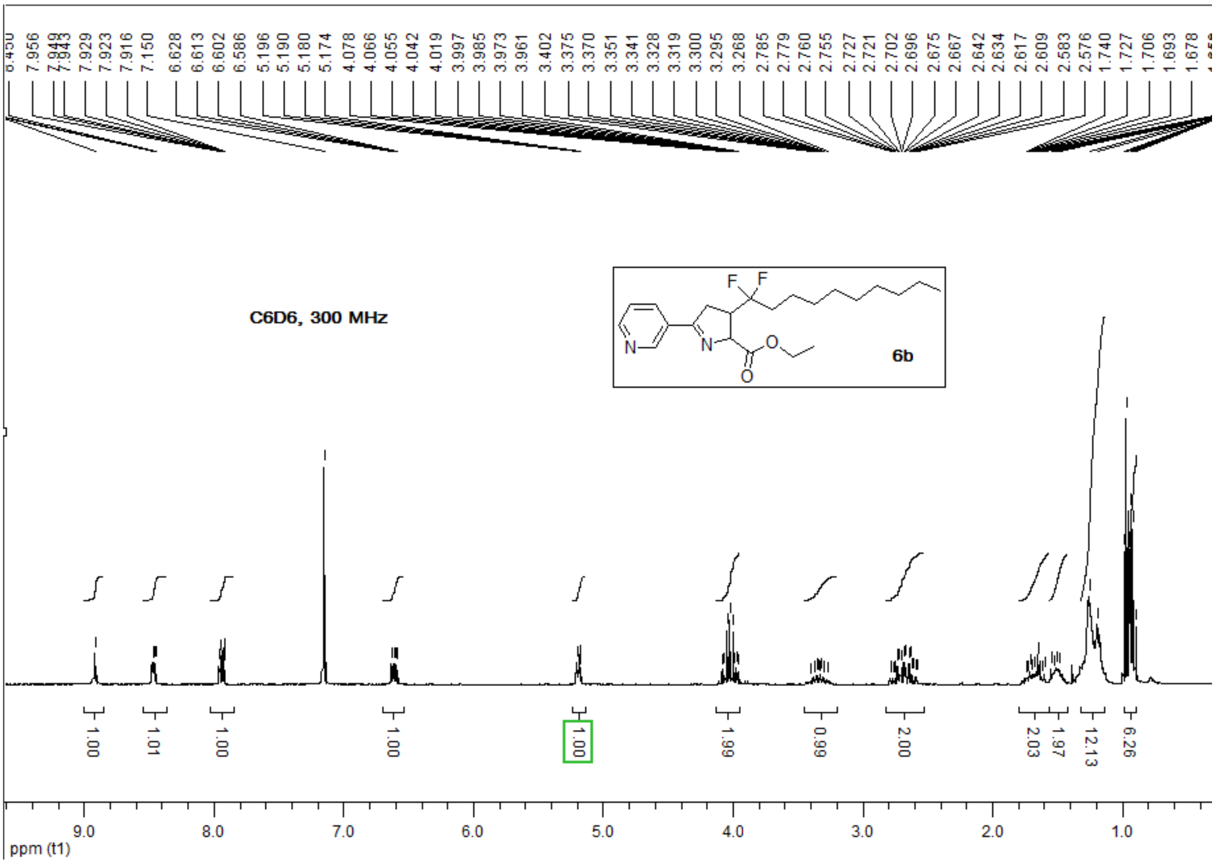
7.956
7.949
7.943
7.929
7.923
7.916
7.150
6.628
6.613
6.602
6.586
5.196
5.190
5.180
5.174
4.078
4.066
4.055
4.042
4.019
3.997
3.985
3.973
3.961
3.402
3.375
3.370
3.351
3.341
3.328
3.319
3.300
3.295
3.268
2.785
2.779
2.760
2.755
2.727
2.721
2.702
2.696
2.675
2.667
2.642
2.634
2.617
2.609
2.583
2.576
1.740
1.727
1.706
1.693
1.678
C6D6, 300 MHz
F
F
N
N
O
O
6b
1.00
1.01
1.00
1.00
1.00
1.99
0.99
2.00
2.03
1.97
12.13
6.26
9.0
8.0
7.0
6.0
5.0
4.0
3.0
2.0
1.0
ppm (t1)

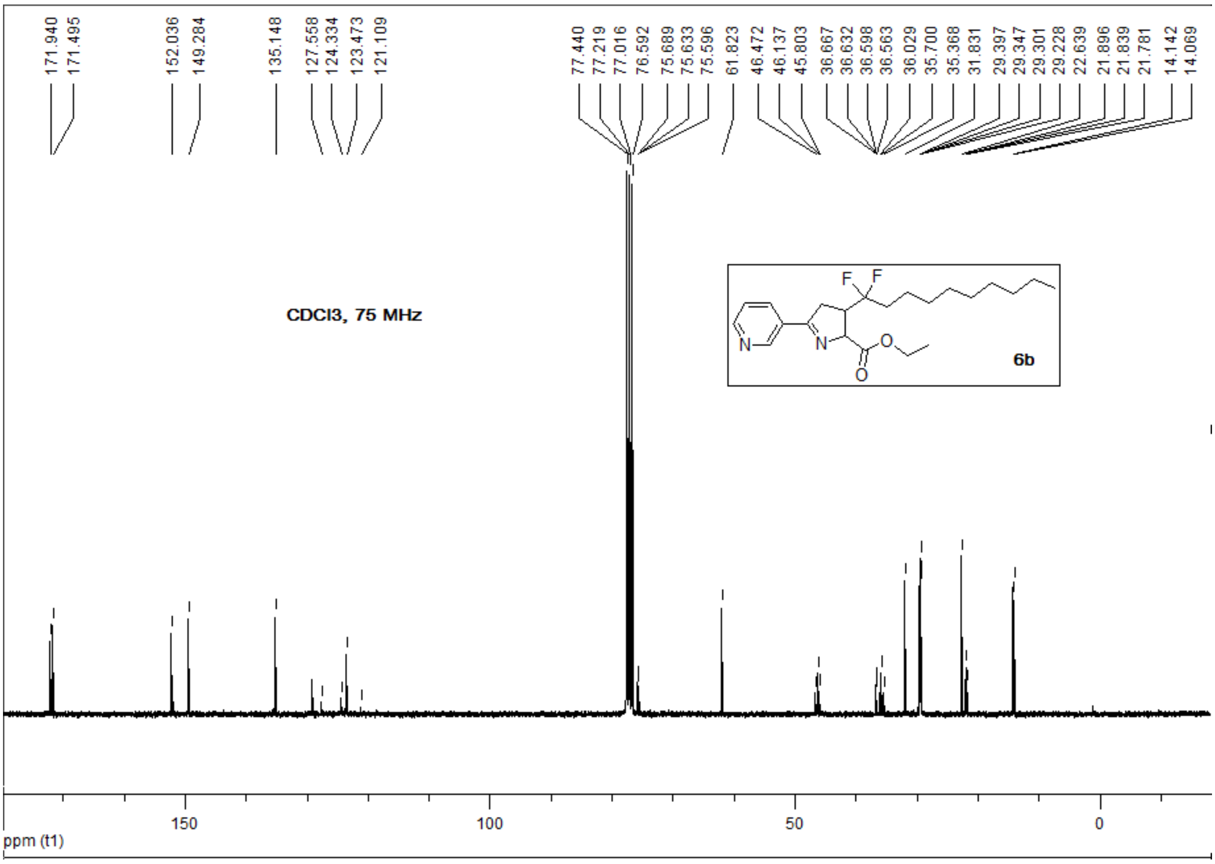
171.940
171.495
152.036
149.284
135.148
127.558
124.334
123.473
121.109
77.440
77.219
77.016
76.592
75.689
75.633
75.596
61.823
46.472
46.137
45.803
36.667
36.632
36.598
36.563
36.029
35.700
35.368
31.831
29.397
29.347
29.301
29.228
22.639
21.896
21.839
21.781
14.142
14.069
CDCl3, 75 MHz
F
F
N
N
O
O
6b
150
100
50
0
ppm (t1)

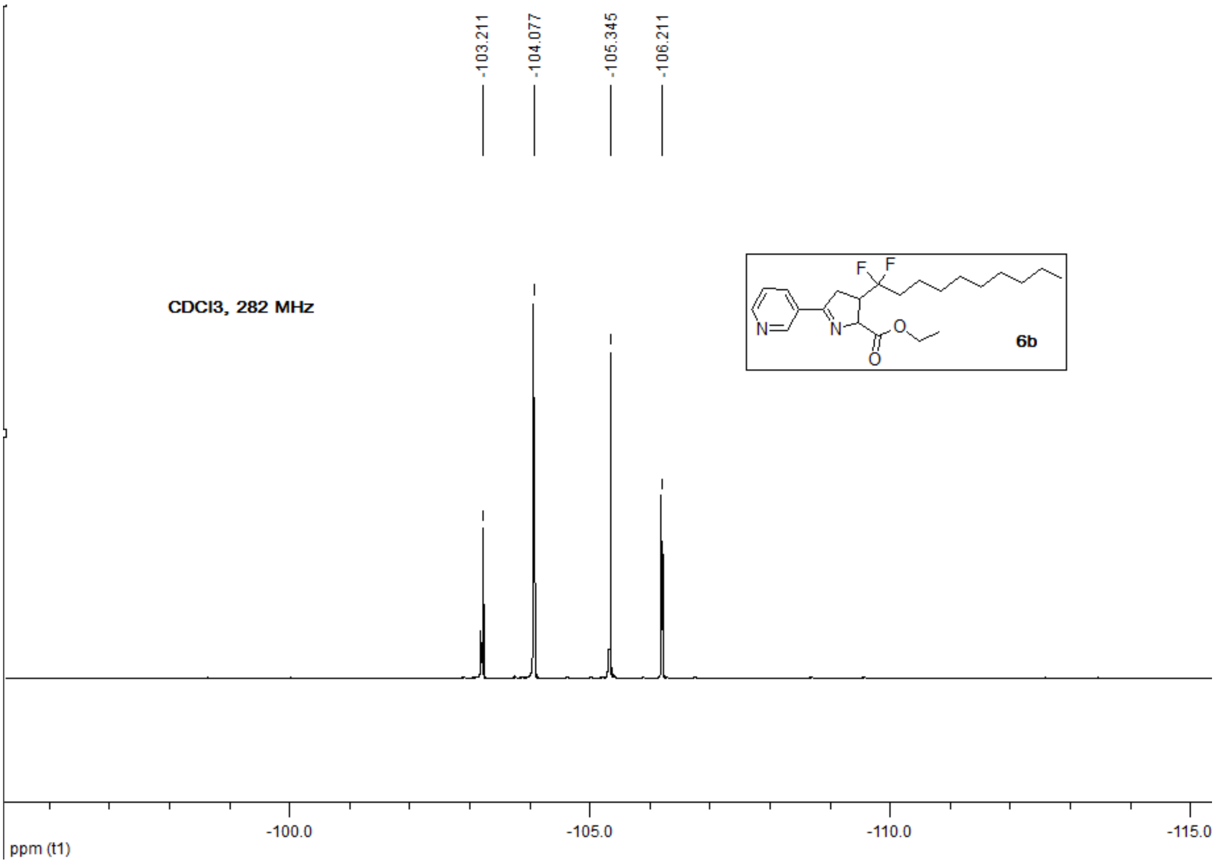

-103.211
-104.077
-105.345
-106.211
CDCl3, 282 MHz
6b
-100.0
-105.0
-110.0
-115.0
ppm (t1)

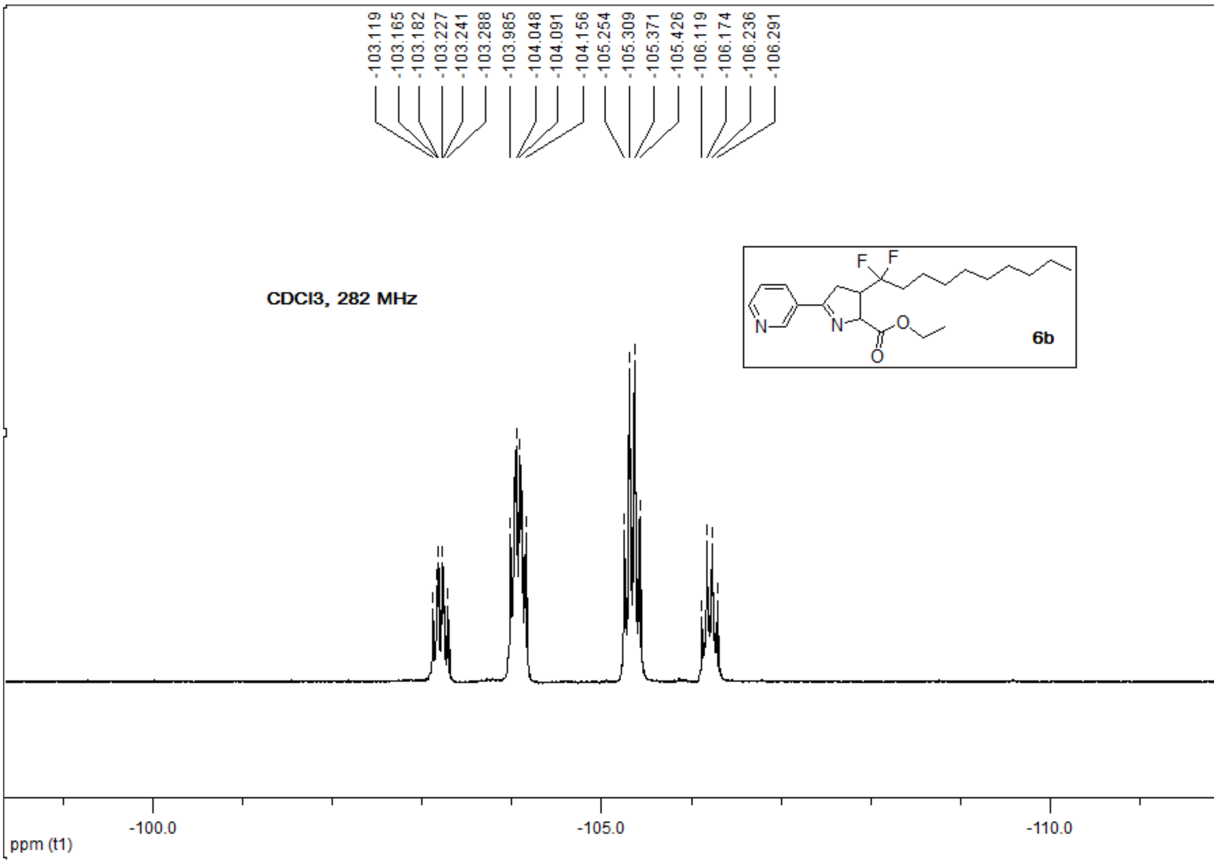

-103.119
-103.165
-103.182
-103.227
-103.241
-103.288
-103.985
-104.048
-104.091
-104.156
-105.254
-105.309
-105.371
-105.426
-106.119
-106.174
-106.236
-106.291
CDCl3, 282 MHz
6b
-100.0
-105.0
-110.0
ppm (t1)

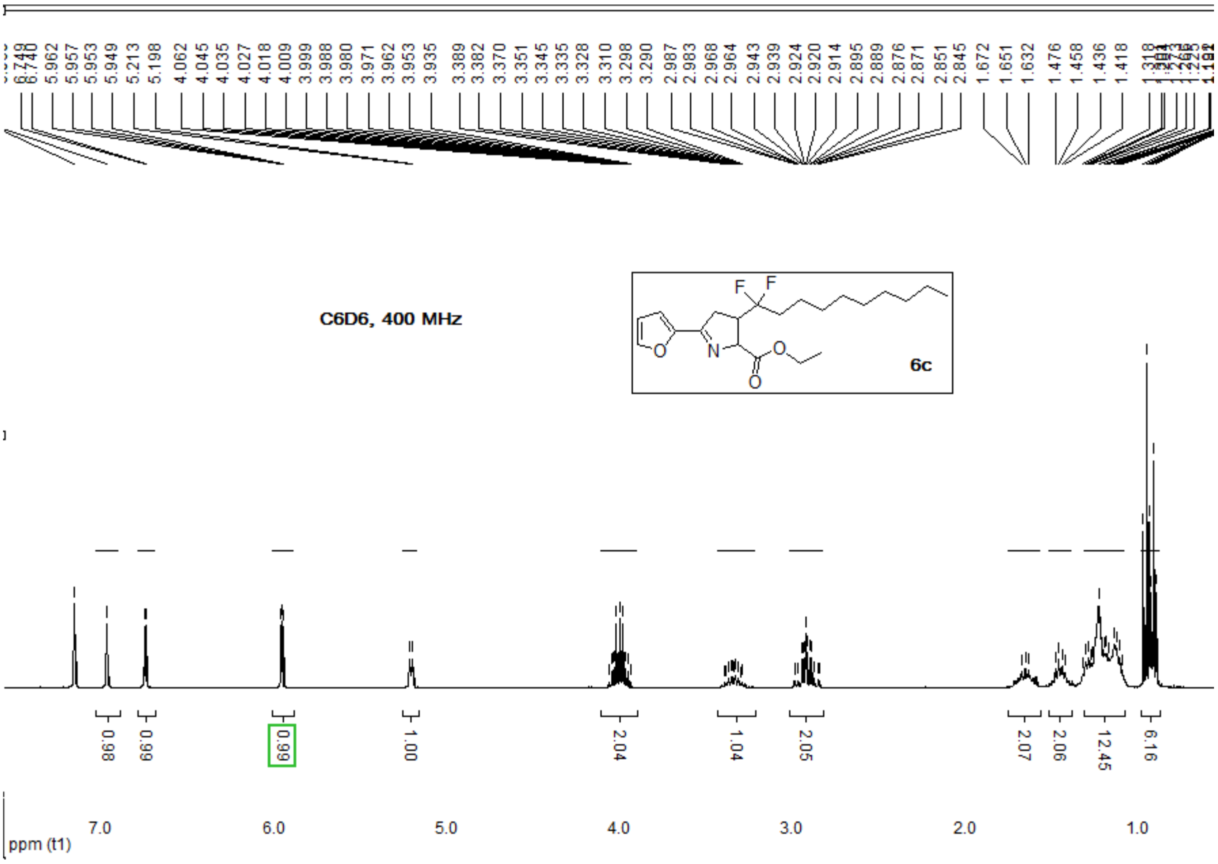
C6D6, 400 MHz
6c
F
F
O
N
O
O
6.749
6.740
5.962
5.957
5.953
5.949
5.213
5.198
4.062
4.045
4.035
4.027
4.018
4.009
3.999
3.988
3.980
3.971
3.962
3.953
3.935
3.389
3.382
3.370
3.351
3.345
3.335
3.328
3.310
3.298
3.290
2.987
2.983
2.968
2.964
2.943
2.939
2.924
2.920
2.914
2.895
2.889
2.876
2.871
2.851
2.845
1.672
1.651
1.632
1.476
1.458
1.436
1.418
1.318
0.98
0.99
0.99
1.00
2.04
1.04
2.05
2.07
2.06
12.45
6.16
7.0
6.0
5.0
4.0
3.0
2.0
1.0
ppm (t1)

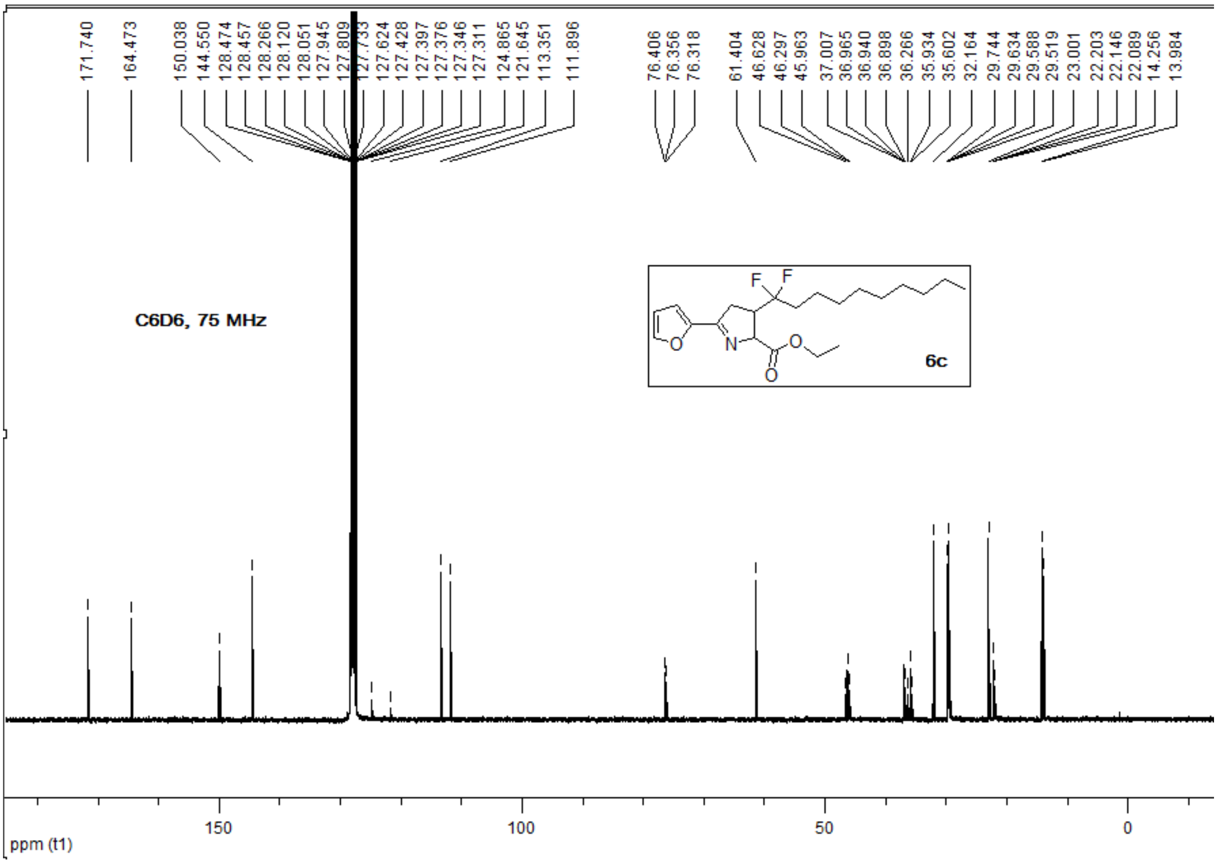
C6D6, 75 MHz
6c
F
F
O
N
O
O
171.740
164.473
150.038
144.550
128.474
128.457
128.266
128.120
128.051
127.945
127.809
127.733
127.624
127.428
127.397
127.376
127.346
127.311
124.865
121.645
113.351
111.896
76.406
76.356
76.318
61.404
46.628
46.297
45.963
37.007
36.965
36.940
36.898
36.266
35.934
35.602
32.164
29.744
29.634
29.588
29.519
23.001
22.203
22.146
22.089
14.256
13.984
150
100
50
0
ppm (t1)

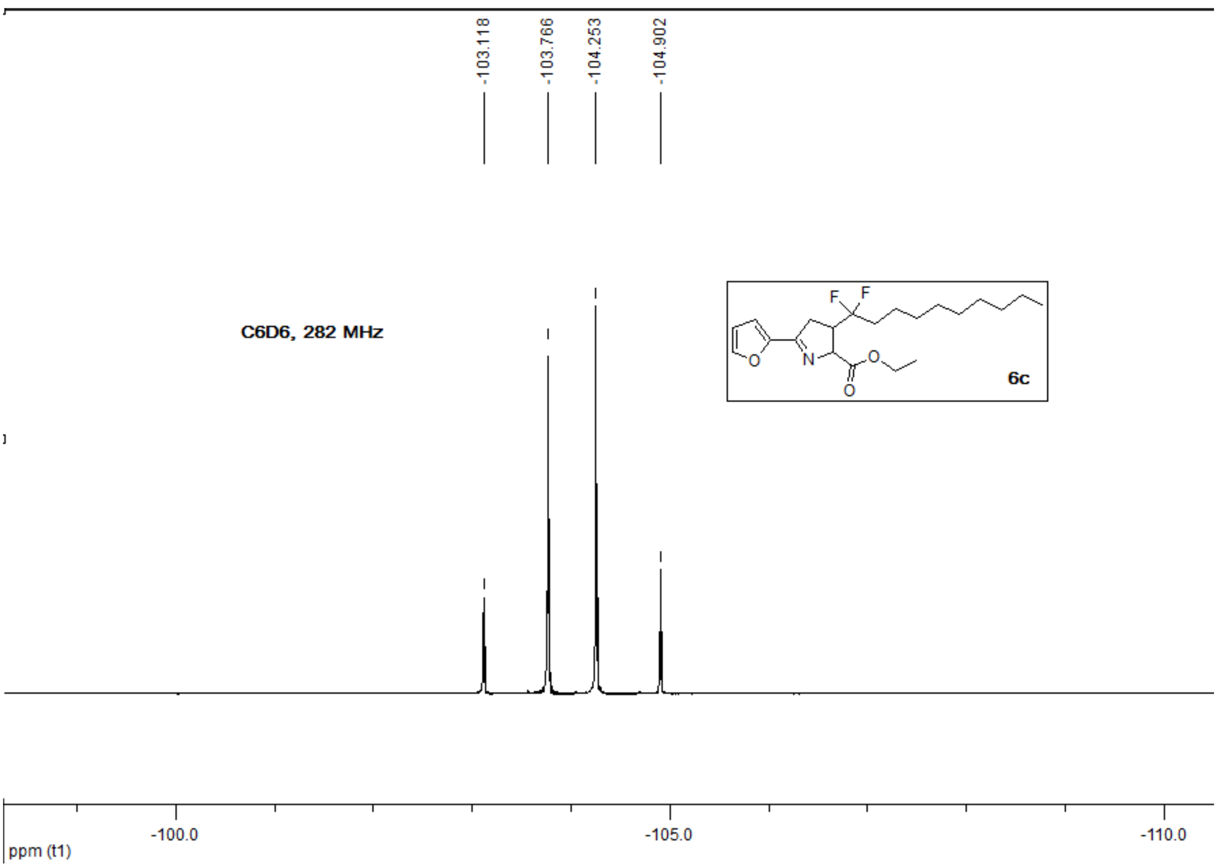

-103.118
-103.766
-104.253
-104.902
C6D6, 282 MHz
F
F
O
N
O
O
6c
-100.0
-105.0
-110.0
ppm (t1)

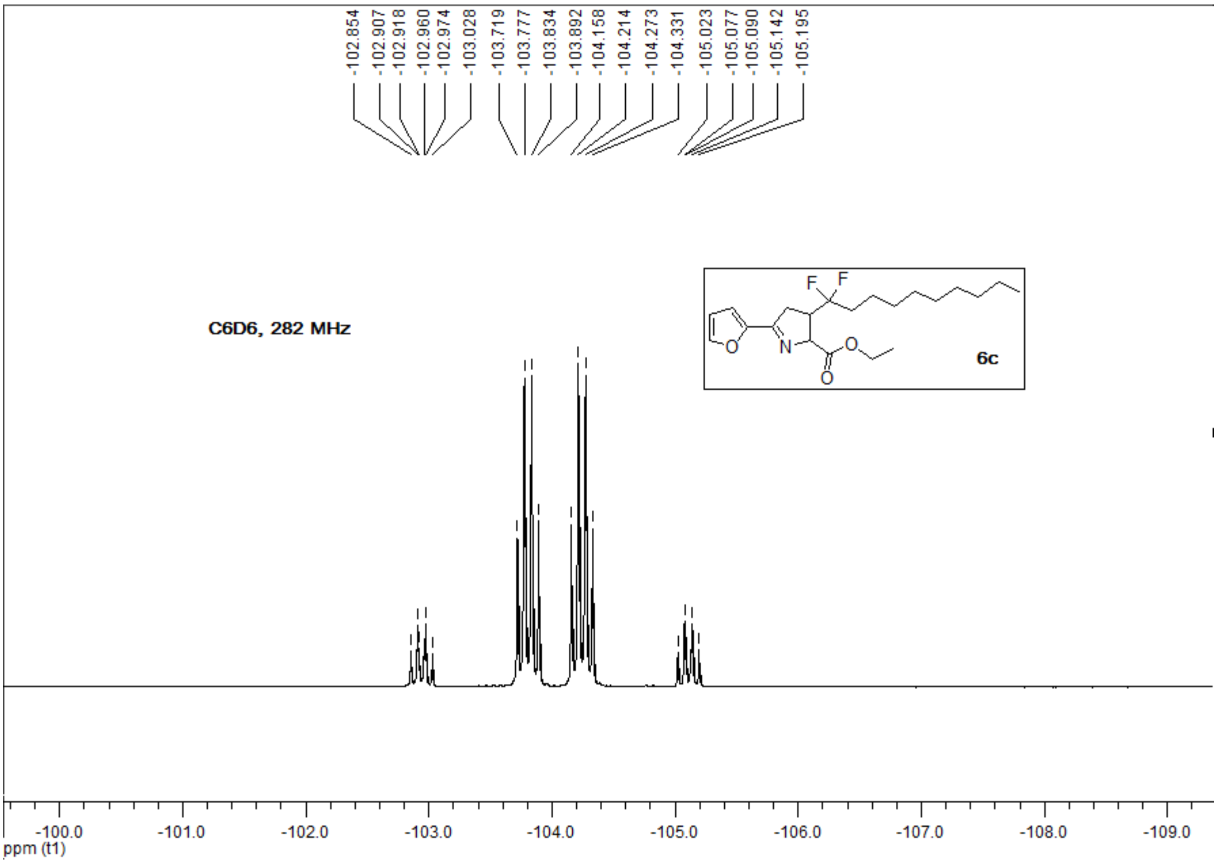

-102.854
-102.907
-102.918
-102.960
-102.974
-103.028
-103.719
-103.777
-103.834
-103.892
-104.158
-104.214
-104.273
-104.331
-105.023
-105.077
-105.090
-105.142
-105.195
C6D6, 282 MHz
F
F
O
N
O
O
6c
-100.0
-101.0
-102.0
-103.0
-104.0
-105.0
-106.0
-107.0
-108.0
-109.0
ppm (t1)

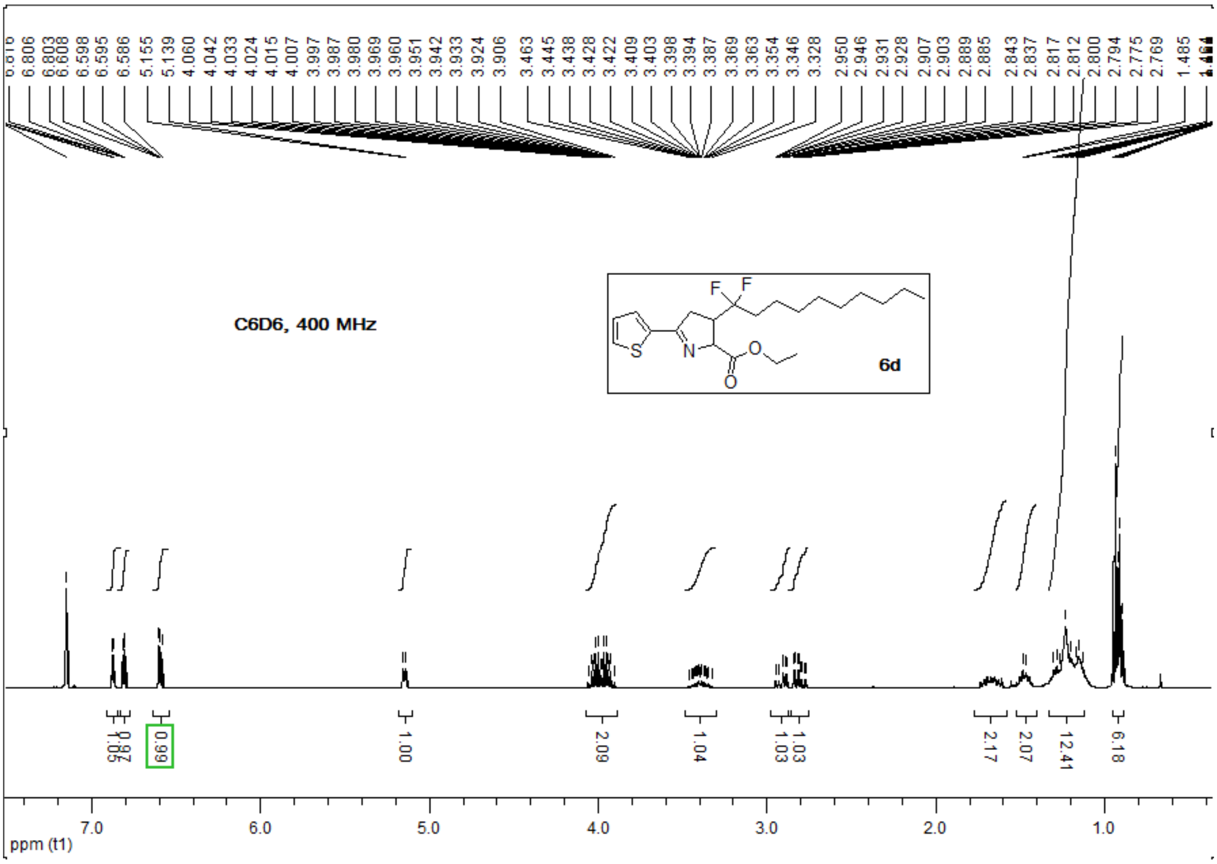
C6D6, 400 MHz
6d
ppm (t1)

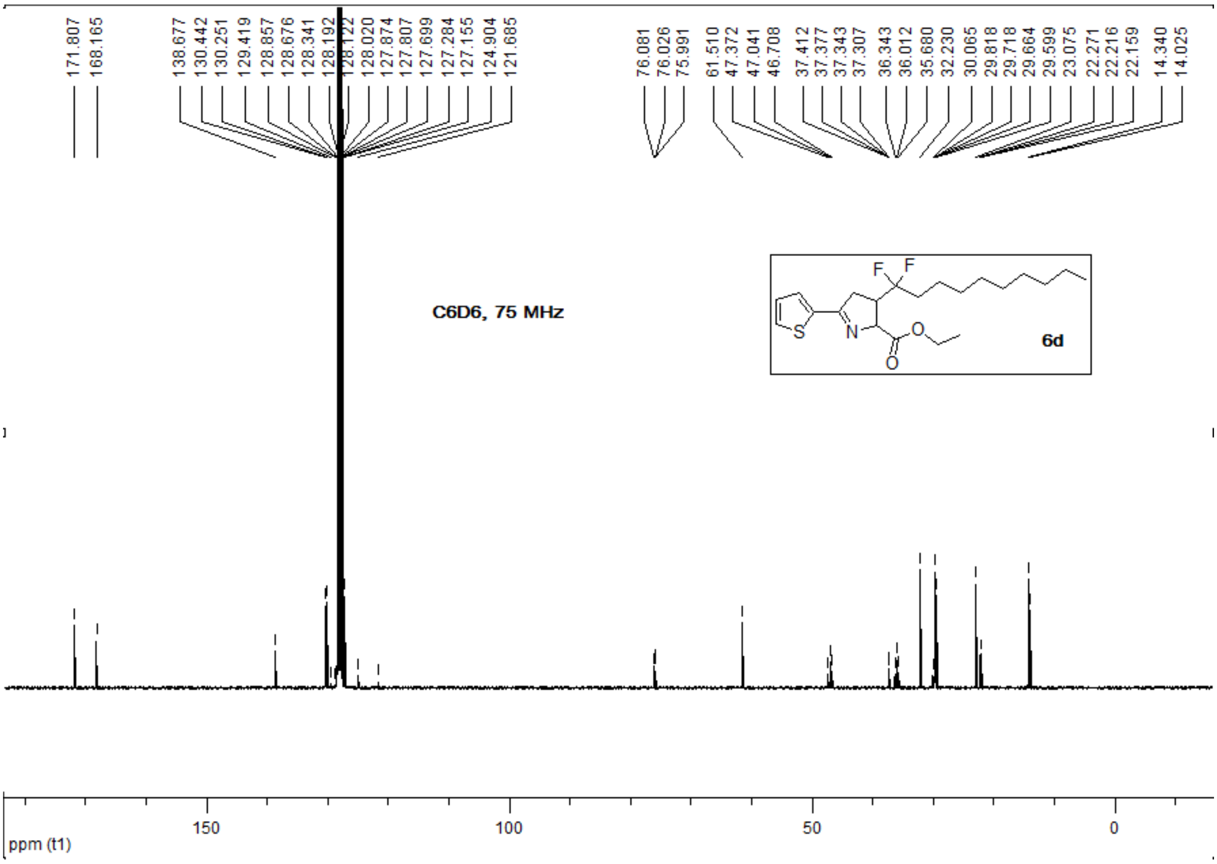
C6D6, 75 MHz
6d
ppm (t1)

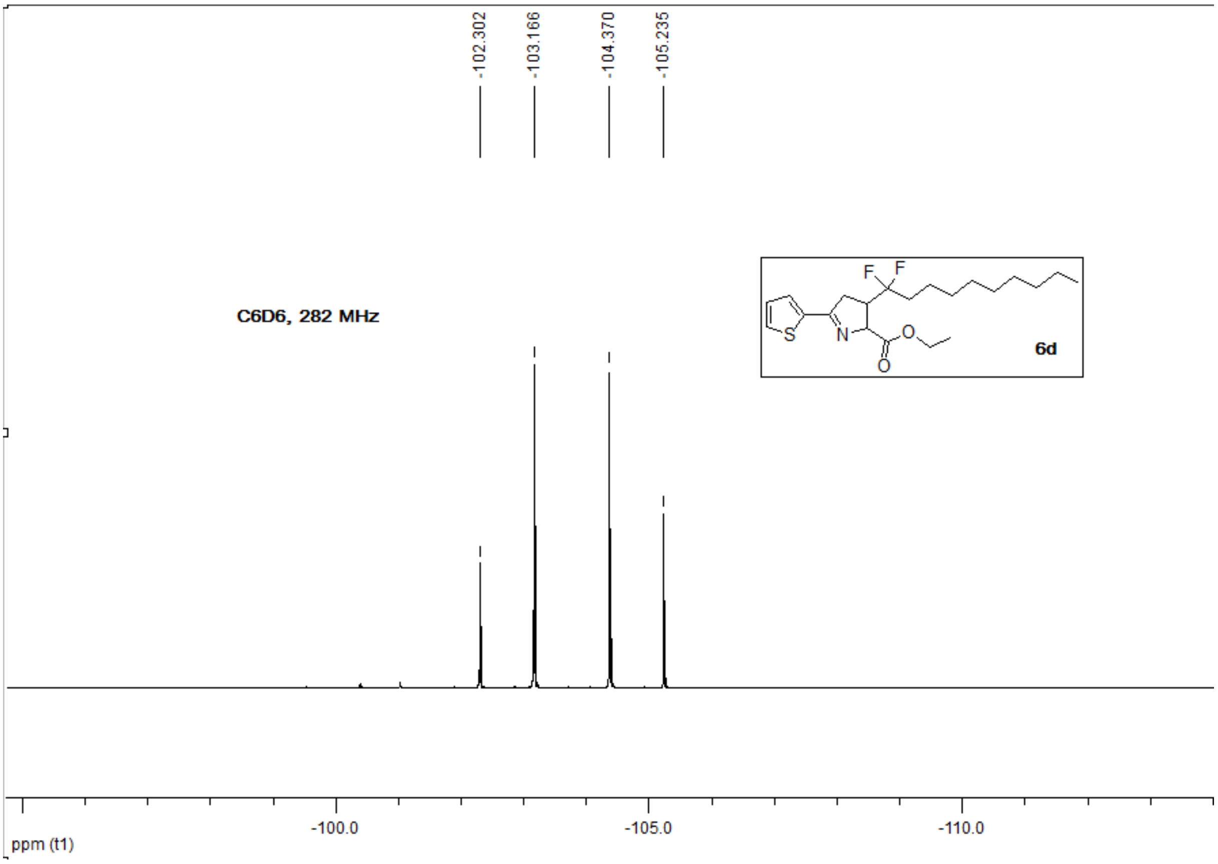

-102.302
-103.166
-104.370
-105.235
C6D6, 282 MHz
6d
-100.0
-105.0
-110.0
ppm (t1)

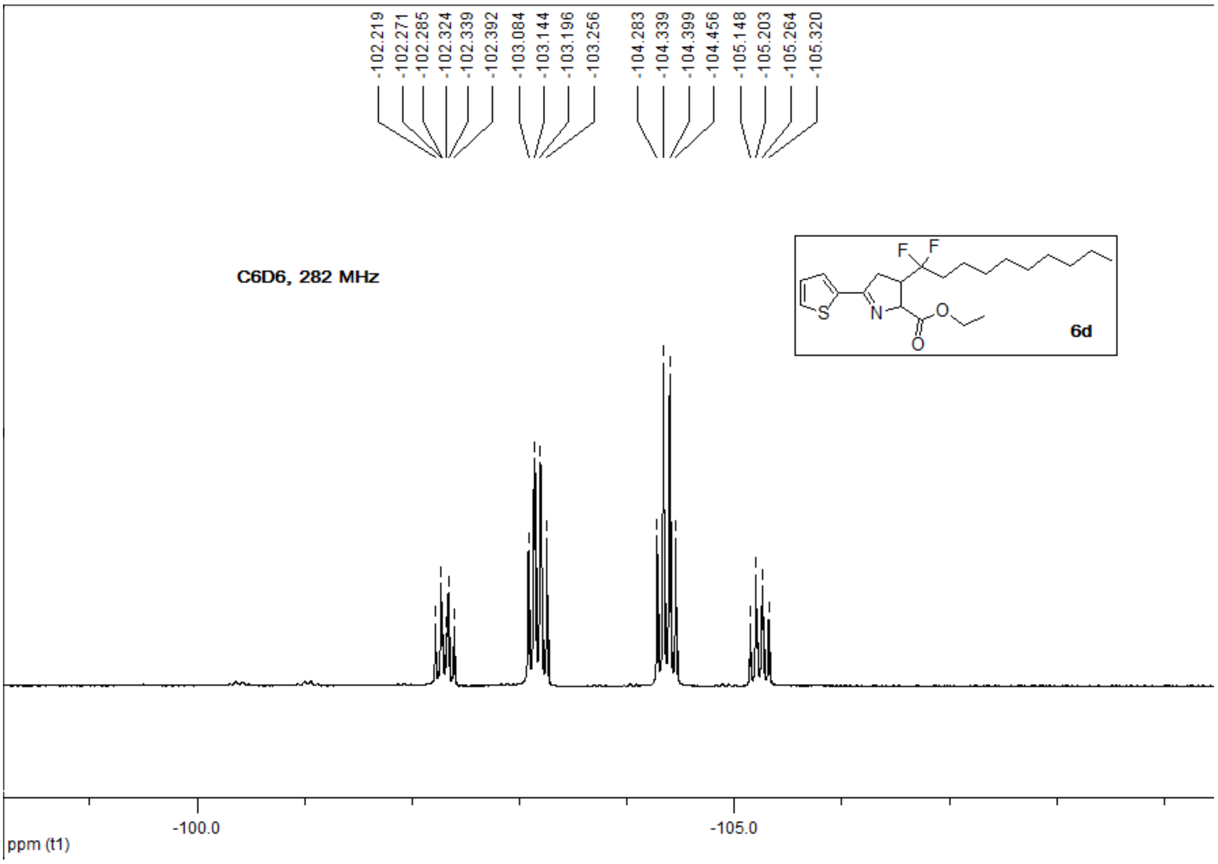

-102.219
-102.271
-102.285
-102.324
-102.339
-102.392
-103.084
-103.144
-103.196
-103.256
-104.283
-104.339
-104.399
-104.456
-105.148
-105.203
-105.264
-105.320
C6D6, 282 MHz
6d
-100.0
-105.0
ppm (t1)

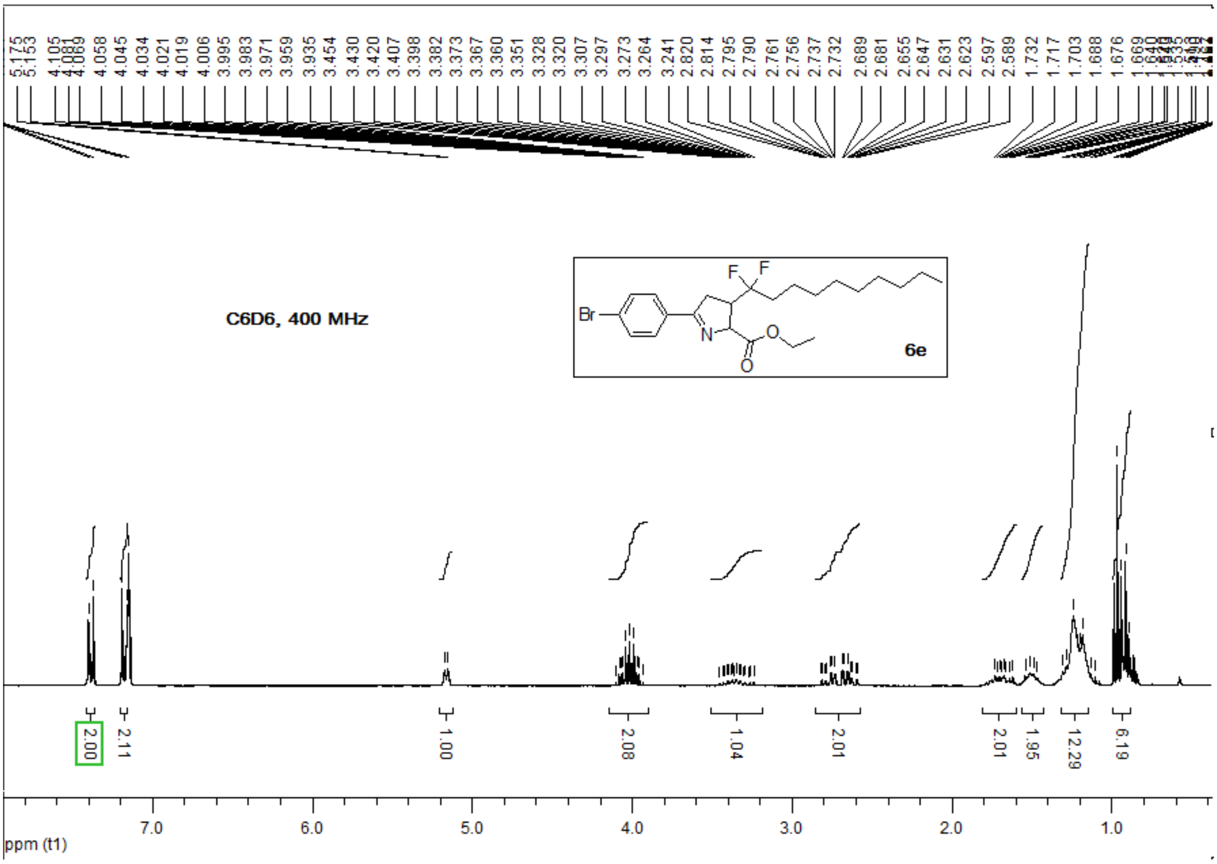
C6D6, 400 MHz
6e
ppm (t1)

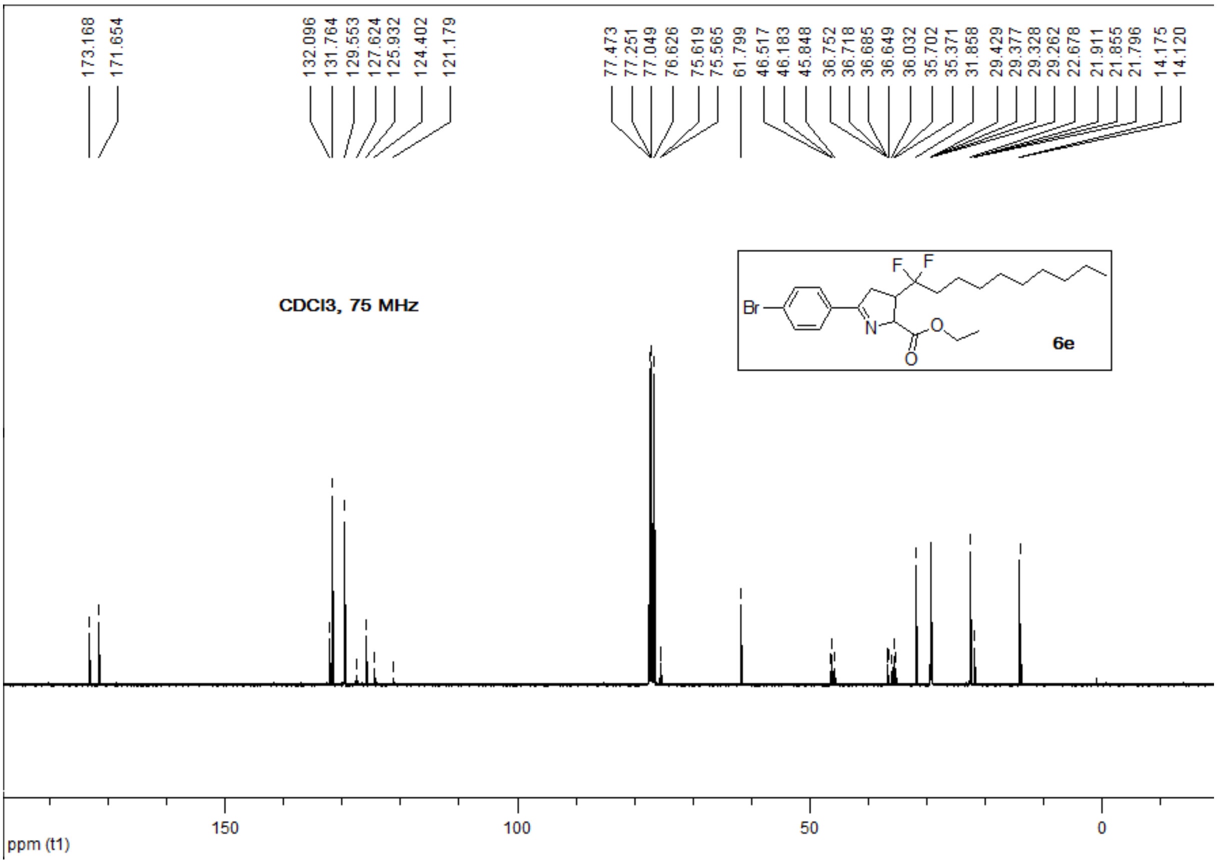
CDCl3, 75 MHz
6e
ppm (t1)

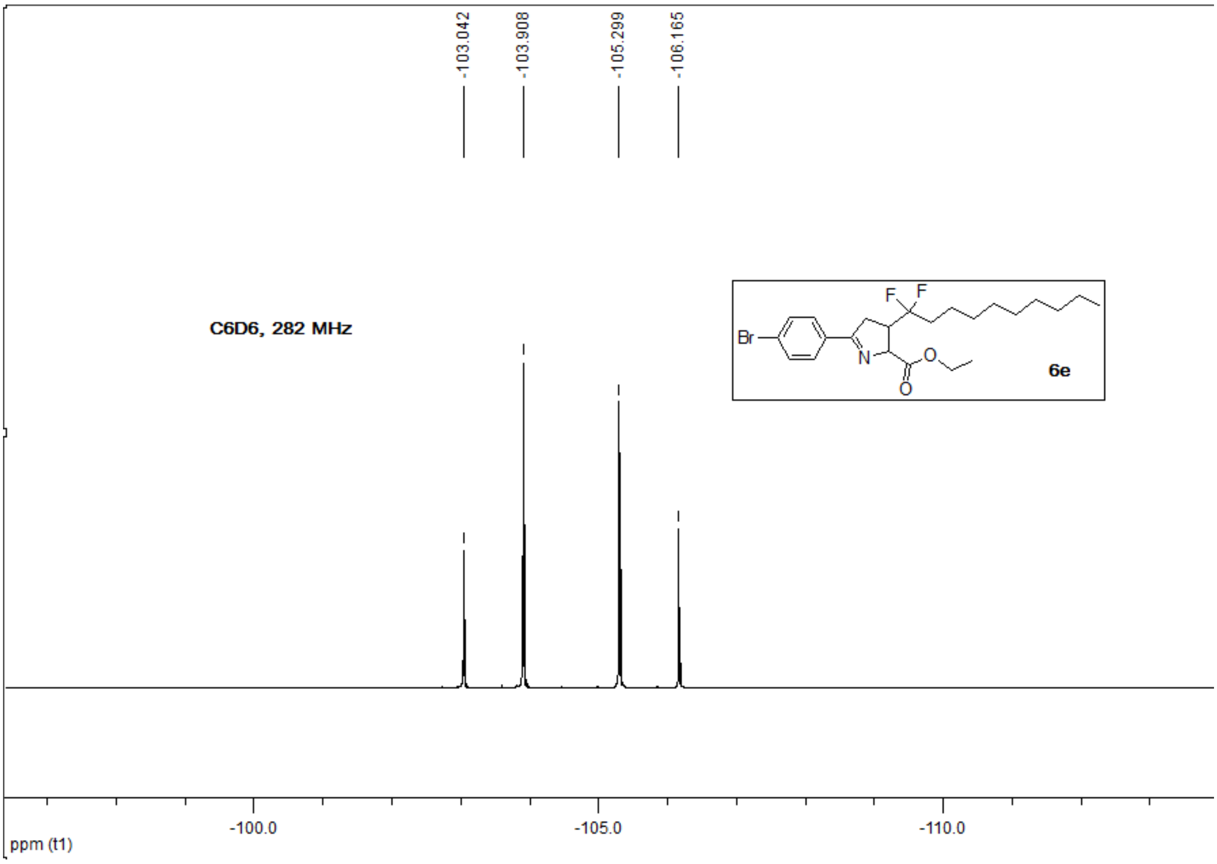
-103.042
-103.908
-105.299
-106.165
C6D6, 282 MHz
Br
F
F
N
O
O
6e
-100.0
-105.0
-110.0
ppm (t1)

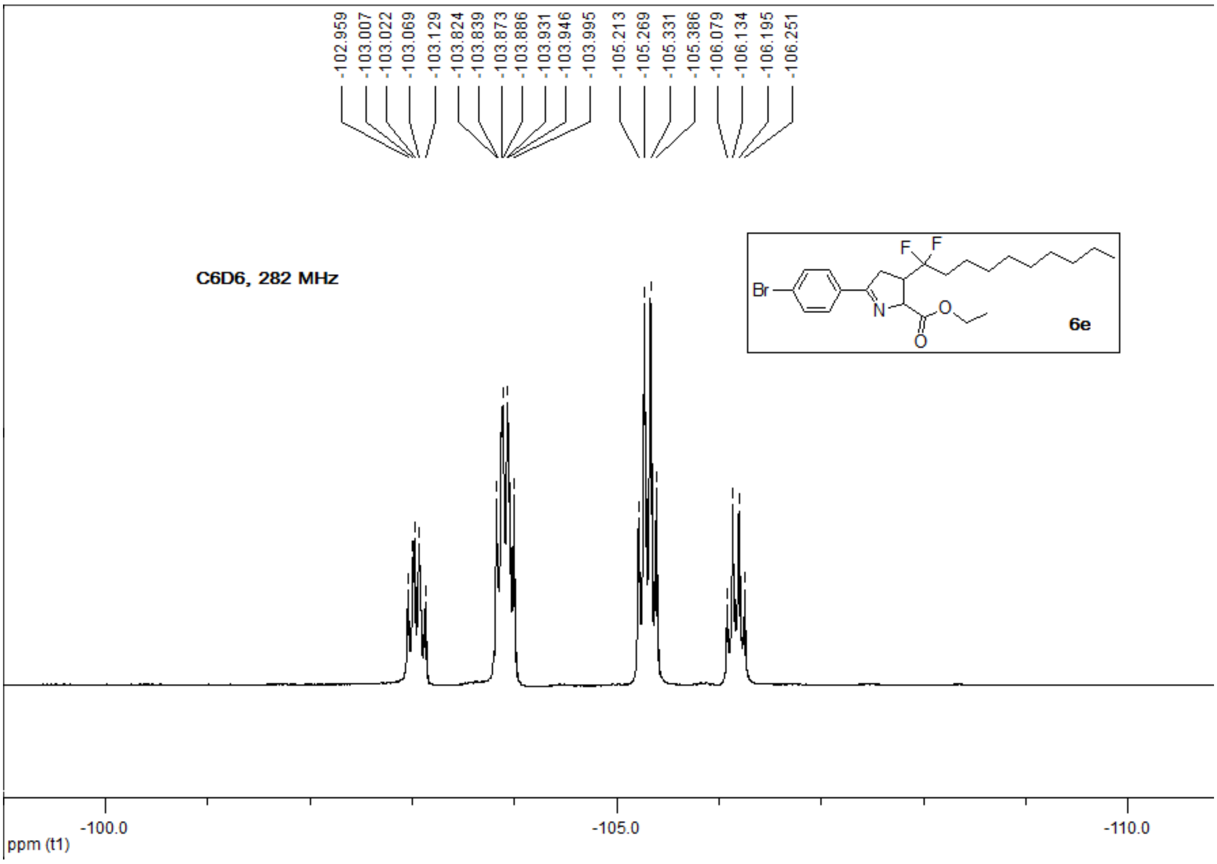
-102.959
-103.007
-103.022
-103.069
-103.129
-103.824
-103.839
-103.873
-103.886
-103.931
-103.946
-103.995
-105.213
-105.269
-105.331
-105.386
-106.079
-106.134
-106.195
-106.251
C6D6, 282 MHz
Br
F
F
N
O
O
6e
-100.0
-105.0
-110.0
ppm (t1)

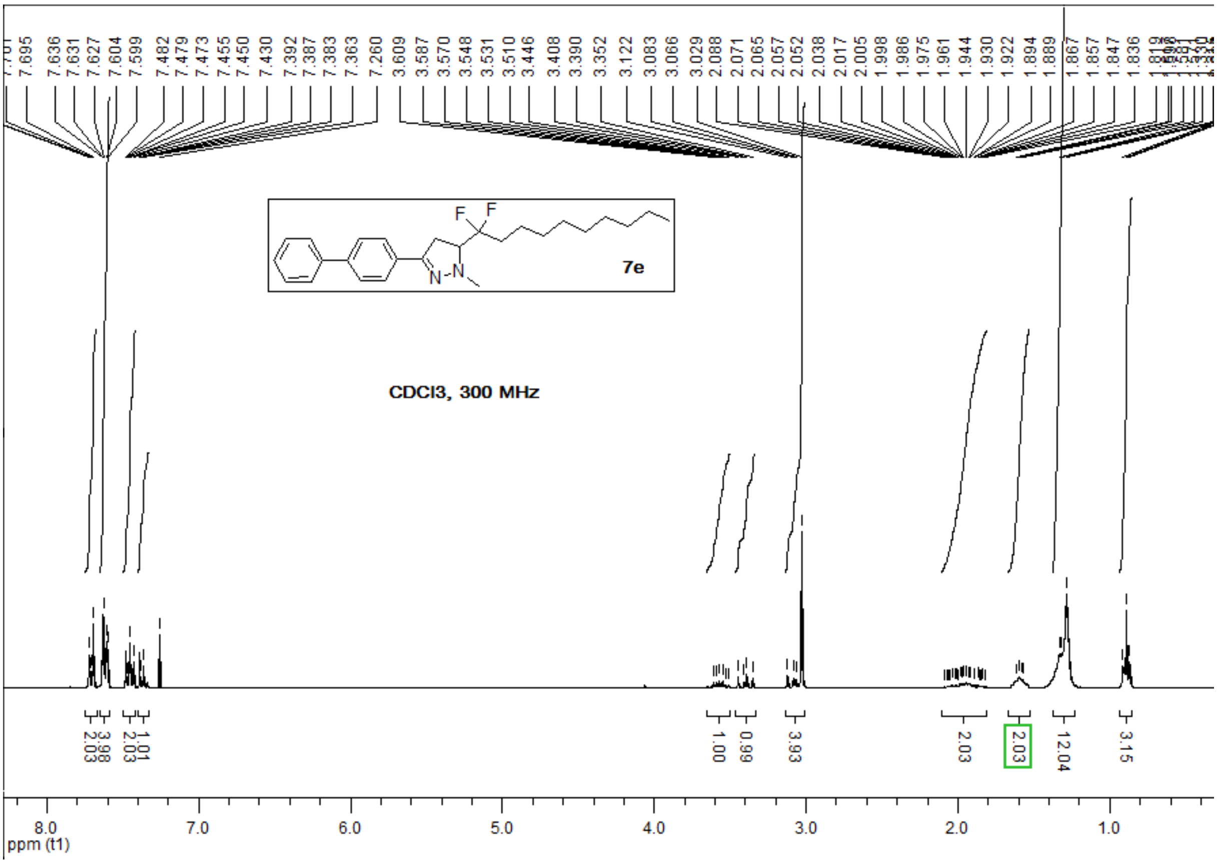
CDCl3, 300 MHz
7e
F F
N-N
ppm (t1)
8.0
7.0
6.0
5.0
4.0
3.0
2.0
1.0
2.03
3.98
2.03
1.01
1.00
0.99
3.93
2.03
2.03
12.04
3.15

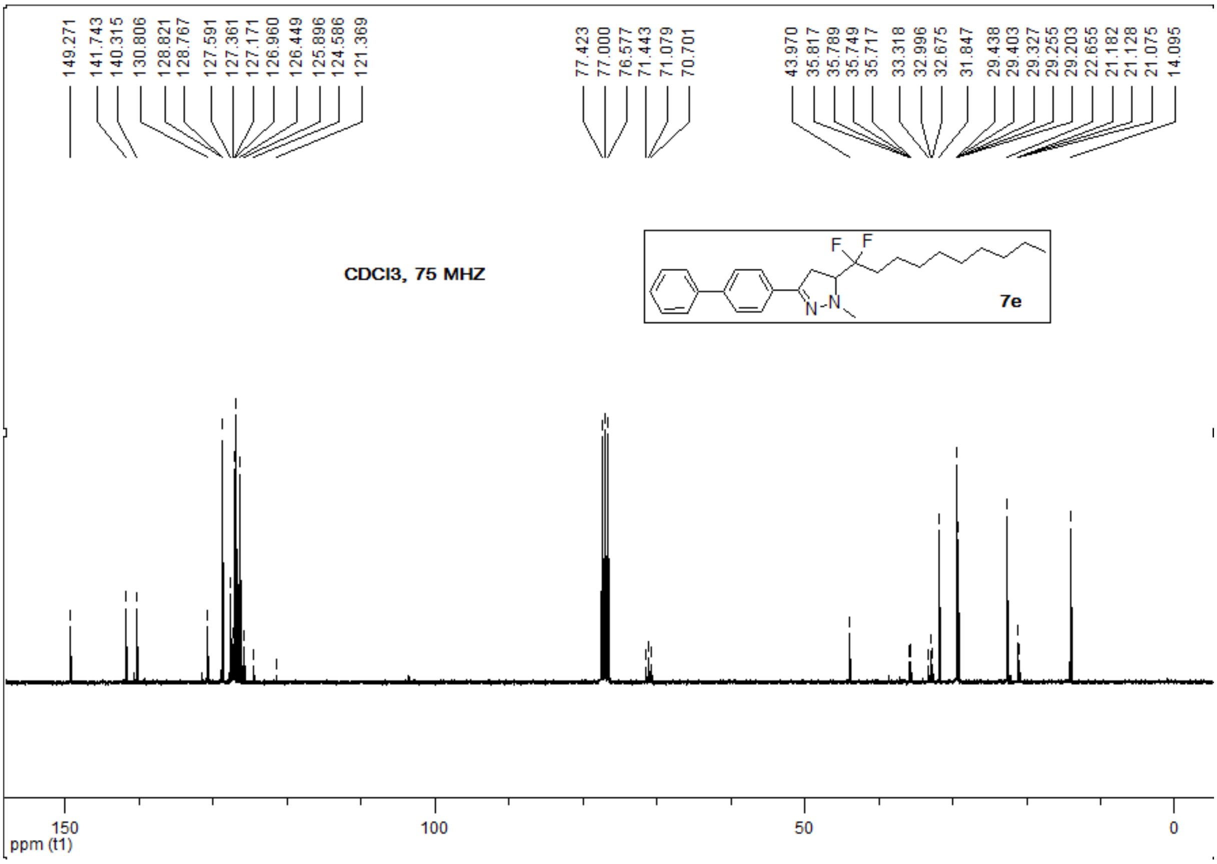
CDCl3, 75 MHZ
7e
F F
N-N
149.271
141.743
140.315
130.806
128.821
128.767
127.591
127.361
127.171
126.960
126.449
125.896
124.586
121.369
77.423
77.000
76.577
71.443
71.079
70.701
43.970
35.817
35.789
35.749
35.717
33.318
32.996
32.675
31.847
29.438
29.403
29.327
29.255
29.203
22.655
21.182
21.128
21.075
14.095
ppm (t1)
150
100
50
0

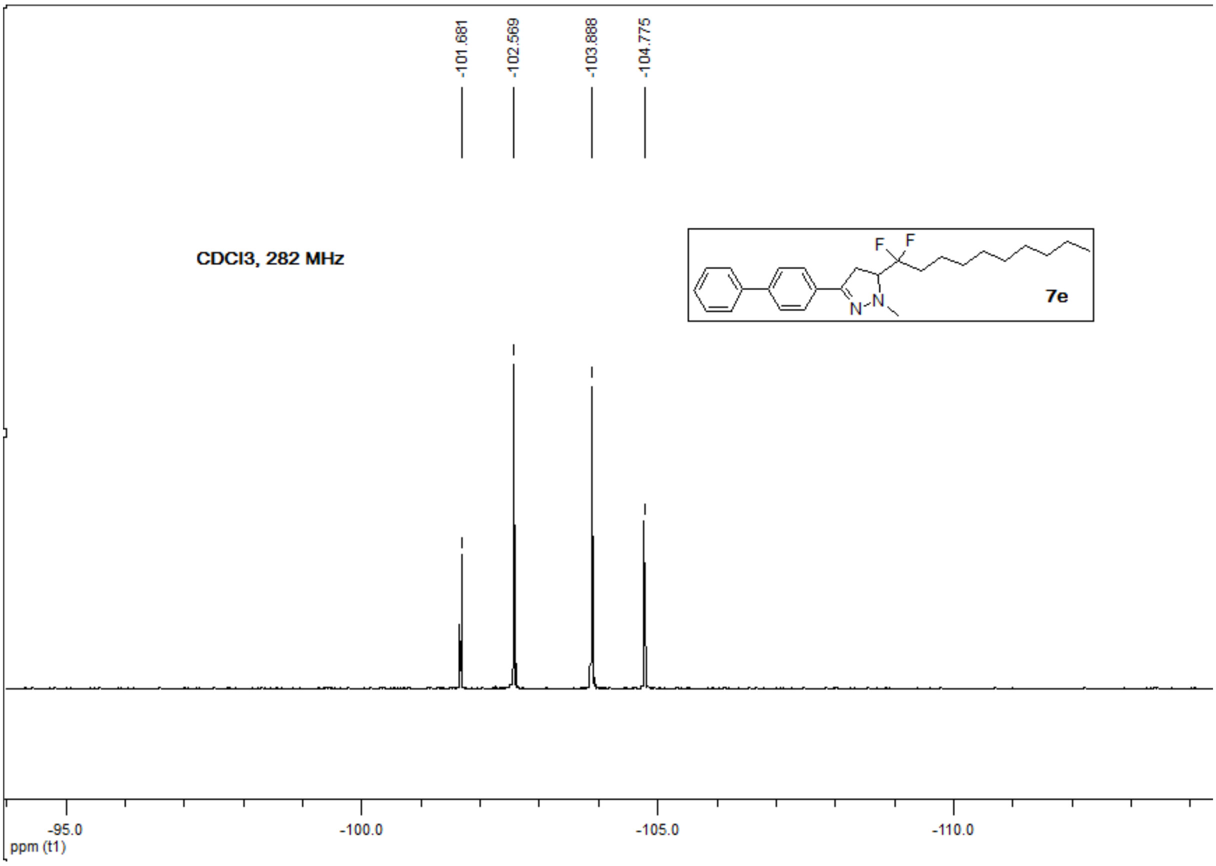
-101.681
-102.569
-103.888
-104.775
CDCl3, 282 MHz
F F
N N
7e
-95.0
-100.0
-105.0
-110.0
ppm (t1)

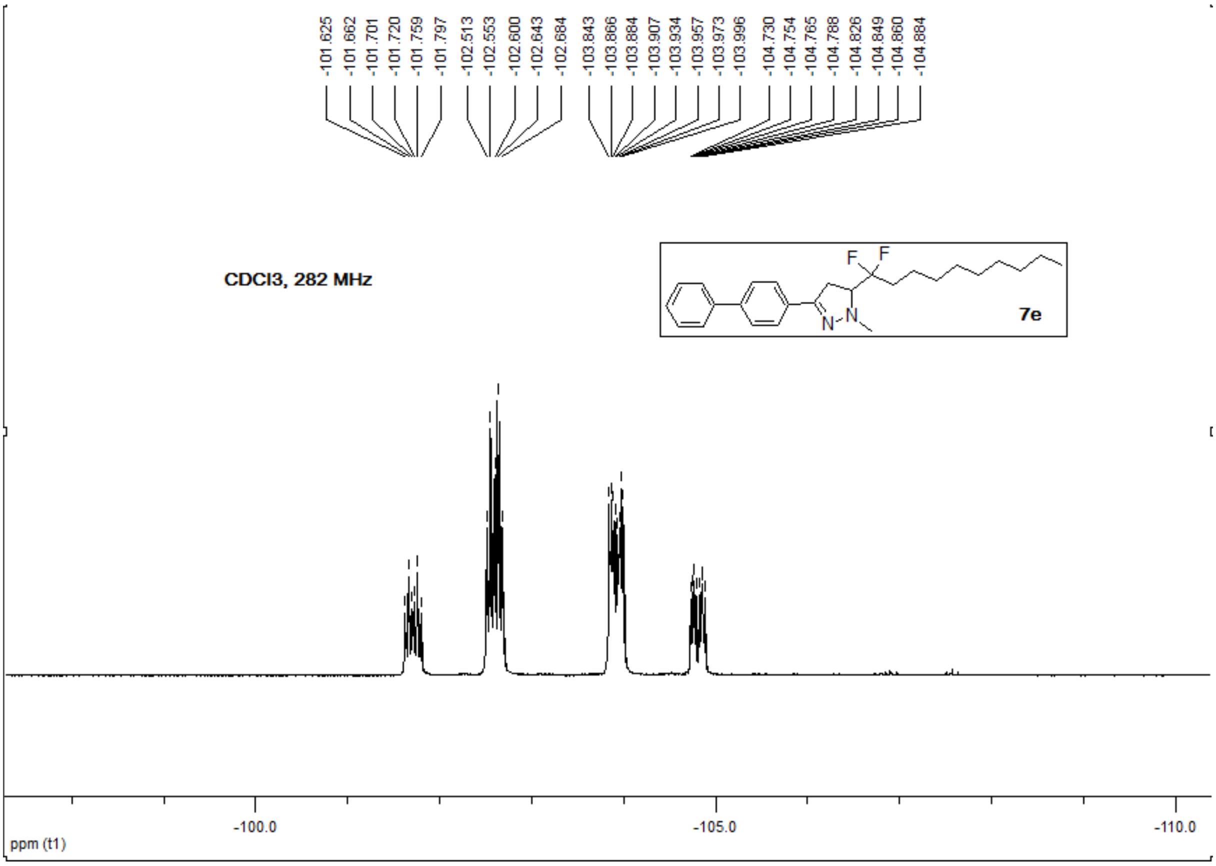
-101.625
-101.662
-101.701
-101.720
-101.759
-101.797
-102.513
-102.553
-102.600
-102.643
-102.684
-103.843
-103.866
-103.884
-103.907
-103.934
-103.957
-103.973
-103.996
-104.730
-104.754
-104.765
-104.788
-104.826
-104.849
-104.860
-104.884
CDCl3, 282 MHz
F F
N N
7e
-100.0
-105.0
-110.0
ppm (t1)

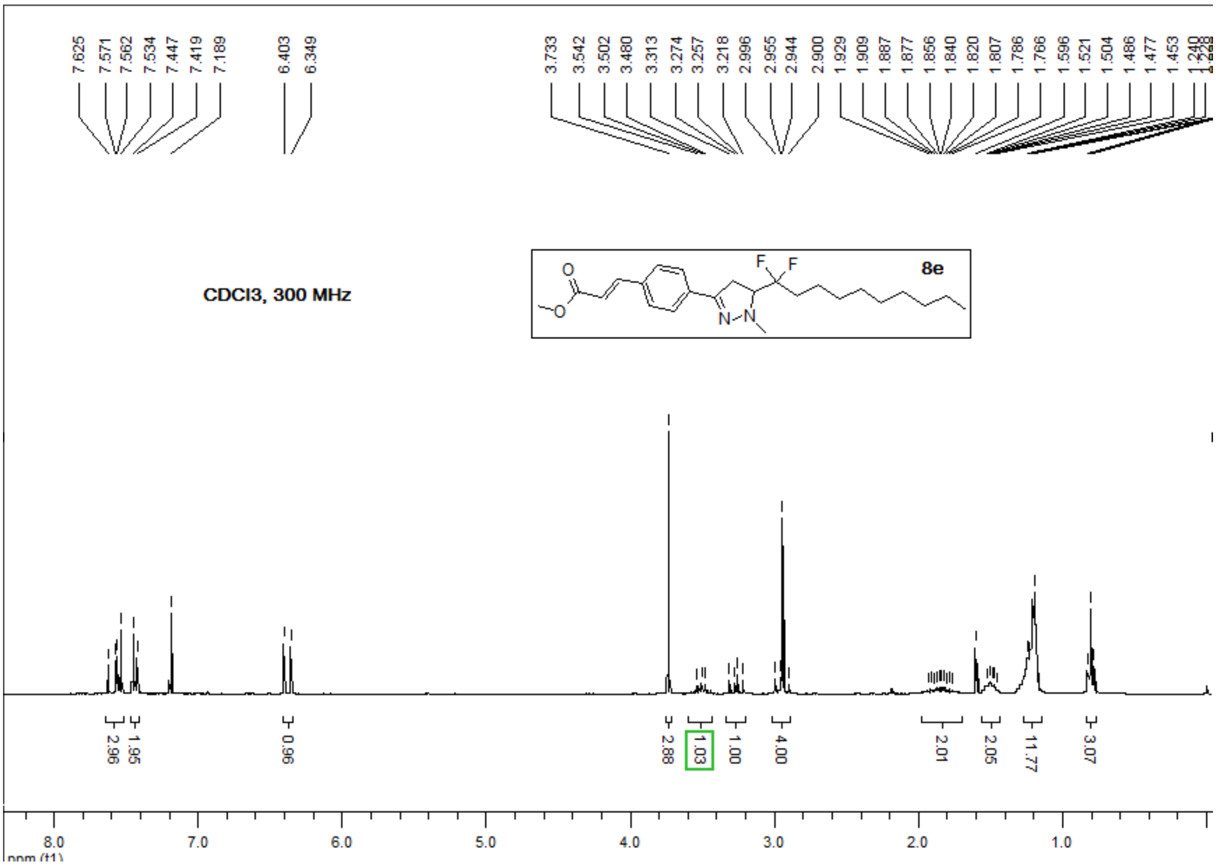
CDCl3, 300 MHz
8e
ppm (t1)

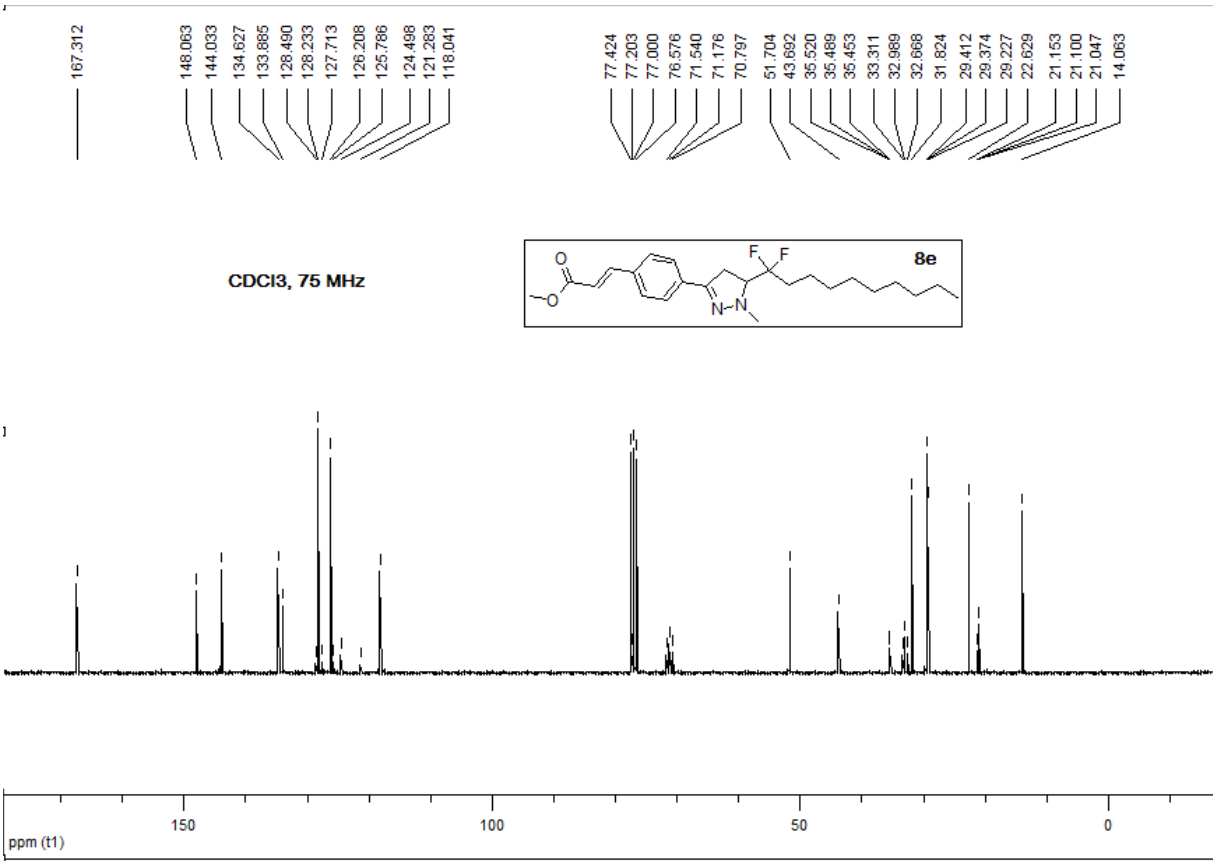
CDCl3, 75 MHz
8e
ppm (t1)

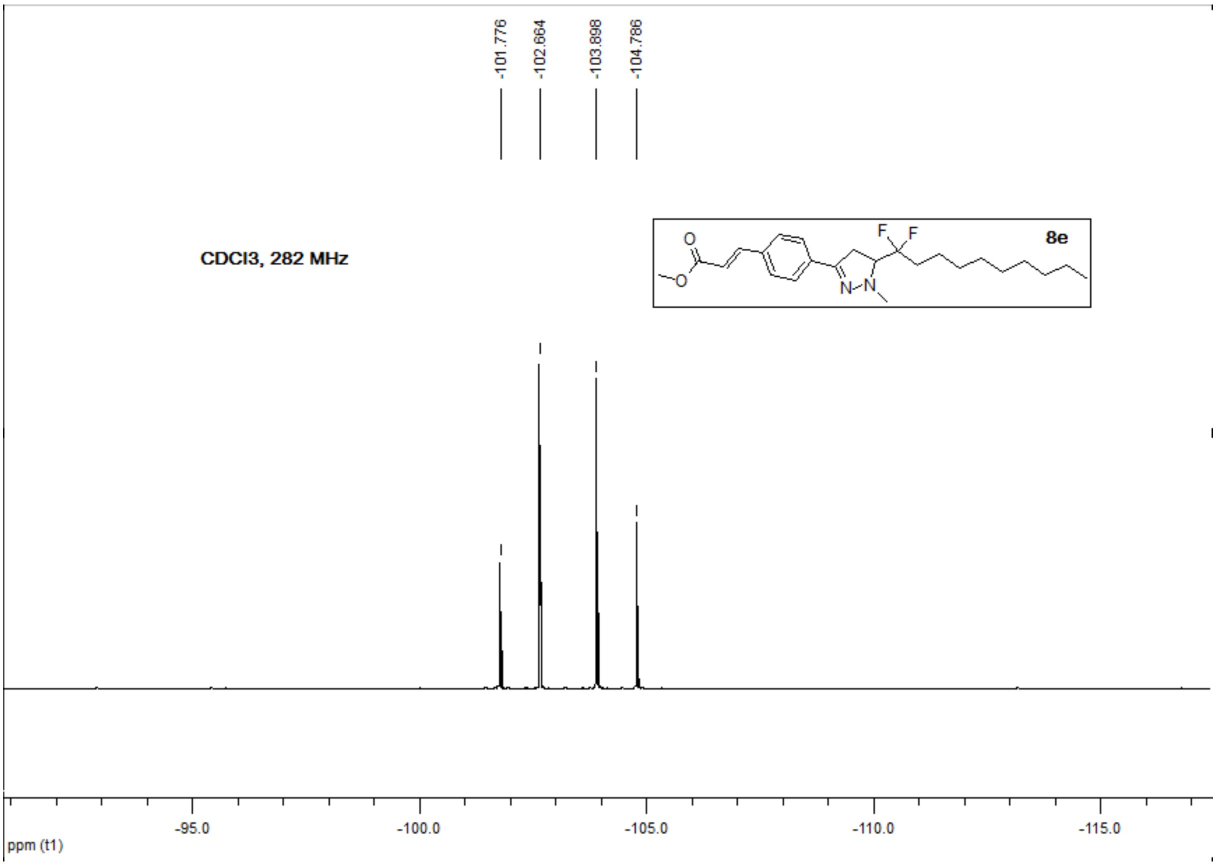
-101.776
-102.664
-103.898
-104.786
CDCl3, 282 MHz
8e
-95.0
-100.0
-105.0
-110.0
-115.0
ppm (t1)

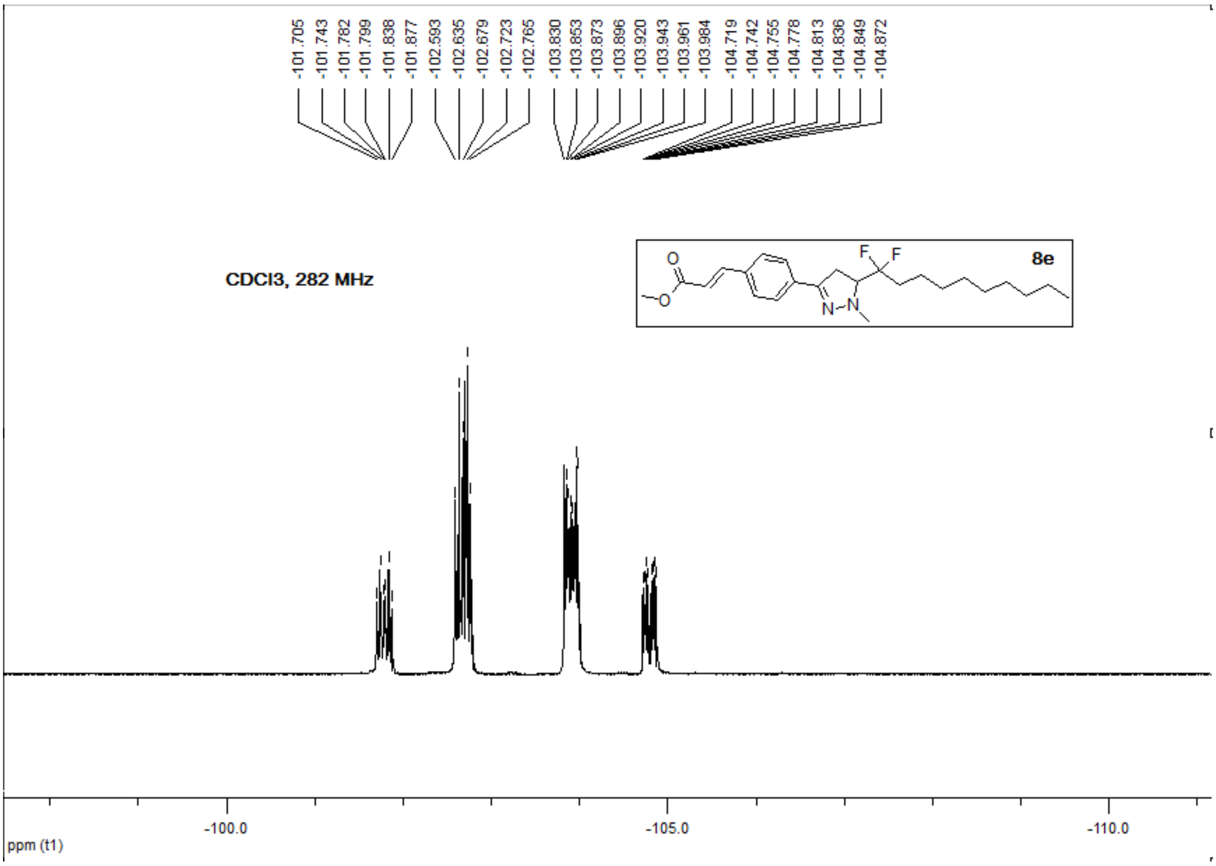
-101.705
-101.743
-101.782
-101.799
-101.838
-101.877
-102.593
-102.635
-102.679
-102.723
-102.765
-103.830
-103.853
-103.873
-103.896
-103.920
-103.943
-103.961
-103.984
-104.719
-104.742
-104.755
-104.778
-104.813
-104.836
-104.849
-104.872
CDCl3, 282 MHz
8e
-100.0
-105.0
-110.0
ppm (t1)

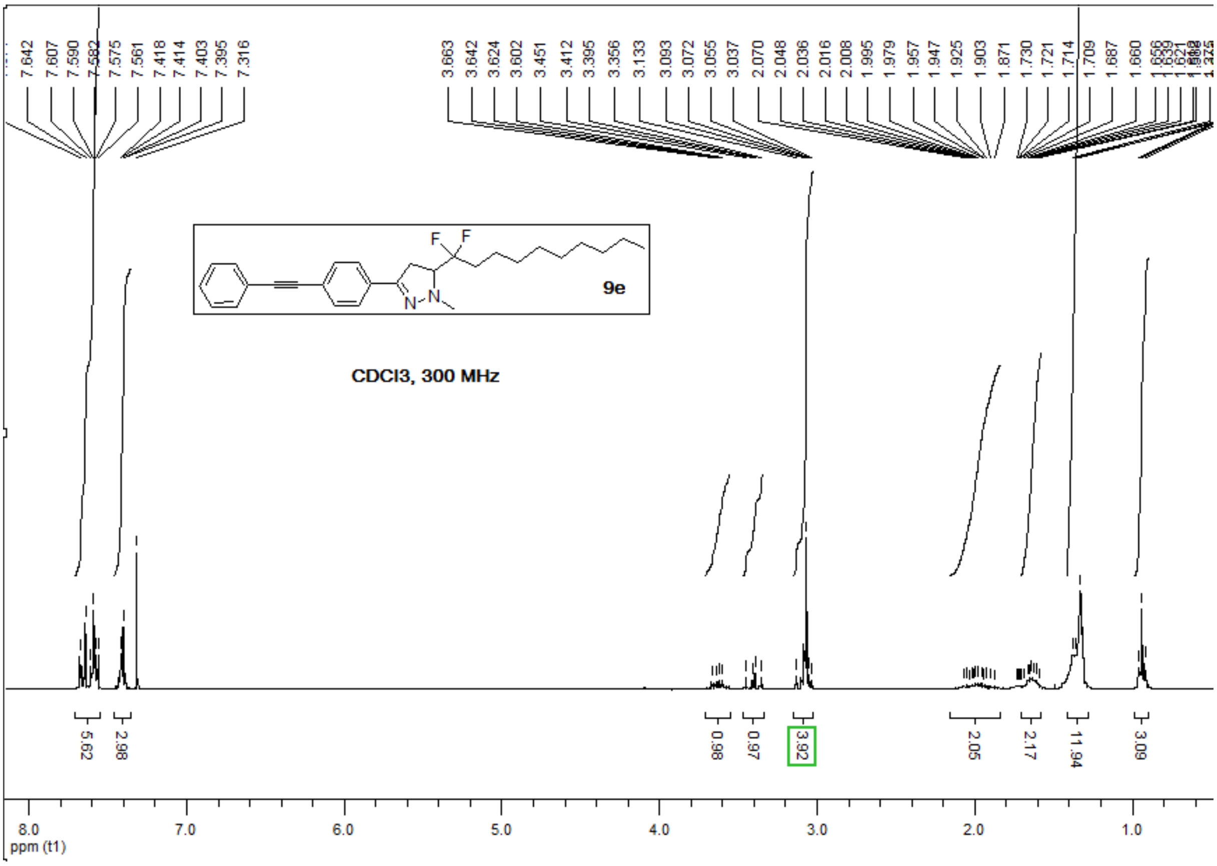

9e
CDCl3, 300 MHz
7.642
7.607
7.590
7.582
7.575
7.561
7.418
7.414
7.403
7.395
7.316
3.663
3.642
3.624
3.602
3.451
3.412
3.395
3.356
3.133
3.093
3.072
3.055
3.037
2.070
2.048
2.036
2.016
2.008
1.995
1.979
1.957
1.947
1.925
1.903
1.871
1.730
1.721
1.714
1.709
1.687
1.660
5.62
2.98
0.98
0.97
3.92
2.05
2.17
11.94
3.09
8.0
7.0
6.0
5.0
4.0
3.0
2.0
1.0
ppm (t1)

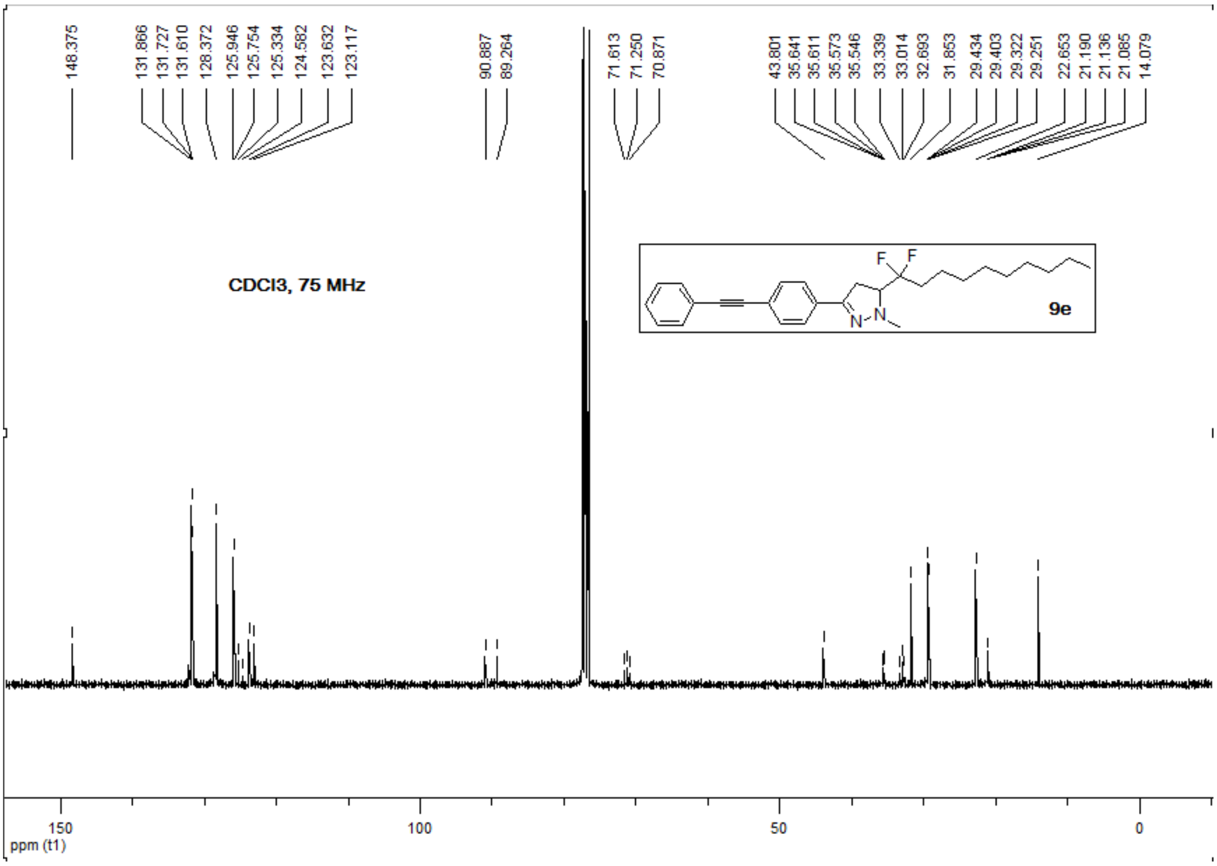

CDCl3, 75 MHz
9e
148.375
131.866
131.727
131.610
128.372
125.946
125.754
125.334
124.582
123.632
123.117
90.887
89.264
71.613
71.250
70.871
43.801
35.641
35.611
35.573
35.546
33.339
33.014
32.693
31.853
29.434
29.403
29.322
29.251
22.653
21.190
21.136
21.085
14.079
150
100
50
0
ppm (t1)

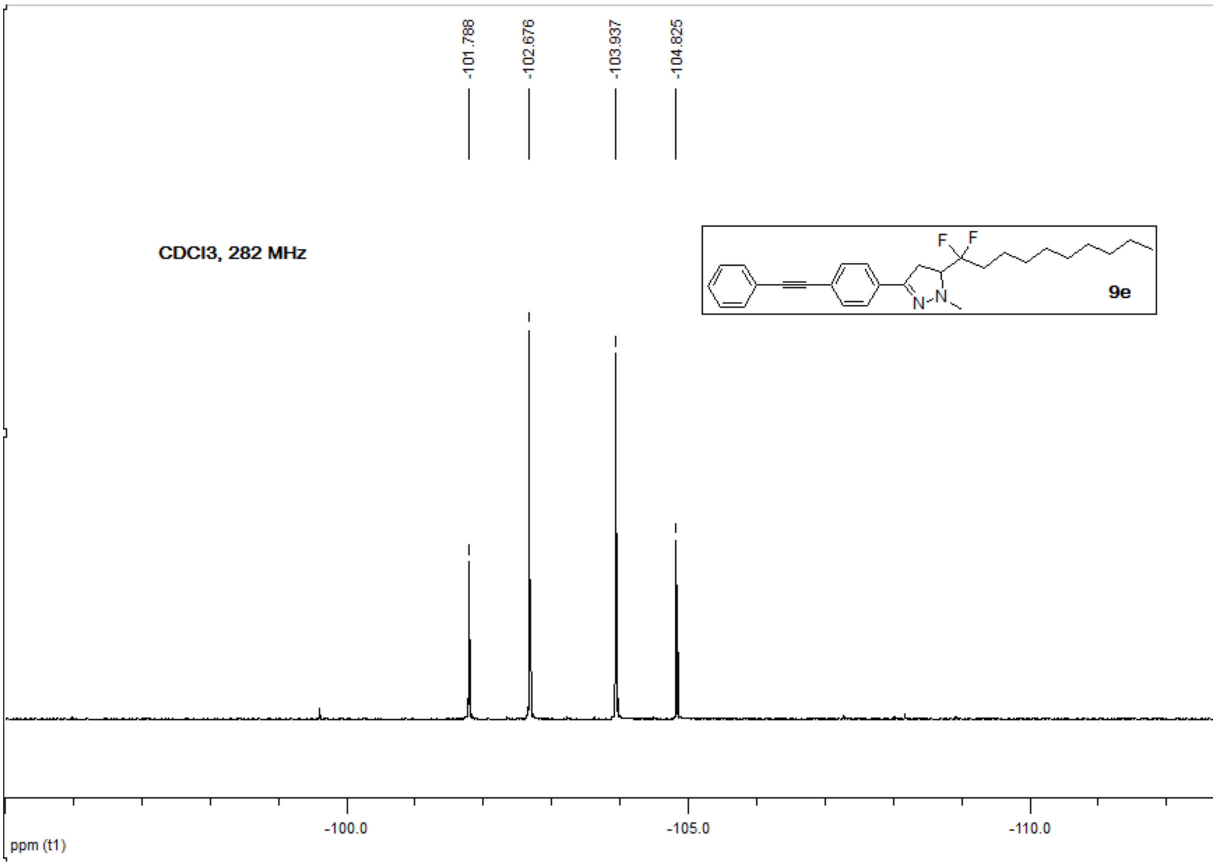
-101.788
-102.676
-103.937
-104.825
CDCl3, 282 MHz
F
F
N
N
9e
-100.0
-105.0
-110.0
ppm (t1)

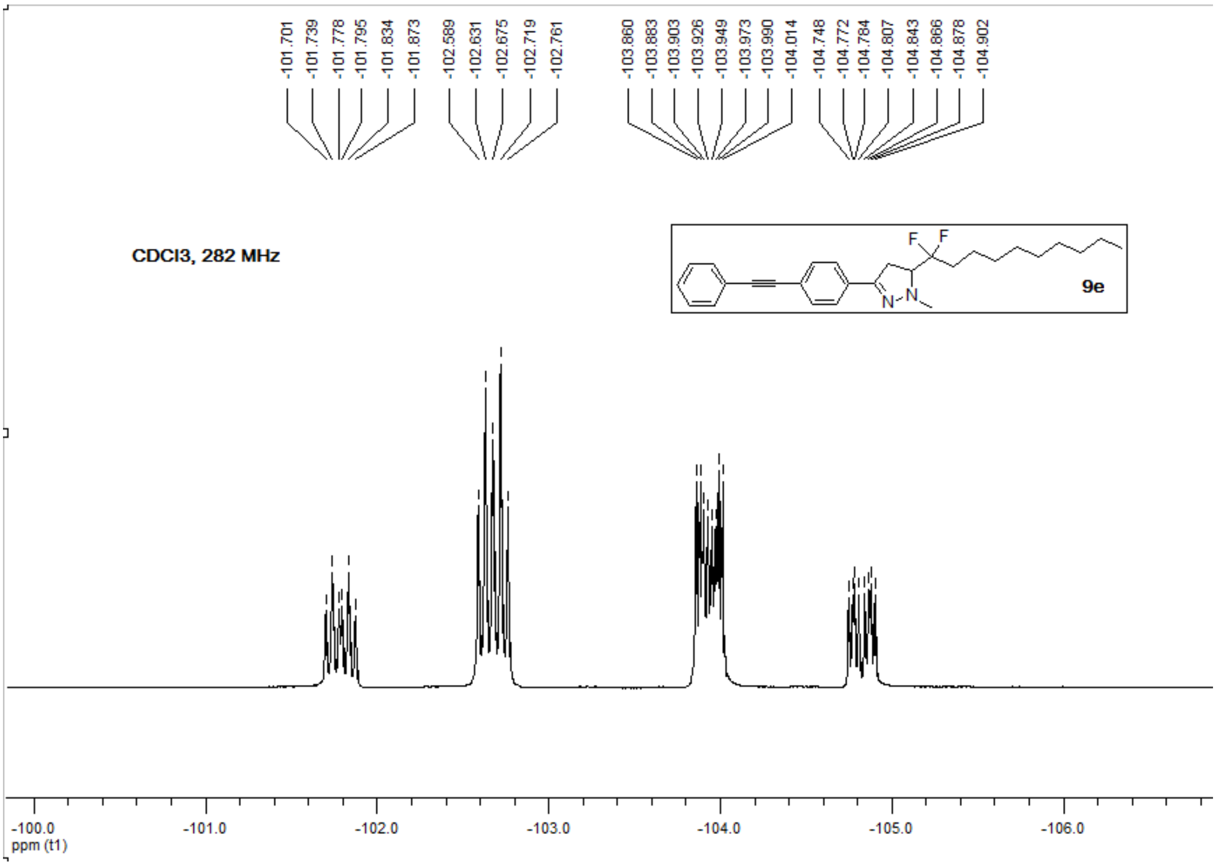
-101.701
-101.739
-101.778
-101.795
-101.834
-101.873
-102.589
-102.631
-102.675
-102.719
-102.761
-103.860
-103.883
-103.903
-103.926
-103.949
-103.973
-103.990
-104.014
-104.748
-104.772
-104.784
-104.807
-104.843
-104.866
-104.878
-104.902
CDCl3, 282 MHz
F
F
N
N
9e
-100.0
-101.0
-102.0
-103.0
-104.0
-105.0
-106.0
ppm (t1)

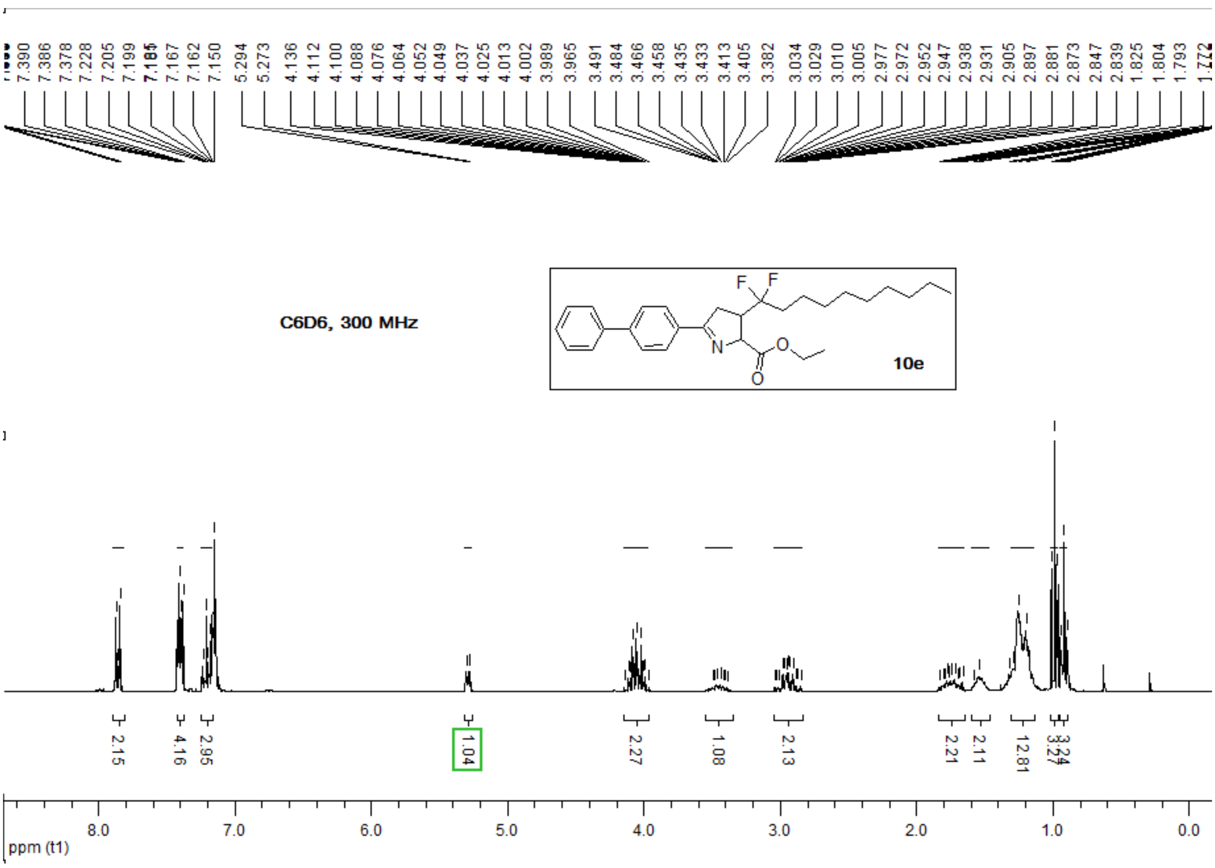
C6D6, 300 MHz
10e
ppm (t1)

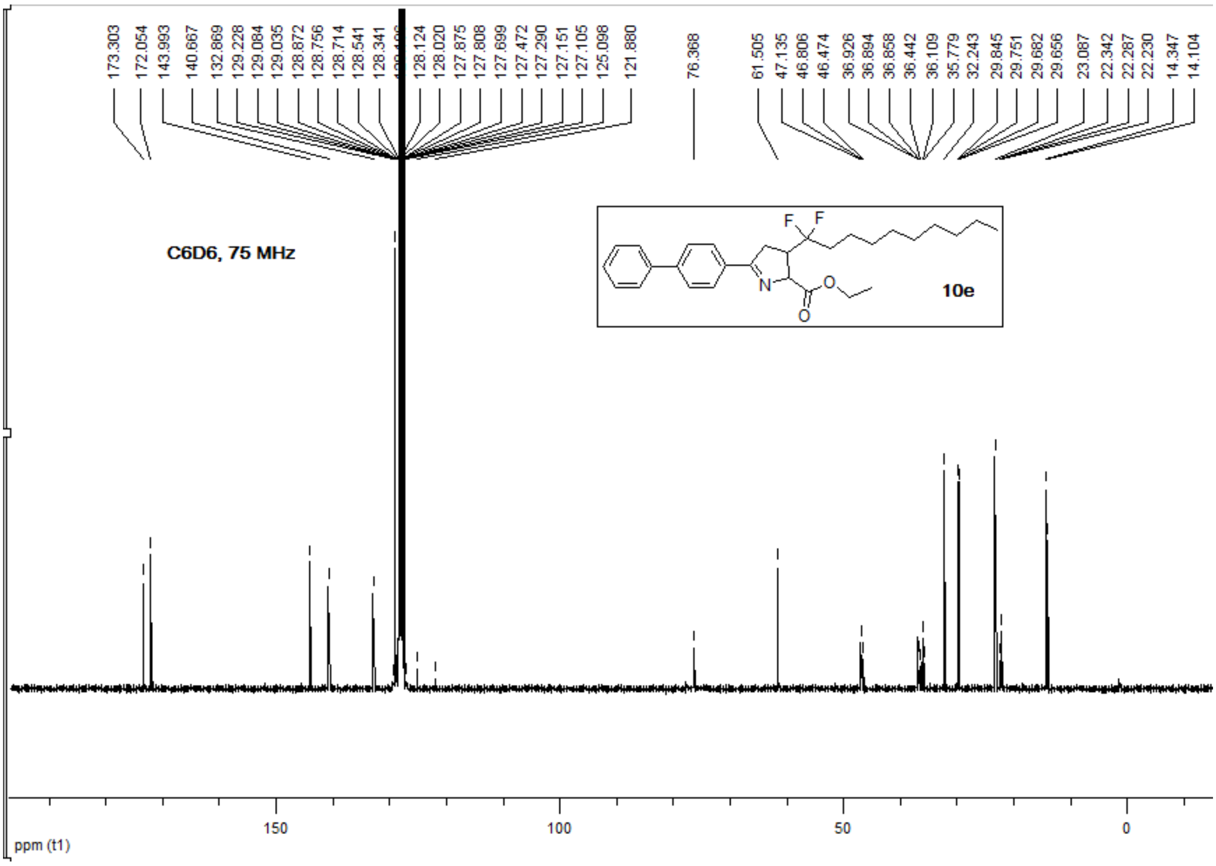
C6D6, 75 MHz
10e
ppm (t1)

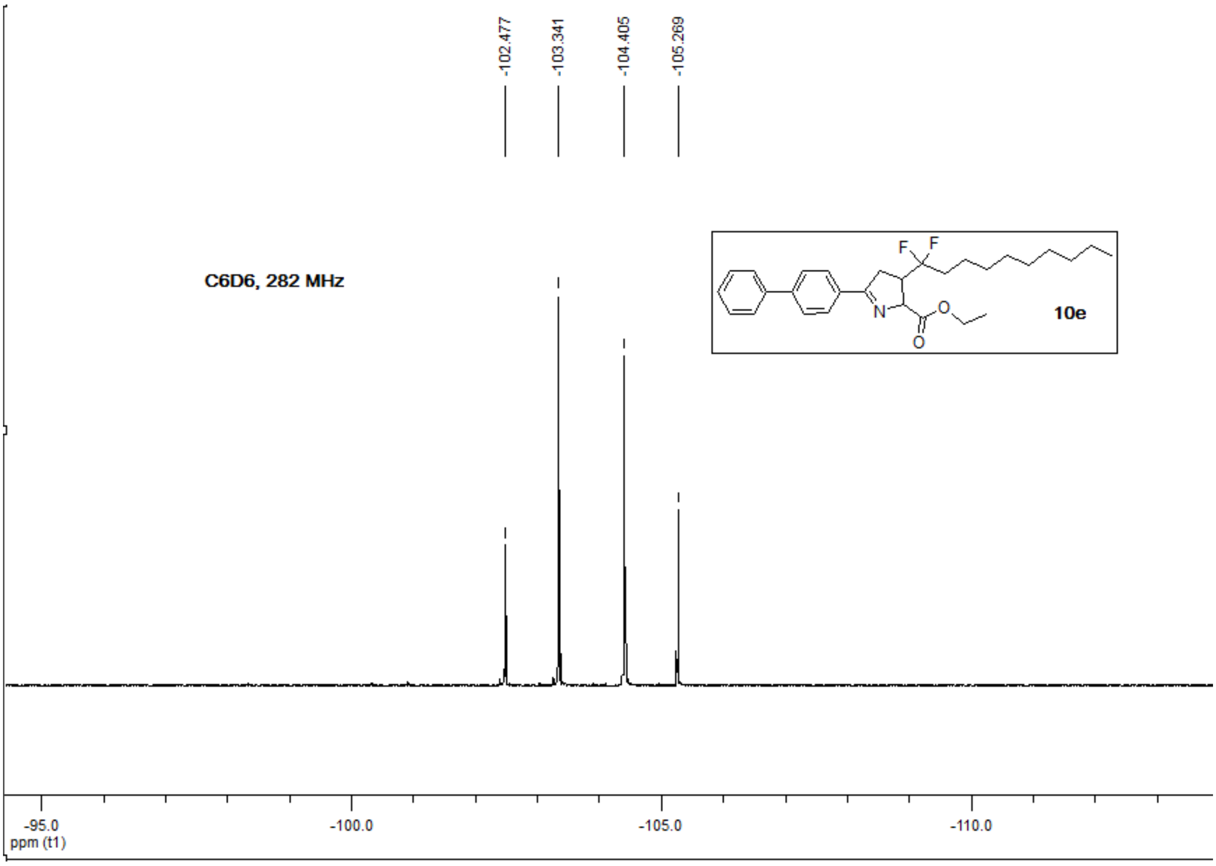
-102.477
-103.341
-104.405
-105.269
C6D6, 282 MHz
F
F
N
O
O
10e
-95.0
ppm (t1)
-100.0
-105.0
-110.0

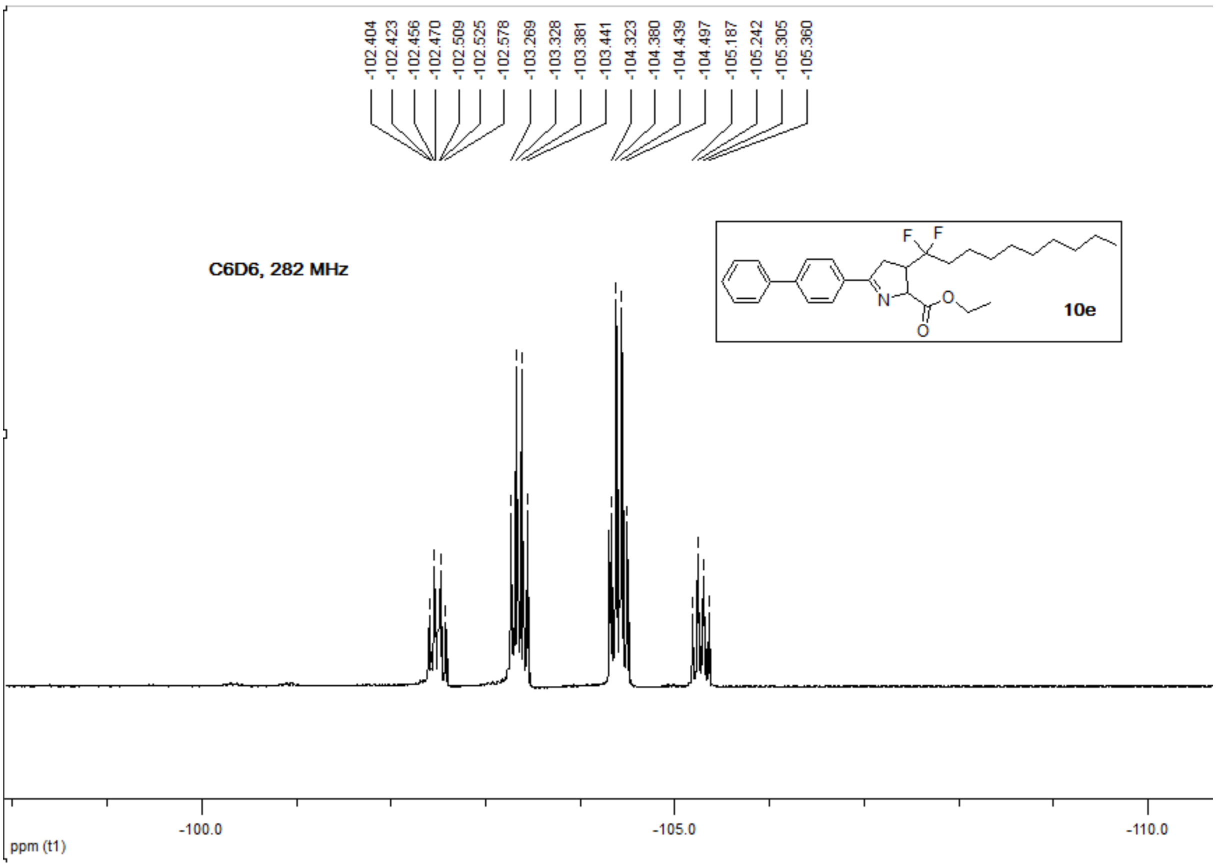
-102.404
-102.423
-102.456
-102.470
-102.509
-102.525
-102.578
-103.269
-103.328
-103.381
-103.441
-104.323
-104.380
-104.439
-104.497
-105.187
-105.242
-105.305
-105.360
C6D6, 282 MHz
F
F
N
O
O
10e
-100.0
-105.0
-110.0
ppm (t1)

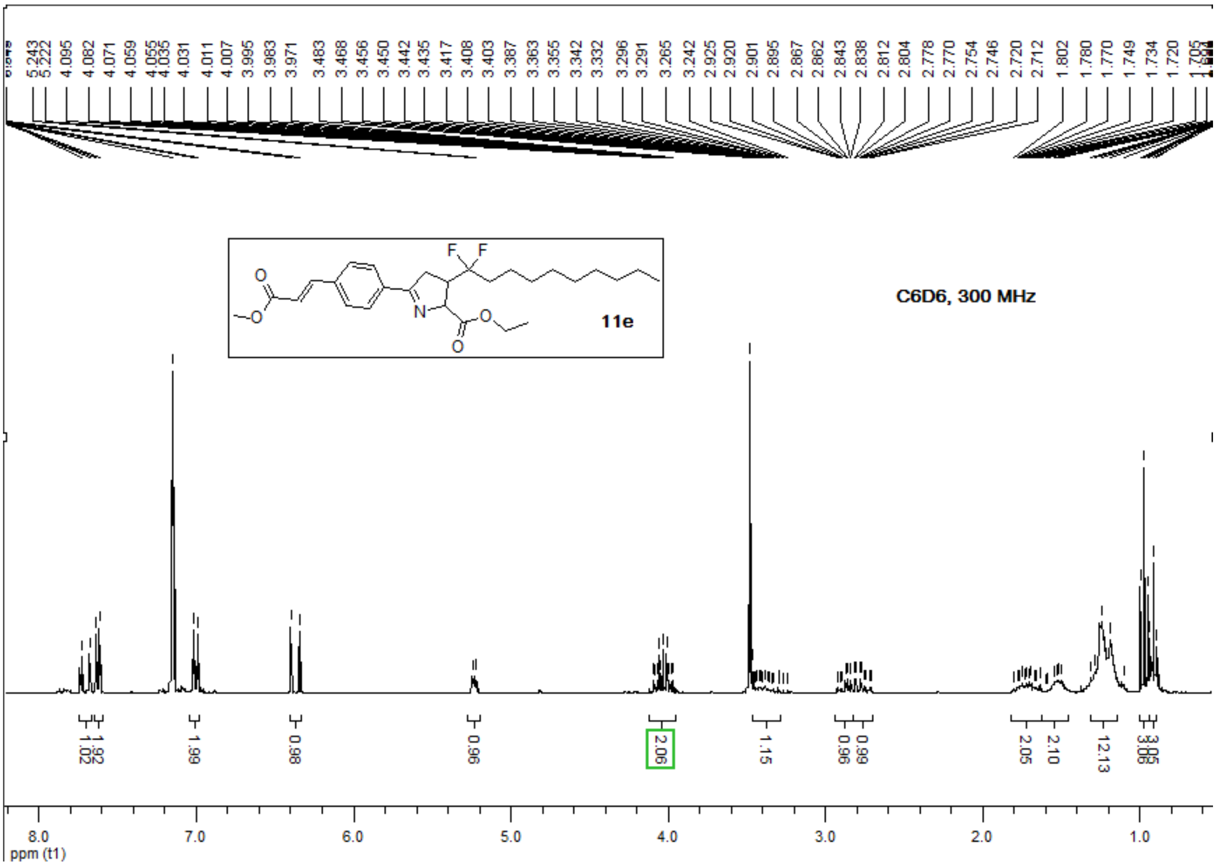

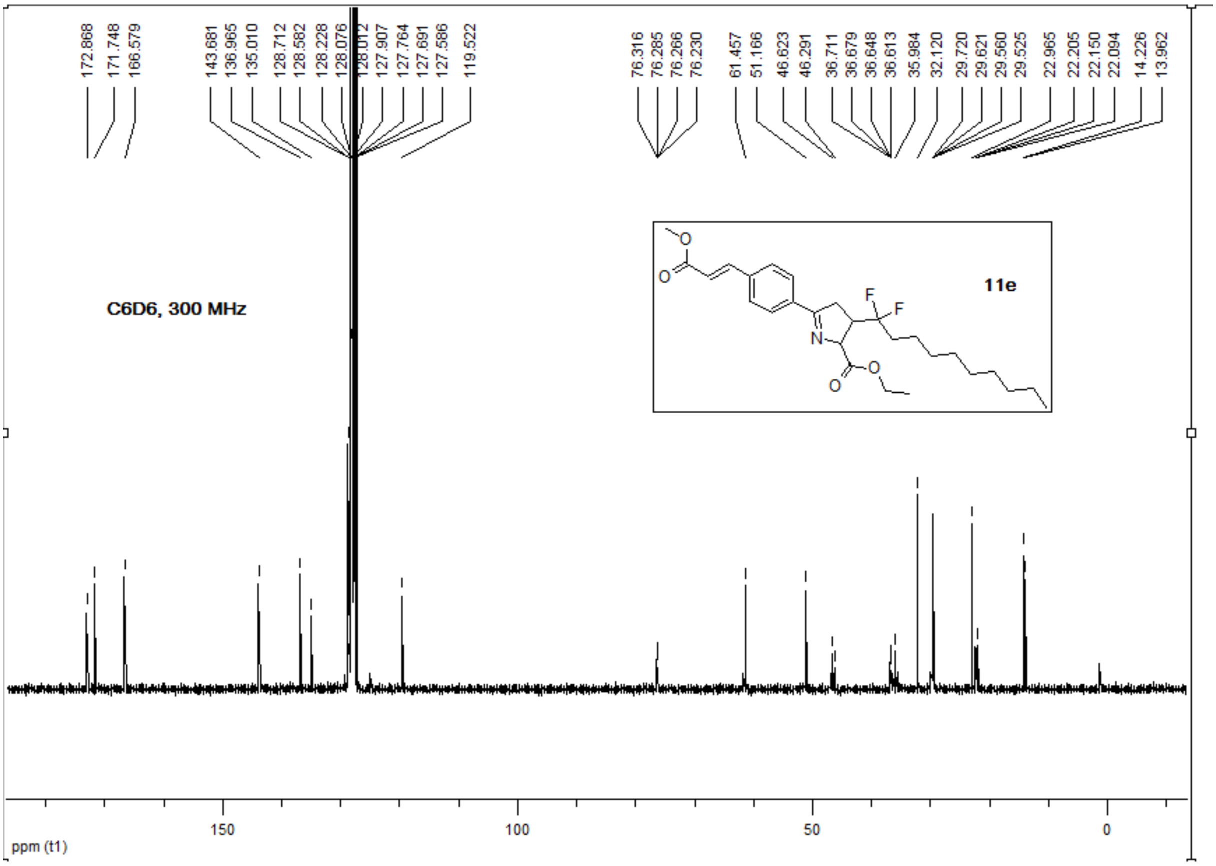

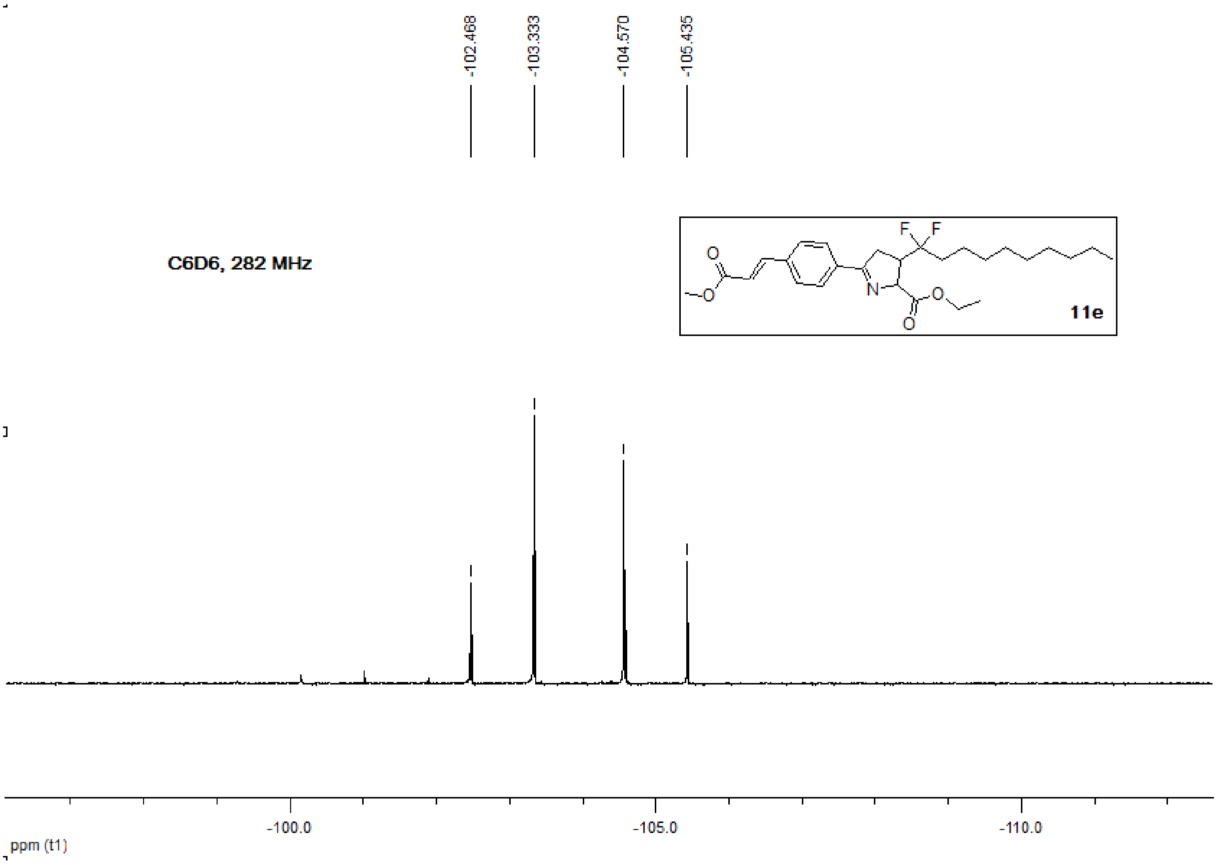
-102.468
-103.333
-104.570
-105.435
C6D6, 282 MHz
11e
-100.0
-105.0
-110.0
ppm (t1)

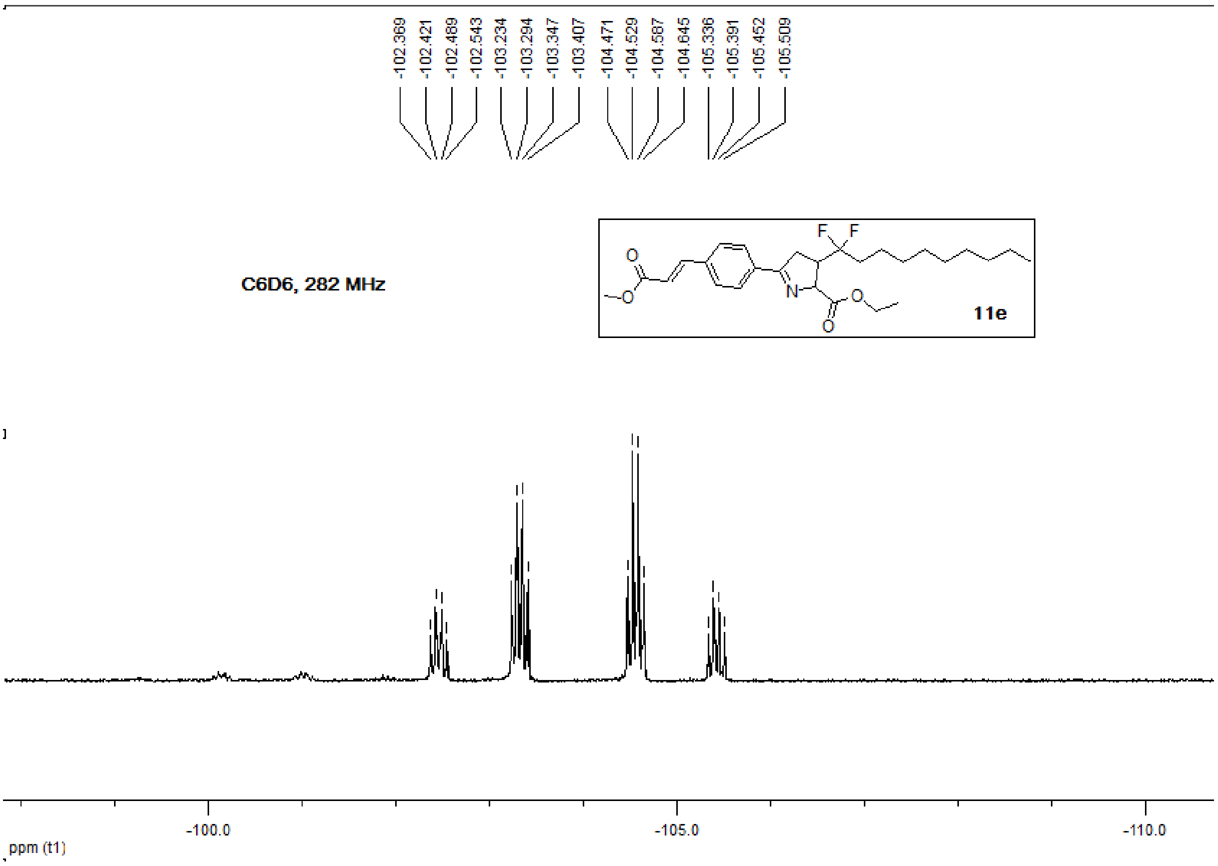
-102.369
-102.421
-102.489
-102.543
-103.234
-103.294
-103.347
-103.407
-104.471
-104.529
-104.587
-104.645
-105.336
-105.391
-105.452
-105.509
C6D6, 282 MHz
11e
-100.0
-105.0
-110.0
ppm (t1)

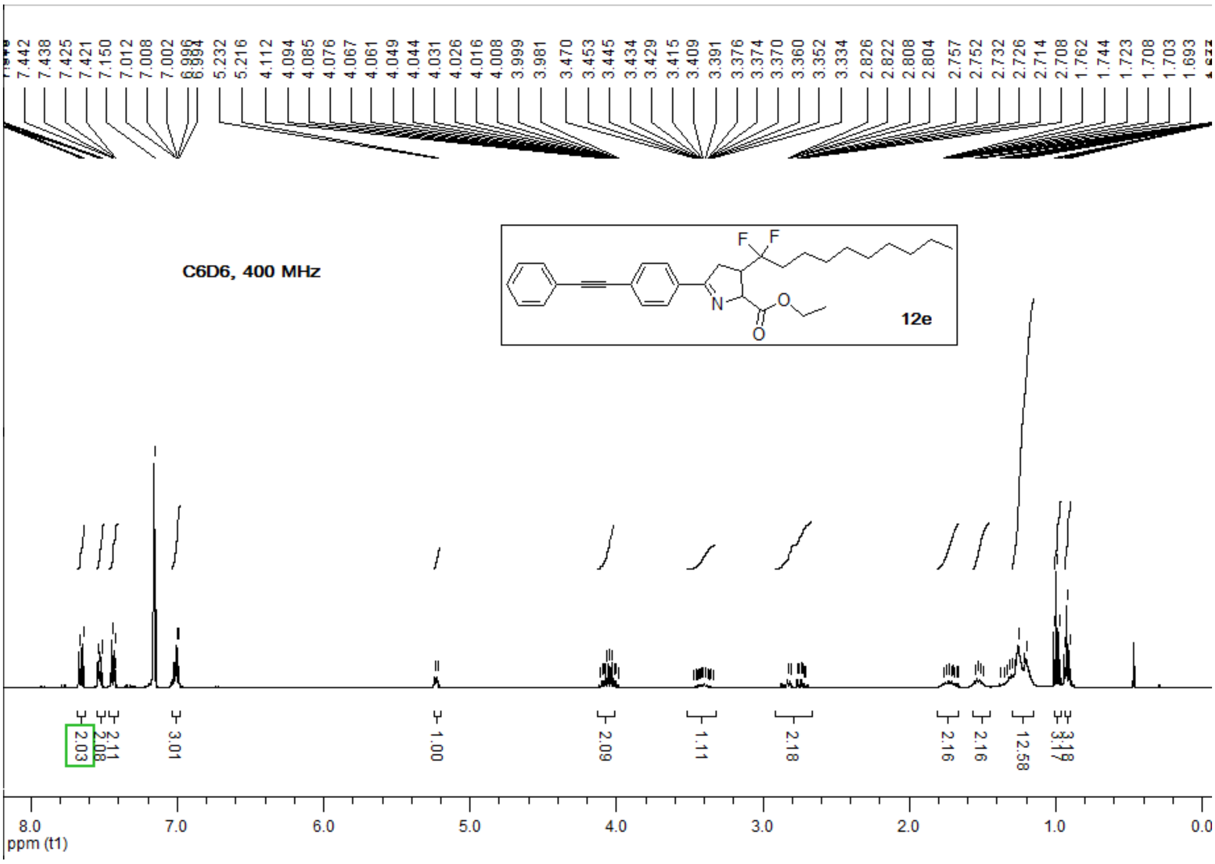
C6D6, 400 MHz
12e
ppm (t1)

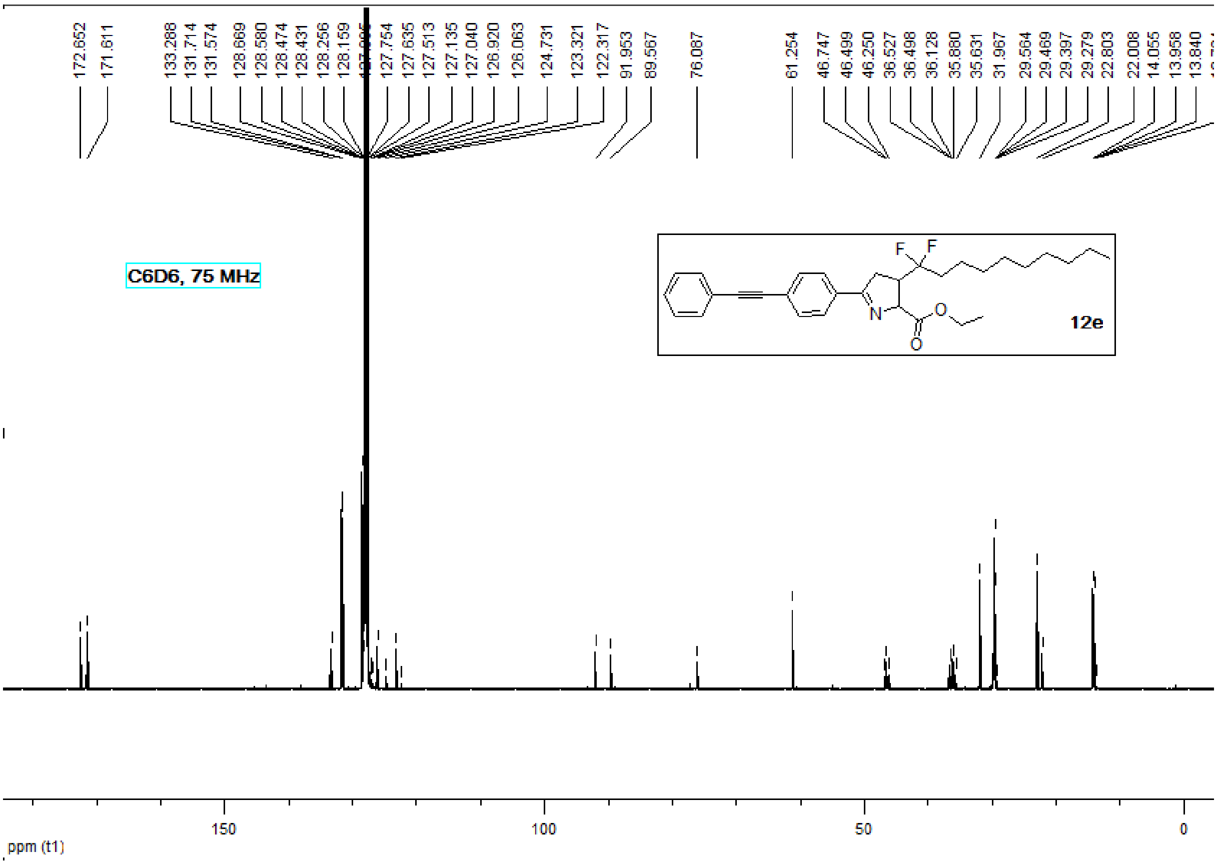
C6D6, 75 MHz
12e
ppm (t1)

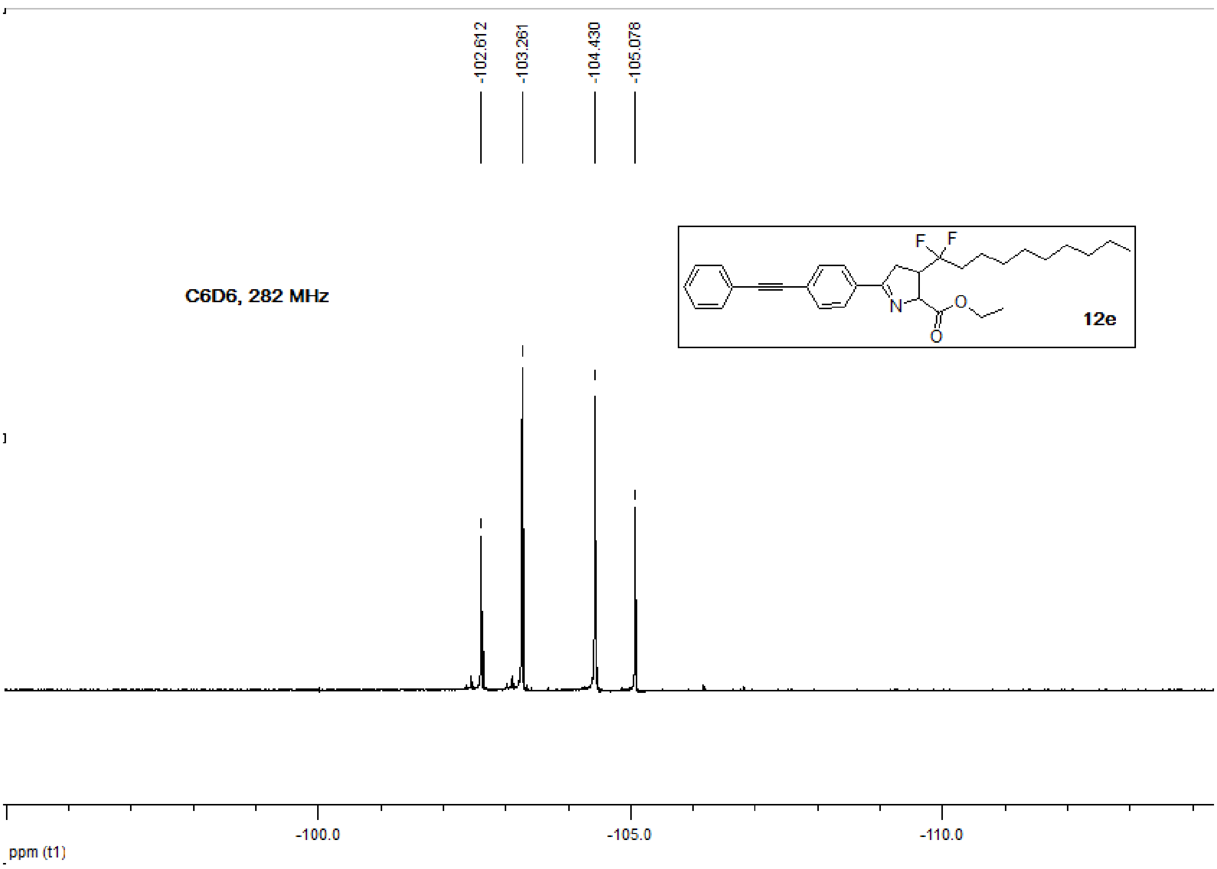
-102.612
-103.261
-104.430
-105.078
C6D6, 282 MHz
F F
N
O
O
12e
-100.0
-105.0
-110.0
ppm (t1)

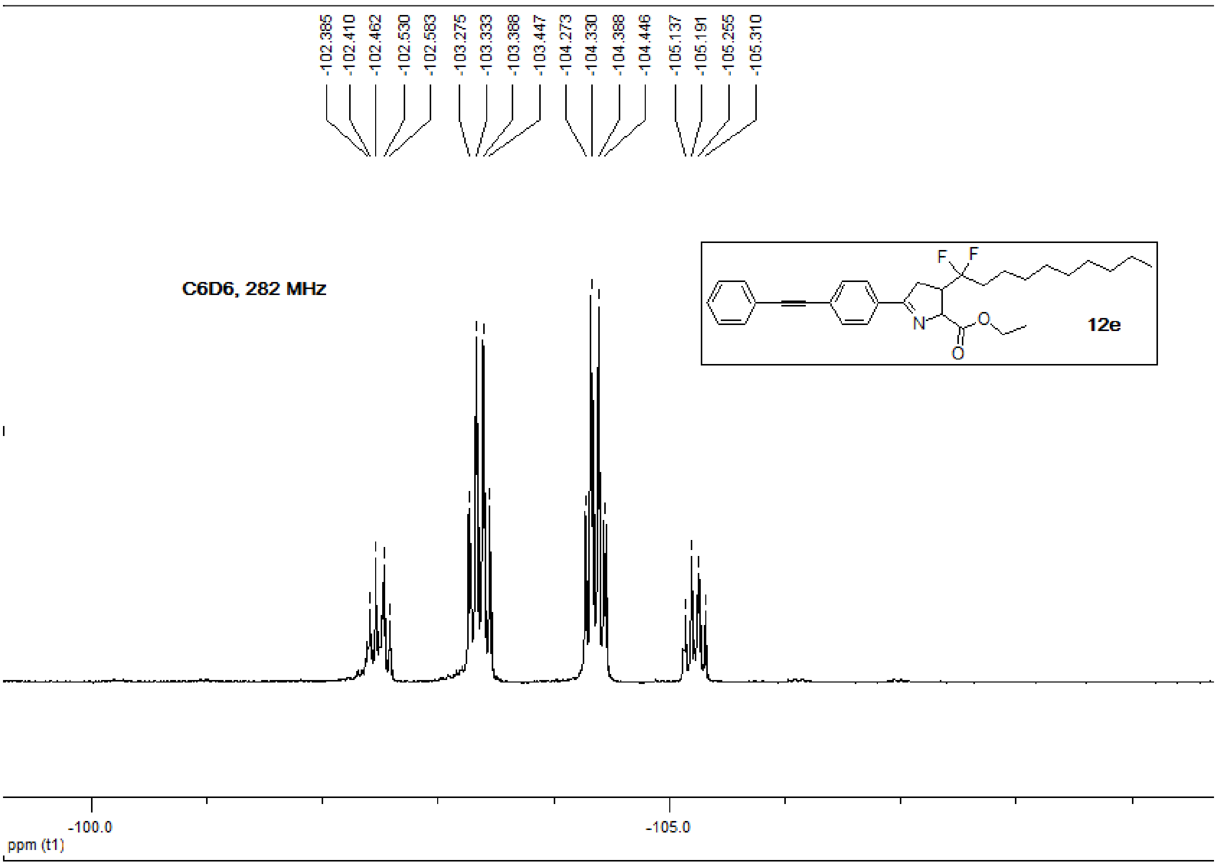
-102.385
-102.410
-102.462
-102.530
-102.583
-103.275
-103.333
-103.388
-103.447
-104.273
-104.330
-104.388
-104.446
-105.137
-105.191
-105.255
-105.310
C6D6, 282 MHz
F F
N
O
O
12e
-100.0
-105.0
ppm (t1)
